# BRInging the Diabetes prevention program to GEriatric populations (BRIDGE): a feasibility study

**DOI:** 10.1186/s40814-019-0513-7

**Published:** 2019-11-11

**Authors:** Jeannette M. Beasley, Lindsey Kirshner, Judith Wylie-Rosett, Mary Ann Sevick, Laura DeLuca, Joshua Chodosh

**Affiliations:** 1 Department of Medicine, NYU Langone Health, 462 First Avenue, 6th Floor, New York, NY 10016 USA; 2 Department of Epidemiology and Population Health, Albert Einstein College of Medicine, 1300 Morris Park 438 Avenue, 1307 Belfer Building, Bronx, NY 10461 USA; 3 Department of Population Health, NYU Langone Health, 227 East 30th Street, 6th Floor, New York, NY 10016 USA; 4 Ferkauf Graduate School of Psychology at Yeshiva University, 1165 Morris Park Ave, Bronx, NY 10461 USA; 5 VA New York Harbor Healthcare System, New York, NY 10016 USA

**Keywords:** Aging, Technology, Translation of evidence-based interventions, Energy balance, Nutrition, Physical activity, Diabetes Prevention Program

## Abstract

**Background:**

The purpose of this 6-week intervention was to test the feasibility and acceptability of implementing a telehealth-adapted Diabetes Prevention Program (DPP) at a senior center.

**Methods:**

Older adults (*n* = 16) attended weekly interactive webinars. At each measurement time point, participants completed questionnaires covering lifestyle, physical activity, quality of life, and food records and wore physical activity trackers. Qualitative data were gathered from 2 focus groups inviting all 16 participants with 13 and 10 participants attending, respectively.

**Results:**

Over 2000 senior center members were contacted, approximately 2% (*n* = 39) responded to the recruitment email, and 16 were recruited into the study. Retention was 75%, and attendance rates averaged 80% across the six intervention sessions. The focus group participants provided positive opinions for most program components, especially the webinar group interaction and using physical activity trackers. Suggestions for improvement included a greater focus on specific needs of older adults (i.e., adapting activities) and placing a greater emphasis on dietary strategies to prevent diabetes. Mean weight loss was 2.9% (2.7 kg [95% CI 1.6, 3.7]; *p* value = 0.001).

**Conclusion:**

The feasibility of providing DPP via webinar appears to be high based on the retention and attendance rates. Similar to other behavioral interventions engaging older adults, recruitment rates were low. Acceptability was evidenced by high attendance at the intervention sessions and feedback from participants during focus group sessions. The intervention efficacy should be evaluated based on CDC criteria for program recognition in a larger scale randomized trial.

**Trial registration:**

NCT03524404. Registered 14 May 2018—retrospectively registered. Trial protocol will be provided by the corresponding author upon request.

## Background

The number of Americans 65 years or older (49.1 million as of 2016) is projected to more than double by 2060 [1]. Obesity among older adults is also increasing, from a prevalence of 31% in 2003–2004 to 35% in 2011–2012 [2]. Without adopting healthier lifestyles, 15–30% of the 87.3 million people in the USA with pre-diabetes will develop type 2 diabetes within 5 years [3]. Despite the availability of effective pharmacological [4, 5] and behavioral interventions [6–11], chronic diseases, such as type 2 diabetes and cardiovascular disease (CVD), remain major public health problems [12].

Among the 20% of participants in the Diabetes Prevention Program (DPP) who were 60 years and older (*n* = 648), the diet and physical activity intervention conferred a 71% reduction in risk of type 2 diabetes [13]. However, few individuals in this age group at risk for type 2 diabetes or CVD adopt healthy diets and lifestyles [14, 15].

Socioeconomically disadvantaged older adults are at particularly high risk of type 2 diabetes and its consequences, and they often do not have ready access to services designed to reduce those risks. In the Northern Manhattan Cohort Study, incidence of type 2 diabetes was twice (HR 2.0; 95% CI 1.2–3.6) as likely among blacks compared to whites [16]. In the National Health Interview Survey, people with type 2 diabetes having the lowest level of education had a 1.52 (95% CI 1.04–2.23) times greater risk of death compared to those with greatest amount of education [17].

As of April 1, 2018, Medicare reimburses for provision of a CDC-approved DPP curriculum [18]; therefore, the intervention is now more accessible to older adults. However, the DPP program is typically offered only at hospitals, YMCA’s, and health departments, limiting accessibility for older adults. Furthermore, current regulations exclude DPP programs offered online, suggesting that more data are needed to support efficacy of online programs. Adaptations of evidence-based lifestyle approaches to chronic disease prevention are needed to meet the needs of the most vulnerable, low-resource communities.

Offering an online program within senior center environments could be an innovative approach to extending the reach of the DPP to low-income seniors. The online format minimizes cost to the senior centers for implementing and administering the program. Nesting the program within senior centers provides built-in supports to reduce barriers to accessing technology.

The primary aim of this study was to test the feasibility of implementing a telehealth adaptation of the DPP within a New York City (NYC) senior center. Measures used to assess feasibility were proportion of responses from emailed invitations, proportion of those screened who were eligible for group meeting attendance, proportion of follow-up visits completed, and obtaining measurements of body weight, diet, and physical activity. Attendance at weekly sessions was used to measure acceptability. Participant focus groups were also used to qualitatively characterize intervention acceptability and feasibility. We hypothesized that the telehealth adaptation would be both feasible and acceptable for older adults within senior centers.

By first examining the feasibility of this DPP telehealth adaptation, our intention is to incorporate feedback from participants to inform the design of an effectiveness trial. As part of our examination of feasibility, we compared (as secondary outcomes) changes in weight, diet, and physical activity after implementing the telehealth program.

## Methods

### Participants

We recruited a purposive sample of older adults in March 2018 by sending emails to over 2000 community center members, which was supplemented by two in-person recruitment sessions at a NYC community center that offers older adults’ programs to build skills in using technology. To align with typical DPP group size and due to space constraints, the targeted sample size was 10–15 participants. Inclusion criteria included being 60 years of age or older and having a Diabetes Risk Score of greater or equal to 5 [19, 20]. The Diabetes Risk Score contains 7 items querying age, gender, history of gestational diabetes, family history, hypertension, physical activity, and weight for a total score ranging from 0 to 20. Those who lacked decisional capacity to consent were also excluded from this study. All participants provided written informed consent. This study was approved by the Institutional Review Board at New York University Langone School of Medicine. Each participant was given a $50 gift card at the conclusion of the follow-up visit.

### Design

This study was a single-group design with measurements collected at baseline and 7 weeks. A certified DPP group facilitator led weekly interactive, live webinars of the first 6 of 12 core sessions of the University of Pittsburgh’s Group Lifestyle Balance 2017 Diabetes Prevention Program (see Additional file 1) [21]. Six sessions were chosen as it was believed to provide sufficient data to assess the feasibility of delivering the intervention via telehealth. The study participants gathered in person at one senior center, while the facilitator joined remotely via FaceTime for weekly hour-long sessions. The session topics included an introduction to the program, tips about eating fewer calories and fat, components of healthy eating, the benefits of and methods for tracking physical activity, and how to manage personal and social eating triggers. A research assistant measured and recorded each participant’s weight at each weekly session. The checklist for the CONSORT extension for pilot trials was completed (see Additional file 2).

### Measurements

#### Primary measures

The proportion of responses from emailed invitations, proportion of those screened who were eligible for group meeting attendance, and proportion of follow-up visits completed were collected and analyzed to assess feasibility and acceptability. Additionally, qualitative data was collected from focus groups and used to better understand participant perceptions of the intervention.

#### Secondary measures

To establish feasibility of using the measures, baseline and follow-up (week 7) measurements included weight (Tanita BC-545, Tokyo, Japan), self-reported physical activity (Cardiovascular Healthy Activities Model Program for Seniors) [24], dietary intake using a 4-day food record, waist circumference, alcohol consumption (AUDIT-C) [22], smoking status (never, current, former), depression (Center for Epidemiologic Studies-Depression Scale) [23], and quality of life (RAND-36) [25, 26]. At baseline, a research assistant collected demographic information and measured subjects’ height and stored all anthropometric, demographic, and lifestyle assessment data in REDCap, a secure online web application (Research Electronic Data Capture) [27]. Physical activity trackers (Fitbit Charge 2™, Fitbit, Inc., San Francisco, CA, 2016) were provided and distributed to all participants after the second session.

### Analysis

#### Qualitative measures

The principal investigator (JMB) led focus groups discussing the program’s acceptability and relevance for all participants in attendance after sessions 3 (*n* = 13) and 5 (*n* = 10). The rationale for conducting two focus groups among the same participants was to gather perspectives after a session on healthy eating at the midpoint and after physical activity had been introduced during sessions 4 and 5. Each focus group took place immediately after the intervention session and lasted approximately 45 min. Table 1 outlines the focus group questions asked at each focus group. Two research assistants recorded responses to the facilitator’s questions. The facilitator and two research assistants independently answered ten post-focus group summary questions and met to discuss the focus group feedback. Qualitative results were analyzed by condensing transcripts from the two research assistants, grouping results by response, and summarizing major themes.

**Table 1:** Focus group questions

• What did you think of the information in the presentations? (Amount of information, length of presentation)	
• What did you like the least (best) about the presentation? (General/amount of information, activity, speaker, time commitment, convenience)	
• For the future, if we had to make one change that would make the presentations better, what could we do to improve the presentations?	
• What are your thoughts on the handouts? (Tracking with materials, helpful)	
• When you think of “Diabetes prevention,” what are some words/phrases that come to mind?	
• We know that you are interested in learning about technology because you are here at Senior Planet. Do you see your peers participating in a technology-based program like this? If not, why?	
• How much of this information was new to you? If it was not new to you, where have you heard the information in the past?	
• Some of you shared ways that you track your food intake. How do you think using a nutrition smartphone application would change your participation in this program?	
• Fitbit has a tool where you can track each other’s activity. Do you see yourself using this to interact with other group members to see their physical activity?	
• If this program were repeated, what would you like to be added?	

#### Quantitative measures

We calculated program participation using the proportion of enrolled participants who attended each intervention session of the 6-week program. We used descriptive statistics to summarize demographics among all participants. To identify possible effect direction and magnitude, we analyzed pre-post differences of continuous scale outcomes using paired *t* tests and pre-post changes in categorical variables using chi-square tests. Caution is warranted in interpreting level of statistical significance of these comparisons, given the small sample size of this study. We report 95% confidence intervals for differences in means.

A research assistant (LK) analyzed food records using Nutrition Data System for Research (NDSR 2016, Nutrition Coordinating Center, University of Minnesota, MN). Self-reported physical activity was calculated by summing the hours per week of all physical activity, with moderate-intensity, exercise-related activities and then multiplying by 60 to convert to minutes per week. Physical activity tracker algorithms classified activity as lightly active, fairly active, or very active. Physical activity tracker data was considered invalid if recorded days were < 600 min, 1440 sedentary minutes, or 0 steps. Food records were considered invalid if < 4 days were recorded.

We conducted sensitivity analyses to compare demographic characteristics between all participants (*n* = 16) with those who completed the follow-up visit (*n* = 12), and to impute missing weight data using the last observation carried forward to assess whether attrition may have influenced inferences. Data analysis was conducted using SPSS (IBM Corp. Released 2015. IBM SPSS Statistics for Windows, Version 23.0. Armonk, NY: IBM Corp.).

## Results

The mean age of participants was 70.1 ± 5.6 years including 12 participants (75%) who were aged 65 or over (Table 2). As shown in Table 2, the sample was largely female, college educated, un-partnered, overweight, or obese. Somewhat more than half of the participants were white.

**Table 2:** Baseline demographics, *n* = 16

Age, mean ± SD	70.1 ± 5.6
Sex, female (%)	11 (68.8)
BMI, kg/m^2^, mean ± SD	31.2 ± 7.3
Normal, 18 to < 25 kg/m^2^ (*n*, %)	3 (18.8)
Overweight, 25 to < 30 kg/m (*n*, %)	6 (37.5)
Obese, ≥ 30 kg/m^2^ (*n*, %)	7 (43.8)
Race (*n*, %)	
White	9 (56.3)
Black or African American	6 (37.5)
Asian	1 (6.3)
Hispanic ethnicity (*n*, %)	1 (8.3)
Marital status (*n*, %)	
Married/living with partner	2 (12.5)
Divorced	6 (37.5)
Widowed	3 (18.8)
Never married	5 (31.3)
Education (*n*, %)	
High school graduate	1 (6.3)
Some college or technical school	5 (31.3)
College graduate	10 (62.5)

### Feasibility and acceptability measures

**Fig. 1:**
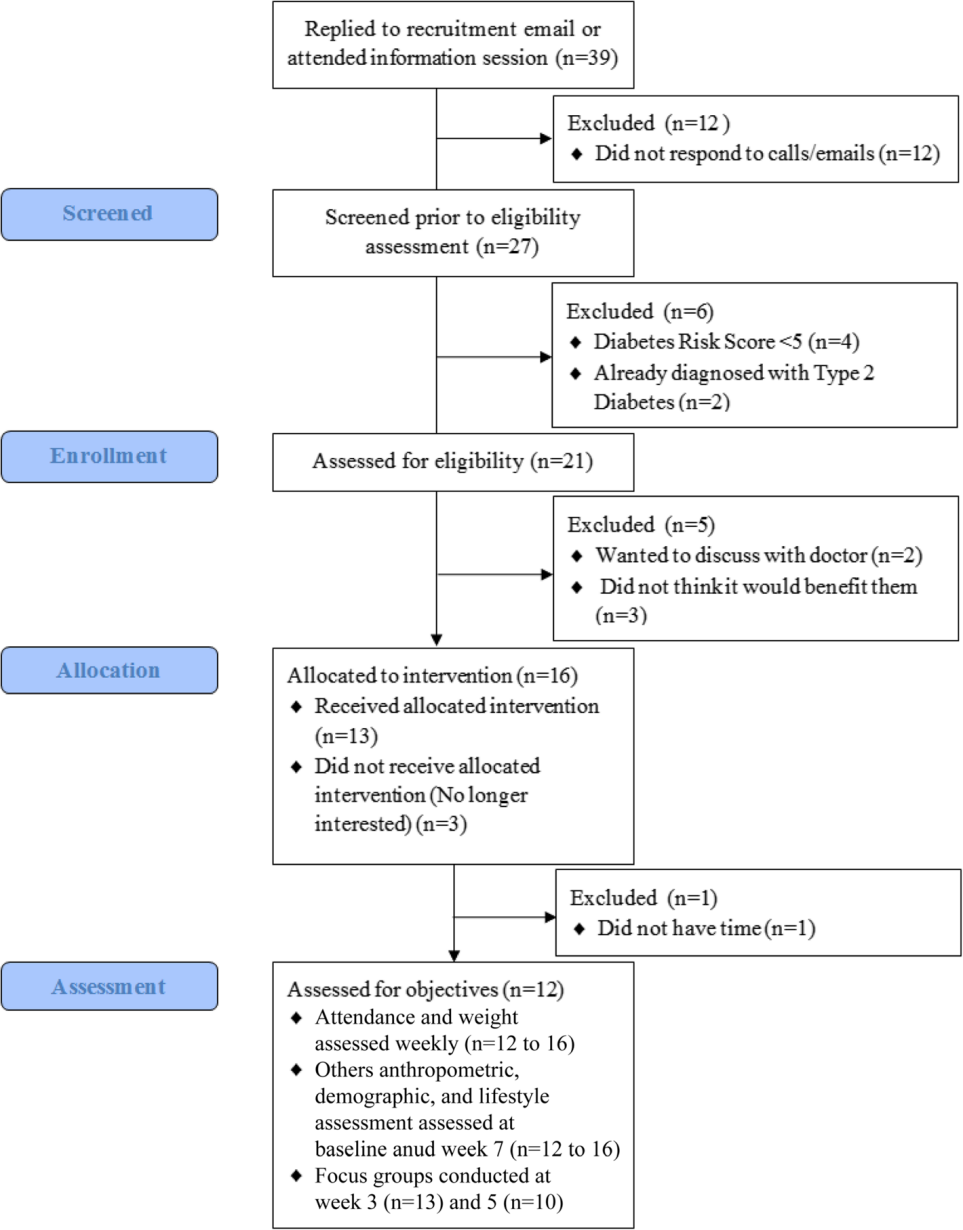
Flow diagram

Figure 1 outlines the flow diagram of participant enrollment. Approximately 2% (*n* = 39) of the over 2000 senior center members responded to the recruitment email. Based on the feasibility outcome measures, 69.2% (*n* = 27) of total participants (*n* = 39) who responded to the email invitation were then screened for the feasibility study. Of the 27 participants screened, 77.8% (*n* = 21) of participants were eligible for group meetings. The number needed to screen to enroll 1 participant was 2.1 (Fig. 1, 27 screened/13 enrolled).

As a measure of program acceptability, retention was 75%, with the majority of participants attending all intervention sessions (Table 3). All participants provided valid food records at baseline, and 12/13 (92%) provided valid food records at follow-up (Table 4). For the physical activity trackers, over 80% of participants provided valid physical activity tracker data (11/13) at week 3 follow-up, and 69% (9/13) provided valid data at week 6 follow-up (Table 5). Focus group attendees (*n* = 13 at the first focus group; *n* = 10 at the second focus group) indicated that the most favorable aspects of the program were interacting with each other, using the physical activity trackers, the resources and worksheets provided, and the session length. Unfavorable aspects of the program included the following: difficulty interacting with the health coach due to ambient noise and poor sound quality through the television, and the lack of in-depth nutritional information presented. Participants wanted “more substance” and “factual information” provided in the facilitated group sessions. A couple of participants requested the sessions be led by a dietitian, so that more advanced information could be provided beyond what is available in the handouts. In particular, program participants wanted more explanation of why reducing fat was the primary focus rather than restricting carbohydrates. Other participants’ suggestions for improvement included adapting the information for older adults by providing modified exercises and reducing environmental distractions.

**Table 3:** Attendance and weight change

	Attendance		Weight, kg (mean ± SD)	Weight change from baseline	
	*n*	%		Mean [95% CI]	%
Baseline	16	100	86.7 ± 23.3		
Week 1	14	87.5	88.3 ± 23.3	− 0.5 [− 1.1, 0.1]	− 0.6
Week 2	14	87.5	90.2 ± 21.0	− 0.4 [− 1.1, 0.3]	− 0.4
Week 3	13	81.3	87.3 ± 20.2	− 1.0 [− 1.9, − 0.1]	− 1.2
Week 4	13	81.3	87.2 ± 19.8	− 1.0 [− 1.5, − 0.5]	− 1.1
Week 5	10	62.5	80.9 ± 15.9	− 1.2 [− 2.4, − 0.1]	− 1.3
Week 6	13	81.3	86.2 ± 19.0	− 2.0 ± [− 2.9, − 1.1]	− 2.2
Follow-up (Week 7)	12	75.0	85.0 ± 19.5	− 2.7 ± [− 3.8, − 1.6]	− 2.9

**Table 4:** Diet quality, daily mean ± SD

	Baseline (*n* = 13)	Follow-up (*n* = 12)	Change (mean [95% CI])
Calories, kcal	1338 ± 443	1306 ± 496	− 32 [− 338, 274]
Total fat, g	55 ± 27	51 ± 26	− 5 [− 19, 9]
Saturated fat, g	18 ± 12	15 ± 10	− 3 [− 9, 3]
Trans fat, g	1 ± 1	1 ± 2	0 [− 1, 1]
Cholesterol, mg	232 ± 125	258 ± 171	18 [− 49, 85]
Sodium, mg	2225 ± 697	2056 ± 1405	− 185 [− 986, 616]
Total carbohydrate, g	151 ± 43	156 ± 51	4 [− 30, 38]
Dietary fiber, g	18 ± 8	18 ± 6	− 1 [− 5, 3]
Total sugars, g	56 ± 21	58 ± 15	− 3 [− 17, 11]
Added sugars	25 ± 11	25 ± 13	− 2 [− 12, 8]
Total protein, g	63 ± 20	62 ± 26	1 [− 16, 18]
Vitamin D, mcg	5 ± 5	6 ± 4	3 [0,6]
Calcium, mg	643 ± 439	583 ± 273	− 77 [− 287, 133]
Iron, mg	14 ± 6	10 ± 4	− 4 [− 8, 0]
Potassium, mg	2093 ± 746	2281 ± 711	141 [− 307, 589]
Total fruits, servings^a^	1.6 ± 1.0	1.9 ± 1.2	0.2 [− 0.4, 0.8]
Whole fruits, servings^b^	1.4 ± 1.1	1.6 ± 1.3	0.1[− 0.5, 0.7]
Vegetables, servings^c^	3.1 ± 2.4	3.6 ± 1.9	0.3 [− 0.7, 1.3]

None of the changes were statistically significant (all *p* > 0.05)

^a^Total fruit: 100% citrus juice, 100% fruit juice excluding citrus juice, citrus fruit, fruit excluding citrus fruit, avocado and similar

^b^Whole fruit: citrus fruit, fruit excluding citrus fruit, avocado and similar

^c^Vegetables: dark-green vegetables, deep-yellow vegetables, tomato, white potatoes, other starchy vegetables, legumes, other vegetables, 100% vegetable juice

**Table 5:** Self-reported and objective physical activity, mean ± SD

	Minutes per week		Change
	Baseline (*n* = 16)	Follow-up (*n* = 12)	
Self-reported			
Moderate-intensity exercise-related activities	330 ± 301	395 ± 323	66 [− 178, 310]
All exercise-related activities	1043 ± 526	1170 ± 458	127 [− 264, 518]
	Week 3 (*n* = 11)	Week 6 (*n* = 9)	
Physical activity tracker			
Lightly active	175 ± 57	168 ± 47	− 10 [− 55, 35]
Fairly active	20 ± 17	23 ± 17	− 2 [− 13, 9]
Very active	29 ± 23	30 ± 21	− 3 [− 4, − 2]
Total activity	224 ± 75	221 ± 56	− 15 [− 76, 46]

None of the changes were statistically significant (all *p* > 0.05)

### Secondary measures

We were able to collect measurements of weight, dietary intake, and physical activity among participants who attended the intervention sessions. At follow-up, mean weight loss of participants was 2.7 ± 1.9 kg (2.9%) (Table 3). Imputing missing weight data did not substantively change weight change estimates (2.3 ± 1.9 kg or 2.4% weight loss after imputation).

There were no significant differences in self-reported energy, nutrients, or fruit and vegetable consumption (Table 4).

Similarly, although no significant changes in pre-post physical activity were reported (Table 5), participants completed 66 (95% CI − 178, 310) min more of moderate-intensity exercise-related activities from baseline to follow-up, and 127 (− 264, 518) min more of all exercise-related activities (Table 5). The Fitbits recorded greater intensity at follow-up, but overall minutes of activity did not increase from baseline to follow-up.

## Discussion

To our knowledge, this is the first feasibility study testing telehealth delivery of the DPP in a senior center. We demonstrated feasibility through our ability to recruit the target sample and deliver the 6-week program. Program acceptability was high, with 75% of participants completing all six sessions, and valid self-reported diet, objective physical activity, and weight information obtained from the majority of participants. Secondary outcomes demonstrated a significant weight change of 2.9% at a rate of 0.2 to 0.5 kg per week over the 6-week intervention period. Suggestions for improvement included improving the sound quality of the connection between the health coach and participants, increasing the amount and depth of nutrition information presented, and adapting materials for older adults. Participants’ suggestions will be incorporated into the development of a larger scale study with sufficient power to assess the effectiveness of a telehealth adaptation of a year-long DPP intervention.

The seminal DPP trial demonstrated that lifestyle intervention reduced diabetes incidence by 58% (95% CI 48 to 66%) [13]; since then, the intervention has been implemented in hospital, community, workplace, and other settings [28]. In a study by West et al., the DPP program administered by lay health workers in senior centers resulted in a greater percentage of weight loss compared to control senior centers (38% of intervention group participants lost ≥ 5% of weight versus 5% among controls, *p* < 0.001) after 4 months [29]. The West et al. study had a high retention (83% attended at least 50% of sessions) [29] at an average cost of over $2731 per community center or $165 per participant [29, 30]. Medicare currently reimburses up to $690 per DPP participant achieving at least 5% weight loss and $190 for beneficiaries that do not meet the minimum weight loss goal [31]. However, 7 years after the West et al. study was completed, fewer than 10 of the 1452 nationwide DPP programs are based in senior centers.

Despite low penetrance of DPP programs, senior centers are well attended and remain a viable option. Congregate meals are enormously popular, with 95% of seniors in a nationwide survey indicating they would recommend them to a friend [32]. This highlights the importance of our feasibility findings and the opportunity for an increase of telehealth adaptations of the DPP in senior centers. Of the five senior centers we have partnered with in New York City, all but one have the resources available to conduct a telehealth adaptation of the DPP in their center.

There were several strengths of this feasibility study, including testing a proof of concept for an approach with broad scalability, fostering academic and community partnerships, and measuring quantitative and qualitative outcomes. Limitations of our study include a small sample size that was underpowered to detect changes in secondary outcomes and recruiting at one senior center already utilizing technology with older adults, who may be predisposed to have an interest and/or increased knowledge in technology use. Similar to other behavioral interventions that engage older adults, recruitment rates were very low [33, 34]. The 6-week intervention period was abbreviated from the year-long DPP intervention; however, the average number of DPP sessions attended in a large healthcare organization is six [35]. The space at this particular senior center was limited with frequent technology/connection difficulties, which may have impacted our ability to deliver the intervention. The low response rate from our initial outreach email suggests that alternative recruitment strategies would be necessary to achieve enrollment targets for a larger trial. At the recommendation of a senior center director, we are using recruitment strategies by telephone for our next study. Furthermore, the Diabetes Risk Screener was designed for physicians to identify people who may need testing for type 2 diabetes and not as a tool for referral to an intervention. As a result, three participants in the normal weight category met eligibility criteria due to age and other type 2 diabetes risk factors. Opportunities for improvement include environmental modification to accommodate a high prevalence of hearing loss that inhibits communication, tracking participants’ contributions to focus groups, so that changes in viewpoints could be assessed over time, allowing for greater flexibility for participants to return for follow-up appointments, and replacing the Diabetes Risk Score with blood glucose measures as the criteria for prediabetes. There were no significant differences in diet and physical activity measures despite significant weight loss, suggesting these measures may better serve as process, rather than outcome measures.

## Conclusion

This study demonstrated the feasibility and acceptability for delivering an evidence-based intervention using telehealth among older adults. Results showed that telehealth adaptations are feasible for older adults and may be effective given the 2.9% weight loss at a rate of 0.2 to 0.5 kg per week over the 6-week intervention period. Moreover, this would meet the DPP goal of 5% weight loss if a larger program was sustained. Before evaluating a Medicare-reimbursable 12-month DPP intervention model, we are partnering with social support services such as the home-delivered meal program to investigate the feasibility of delivering a telehealth adaptation of the DPP within this population. This approach could provide an alternative strategy for disseminating information to improve health and reduce disease burden among aging populations.

## Supplementary information

**Additional file 1.** Diabetes Prevention Program Group Lifestyle Balance™.

**Additional file 2.** CONSORT 2010 checklist of information to include when reporting a pilot or feasibility trial*.

## Data Availability

The datasets used and/or analyzed during the current study are available from the corresponding author on reasonable request.

## Abbreviations

BMI: Body mass index
BRIDGE: BRInging the Diabetes prevention program to GEriatric populations
CDC: Centers for Disease Control and Prevention
CVD: Cardiovascular disease
DPP: Diabetes Prevention Program
NYC: New York City
REDCap: Research Electronic Data Capture
SD: Standard deviation
