# Supplementary material for: BRInging the Diabetes prevention program to GEriatric populations (BRIDGE): a feasibility study
Source: Pilot Feasibility Stud. 2019 Nov 11;5:129. doi: 10.1186/s40814-019-0513-7 (PMC6849183; doi:10.1186/s40814-019-0513-7)
Supplement: Supplementary file 1 — Additional file 1. Diabetes Prevention Program Group Lifestyle Balance™. [file 40814_2019_513_MOESM1_ESM.pdf]

Diabetes Prevention Program **Group Lifestyle Balance™**

DIABETES PREVENTION PROGRAM
GROUP LIFESTYLE BALANCE™

A Modification of the Diabetes Prevention Program's
Lifestyle Change Program

© 2008; 2010; 2011; 2017, by the University of Pittsburgh.*

Developed by the Diabetes Prevention Support Center faculty of the University of Pittsburgh (Kaye Kramer, Andrea Kriska, Trevor Orchard, Linda Semler, and Elizabeth Venditti), and Bonnie Gillis, MS, RD, with funding support from National Institutes of Diabetes and Digestive and Kidney Diseases, National Institutes of Health & United States Air Force administered by the U.S. Army Medical Research Acquisition Activity, Fort Detrick, Maryland, Award Number W81XWH-04-2-0030. Review of materials does not imply Department of the Air Force endorsement of factual accuracy or opinion.

* The Diabetes Prevention Program GROUP LIFESTYLE BALANCE™ (DPP GLB) manual and materials are made available to the public subject to the following Creative Commons License: Creative Commons - Attribution - ShareAlike 4.0 International. Accordingly, the manual and materials may be downloaded, duplicated, transmitted and otherwise distributed for educational or research purposes, as well as commercially, provided proper credits are given to the University of Pittsburgh Diabetes Prevention Support Center and the Diabetes Prevention Program research team. In addition, you must provide a link to the license and also indicate if any changes were made to the materials. If you remix, transform, or build upon the materials, you must distribute your contributions under the same license as the original.
For further information on use of the DPP GLB manual or materials, contact the University of Pittsburgh's Office of Technology Management at 412-648-2206.

Acknowledgments

The Diabetes Prevention Program

The Group Lifestyle Balance™ Program is based on the highly successful lifestyle intervention used in the Diabetes Prevention Program (DPP). The DPP was conducted at 27 health centers in the United States and was funded by the National Institutes of Health.

More than 3,000 adults took part in the DPP. All were at high risk for Type 2 diabetes. About half of them were African Americans, Hispanics, American Indians, Asians, or Pacific Islanders. Diabetes is very common in those groups. Two out of ten were 60 years old or older, another high-risk group. The average starting weight was 207 pounds.

Each person was assigned by chance to one of three groups.

- One group took part in a program to lose weight and be more active.
- One group took metformin, a pill used to treat diabetes.
- One group took a placebo or sugar pill.

The DPP was a great success. The volunteers were followed for about 3 years.

- Taking metformin cut the risk of Type 2 diabetes by 31 percent.
- The program to lose weight and be more active was even more successful.
- **Losing 10 to 15 pounds and being moderately active (similar to a brisk walk) for a minimum of 150 minutes per week cut the risk of Type 2 diabetes by 58% (more than half).**
- **For those over 60, it cut the risk by 71% (almost three-quarters).**

As a result of the DPP, many international, national, state, and local groups have begun programs like this one to help people lose weight and be more active.

DPP Staff

As mentioned above, the Group Lifestyle Balance Program is based on the highly successful lifestyle intervention used in the Diabetes Prevention Program (DPP). The DPP lifestyle intervention was developed and authored by the Lifestyle Resource Core, working in close collaboration with the DPP Lifestyle Advisory Group and other members of the DPP Interventions Subcommittee. Invaluable feedback and contributions were made by the 27 DPP clinical centers and the Lifestyle Coaches. The procedures for the lifestyle intervention were discussed and approved by the DPP Steering Committee.

The DPP Lifestyle Resource Core at the University of Pittsburgh Medical Center consisted of the following individuals: Rena Wing, PhD; Beth Venditti, PhD; and Bonnie Gillis, MS, RD.

Copyright © 2017 by the University of Pittsburgh

The DPP Lifestyle Advisory Group consisted of the following individuals: Jim Hill, PhD; Mary Hoskin, MS, RD; Andrea Kriska, PhD; Wylie McNabb, PhD; Xavier Pi-Sunyer, MD; Michael Pratt, PhD, Judith Wylie-Rosett, PhD, RD and Linda M. Delahanty MS, RD and Steven E. Kahn M.B., Ch.B..

Special Consultants
In addition, valuable contributions to the editorial and graphic content of the DPP lifestyle intervention were made by the following programs: The Women's Health Initiative; The TONE Weight Reduction Program to Control High Blood Pressure; United Weight Control, in affiliation with St. Luke's Roosevelt Hospital Center; The Black American Lifestyle Intervention (BALI); and The Eat Well, Live Well Nutrition Program Workbook: Women Helping Women Make Healthy Choices.

Diabetes Prevention Support Center Faculty (established 2006)
At the heart of the lifestyle intervention framework provided in this manual is the Diabetes Prevention Support Center (DPSC) administrative core. The DPSC mission is to provide training and on-going support for those individuals and organizations who implement lifestyle treatment programs for the prevention and delay of diabetes and other co-morbid conditions. The University of Pittsburgh team that forms the core of the DPSC has been at the forefront of diabetes prevention. The DPP lifestyle intervention was developed in Pittsburgh, under the direction of Dr. Rena Wing, by Bonnie Gillis, MS, RD, Dr. Beth Venditti, and Dr. Andrea Kriska. Original adaptation of the DPP curriculum in 2008 was conducted by Bonnie Gillis, MS, RD with the DPSC Faculty (Dr. Trevor Orchard, Dr. M. Kaye Kramer, Dr. Venditti and Dr. Kriska).

The following individuals contributed to the ongoing and current adaptation of the DPP curriculum to the DPP Group Lifestyle Balance™:

M. Kaye Kramer, DrPH, MPH, RN
Andrea Kriska, PhD
Linda Semler, MS, RD, LDN
Elizabeth Venditti, PhD

Additional acknowledgement is made to Rebecca Meehan, MS, RD, LDN, Marni Armstrong, PhD, and Bonny Rockette-Wager for their contributions to the 2017 adaptation.

Copyright © 2017 by the University of Pittsburgh

Group Lifestyle Balance™:
Diabetes Prevention Program Lifestyle Change Program
Manual of Operations
Table of Contents

Acknowledgments i
Table of Contents iii
Revision History v
Overview xi
How to Use This Manual xii
Revision Policy xii
Group Lifestyle Balance Coach Training xii

Section 1: General Guidelines for Conducting the Group Lifestyle Balance™ Program

1.1 Overview 1-1
1.2 Materials and Supplies 1-2
1.3 Session Structure 1-3
1.4 Reviewing Tracking Records 1-4
1.5 Optional Supervised Group Activity 1-4
1.6 Documentation 1-6
1.7 Using Other Resources 1-6

Section 2: The Group Lifestyle Balance™ Goals and an Overview of Strategies to Achieve Them

2.1. Weight Goal 2-1
2.1.1. Rationale for the Weight Goal 2-1
2.1.2. Self-Monitoring Weight 2-2
2.1.3. Fat Intake Goal 2-3
2.1.4. Setting a Calorie Goal 2-3
2.1.5. Self-Monitoring Calories and Fat 2-4

2.2. Physical Activity Goal 2-5
2.2.1. Rationale for the Physical Activity Goal 2-5
2.2.2. Self-Monitoring of Physical Activity 2-6
2.2.3. Definition and Examples of Moderate Physical Activities 2-6

Copyright © 2017 by the University of Pittsburgh

Section 3: Key Principles Underlying the DPP Group Lifestyle Balance™ Program

3.1. Goals 3-1
3.2. Participant Self-Management 3-1
3.3. Tailoring the Program 3-2

Section 4: Participant Handouts and Leader Guides 4-1

Session 1: Welcome to the Diabetes Prevention Program Group Lifestyle Balance
Session 2: Be a Calorie Detective
Session 3: Healthy Eating
Session 4: Move Those Muscles
Session 5: Tip the Calorie Balance
Session 6: Take Charge of What's Around You
Session 7: Problem Solving
Session 8: Step Up Your Physical Activity Plan
Session 9: Manage Slips and Self-defeating Thoughts
Session 10: Four Keys to Healthy Eating Out
Session 11: Make Social Cues Work *for* You
Session 12: Ways to Stay Motivated
Session 13: Strengthen Your Physical Activity Plan
Session 14: Take Charge of Your Lifestyle
Session 15: Mindful Eating, Mindful Movement
Session 16: Manage Your Stress
Session 17: Sit Less for Your Health
Session 18: More Volume, Fewer Calories
Session 19: Stay Active
Session 20: Balance Your Thoughts
Session 21: Heart Health
Session 22: Look Back and Look Forward

Copyright © 2017 by the University of Pittsburgh

Revision History

Date	Revision
8/30/2004	Materials distributed at Lifestyle Balance Training Workshop, Pittsburgh, PA
1/30/2008	Scripts and participant handouts updated based on preventionist/lifestyle coach feedback.
11/12/2008	General updates to materials.<br>Reorganization of Sessions 4 and 10.
1/7/2010	Participant Handouts:<br>Session 1:<br>• Addition of pre-diabetes definition<br>• Addition of DPP Outcomes Study results<br><br>Session 2: Minor grammatical corrections<br>Session 3:<br>• Addition of American Heart Association recommendations for Omega 3 fatty acids and caution to clear all supplements with health care provider before taking.<br>• Updated to better reflect MyPyramid guidelines<br>Session 4: Addition of photos for stretching demonstration<br>Session 5: Minor grammatical corrections<br>Session 8: Updated terms and Fast Food Nutritional values to reflect current menus<br><br>Miscellaneous Handouts:<br>GLB Eating Plans: Updated terms and nutrition information<br><br>Leader's Scripts:<br>Session 1:<br>• Addition of pre-diabetes definition<br>• Addition of DPP Outcomes Study results<br><br>Session 3: Addition of American Heart Association recommendations for Omega 3 fatty acids and caution to clear all supplements with health care provider before taking
1/25/2011	Addition of 10 post-core sessions

Copyright © 2017 by the University of Pittsburgh

Participant Handouts:

Session 1:
- Inclusion of Be a Good Group Member in handouts
- Updated names of measuring tools

Session 2:
- Minor grammatical changes
- Updated names of measuring tools
- Updated calorie and fat gram information for food item examples used throughout the handout
- Included an example of a fast food meal from Burger King

Session 3:
- Addition of the Plate model as an option for meal planning
- Reduced sections related to MyPyramid
- Simplified "Rate Your Plate" in-class assignment and assignment for using and evaluating MyPyramid and the Plate Model

Session 4
- Minor grammatical changes
- Addition of "unstructured" activity to spontaneous activity as activity time that is not recorded
- Modification of "When to Stop Exercising" handout
- Clarification of physical activity goal for those doing more than 60 minutes of activity when program starts
- Introduction of the idea that it's not only important to increase physical activity, but also to decrease inactive or sedentary time

Session 5:
- Updated names of measuring tools
- Added reference to structured meal plans

Session 8: Updated terms and Fast Food Nutritional values to reflect current menus

Session 9: Expanded the definition of "negative thoughts" to include, more broadly, any self-defeating thoughts that get in the way of healthy change (e.g. excuses and rationalizations)

Session 10:
- Minor grammatical changes
- Addition of "step credit" calculation for activity not recorded by pedometer

Session 12:
- Rephrased the direct statement "add variety to your activity and meal routines" to a suggestion to "consider whether you need to add variety to your activity and meal routines"

Copyright © 2017 by the University of Pittsburgh

- Updated to reflect focus on year long program

Miscellaneous Handouts:

- Moved “How to Be a Good Group Member” handout to Session 1
- Revised How am I Doing for Weight and Activity
- Added a How am I Doing Weight chart sample
- Updated Low-Calorie, Low-Fat Cookbook list

Leader’s Scripts:

Session 1: Updated names of measuring tools

Session 2:

- Added option of displaying the amount of fat in a food item using shortening on a small plate.
- Added information about using the fat and calorie counter and a group activity to complete the Practice Page
- Updated the names of measuring tools
- Addition of optional measuring activity using cereal
- Replaced activity of measuring high fat/high calorie foods in class with discussion of high fat/high calorie foods with a display of the fat content of several

Session 3:

- Addition of the Plate Model as an option for meal planning
- Reduced sections related to MyPyramid
- Included option of giving participants a 9-inch Styrofoam divided plate
- Added “Rate Your Plate” group activities
- Simplified the assignment for using and evaluating MyPyramid and the Plate Model

Session 4: Addition of "step credit" calculation for activity not recorded by pedometer

Session 5: Minor grammatical changes

Session 8

- Minor grammatical changes
- Addition of several suggested group activities
- Added conclusion

Session 12

- Rephrased the direct statement “add variety to your activity and meal routines” to a suggestion to “consider whether you need to add variety to your activity and meal routines”
- Updated to reflect focus on year long program

Copyright © 2017 by the University of Pittsburgh

July 25, 2011 Revision to include 2011 USDA MyPlate and changes in The Dietary Guidelines for Americans, 2010.

August 1, 2017
To provide clarity and consistency, the 2017 curriculum is referred to as the Diabetes Prevention Program: Group Lifestyle Balance (DPP-GLB). A summary of the historical background regarding modifications to the original DPP lifestyle intervention is below:

- **Main changes across sessions:**
  - Focused on dietary self-monitoring shifted to calories, more than fat, to reflect current health recommendations.
  - Referred to “Food and Activity records” rather than “Keeping Track books”, as many programs/participants use other preferred tracking methods, including digital tracking options.
  - Formatted the “To Do” (home assignment) pages for consistency across all sessions.
  - Added a “Resources” section at the end of each session for optional/additional participant handouts and reference information.
  - Added more language about the importance of the physical activity approach and self-awareness of physical movement throughout the GLB curriculum.
  - Changed the order of some sessions and modified session titles.

- **Session 1: Welcome to the Diabetes Prevention Program: Group Lifestyle Balance**
  - Moved information about finding a personal weight goal to Session 9.
  - Moved information about minimum weight goal to Session 9.
  - Weight charts go up to 450 pounds. • Added information about tracking options: Food and Activity records/digital options.
  - Followed recent ACSM guidelines, encouraging “talk with your health care provider about becoming physically active”.
  - Revised “Weekly Record”. Space to record goals for calories, fat, and 7% weight loss.
  - Modified “How Am I Doing” template so participants may track weight for one year.

- **Session 2: Be a Calorie Detective:**
  - Focused on calories more than fat.
  - Deleted “Menu Make-Over” from original.
  - Focused on eating fewer calories, less fat, and less *unhealthy* fat.
  - Discussed the use of healthier fats (comparison examples have been modified).
  - Added information on the rationale for the calorie and fat gram goals, specifically: o Acknowledged that there are individual differences in achieving weight loss

Copyright © 2017 by the University of Pittsburgh

goals, but the goals provide a good evidence-based starting point.
  - Emphasized that one can expect 1-2 pounds of weight loss/week when achieving calorie and fat goals as prescribed.
  - Clarified that 25% of calories from fat is considered a *moderate* fat eating plan, not low-fat.
  - Included information on the importance of not going below 1200 calories/day.
  - Added "menus, menu boards, and displays" as a source of nutrition information to reflect pending federal regulations.
  - Changed sample Nutrition Facts label to reflect pending federal regulations.

- **Session 3: Healthy Eating**
  - Moved "Build a Better" (snack, meal) pages to Session 9.
  - Focused on staying close to calorie goal, more than fat gram goal.
  - Added information on importance of increasing physical activity levels for weight loss.
  - Added language to "replace", "limit", or "include" certain foods to improve health.
  - Added information about MyPlate based on 2016 revisions, included new graphic.
  - Added information about "healthy eating pattern" based on 2015 Dietary Guidelines.
  - Deleted section about Cholesterol due to changes in 2015 Dietary Guidelines.
  - Added "use a slow cooker" and directions for oven roasting vegetables.

- **Session 5: Tip the Calorie Balance**
  - Added more information about the role of physical activity.
  - Added information about liquid calories.
  - Added guidelines about caffeine as outlined in the 2015 Dietary Guidelines.
  - Added websites for additional meal plans per emphasis in 2015 Dietary Guidelines.
  - Modified "To Do" page language about the activity goal in Sessions 5-8.

- **Session 7: Problem Solving**
  - Added problem-solving activity ("Sam and Sadie") and worksheets.

- **Session 8: Step Up Your Physical Activity Plan**
  - Moved this session from 10 to 8. Rationale was to better balance the sequence of diet and activity focused sessions, and provide pedometer learning activities earlier.

- **Session 9: Manage Slips and Self-Defeating Thoughts**
  - Changed "negative thoughts" to "self-defeating thoughts".
  - Added personal weight goal and BMI chart to this session.
  - Clarified personal weight goal using a 5-pound weight range (rather than a 3-5 pound weight range).

Copyright © 2017 by the University of Pittsburgh

- Added Slippery Slope reflection/discussion activity.
- Added "Checking in with the Pedometer" section.
- Added "Build a Better" (snack, meal) pages to this session.
- Added step goal to the "To Do" in sessions 9-11.

- **Session 10: Four Keys to Healthy Eating Out**
  - Addressed nutrition information on menus, menu boards, and displays to reflect new federal regulations.
  - Added small group activity and practice sheets.

- **Session 12: Ways to Stay Motivated**
  - Revised "What's Your Purpose Now".
  - Increased emphasis on physical activity
  - Added structured menu of tracking options: gold, silver, bronze.

- **Session 13: Strengthen Your Physical Activity Plan**
  - Added a new Resistance Training poster and self-monitoring log.
  - Added new "Basics" for the "To Do" pages, Sessions 13-21.

- **Session 14: Take Charge of Your Lifestyle**
  - Added weight loss plateau information.
  - Added "Resistance Training Review" section.
  - Added "When You Reach Your Goal Weight" handout.

- **Session 15: Mindful Eating; Mindful Movement**
  - Added Mindful Movement.

- **Session 16: Mange Your Stress**
  - Added 7-Day Sitting Diary.

- **Session 17: Sit Less for Your Health**
  - New session to reflect increasing focus on the importance of reducing sedentary behaviors throughout the day.

Copyright © 2017 by the University of Pittsburgh

Overview

This manual provides detailed information and instructions for implementing the Diabetes Prevention Program Group Lifestyle Balance™ (GLB). The intent of this manual is to instruct and support health professionals in various settings in the delivery of a consistent and high-quality lifestyle change program.

The Diabetes Prevention Program Group Lifestyle Balance™ (GLB) is a comprehensive lifestyle behavior change program adapted directly from the successful lifestyle intervention used in the National Institutes of Health funded Diabetes Prevention Program (see acknowledgements for a description of the DPP). The original, individually administered, DPP Lifestyle Balance intervention (copyright 1996; 2011) was developed and written at the University of Pittsburgh by the DPP Lifestyle Resource Core on behalf of the DPP Research Group. Members from the original DPP lifestyle team collaborated to adapt and update the individual intervention to a group–based program with a recommended delivery schedule of 22 sessions during a 1-year period of time.

This program differs from that used in the DPP in several ways:

- In the DPP, the core curriculum sessions were conducted by a Lifestyle Coach with an individual participant. Individual sessions were necessary because of the staged recruitment for the study, which limited the formation of groups. After the core curriculum, individual sessions were held less frequently and were supplemented by group sessions. This program focuses primarily on **group sessions**, which allows the program to be offered more economically and provides group support throughout the program.

- The instructional content of the DPP 16-session curriculum was fully consolidated into 12-sessions and designed to be administered weekly. Subsequent to the 12 weekly sessions, a series of 4 transition sessions have been provided with a recommendation that the frequency of weekly group meetings be faded from weekly, to bi-weekly, and finally to monthly group contacts. This can be done with some scheduling flexibility during months 4-6 of the program. Six monthly sessions, which may be used interchangeably, have also been included to provide ongoing support throughout the remainder of the year.

- Other modifications to core content include a broad behavioral focus on the principles for making healthy food choices and meal planning rather than a central emphasis on the USDA food pyramid, instructions to combine both calorie and fat self-awareness and monitoring from the beginning of the intervention and inclusion of the pedometer as a valuable tool to help increase self-awareness and motivation for increasing overall movement and physical activity levels. As in the DPP, the goals of the GLB intervention

Copyright © 2017 by the University of Pittsburgh

are to achieve and maintain a 7% weight loss, and to safely and progressively increase to 150 minutes per week of moderately intense physical activity similar to a brisk walk.

- During the 4 transition sessions, group members are encouraged to practice their behavior change skills more independently as the level of group and facilitator support is reduced. The transition sessions reinforce core session learning as well as introduce the cognitive and behavioral strategies critical for long-term weight management. Other instructional content in this phase relates to the importance of including high-fiber, low-caloric density (e.g. plant-based) foods in meal planning, managing self-defeating thinking patterns, and incorporating strength-training (e.g. resistance band) exercises for a complete physical activity regimen in line with national standards.

- The final six (monthly) support sessions are designed to be delivered interchangeably depending on group and facilitator preference. These support sessions amplify core diabetes prevention and cardiovascular health messages and allow for continued weight, diet, and activity monitoring and accountability. In the monthly support phase, the majority of session time is utilized for either skills practice (e.g. flexibility and balance exercises, eating slowly and mindfully, how to spend less time sitting) or group problem solving (addressing barriers and challenges and making action plans for the future).

How to Use This Manual

This manual covers the initial 12 sessions and is comprised of two sections: 1) Lifestyle coach guidelines for conducting each session, and 2) Participant Handouts.

The purpose of this manual is to provide:
- Detailed information on the lifestyle intervention goals,
- A description of the key principles underlying the intervention,
- Strategies for responding to adherence problems, and
- Supplementary materials for participants and Lifestyle Coaches.

Revision Policy

Suggested revisions to this manual will be reviewed and approved by the Diabetes Prevention Support Center.

Group Lifestyle Balance Coach Training

One of the keys to the success of the DPP lifestyle intervention was the training and support that the Lifestyle Coaches received. In translating the DPP lifestyle intervention to "real world" settings, it is highly recommended that individuals who are planning to deliver the Group Lifestyle

Copyright © 2017 by the University of Pittsburgh

Balance program complete the two-day training workshop offered by the Diabetes Prevention Support Center of the University of Pittsburgh. In 2017, Innovative Wellness Solutions™ (IWS) licensed the training and continues to work with the DPSC to provide the training to the community. The training workshop provides knowledge and skills to enhance the expertise of the Lifestyle Coach. In addition, IWS and the DPSC faculty are available after training to provide support for implementation via telephone, e-mail and the DPSC website. The DPSC website hosts a support area to which only those who have completed the training workshop have access. While recommended, training is not required to utilize the GLB program materials.

Information regarding GLB training may be obtained by contacting:

Phone: 888-330-6891
E-mail: DPPInfo@iwellnessnow.com

Copyright © 2017 by the University of Pittsburgh

Section 1:
General Guidelines for Conducting the Group Lifestyle Balance™ Program

1.1 Group Overview

- The Group Lifestyle Balance Program should be offered in **groups of about 12-20 participants;** physician permission for physical activity is highly recommended for participation.

- Trained Lifestyle Coaches, will lead the groups. Lifestyle Coaches are providers who have training and experience in health care, particularly in nutrition and/or diabetes care (such as a nurse, dietitian, health educator, exercise specialist, social worker, diabetes educator, behaviorist, physician, etc.). It is highly recommended that those wishing to provide the Group Lifestyle Balance program complete the Group Lifestyle Balance 2-day training workshop.

- When possible, it is recommended that **groups are offered at different times during the week** (e.g., during the day, evening, and on the weekend). If possible, a survey of participating individuals beforehand to determine interest and which days and times would be most convenient for them to attend is encouraged.

- The GLB sessions should be held in a **private area**. A scale (balance beam or digital electric) must be available so that the participant can be weighed **privately** at each session.

- Participants should be assigned to a specific group and expected to attend the sessions with that group. Sites should determine how they will distribute session handouts when a participant misses (e.g. e-mail, USPS mail, or site determined make-ups). The GLB-DVD may also be utilized for make-up sessions where available.

- The goal is to offer the initial 12 GLB sessions over a period of 12-14 weeks. Subsequent to the 12 weekly sessions, the series of 4 transition sessions should be completed. It is recommended that the frequency of weekly group meetings fade from weekly, to bi-weekly, and finally to monthly group contacts. This can be done with some scheduling flexibility during months 4-6 of the program. Six monthly sessions, which may be used interchangeably, have been included to provide ongoing support and accountability throughout the remainder of the year.

Copyright © 2017 by the University of Pittsburgh

A guideline for the recommended sequence for delivery of the year-long GLB program is provided below:

Schedule	Group Lifestyle Balance™ Curriculum
**Month 1**	1. Welcome to the Diabetes Prevention Program: Group Lifestyle Balance™
	2: Be a Calorie Detective
	3: Healthy Eating
	4: Move Those Muscles
**Month 2**	5: Tip the Calorie Balance
	6: Take Charge of What's Around You
	7: Problem Solving
	8: Step Up Your Physical Activity Plan
**Month 3**	9: Manage Slips and Self-Defeating Thoughts
	10: Four Keys to Healthy Eating Out
	11: Make Social Cues Work for You
	12: Ways to Stay Motivated
**Month 4**	13. Strengthen Your Physical Activity Plan
	14: Take Charge of Your Lifestyle
**Month 5**	15: Mindful Eating, Mindful Movement
**Month 6**	16: Manage Your Stress
**Month 7**	17: Sit Less for Your Health
**Month 8**	18: More Volume, Fewer Calories
**Month 9**	19: Stay Active
**Month 10**	20: Balance Your Thoughts
**Month 11**	21: Heart Health
**Month 12**	22: Look Back and Look Forward

1.2 GLB Materials and Supplies

- **Each individual planning to provide the GLB program will need to have the following supplies on hand:**

    1. Manual of Operations (one per Lifestyle Coach)
    2. Set of participant handouts (one per participant)
    3. Keeping Track booklets (one per participant)
    4. Fat and Calorie Counters (one per participant)
    5. Pedometers (one per participant)

Copyright © 2017 by the University of Pittsburgh

6. Three-ring binders (one per participant)
7. Other supplies (such as food and food models) as indicated in the session scripts

- **Other items that participants should have on hand, or that should be provided for them:**
  1. A bathroom scale
  2. Tools for weighing and measuring foods (metal or plastic measuring cups and spoons, a glass measuring cup, a food scale that measures in ounces, a ruler).

  Lifestyle Coaches should be prepared with information on where to purchase these items most affordably as they will be cost-prohibitive for some participants. Each setting delivering the program will need to develop their own policies for handling this situation should it arise (e.g., provision of bathroom scales)

1.3 Session Structure

- **The basic content and sequence of the GLB sessions** should be consistent with the order that they are presented in this manual. This will ensure that all participants receive the same intervention program, that sessions on physical activity, healthy eating, and behavioral topics are interspersed, and that topics that build on those presented earlier come in the correct sequence.

- Each GLB participant should be given a three-ring binder **and at each session will receive a copy of the materials for that session.** Participants should generally *not* be given the entire set of materials at one time; however, this may vary depending on the individual setting. Participants should take the binder home with them at the end of each session and bring it to the next session. If a participant misses a session, the handouts for that session will be given to the participant at the next session attended. However, each site will determine the scope of methods used to deliver missed session materials (e.g., mailings, pick-up times and locations).

- The Lifestyle Coach should use the participant work sheets during the session to present the main points while the participants follow along. When possible, we recommend that Coaches display overheads or a Power Point of the participant handouts as the sessions are presented. A flip chart may also be used. However, it is important to keep in mind that the **emphasis is to be on group interaction and discussion.** Do *not* simply read the participant handouts to the group.

- **Complete all work sheets interactively with the group** (give individuals time to complete the work sheets on their own, then ask volunteers for sample responses and encourage group discussion). Whenever possible, introduce topics interactively by asking the group open-ended

Copyright © 2017 by the University of Pittsburgh

questions, sharing interesting anecdotes, and using various group activities. Be creative and enthusiastic.

- While maintaining a standard curriculum in terms of the basic content and sequence of the sessions, the Lifestyle Coach may tailor the presentation of the sessions to the overall group. The Coach should feel free to use supplementary educational aides if it is clear that this approach will enhance learning in the group and not draw attention or time away from the basic concepts presented. A guiding principle is to provide just enough material to be discussed during the hourly session (and minimal additional handouts to be reviewed at home) so participants are not inundated by information.

  Some examples of appropriate ways to tailor a session: Displaying test tubes filled with shortening to varying levels to illustrate the fat content of different foods, providing individual samples of low-fat food products to taste, providing local information relevant to the session topic

  Some examples of inappropriate ways to tailor a session: Having a hypnotist come to the session on motivation, dropping the session on slips because the group has not had any lapses, or recommending specific popular diet books.

1.4 Reviewing Tracking Records

- **Coaches should review the self-monitoring records periodically, making written comments on them and returning them at the next session to the participants.** The comments should highlight examples of positive changes the participant has made and help the participant solve any problems encountered, particularly those related to the topics discussed at the previous session. Because the self-monitoring records are intended to help the participant make behavior changes rather than serve as a source of dietary data, the review should not be as detailed or extensive as would be the case when documenting food records to be entered for nutrient analysis.

- **More frequent review of self**-monitoring records is optional. The Coach may opt instead to take time at the beginning of the sessions to ask volunteers for examples from their self-monitoring records and use these examples to make the points highlighted in the scripts. Coaches are also to feel free during any of the sessions to ask for examples from participants' records to support the session content.

1.5 Optional Supervised Group Activity

- **Supervised activity sessions are optional.** In the DPP, every clinical center was required to provide supervised physical activity sessions at least two times per week, and the study kept

Copyright © 2017 by the University of Pittsburgh

logs of attendance and paid any fees. Many participants found the sessions helpful in achieving their activity goal and providing group support.

Settings that choose to provide supervised activity sessions should strongly recommend that all participants who can perform at least 30 minutes of continuous, moderate intensity activity attend the sessions. The goal is for all participants to have the opportunity to give the sessions a "good try," receive hands-on physical activity instruction and encouragement from the session leaders, and meet other participants with whom they can develop support networks for being active. Throughout the program, participants who are having difficulty meeting their exercise goal should in particular be encouraged to attend.

The supervised activity sessions should **last about 45 minutes to 1 hour and include a warm-up period, followed by about 30-40 minutes of exercise and a cool-down period.** Possible locations include malls, parks, gymnasiums, or exercise facilities such as the Y or private health club. The types of physical activity may vary and should be tailored to the skills and interests of the participants. It is recommended that at least one session per week involve brisk walking. Other possible types of activities include aerobic dance, resistance training, and step aerobics. The activities offered should be equivalent to brisk walking.

The supervised activity sessions should be scheduled **at times and locations to accommodate as many participants as possible.** Coaches may need to experiment with various types of activities, times and locations in order to attract more participants. To determine the types of activities to offer and the most convenient times and locations, Coaches may want to periodically survey participants by mail.

Keep in mind the following safety issues:

- If possible, during the activity sessions, considerations should be made for participants of different fitness levels. For example, the leader may need to split his or her time between the slow and fast walkers or consider walking on a track so that a variety of paces can be accommodated. Some settings may have adequate staff to provide more than one leader.

- If the activity session is being held at a remote location with limited access to emergency medical services or a telephone (such as on a hiking trail), the leader should have a cell phone for emergencies and if possible, a First Aid kit with bandages, ace wrap and cold pack for minor injuries.

- Leaders should emphasize hydration during the activity sessions (especially in warmer weather) and should have water available for participants who do not bring water.

Copyright © 2017 by the University of Pittsburgh

In general, it is anticipated that most of the supervised physical activity sessions will be **led by a member of the program staff**. All activity session leaders should be trained in CPR. **Another option is to use an exercise class or facility in the community** (in some cases, you may be able to negotiate a discount on any fees for program participants). If you use an outside class or facility:

1. Before participants attend the class, **meet with the leader** to evaluate the facility and the nature of the class and explain the purpose of the program and the activity goal. For as long as participants attend, contact the leader periodically to check on how the participants are doing. If the class leader changes, meet with the new leader to orient him or her to the program and the activity goal.
2. **Advertise the class to all participants who are at an appropriate fitness level.** In the DPP, a number of models were used for supervised activity that some practices may be able to use:

Neighborhood Group Walks
In the DPP, two or more group walks were offered per week in separate neighborhoods around the city, convenient to different participants. The Lifestyle Coach led the walks. When possible, the walks were tied into training for a community walking event such as the local American Diabetes Association walk.

Cardiac Rehabilitation Unit
Participants used a cardiac rehabilitation unit with a treadmill, exercise bike, recumbent bike, stair master, and free weights. The Coach introduced the Lifestyle Balance Program participants to the unit, and then participants set up a regular schedule with the center staff for at least 2-3 times per week.

Community Exercise Class or Facility
Participants attended aerobic dance classes or step aerobic classes at a local Wellness Center or the Y. The Coach introduced the participants to the class leaders.

1.6 Documentation

- **Documentation is tailored to the needs of each setting.** Depending on your setting, Lifestyle Coaches may be responsible for completing documentation. A sample Group Lifestyle Balance Leader's Log is included in the Appendix section of this manual and may be used if desired.

1.7 Using Other Resources

- Some participants may raise problems during the program that are outside the expertise of the Lifestyle Coach, such as a significant clinical depression, anxiety, or a clinical eating disorder.

Copyright © 2017 by the University of Pittsburgh

Each Lifestyle Coach should be prepared to make referrals to behavioral and mental health resources to address such problems.

- Each Lifestyle Coach should also be prepared to provide participants with **information about community resources** that can be used after the program to support healthy eating, physical activity, and weight loss/weight maintenance.

- Lifestyle Coaches may want to **contact local businesses and arrange for discounted services or products for participants who complete the program and/or reach their program goals** (for example, a discount on athletic shoes or equipment, gym membership, or fruits and vegetables).

Copyright © 2017 by the University of Pittsburgh

Section 2:
The Group Lifestyle Balance™ Goals
and an Overview of the Strategies to Achieve Them

As in the DPP, all Group Lifestyle Balance participants are to try to achieve two program goals:

- To achieve and maintain a weight loss of 7% of their initial body weight, and
- To achieve and maintain an energy expenditure of 700 kilocalories per week through moderate physical activity (equivalent to approximately 2 ½ hours (150 minutes) per week of brisk walking).

2.1. Weight Goal

The weight goal for the GLB program is to lose 7% of initial body weight (as measured at Session 1, the first GLB session, rounded to the nearest pound) and maintain that weight loss. The recommended pace of weight loss is 1 to 2 pounds per week, for a 7% loss within approximately 24 weeks. Individual weight goals are provided in Session 1 (see the participant handout entitled Goal Weights for the Group Lifestyle Balance Program).

2.1.1. Rationale for the Weight Goal

A 7% weight loss was used in the DPP because it was believed to be safe, effective, and feasible. Previous studies had shown that a 10% weight loss lowered glucose and improves cardiovascular risk factors, with an apparent dose-response relationship between magnitude of weight loss and improvement in these parameters. In addition, standard behavioral weight loss programs produce initial weight losses of approximately 10% of body weight. However, the DPP goal was not only to produce but also to maintain a weight loss for up to 6 years, and maintenance of weight loss has been shown to be difficult, with 10% weight loss at long-term follow-up rarely achieved in weight control programs or clinical trials. Therefore, the goal of a 7% weight loss was selected as more feasible for participants to maintain over the course of the DPP.

As in the DPP, GLB participants who wish to lose *more* than 7% of their starting weight may be encouraged to do so, although weight loss below the goal should be encouraged only if the participant continues to have a BMI of greater than 21 (see the Session 1 participant handout entitled Minimum Recommended Weight for Height) after achieving the weight goal. For example, a participant who weighs 130 pounds at Session 1 would be given a weight goal of 121 pounds (Table 2.1). If the participant reaches that goal and wants to continue losing weight, the Coach should refer to the Minimum Recommended Weight for Height handout. If the participant's height is 65 inches, the participant is already below a BMI of 21 (that is, below 126 pounds), so weight maintenance at 121 pounds should be encouraged rather than further weight

Copyright © 2017 by the University of Pittsburgh

loss. On the other hand, if the participant's height is 62 inches, the Coach would be able to encourage further weight loss to 115 pounds (a BMI of 21).

Sustained weight losses of more than 3 pounds per week are not to be advised because of safety issues.

Participants who wish to lose *less* than 7% of their starting weight should be encouraged to reach a 7% loss in a step-wise fashion, but the program goal should remain firm.

The weight goal is set at a level that should be challenging but reasonable. It is recognized that not all participants will achieve the goal at all times throughout the program. However, all participants, with the aid of their Lifestyle Coach, should endeavor to achieve and maintain the goal.

The goals are always based on weight loss from Session 1. For example, if a participant weighs 180 at session 1, his/her weight goal is 167 pounds; this remains the weight goal even if the participant at some time gains weight to 190 pounds.

2.1.2. Self-Monitoring Weight

As in the DPP, all participants will be weighed at every session. Participants should be weighed **in private** at the beginning of the session. Weight can be taken with either a balance beam or a digital scale. The type of scale is not important, but an effort should be made to use the same scale throughout the program. Sites should also adhere to the scale calibration procedures provided by the manufacturer or to other measurement standards in use at their facility. Participants should be weighed in light-weight, indoor clothes, without shoes.

Weights taken at the sessions should be recorded using the Weight Log provided with this manual and on the weight graph in the participant's notebook. Alternatively, Lifestyle Coaches may develop their own weight recording form for use at their site, or may use the log included with this manual. Participants should be encouraged to complete the weight graph themselves, if possible. The Lifestyle Coach (or other individual who weighs the participant at the start of each session) and participant should discuss the participant's weight in relation to the 7% weight loss goal, and encourage the participant to achieve the 7% weight loss goal over six months.

In addition to being weighed at every session, all participants will be encouraged to weigh themselves at home at least weekly and record their weight on the back of their self-monitoring booklets. Participants will be instructed to weigh themselves on the same day(s) of the week and at the same time of day (for example, on Monday mornings).

Copyright © 2017 by the University of Pittsburgh

At the beginning of the program, Coaches may want to assign more frequent self-monitoring of weight, for example, daily, and continue to encourage it if the participant finds it helpful. Some participants may respond to frequent fluctuations in their weight by becoming discouraged. Importantly, the coach should acknowledge that although some participants have had previous negative experiences with self weighing or being weighed by others that in this lifestyle intervention the goal is to "make peace with the scale" because it is an essential feedback mechanism in making behavior changes. In this manner, the Coach can use a participant's record of frequent ups and downs in weight to teach the participant to focus on **trends** rather than on single values and to respond promptly to slips with positive behavior changes until the results are seen consistently on the scales.

2.1.3. Fat Intake Goal

To help participants achieve and maintain the weight goal, all participants will be given a goal for daily calories and total fat. As in the DPP, the fat goals have been calculated based on 25% of total calories from fat, using a calorie level estimated to produce a weight loss of 1 to 2 pounds per week (described in detail below). The various fat gram levels were then collapsed into one of four goals: 33, 42, 50, or 55 grams of fat.

A level of 25% of calories from fat was selected because it is believed to be effective, safe, and feasible. In the Women's Health Trial, a low-fat dietary-intervention trial, more than 80% of the intervention group had met their fat gram goal, calculated as 20% of baseline calories, within 3 months of randomization and maintained that goal through the end of the trial at 3 years. Although women in this study were not encouraged to decrease energy intake or lose weight, the reduction in fat intake was associated with a 25% reduction in total calories and a weight loss of 3.1 kg after 1 year. Weight loss was more strongly associated with change in percent energy from fat than with change in total energy intake.

All participants are to be given a fat intake goal, but it should be recognized that not all participants will immediately achieve this goal. For example, a participant who eats 40% of their calories from fat may initially find it difficult to achieve the 25% goal and may first reduce to 35% fat and then to 30% fat. However, the participant should be assigned the 25% fat goal, and all progress toward reaching this goal should be praised.

2.1.4 Setting a Calorie Goal

In the DPP, all participants were given a fat goal at the start of the study. Then after four to eight weeks, a calorie goal was introduced for participants who had not lost weight as expected. In the Group Lifestyle Balance program, participants receive both a calorie and fat goal at the start of the program, to enhance the pace of weight loss.

Copyright © 2017 by the University of Pittsburgh

As in the DPP, the calorie goals have been calculated by first estimating the daily calories needed to maintain starting weight (starting weight multiplied by 12). Next, between 500 and 1000 calories were subtracted to estimate the calories needed to lose 1 to 2 pounds per week and achieve the weight loss goal within the first 24 weeks. More calories were subtracted for heavier participants with the rationale that they have more weight to lose to reach the 7% weight loss goal (500 calories were subtracted for starting weights less than 150 pounds, 750 calories for starting weights between 150 and 200 pounds, and 1000 calories for starting weights over 200 pounds.) Finally, the ranges of calories estimated for weight loss were collapsed into one of four standard calorie levels: 1200, 1500, 1800 or 2000.

Some participants may report a low fat/calorie intake without losing weight. In this case, the Lifestyle Coach should review the quality of the participants' self-monitoring and if lacking, (for example, if portion sizes are being inaccurately reported, if additions such as cream to coffee are routinely forgotten, etc.), the Coach should help the participants improve their self-monitoring skills. If after attempts to improve self-monitoring, a participant is still not losing weight, it may be necessary to lower the calorie goal further to help him or her achieve the weight loss goal.

As in the DPP, lowering dietary calories to a specific level is used in this program as a means to achieving the weight loss goal, rather than as a goal in and of itself. Thus, if a participant is consuming more than the assigned calorie goal, but is achieving the weight goal, there is no need to focus on greater reductions in calories.

2.1.5 Self-Monitoring Calories and Fat

All participants will be instructed to self-monitor fat intake in grams **daily throughout the program**. In the DPP, all participants were asked to record their intake daily for 24 weeks because of the extensive evidence that self-monitoring is highly correlated with success in reaching dietary change goals. For the Group Lifestyle Balance program, participants will also be instructed to self-monitor calories. Numerous studies have shown a dose-response relationship between frequency of self-monitoring and level of success in losing weight and/or improving cardiovascular risk factors. **Many experts consider self-monitoring the single most effective approach to changing dietary intake.** Participants in clinical trials and behavioral weight loss studies are typically asked to record their intake daily for the first several months of the intervention.

As in the DPP, participants should be given the following standard self-monitoring tools:

- A booklet (or online option) for recording seven days of food intake with fat and/or calorie values, as well as physical activity.
- A nutrient counter (or online option) alphabetized by food name, with the fat gram and calorie content of household portions and restaurant items.
- All participant handouts and a weight chart

Copyright © 2017 by the University of Pittsburgh

- A pedometer (or other physical activity tracking tool)

Other items that participants should have on hand or that should be provided for them:

- Tools for weighing and measuring foods (a food scale, metal or plastic measuring cups and spoons, a glass measuring cup, ruler).
- A bathroom scale

Participants will be encouraged to be complete and accurate in self-monitoring and at the same time to feel free to use abbreviations and short-cuts that work for them. In other words, the **participant is NOT taught to self-monitor with the thoroughness and detail that would be required if the records were to be entered into a computer for nutrient analyses.**

It is recognized that not all participants will self-monitor daily at all times throughout the program. However, all participants should endeavor to achieve and maintain daily self-monitoring and should receive a strong and clear message that self-monitoring is the key behavior change strategy in the program.

Lifestyle Coaches are to review the self-monitoring records periodically, making written comments on them and returning them at the next session to the participants. The comments should highlight examples of positive changes the participant has made and help the participant solve any problems encountered, particularly those related to the topics discussed at the previous session. Because the self-monitoring records are intended to help the participant make behavior changes rather than serve as a source of dietary data, the review should *not* be as detailed or extensive as would be the case when documenting food records to be entered for nutrient analysis.

2.2. Physical Activity Goal

The Group Lifestyle Balance activity goal is to reach and maintain a weekly activity level of 2½ hours (150 minutes) of moderate physical activity (such as brisk walking) per week. Physical activity is introduced in Session 4. If participants do not achieve the goal within five weeks of beginning activity, they should be encouraged to achieve it as soon as possible thereafter. Participants who enter the program already at the goal or better are encouraged to maintain that level.

2.2.1. Rationale for the Physical Activity Goal

A physical activity goal of 2½ hours (150 minutes) per week of moderate intensity activity was selected in the DPP because previous studies had shown that this level was beneficial

Copyright © 2017 by the University of Pittsburgh

(demonstrating improvements in weight, glucose, and insulin sensitivity) as well as being attainable.

As in the DPP, the GLB program's physical activity goal is a minimum. Participants who wish to be more active may be encouraged to do so. Participants who are already active when they enter the program will need to determine the amount of time they are currently spending in physical activity and then add further activity to reach the 2½-hour goal. For example, a participant who already does aerobic dance for 2 hours per week may continue this and add another ½ hour of aerobic dance or another type of moderate activity to reach the 2½-hour goal. In addition, participants who are active sporadically (e.g., seasonally) should be encouraged to achieve the goal consistently throughout every month of the program.

It is recognized that not all participants will achieve the activity goal at all times throughout the program. However, all participants, with the aid of their Coach, should endeavor to achieve and maintain the goal.

2.2.2. Self-Monitoring of Physical Activity

All participants will be instructed to **self-monitor physical activity daily throughout the program.** Self-monitoring records are to be reviewed periodically by the Lifestyle Coach and returned to the participant, with written or verbal comments from the Coach. The comments should highlight examples of positive changes the participant has made and help the participant address any barriers to physical activity encountered.

2.2.3. Definition and Examples of Moderate Physical Activities

As in the DPP, the intent of the GLB program is to encourage all types of physical activity. We believe that most participants will use walking as their primary type of physical activity. These individuals should be instructed to walk briskly for 2 ½ hours during the week. Other activities that are similar in intensity to brisk walking are listed in a participant handout in Session 4.

The Lifestyle Coach should discuss with the participants the types of physical activities they plan to do and evaluate each activity on a case-by-case basis in terms of its application toward the program goal. The key is to get the participants moving and gradually and steadily progressing toward the program goal.

The following general guidelines are provided to help Lifestyle Coaches judge whether an activity is equivalent to brisk walking:

- **The activity should last at least 10 minutes,** not including breaks (although some activities such as tennis or jumping rope may involve short "breaks" in the activity).

Copyright © 2017 by the University of Pittsburgh

- **For job-related activities, in addition to the above two criteria, the physical activity should comprise at least 50% of the job.**

For example:

**Equivalent to brisk walking**	**Not equivalent to brisk walking**
Using a gas-powered push mower (not a riding mower) to mow the lawn with some exertion.	Using a riding mower to mow the lawn.
Delivering the mail if 75% of the day is spent walking.	Delivering the mail if 75% of the day is spent driving a truck.
Going to a dance and dancing most of the evening.	Going to a dance and dancing only a few times during the evening. Spending most of the time socializing and watching others dance.

Some sports and leisure activities are clearly not equivalent in intensity or duration to brisk walking, such as archery, bowling and billiards. These are to be encouraged as part of an active lifestyle but are not to be applied toward the activity goal. Likewise, other activities, such as light yard work and light housework are to be encouraged as part of an active lifestyle but not self-monitored or applied toward the goal because they usually do not represent a level of activity equivalent to brisk walking. The criteria of "equivalent to brisk walking" is used with the rationale that such activities will be most likely to help participants lose weight, lower glucose, and improve cardiovascular risk factors.

Copyright © 2017 by the University of Pittsburgh

Section 3:
Key Principles Underlying the Group Lifestyle Balance™ Program

3.1 Program Goals

The program is based on clearly defined goals.

All participants receive a goal for weight loss and physical activity. From the beginning of the intervention, Lifestyle Coaches should state these goals without equivocation and set high expectations for participants in terms of achieving and maintaining them for the length of the trial. The rationale is that reaching and maintaining the goals is what will reduce the risk of diabetes onset.

3.2 Participant Self-Management

The program is based on participant self-management.

Although firm goals are provided, each participant makes personal choices about how to achieve the goals. This allows flexibility and reinforces the ability of the participants to shape and evaluate their own progress by self-monitoring, developing personal goals and action plans, and problem solving. The role of the Coach is to guide and support the participants in the process of self-management.

To achieve a balance between firm goals and participant self-management, Lifestyle Coaches will need to draw on all of their professional skills and experience. Central to the success of the intervention is the relationship between Coach and participant. Ideally, this relationship might be compared to that between a talented coach and a prized member of an athletic team. As "lifestyle coaches," we recommend that group leaders practice the following.

- **Express support and acceptance** for participants regardless of their progress toward program goals.
- **Look for success and build on it**, no matter how small or gradual.
- At the same time, **maintain the highest of standards and expectations.** A Lifestyle Coach should not lessen the program goals to match what seems "realistic" or "do-able"

Copyright © 2017 by the University of Pittsburgh

for a participant, any more than a health care provider would ask a patient to aim for less than optimal glucose monitoring and regulation. Instead, the Coach should express ongoing confidence that the participant will be able to reach and maintain the goals and then provide the utmost support in helping the participant address any barriers to that end. As we all know, expectations are often self-fulfilling. If expected to do poorly, participants are more likely to do poorly; if expected to do well, many participants will rise to the occasion.

- Along the same lines, **do not assume that a barrier to the goals exists until it is evident** (for example, that a participant who has a lower level of education will be unable to calculate fat grams when self-monitoring). Such assumptions are often based on hidden biases that may prove false (for example, some interventionists have reported that it is the less educated participants who do the most thorough self-monitoring).

- **When barriers do become evident, involve the participant as much as possible in addressing them, through goal setting and problem solving.** Use and convey an experimental approach--the evidence of a barrier is not a sign of failure on the part of the Coach or the participant but rather is a valuable piece of information to be used to design and test a better experiment, together.

- **Be the expert.** Be confident and firm when assigning the strategies for change presented in the intervention (such as self-monitoring of fat gram intake and physical activity). Stress that previous research has shown these strategies to be highly successful for many, many people. However, be flexible about using other strategies as needed. Information and behavioral strategies have been included in the intervention because of their likelihood of enhancing achievement and maintenance of the goals, not as ends in themselves.

3.3 Tailoring the Program

The program is to be tailored to participant lifestyle, learning style, and culture.

The Group Lifestyle Balance program should be tailored to each participant’s lifestyle, learning style, and culture. Many, many factors (such as ethnic heritage, socioeconomic status, marital status, and roles at work and at home) will have an impact on the eating and activity behaviors of participants. Such factors will also be at work in the lives of the Lifestyle Coaches themselves and will influence the way they interact with participants.

Copyright © 2017 by the University of Pittsburgh

Lifestyle Coaches should therefore remain open and sensitive to whatever factors may be important to each individual participant and at the same time, avoid stereotyping or making assumptions. The goal is to implement the GLB program with awareness, consideration, and careful communication so that differences can be used to enhance the intervention rather than get in its way.

Some points to keep in mind regardless of a participant's lifestyle or cultural heritage:

- Be careful to avoid interpreting a behavior within your own cultural context without asking.
- Low-literacy English is not a sign of intelligence or a predictor of success with the program.

Copyright © 2017 by the University of Pittsburgh

Section 4:
Participant Handouts and Leader Guides

Participant handouts are available for each of the sessions. Leader guides are included for each session for the group leader. The leaders of the sessions should follow the scripts while tailoring how the topics are presented (such as the language and examples used) to the participant's learning style and group process.

Keep in mind that the emphasis in these group sessions should be on *group interaction and discussion*. Do not simply read the handouts to the group.

Copyright © 2017 by the University of Pittsburgh

Participant Notebook

Welcome to the Diabetes Prevention Program:
Group Lifestyle Balance™

Your Name:

____

Your Lifestyle Coach:

____

How to Contact Your Lifestyle Coach:

____

____

____

Copyright 2017 University of Pittsburgh

Welcome to the Diabetes Prevention Program: Group Lifestyle Balance™

© 2004; 2008; 2011; 2017 by the University of Pittsburgh.*

Developed by the Diabetes Prevention Support Center faculty of the University of Pittsburgh (Kaye Kramer, RN, MPH, DrPH, CCRC; Andrea Kriska, PhD; Trevor Orchard, MD; Linda Semler, MS, RD, LDN; and Elizabeth Venditti, PhD), and Bonnie Gillis, MS, RD, LDN, with funding support from National Institutes of Diabetes and Digestive and Kidney Diseases, National Institutes of Health and United States Air Force administered by the U.S. Army Medical Research Acquisition Activity, Fort Detrick, Maryland, Award Number W81XWH-04-2-0030. Review of materials does not imply Department of the Air Force endorsement of factual accuracy or opinion.

*The GROUP LIFESTYLE BALANCE™ (GLB) manual and materials are made available to the public subject to the following Creative Commons License: Creative Commons – Attribution – ShareAlike 4.0 International. Accordingly, the manual and materials may be downloaded, duplicated, transmitted and otherwise distributed for educational or research purposes, as well as commercially, provided proper credits are given to the University of Pittsburgh Diabetes Prevention Support Center and the Diabetes Prevention Program research team. In addition, users must provide a link to the license and also indicate if any changes were made to the materials. If you remix, transform, or build upon the materials, you must distribute your contributions under the same license as the original.

Session 1: Welcome to the Diabetes Prevention Program: Group Lifestyle Balance™

Be a Good Group Member

In this program, you will be part of a group. A group is like a team. By working together, group members can:

- Support and encourage each other.
- Share ideas to solve problems.
- Motivate one another.

Being a good group member is a responsibility to others. It is also the best way to help YOU be successful. Team spirit helps *everyone* on the team.

10 Ways to Be a Good Group Member

- Do your very best to come to every meeting. This is important even when it's hard to follow the program.
- Be on time. Contact your Lifestyle Coach if you can't come.
- Complete the things you're supposed to do at home.
- Bring your notebook and Food and Activity record to every meeting.
- Take part in sharing your ideas with other group members.
- Let everyone have a chance to share. Be careful how much time you spend talking.
- Be willing to listen to the concerns of others. Do what you can to show you understand and care. Share what has worked for you.
- Do not repeat to other people outside of the group anything personal that is talked about in the group.
- Respect other people's ideas. Avoid putting others down.
- Stress the good things.

Copyright 2017 University of Pittsburgh

Remember Your Purpose

Let's reflect on and discuss these questions:

- Why did I join Group Lifestyle Balance™?

  ____

  ____

  ____

- What do I hope to achieve by taking part in Group Lifestyle Balance™?

  ____

  ____

  ____

- How will healthy eating and being active help me and/or others?

  ____

  ____

  ____

Copyright 2017 University of Pittsburgh

The Diabetes Prevention Program

Let's start with some background.

This program is based on a landmark study, funded by the National Institutes of Health. It was called the Diabetes Prevention Program (or DPP).

The DPP involved more than 3,000 adults with pre-diabetes. In pre-diabetes, a person's blood glucose (this is sometimes referred to as blood sugar) levels are above normal. However, these levels aren't high enough to have a diagnosis of diabetes.

- About half of participants in the DPP were African Americans, Hispanics, American Indians, Asians, or Pacific Islanders. Two out of 10 were 60 years of age or older. Diabetes is very common in these groups.
- Each person was assigned by chance to one of three groups:
  - One group took part in a "lifestyle program" to lose weight and be more active.
  - One group took metformin, a pill used to treat diabetes.
  - One group took a placebo (sugar pill).

The DPP was a great success.

1. Compared to the placebo group, taking metformin cut the chances of getting type 2 diabetes by 31%.
2. The lifestyle program to lose weight and be more active was even more successful.

- Losing 10 to 15 pounds and being moderately active (similar to a brisk walk) for at least 150 minutes per week cut the chances of getting type 2 diabetes by 58%. More than half.
- For those over the age of 60, the lifestyle program cut the chances of getting diabetes by 71%. Close to three-quarters.

3. The DPP Outcomes Study (DPPOS) continues to show positive benefits of modest weight loss and physical activity.

The bottom line is that losing weight and being more active lower your chances of getting type 2 diabetes.

Now there are programs like the DPP all over the world.

Copyright 2017 University of Pittsburgh

The DPP and the Metabolic Syndrome

The DPP lifestyle program also reduced the chances of the metabolic syndrome by 41%.

Someone with the metabolic syndrome has three or more of the following:

- More fat stored in the belly than on the hips (being "apple shaped")
- High triglycerides (a kind of fat in the blood)
- Low HDL cholesterol ("good cholesterol")
- High blood pressure
- High fasting blood glucose

One of every four American adults has the metabolic syndrome. Metabolic syndrome increases your chances of:

- Heart attack
- Diabetes
- Stroke
- Other problems related to these conditions such as eye, nerve, and kidney problems

Group Lifestyle Balance™ (or GLB) is a direct adaptation of the successful DPP lifestyle program. GLB is designed to help you make lifestyle changes to prevent diabetes and also prevent or treat the metabolic syndrome.

Copyright 2017 University of Pittsburgh

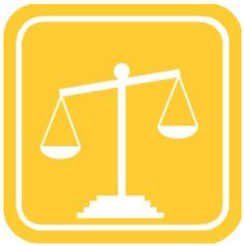

Group Lifestyle Balance™ Goals

We call this program "Lifestyle Balance" because, just like the DPP, we will help you reach a healthy balance between two parts of your lifestyle:

- What you eat and
- How physically active you are.

Your goals will be the same as the DPP lifestyle goals. Many studies, including the DPP, have shown that these goals are safe, can be reached, and kept up over time.

Goal 1. Lose 7% of your weight.

You will lose weight at a pace that is safe and do-able: about 1-2 pounds per week.

To get started: Look at your Weekly Record to check how much you weighed today. Find that weight in the first column of the boxes on the next pages.

Circle it. Then circle the other numbers in that row. Copy them below.

Starting weight (Session 1: today): _____________

Pounds to lose: _____________

Goal weight (7% loss): _____________

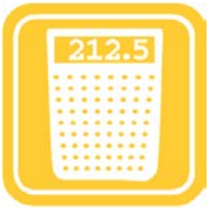

Also write the numbers at the top of your Weekly Record**.**

Goal 2. Do at least 150 minutes of physical activity each week.

Your physical activity program will be one that you CAN achieve. We will help you reach your activity goal at a pace that is safe and do-able.

In Session 4, we will help you get started on reaching your physical activity goal.

For now, pay close attention to how active you are now and where you may be able to add activity to your day.

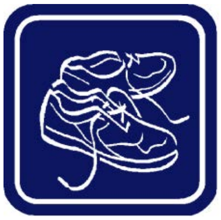

Copyright 2017 University of Pittsburgh

Group Lifestyle Balance™
Goal Weights

Starting Weight	Pounds to Lose	Goal Weight (7% Loss)
120	8	112
121	8	113
122	9	113
123	9	114
124	9	115
125	9	116
126	9	117
127	9	118
128	9	119
129	9	120
130	9	121
131	9	122
132	9	123
133	9	124
134	9	125
135	9	126
136	10	126
137	10	127
138	10	128
139	10	129
140	10	130
141	10	131
142	10	132
143	10	133
144	10	134
145	10	135
146	10	136
147	10	137
148	10	138
149	10	139
150	11	139
151	11	140
152	11	141

Starting Weight	Pounds to Lose	Goal Weight (7% Loss)
153	11	142
154	11	143
155	11	144
156	11	145
157	11	146
158	11	147
159	11	148
160	11	149
161	11	150
162	11	151
163	11	152
164	11	153
165	12	153
166	12	154
167	12	155
168	12	156
169	12	157
170	12	158
171	12	159
172	12	160
173	12	161
174	12	162
175	12	163
176	12	164
177	12	165
178	12	166
179	13	166
180	13	167
181	13	168
182	13	169
183	13	170
184	13	171

Copyright 2017 University of Pittsburgh

Starting Weight	Pounds to Lose	Goal Weight (7% Loss)
185	13	172
186	13	173
187	13	174
188	13	175
189	13	176
190	13	177
191	13	178
192	13	179
193	14	179
194	14	180
195	14	181
196	14	182
197	14	183
198	14	184
199	14	185
200	14	186
201	14	187
202	14	188
203	14	189
204	14	190
205	14	191
206	14	192
207	14	193
208	15	193
209	15	194
210	15	195
211	15	196
212	15	197
213	15	198
214	15	199
215	15	200
216	15	201
217	15	202
218	15	203
219	15	204
220	15	205
221	15	206
222	16	206
223	16	207

Starting Weight	Pounds to Lose	Goal Weight (7% Loss)
224	16	208
225	16	209
226	16	210
227	16	211
228	16	212
229	16	213
230	16	214
231	16	215
232	16	216
233	16	217
234	16	218
235	16	219
236	17	219
237	17	220
238	17	221
239	17	222
240	17	223
241	17	224
242	17	225
243	17	226
244	17	227
245	17	228
246	17	229
247	17	230
248	17	231
249	17	232
250	18	232
251	18	233
252	18	234
253	18	235
254	18	236
255	18	237
256	18	238
257	18	239
258	18	240
259	18	241
260	18	242
261	18	243
262	18	244

Copyright 2017 University of Pittsburgh

Starting Weight	Pounds to Lose	Goal Weight (7% Loss)
263	18	245
264	18	246
265	19	246
266	19	247
267	19	248
268	19	249
269	19	250
270	19	251
271	19	252
272	19	253
273	19	254
274	19	255
275	19	256
276	19	257
277	19	258
278	19	259
279	20	259
280	20	260
281	20	261
282	20	262
283	20	263
284	20	264
285	20	265
286	20	266
287	20	267
288	20	268
289	20	269
290	20	270
291	20	271
292	20	272
293	21	272
294	21	273
295	21	274
296	21	275
297	21	276
298	21	277
299	21	278
300	21	279
301	21	280

Starting Weight	Pounds to Lose	Goal Weight (7% Loss)
302	21	281
303	21	282
304	21	283
305	21	284
306	21	285
307	21	286
308	22	286
309	22	287
310	22	288
311	22	289
312	22	290
313	22	291
314	22	292
315	22	293
316	22	294
317	22	295
318	22	296
319	22	297
320	22	298
321	22	299
322	23	299
323	23	300
324	23	301
325	23	302
326	23	303
327	23	304
328	23	305
329	23	306
330	23	307
331	23	308
332	23	309
333	23	310
334	23	311
335	23	312
336	24	312
337	24	313
338	24	314
339	24	315
340	24	316

Copyright 2017 University of Pittsburgh

Starting Weight	Pounds to Lose	Goal Weight (7% Loss)
341	24	317
342	24	318
343	24	319
344	24	320
345	24	321
346	24	322
347	24	323
348	24	324
349	24	325
350	25	325
351	25	326
352	25	327
353	25	328
354	25	329
355	25	330
356	25	331
357	25	332
358	25	333
359	25	334
360	25	335
361	25	336
362	25	337
363	25	338
364	25	339
365	26	339
366	26	340
367	26	341
368	26	342
369	26	343
370	26	344
371	26	345
372	26	346
373	26	347
374	26	348
375	26	349
376	26	350
377	26	351
378	26	352
379	27	352

Starting Weight	Pounds to Lose	Goal Weight (7% Loss)
380	27	352
381	27	354
382	27	355
383	27	356
384	27	357
385	27	358
386	27	359
387	27	360
388	27	361
389	27	362
390	27	363
391	27	364
392	27	365
393	28	365
394	28	366
395	28	367
396	28	368
397	28	369
398	28	370
399	28	371
400	28	372
401	28	373
402	28	374
403	28	375
404	28	376
405	28	377
406	28	378
407	28	379
408	29	379
409	29	380
410	29	381
411	29	382
412	29	383
413	29	384
414	29	385
415	29	386
416	29	387
417	29	388
418	29	389

Copyright 2017 University of Pittsburgh

Starting Weight	Pounds to Lose	Goal Weight (7% Loss)
419	29	390
420	29	391
421	29	392
422	30	392
423	30	393
424	30	394
425	30	395
426	30	396
427	30	397
428	30	398
429	30	399
430	30	400
431	30	401
432	30	402
433	30	403
434	30	404

Starting Weight	Pounds to Lose	Goal Weight (7% Loss)
435	30	405
436	31	405
437	31	406
438	31	407
439	31	408
440	31	409
441	31	410
442	31	411
443	31	412
444	31	413
445	31	414
446	31	415
447	31	416
448	31	417
449	31	418
450	31	419

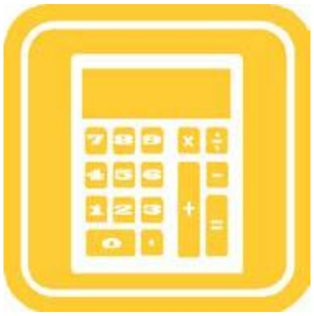

Keep an eye on *your* 7% weight loss goal.

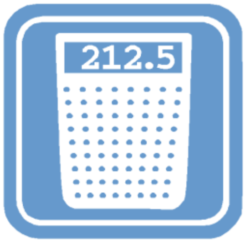

How Reaching the Group Lifestyle Balance™ Goals Can Help You

Losing weight and being more physically active:

1. May prevent diabetes and the metabolic syndrome.
   The DPP proved this to be true for many people.

2. Will help you be healthier, look better, and feel better.

Research has shown that being active and losing weight can:

- Ease tension, help you relax and sleep.
- Give you more energy, and make it easier to get around.
- Lower blood pressure.
- Lower blood levels of LDL ("bad" cholesterol or fat in the blood).
- Raise blood levels of HDL ("good" cholesterol or fat in the blood).

3. Will set a good example for your family, friends, and community.

Changing eating and activity behaviors takes work.

You *can* do it and we are here to help.

Copyright 2017 University of Pittsburgh

Group Lifestyle Balance™
Program Design

We will help you reach the study goals by making gradual, healthy, and reasonable changes in your eating and activity. The kind of changes you can stick with for a lifetime.

In this program you will learn:

- Facts about healthy eating and being active.
- What makes it hard for you to eat healthy and be active.
- How to change these things so they work *for* you, not against you.

For example, you'll learn how to:

- Be more aware of what you are eating and how to make healthy food choices.
- Be more aware of how much activity you do in a day and find time to be active.
- Ask for what you want when you eat out.
- Keep things around you at home and at work that make it easier for you to be active and make healthy food choices. Get rid of things that get in your way.
- Stop self-defeating thoughts and replace them with positive ones.
- Get back on your feet again when you slip from your plans for healthy eating and being active.
- Handle stress, social events, and other people that make it hard for you to change.

Copyright 2017 University of Pittsburgh

Group Lifestyle Balance™
Sessions

Group Lifestyle Balance™ (GLB) includes 22 sessions, which are held over the course of about one year.

Month 1	1. Welcome to the Diabetes Prevention Program: Group Lifestyle Balance™
	2: Be a Calorie Detective
	3: Healthy Eating
	4: Move Those Muscles
Month 2	5: Tip the Calorie Balance
	6: Take Charge of What's Around You
	7: Problem Solving
	8: Step Up Your Physical Activity Plan
Month 3	9: Manage Slips and Self-Defeating Thoughts
	10: Four Keys to Healthy Eating Out
	11: Make Social Cues Work for You
	12: Ways to Stay Motivated
Month 4	13. Strengthen Your Physical Activity Plan
	14: Take Charge of Your Lifestyle
Month 5	15: Mindful Eating, Mindful Movement
Month 6	16: Manage Your Stress
Month 7	17: Sit Less for Your Health
Month 8	18: More Volume, Fewer Calories
Month 9	19: Stay Active
Month 10	20: Balance Your Thoughts
Month 11	21: Heart Health
Month 12	22: Look Back and Look Forward

Copyright 2017 University of Pittsburgh

We Will Work
Together as a TEAM

We will count on you to:

- Attend or view the sessions each week and follow your handouts.
- Do your best to reach your goals for eating and activity. That includes doing assigned activities to practice what you learn.
- Keep track of your eating and activity seven days a week. Be honest.
- Keep track of your weight.
- Do your very best to tackle these changes now, while you have support from your coach and the group.
- Let your Lifestyle Coach know if you have any problems.
- Stay willing and open to change. Always "hang in there."

You can count on us to:

- Present accurate facts about healthy eating, physical activity, and weight loss.
- Answer your questions.
- Be honest.
- Believe you can reach your weight, eating and activity goals.
- Always "hang in there" for you.
- Support and help you.

We agree to work together in the ways described above.

Signed: ____

Date: ____

Lifestyle Coach: ____

Copyright 2017 University of Pittsburgh

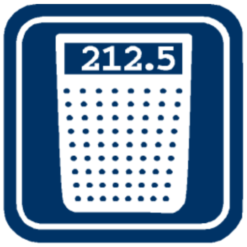

Getting Started Losing Weight

The first step in behavior change is to be aware. Let’s get going.

Be aware of how much you weigh.

- You will be weighed at every session.
- Weigh yourself at home, at least once a week, at the same time of day. Use the same scale from week to week.
- Make peace with the scale. If you have stopped self-weighing, it might feel hard to do this. We will help you with this important behavior.
- Record your weight on the Food and Activity record.

Be aware of what you eat and drink.

- The goal is to help you follow a healthy eating pattern.
- You will find ways to eat fewer calories.
- Being overweight or obese is related to heart disease, diabetes, and other health problems.

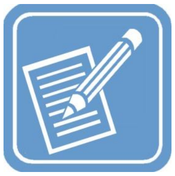

To begin, keep track of everything you eat and drink every day. It's the MOST IMPORTANT part of changing your eating habits.

- Your coach may provide you with Food and Activity books to record your eating, physical activity, and weight.

- If you choose to use a digital option, talk to your coach about the best way to share your weekly Food and Activity record.

Spelling in NOT important. When keeping track it is important to:

- Be honest (record what you really eat).
- Be accurate (measure portions, read labels).
- Be complete (include everything).

Copyright 2017 University of Pittsburgh

Practice Keeping Track

Time	Food: Description and Amount	Calories	Fat Grams
	Daily Totals:		

Physical Activity Description	Minutes	Steps
Daily Activity Totals:		

Copyright 2017 University of Pittsburgh

To Do:

Check the boxes when you complete each item:

☐ Keep track of your weight.

- Weigh yourself at home at least once a week, at the same time of day. Use the same scale.
- Record your weight on the Food and Activity record.
- Talk to your coach if you don't have a scale for self-weighing.

☐ Record everything you eat and drink every day.

- Use one line for each food or drink.
- Include:
  - Time.
  - The amount and name of the food or drink and a description.
  - Skip the calorie and fat columns for now.

☐ Buy a set of measuring cups and spoons for next week (if you don't already have them). A food scale that weighs in ounces is useful but not required.

☐ Start thinking about physical activity.

- What do you do now?
- What might you like to do? (You will track this later.)

☐ Talk with your health care provider.

- Let your health care provider know that you will be making changes in your eating, activity and weight. You may be advised to limit or avoid certain activities.

☐ **Bring to next week's session:**

- Your GLB notebook
- Your Food and Activity record.

Copyright 2017 University of Pittsburgh

Session 1: Resources | Page

Session 1: Resources	Page
Group Lifestyle Balance™ Weekly Record	21-22
How Am I Doing? Sample Tracking Weight Loss	23
How Am I Doing? Tracking Weight Loss	24-25

Copyright 2017 University of Pittsburgh

Name ____

Weekly Record

Use this log to record your progress throughout the upcoming year.

My Goals:

Calories _________ Fat grams ____ 7% weight loss ______

Session	Date	Weight (pounds)	Activity Minutes	Steps
1				
2				
3				
4				
5				
6				
7				
8				
9				
10				
11				
12				
13				
14				
15				
16				

Copyright 2017 University of Pittsburgh

Session	Date	Weight (pounds)	Activity Minutes	Steps
17				
18				
19				
20				
21				
22				

Copyright 2017 University of Pittsburgh

HOW AM I DOING? **Tracking Weight Loss** **Name:** ______John Smith______________________ **Goal Weight:** __246__ **Year:** _2017__

1. In upper shaded block, write down current weight (ex: 265). **2.** Below the same shaded block, write pound increments in decreasing order (ex: 264,263,262).
3. Determine 7% weight loss goal and make a straight line across the page (see example). **4.** In the lower shaded block, write down today's date (ex: 5/18); the next block to the right will be 5/25, etc. **5.** Track your weight weekly for the next six months. **YOUR GOAL IS TO REACH AND THEN STAY AT OR BELOW THE GOAL LINE!**

SAMPLE

265	x																								
264		x																							
263				x																					
262			x																						
261					x																				
260								x																	
259						x	x																		
258																									
257									x																
256													x												
255										x	x														
254												x		x											
253															x	x									
252																	x								
251																									
250																		x							
249																									
248																			x		x				
247																									
246																				x		x			
245																							x	x	
241																									
Week	1	2	3	4	5	6	7	8	9	10	11	12	13	14	15	16	17	18	19	20	21	22	23	24	25
Date	5/18	5/25	6/1	6/8	6/15	6/22	6/29	7/6	7/13	7/20	7/27	8/3	8/10	8/17	8/24	8/31	9/7	9/14	9/21	9/28	10/5	10/12	10/19	10/26	11/2

Copyright 2017 University of Pittsburgh

HOW AM I DOING? Tracking Weight Loss **Name:** ____________________________________ **Goal Weight:** __________ **Year:** _______

1. In upper shaded block, write down current weight. **2.** Below the same shaded block, write pound increments in decreasing order. **3.** Determine 7% weight loss goal and make a straight line across the page (see example). **4.** In the lower shaded block, write down today's date (ex: 5/18); the next block to the right will be 5/25, etc.
5. Track your weight weekly for the next six months. **YOUR GOAL IS TO REACH AND THEN STAY AT OR BELOW THE GOAL LINE.**

Week	1	2	3	4	5	6	7	8	9	10	11	12	13	14	15	16	17	18	19	20	21	22	23	24	25
Date																									

Copyright 2017 University of Pittsburgh

HOW AM I DOING? Tracking Weight Loss

Name: ______________________________________ **Goal Weight:** __________ **Year:** _______

1. Determine 7% (or personal) weight loss goal and make a straight line across the page. **2.** Continue to track your weekly weight.

YOUR GOAL IS TO REACH AND THEN STAY AT OR BELOW THE GOAL LINE.

Week	26	27	28	29	30	31	32	33	34	35	36	37	38	39	40	41	42	43	44	45	46	47	48	49	50
Date																									

Copyright 2017 University of Pittsburgh

Session 1 Leader Guide:
Welcome to the Diabetes Prevention Program - Group Lifestyle Balance™

Objectives

In this session, the participants will:

- Meet the lifestyle coach and other group leader(s).
- Learn about session meeting dates, times, locations, ways to stay in contact.
- Be given the Diabetes Prevention Program - Group Lifestyle Balance™ (GLB) notebook and handouts for Session 1.
- Discuss guidelines for being a good group participant.
- Discuss their initial thoughts about joining GLB.
- Learn about the research that supports the Diabetes Prevention Program (DPP).
- Review conditions that increase risk for diabetes, pre-diabetes, and the metabolic syndrome.
- Receive an overview of GLB.
- Learn the two main GLB goals and why they are important.
- Determine their 7% weight loss goal.
- Review session topics and the scheduled frequency of contact for the program.
- Discuss key aspects of the lifestyle coach-participant working relationship. Sign the lifestyle coach-participant behavioral agreement.
- Begin to record their weight and be assigned self-monitoring of weight.
- Learn the reason for and basic principles of self-monitoring.
- Learn the importance of measuring foods.

To Do Before the Session:

- Access the most recent version of the session handouts from the Diabetes Prevention Support Center (DPSC) website. https://www.diabetesprevention.pitt.edu/
- Log into the GLB Providers Portal to review additional program implementation resources: https://www.diabetesprevention.pitt.edu/my/login.aspx
- Decide how you will instruct participants to self-monitor. Examples include the GLB Keeping Track books or other written or digital food and activity tracking options.
- If providing Food and Activity books, 2 books per participant, for Weeks 1 and 2.
- Prepare participant notebooks (insert the Session 1 materials).

  Please note: A one-year GLB session schedule is provided on Page 15 of the Session 1 participant handout. This is meant to be a guide, with the understanding that there will be variation due to holidays, staff schedules, etc. If applying for CDC recognition (https://nccd.cdc.gov/DDT_DPRP/applicationForm.aspx), the first 16 sessions must be offered within the first 6 months. The first 16 sessions should be delivered in the order listed; however, the last 6 sessions may be used interchangeably. We generally suggest that at least the first 12 sessions be delivered weekly, and then transition to biweekly and monthly as is feasible in your program setting.

Copyright 2017 University of Pittsburgh

To Do Before the Session (continued):

- Sign the lifestyle coach-participant agreement if you are not doing so during the session.
- Determine how participants will contact the lifestyle coach/program organizer for absences and other concerns. Print contact information on the cover page.
- Have a set of measuring cups and spoons, a liquid measuring cup, a ruler, and (optional) a food scale that weighs in ounces (for display).
- Be prepared with information on where one can buy inexpensive measuring tools.
- Make a plan for participants who do not have access to a bathroom scale at home.
- Invite family members to attend with the participant (if appropriate in your setting).
- Determine how the lifestyle coach will document attendance and weight at the scale. Options include using the **Weekly Record** (pages 21-22 in the Session 1 handout; one for each participant), the **Group Lifestyle Balance Leader's Log** (GLB Providers Portal), or another preferred method. Keep all records secure/confidential.
- Have paper towels or wipes available for covering/cleaning the base of the scale (as needed).
- It is helpful to print a GLB Meeting Schedule with dates of each session.
- If lifestyle coaches are delivering the program in community locations, determine secure storage methods for scale and other materials (e.g., locked file cabinet).
- Determine a protocol for managing absences. Decide whether and how you will conduct make-up sessions in your program. There are an increasing number of options, simply be clear and consistent about your expectations and procedures. Examples include: mail or email the session material, create a website access, conduct a make-up session by phone or other digital means. You may wish to provide session material ahead of time to participants who inform you of planned absences.
- **If your program requires health care provider approval to participate, determine appropriate procedures for your participants and setting.**

Available in the GLB Providers Portal:
https://www.diabetesprevention.pitt.edu/my/login.aspx

- GLB Keeping Track books for self-monitoring (PDF for self-printing and assembly and ordering information for professional printing)
- **Group Lifestyle Balance Leader's Log**
- GLB Health Care Provider Approval to Participate template
- Group session meeting schedule template
- **Electronic Tools for Self-Monitoring** handout

Copyright 2017 University of Pittsburgh

General Session Procedures:

- As participants arrive, weigh each person privately. This may occur in the classroom, but scale display and participant weight record should not be visible to others.
- Maintain safety of participants as they step on and off the scale. Make sure weighing area is accessible and chairs are available for those needing extra assistance.
- Participants should remove shoes before weighing.
- Distribute participant notebooks with weekly session material.
- Ask participants to keep the **Weekly Record** (pages 21-22 in the Session 1 handout) at the front of participant notebook for easy access and self-monitoring of weight.
- It is advisable to weigh latecomers following the meeting.
- Conduct group session. Aim to start and end on time. Expected session length is about one hour. You may want to allow more time for the initial session.
- GLB sessions are meant to be highly interactive. Balance session content with prompts for group discussion. Ask if there are any questions, following each section.

General introductions

[The first step is to introduce yourself and ask participants to introduce themselves. Also welcome any family members or other support people present as appropriate.]

Hello. I'm delighted to meet you. My name is [ ]. Welcome to the Diabetes Prevention Program - Group Lifestyle Balance, or GLB.

[Briefly describe your background and qualifications for leading the group. Introduce any other group leaders. Describe your roles.]

GLB is directly adapted from a large clinical research study called the Diabetes Prevention Program, which we will discuss further in a few minutes. The GLB is designed to help you make lifestyle changes and to lose weight through healthy eating and being more physically active. These lifestyle changes have been shown to help reduce risk factors for diabetes and heart disease. This program is called "Group Lifestyle Balance" because we'll be helping you reach a healthy balance between what you eat and how active you are.

Now I'd like to ask each of you to introduce yourselves.

[Provide a few brief questions for each participant in the group to answer, such as to give their name and state something about themselves. Or use another brief ice breaker.]

Before we move on to the session material for today, let's go over some housekeeping details.

Copyright 2017 University of Pittsburgh

Consider discussing these points to inform and manage participant expectations:

- Access to building, meeting room, parking, bathrooms, and other details.
- Weekly weigh-in routine typically includes weighing by the lifestyle coach before each meeting, recording the weight on the **Weekly Record.**
- Review procedures for the weigh-in. Emphasize privacy of weight records.
- Time does not permit extensive coaching at the scale. Participants may address personal concerns after class or at another scheduled time.
- Latecomers will be weighed after class.
- Class time is about one hour.

Introduce the participant notebook

The notebook I've given you will be used throughout the program. It's yours to keep. At every session I'll give you some handouts to put into the notebook, and we'll go over them together.

Feel free to write notes or questions on the handouts, and take the notebook home. Just be sure to bring it with you to every session.

Please write your name on page 1. Here you will see my name and contact information. *[Another option is for you to write your name and contact information on the board. Ask participants to write it on their handout.]*

It's very important that we stay in touch.

I'm looking forward to working with all of you to make our time together a success. How we work together as a group will be very important.

[Use this opportunity to talk about whether or how you will conduct make-up sessions in your program.]

Session 1:
Welcome to the Diabetes Prevention Program: Group Lifestyle Balance™ (page 3)

*[Review **Be a Good Group Member** on page 3. Ask the participants if they have other suggestions.]*

[Encourage attendance. Mention that many studies show that greater session attendance is related to greater program success. Group members are a great source of support, encouragement, and helpful suggestions. Some people tell us that learning in a group is fun and rewarding.]

Copyright 2017 University of Pittsburgh

Remember Your Purpose (page 4)

I'm sure all of you have certain expectations and maybe even hesitations about joining this program.

Let's talk about your initial thoughts about being here.

[Based on group size, budget 10-15 minutes to discuss the three questions on page 4. Ask the questions one at a time. Encourage participants to share thoughts and ideas (and jot them down) during group discussion.]

- What do you think about being in GLB?
- Why did you join?
- What do you hope to achieve by taking part?
- How will healthy eating and being active help you or others?

The Diabetes Prevention Program (page 5)

An important study showed that making these lifestyle changes and keeping them up over time can **prevent diabetes** in people like you who are at risk of diabetes. The study was called "**The Diabetes Prevention Program**." Information about this study is found on page 5.

The Diabetes Prevention Program (or DPP) was conducted at 27 health centers around the country. The DPP was funded by the National Institutes of Health.

More than 3,000 adults took part in the DPP. All were at high risk for type 2 diabetes. This means that they had a condition known as "pre-diabetes". People with pre-diabetes are at increased risk for developing diabetes.

[Review the definition of pre-diabetes.]

[Review information about the study participants and the three groups.]

The DPP was a great success. The volunteers were followed for about 3 years.

[Review the results of the DPP. Emphasize that losing weight and being active have been shown to lower the chances of getting type 2 diabetes.]

The original DPP participants continue to be followed in what is called the DPP Outcomes Study. There continue to be positive benefits of modest weight loss and physical activity for many years.

Copyright 2017 University of Pittsburgh

As a result of the DPP, many national, state, and local groups have begun programs like this one to help people lose weight and be more active. Now there are programs based on the DPP all over the world, including GLB. [*Feel free to name some of the places that you think would resonate with your group.]*

The DPP and the Metabolic Syndrome (page 6)

The DPP also showed that losing weight and being active can reduce the risk of what's called "metabolic syndrome." Someone with metabolic syndrome has three or more of the risk factors listed on page 6. *[Review the list of risk factors.]*

One of every four American adults has metabolic syndrome. *[Review the list of health risks associated with metabolic syndrome.]*

The DPP lifestyle program reduced the risk of metabolic syndrome by 41%.
GLB is designed to help you make lifestyle changes to both prevent diabetes and prevent or treat the metabolic syndrome.

Group Lifestyle Balance™ Goals (page 7)

This program is called "Lifestyle Balance" because we will help you reach a healthy balance between two parts of your lifestyle:

1. What you eat, and
2. How physically active you are.

The GLB goals are the same as the DPP lifestyle goals. Your goals will be to:

1. Lose weight through healthy eating, and
2. Be more physically active.

[Emphasize that many studies have shown that these goals are safe, can be reached, and kept up over time.]

The GLB goals are listed on page 7:

1. **Lose 7% of your weight**
   [Review the information in the Goal 1 text box. Emphasize that they will lose weight at a pace that is safe and do-able; about 1-2 pounds per week.]

   [Use the following to help participants find their goal weight.]

   Turn to page 8, **"Group Lifestyle Balance™ Goal Weights".**

   Find your starting weight in the first column of the boxes on pages 8-12.

Copyright 2017 University of Pittsburgh

Next, read across the row. The second column shows the number of pounds you will need to lose to reach the goal. The third column shows your goal weight.

Write your pounds to lose in the text box on page 7 where it says "Pounds to lose". Also write your goal weight where it says "Goal weight (7% loss)".

Please take out your "Weekly Record". Write your 7% goal weight on the line provided at the top of the page.

[Note: The next time you weigh participants, check the goal they have written down to make sure it is correct.]

Many people reach the 7% weight loss goal by about 4-6 months from starting the program.

To help you reach your weight goal, we will give you a goal for calories and fat grams. This will get you started. I'll explain more about these goals in the next session.

2. **Do at least 150 minutes of physical activity each week.**

   The second main goal of GLB is to **do 150 minutes of physical activity each week** (this would be like taking a brisk walk for 30 minutes on five days of the week).

   [Review the information in the Goal 2 text box. Emphasize that they will reach their activity goal at a pace that is safe and do-able.]

We will help you to reach these goals by making gradual, healthy, and reasonable changes in your eating and activity behaviors.

We'll go over each of these goals in detail, and exactly what they mean for you, as we go along in GLB. You may also have your own personal goals you want to reach, but we encourage you to start with the GLB goals because they have been shown to work with many people. I will do everything I can to help you reach your eating, activity, and weight goals.

Some participants will have their own personal goals and want to lose less or more weight than 7%. Some may already be very active; others may wish to do less than 150 minutes of activity each week. Briefly address their concerns. Here are some examples of ways to address individual concerns:

- If the participant *wants to lose less weight or be less active* than the program goal: "We'll work toward this goal slowly, one step at a time. It's a safe and reasonable goal for you, and I'm very confident that you can do it with our support."
- If the participant *wants to lose more weight or be more active* than the program goal: "Let's work toward this goal first. When you reach this goal, we'll talk about going further."

Copyright 2017 University of Pittsburgh

How Reaching the Group Lifestyle Balance™ Goals Can Help You (page 13)

The GLB goals are **safe and can be reached.**

There is nothing extreme. For example, "Being active" doesn't mean you need to be a marathon runner, although you can if you want to and you train for it. We will help you gradually increase your general level of activity and build a more active lifestyle in a way that works for you.

[Review the information on page 13. Additional topics are listed below.]

1. **May prevent diabetes and the metabolic syndrome.**
   The Diabetes Prevention Program proved this to be true for many people. It showed that losing weight and staying active can prevent diabetes. Moderate weight loss and physical activity improve the body's use of insulin (the hormone that regulates the amount of sugar in your blood).

2. **Will help you be healthier, look better, and feel better**
   Many of you may have health problems like high blood pressure or high blood cholesterol. Research has shown that losing weight and/or being active has many health benefits. *[Review the list. Ask participants to identify which are the most important to them. Discuss.]*

3. **Will set a good example for your family, friends, and community.**
   Many of us live in a family or a culture with high-calorie eating and inactivity. You will face a challenge as you work at doing things differently. But you will also set a good example of what it's like to live a healthier lifestyle, which can be inspiring and encouraging to everyone around you.

Changing your eating and activity behaviors takes work. It takes dedication, hanging in there, and doing what needs to be done every step of the way.

We are here to help. I will do everything **I** can to help all of you reach and stick with your eating, activity, and weight goals. I am confident that **you can do it!**

Your fellow group members will also be a great source of support, encouragement, and helpful suggestions.

Group Lifestyle Balance™ Program Design (page 14)

GLB has been carefully designed. It is based on many research studies of the best ways to help people change. GLB **is NOT a diet; it is a lifestyle change program.**

Copyright 2017 University of Pittsburgh

In this program you will learn:

- **Facts about healthy eating and being active.** We will give you the most up-to date and accurate information.
  But knowing the facts, or what to change, isn't enough. You also need to know **how to change.** So you will also learn:
- **What makes it hard for you to eat healthy and be active.**
- And learn **how to change these things so they work *for* you**, not against you.
  For example, you'll learn how to:
  [Review the information in the text box.]

I will also give you the **long-term support** you need to stick with the changes you make. I will be your **"lifestyle coach."**

Group Lifestyle Balance Sessions (page 15)

Page 15 shows you the topics for **Sessions 1 through 22**. *[If it is appropriate for your setting, tell participants they are welcome to invite a family member or friend to any or all of the sessions.]*

We Will Work Together as a TEAM (page 16)

It is very important that we work together throughout the program as a **team**. Let's review how we will work together as a team.

[Review "We will count on you to". Additional information to include:]

- **Attend the sessions.** *[Ask that participants let you know the week before if they are going to be absent the next week. Also explain how you want participants to contact you if they will miss a meeting. For example, phone call, text message, or email.]*

- **Keep track of your eating and activity 7 days a week.** I'll talk with you more about this in a few minutes. **Be honest.** Don't try to "please" me. I will count on you to write down what you are really eating, and how active you really are, not what you think I want to hear or what would make me happy.

- **Keep track of your weight at home.** You will be weighed at each group meeting. By weighing yourself at home, you will be able to see the pattern of your weight from day to day and see how your changes in eating and activity affect your weight.

Copyright 2017 University of Pittsburgh

- **Try your very best to tackle these changes now**, while you have support from your coach and the group. Then you will be ready to practice for longer periods on your own when we meet less often later in the year….and to continue these healthier eating and activity behaviors for a lifetime.

- **Let me know if you have any problems.** Ask questions when you don't understand something. I am here to help and I need to know when you're having any difficulties. There's no such thing as a "stupid" question--it's *smart* to speak up when you have a question.

[Note: Some participants, because of their cultural heritage or personal history, may consider it rude to ask questions or to bring up difficulties. Be sensitive to the values and norms of your group. Express your acceptance and appreciation when they voice their questions and concerns.]

- **Stay willing and open to change. Always "hang in there."** To change your weight and health, you must change your eating and activity behaviors. Through trial and error, you will find what works best for you. If you run into problems, I will count on you to hang in there and give it your best until we solve the problems together. This is a "can do" program.

[Review "You can count on us to" and include the following:]

- **Present accurate facts about healthy eating, physical activity, and weight loss.** GLB is evidence-based and strives to present current research.in a way that helps you understand it more clearly. However, we understand that the amount of information can be overwhelming at times. Sometimes studies contradict each other. **We will aim to "keep it simple".**

- **Answer your questions.** It's important that you feel free to ask me any questions you have, and I will get the answers for you. Please remember that the program staff members are all appropriately trained, and our job is to help you in any way we can.

- **Be honest.** We will both need to "say it like it is." I will count on you to be honest about how you are doing. And you can count on me to listen and encourage you and the group to solve problems together.

- **Believe you can reach your eating, activity and weight management goals**. We all need someone to believe in us when we are making changes for the better. I know you can do it, and when you get discouraged, I will be here to believe in you. Noticing what you're doing **well** is one of my most important jobs. I will encourage you and build you up and appreciate your efforts.

- **Always "hang in there" for you.** The entire GLB staff will **support and help you** throughout the program. As you make lifestyle changes, there may be times when you struggle to stay focused on your goals. This is when it is **most**

Copyright 2017 University of Pittsburgh

important to keep in touch with me and to attend group meetings so that we can work together to keep on track. Your fellow group members can also help to support and encourage your lifestyle change efforts.

Is there anything else you'd like me to do to help you? *[Explain whether or not you will be able to respond to these requests and suggest other sources that might be able to address their needs if you and the program will not be able to so.]*

I hope we will work together in the ways we just discussed.

Please sign the agreement if you agree to this kind of team approach.

[Give participants time to sign the behavioral agreement. If you did not sign the agreement before class, walk around the room and sign the page for each participant.]

Getting Started Losing Weight (page 17)

Now let's move on to the next **part of this session: Getting Started Losing Weight.** Turn to page 17.

Before you start to make changes in your eating and activity behaviors, it is important for you to know what you are doing right now. The first step toward change is to be aware.

[Review information on page 17. Include the following:]

Be aware of how much you weigh

- It is important for you to get in the habit of weighing yourself at home. Regular self-weighing has been shown to be a very important behavior in weight management. Please weigh yourself at home at least once a week. Daily may be even better.
- Expect to see about a 1-2 pound weight loss per week. Of course, most people don't lose the same amount of weight each week. Many people lose weight faster at first and then the rate levels off. We won't pay as much attention to each weight but rather to the pattern over time.
- Always weigh yourself on the same scale because your weight can vary from one scale to another.
- And weigh yourself at the same time of day, because that can make a difference too.
- What time of day would be best for you?

> If a participant doesn't have a scale and cannot afford to buy one, speak with him/her privately about the fact that self-weighing (outside of the program) is an important behavior for long-term weight management. Discuss options (at a friend's house, community center, gym) or make other provisions.

Copyright 2017 University of Pittsburgh

Be aware of what you eat and drink.

The goal in GLB is to help you find and follow a healthy eating pattern that works for you.

Please be open-minded and willing to change. By using, experimenting with, and applying information you learn in GLB, you will learn to make healthier food choices and find ways to eat fewer calories.

Keep your eye on the prize of reducing your risk of heart disease, diabetes, and other health problems by losing weight, eating a heathier diet, and being more active.

To help you be aware of your food intake, you will keep track of everything you eat and drink every day. This is the MOST IMPORTANT part of changing your eating habits/behaviors. This is called "self-monitoring". Research demonstrates that self-monitoring is a key behavior for weight management. This is something you're going to do throughout the program to help you be successful in reaching and maintaining your goals.

The first step is to **record everything you eat and drink.**

[Explain your program's preferred option for self-monitoring and sharing of the weekly record.]

You may choose to write down everything using a Food and Activity book or use a digital tracking option. *[Optional: Distribute the* ***Electronic Tools for Self-Monitoring*** *handout.]*

Keeping track of what you eat will help you and I see:

- What foods you eat
- How much you eat
- When and where you eat, and
- How your eating habits change over time.

Your Food and Activity records will be the very basis for our working together. You and I will be the only ones to see them, so **spelling is NOT important.**

You can make up **abbreviations** or use your own shorthand if that makes it easier and faster for you to keep track, just so we both know what you mean.

[Note: The use of abbreviations may also help those participants who have difficulty spelling feel less self-conscious.]

What IS important is to:

- **Be honest.** That means to record **what you really eat**, not just what you think will please me or yourself.

Copyright 2017 University of Pittsburgh

- Also, **be accurate**. It's best to record what you eat as soon as possible after you eat it, because it's easy to forget. For example, count the number of slices of cheese you eat and record the kind of cheese. Measure portions and read labels.
- And **be complete**. **Include everything.** The butter on the toast, the cream in the coffee, and the mayonnaise on the sandwich.

It may seem hard to record all of your foods, especially at first. And it does take some time. But it's worth its weight in gold.

Being aware of what you are eating is the first step toward changing your eating habits/behaviors.

Research shows that **people who self-monitor lose more weight than those who don't.**

If using a Food and Activity book, distribute one to each participant.

Ask participants to open the book and notice where they should record the day of the week and date at the top left side of the page. Explain that the Calorie Goal and Fat Gram goal should be left blank for the first week; the focus this week is on getting into the practice of writing things down. The section for Physical Activity does not need to be completed this week either.

Explain that the left and right side is to be used for 1 day. There is space to record 7 days in each book. Explain that they should record the time of day every time they eat. Then measure and write down what they eat and drink.

Encourage participants to record food and drinks while they are eating or as soon as possible afterward; this leads to much greater awareness and accuracy than trying to "think back". Ask participants to draw an X through the pages for the first day and explain that they should start recording tomorrow morning and continue for the next 6 days. A new book is started on the morning of every group meeting, and they will turn in the old book for feedback.

Next, ask them to turn to the back page of the book and write their name and today's date next to "Start Date" on the bottom of the page. For this first week, the only information that needs to be recorded is weight. Draw a line through the "Day 1". Participants will start recording tomorrow, Day 2. Indicate the weight column on the back, and ask participants to circle the day(s) on which they plan to weigh themselves. More information about self-monitoring will be provided next week.

Distribute a second Food and Activity book to each participant. Giving out two books initially will ensure that no days of recording are missed.

Because a new book is started on the morning of every group meeting, a second book is provided.

Ask participants to write their name and next week's date next to "Start Date" on the back of the second book.

Copyright 2017 University of Pittsburgh

Practice Keeping Track (page 18)

Let's practice self-monitoring. Page 18 is a **"practice page."**

The columns to record calories and fat grams should be left blank for this week. You also don't need to record physical activity. *[Some participants are already physically active. They may choose to record their minutes of activity.]*
Let's talk about **what you should record**.

Think about what you ate earlier today, and write down some of those foods and beverages.

- In the first column, write down the time you ate the meal or snack.
- In the second column, record the amount (as best you can remember). In the third column, write down the item's name and a description. For example, baked chicken breast or 2% milk. Skip the columns for calories and fat grams for now- we will be discussing that in more detail next week.

P**ortion size is very, very important**. **Even a small difference in portion size can mean a big difference in calories.**

Starting this week, please measure everything you eat and drink, using measuring cups and spoons, a liquid measuring cup, and a ruler. *[Show measuring cups, measuring spoons, ruler, and food scale (optional).]*

A food scale that weighs in ounces may be helpful, but is not required. At the next session we'll go over measuring in more detail. Over time, you will become better and better at accurately estimating how much you are eating, and you'll only need to measure now and then. But for now, it's very important to measure everything.

Use a set of measuring cups for things like cereal, measuring spoons for things like salad dressing, and a liquid measuring cup for milk, juice, etc. If you have a food scale, it is used for weighing meats and cheese. The ruler is for measuring things like bagels, pizza, pieces of pie, and cookies. *[If participants don't have measuring cups and spoons, provide information on where to purchase inexpensive measuring tools.]*

Emphasize the importance of using them to learn portion sizes and portion control for foods they eat frequently at home.

Copyright 2017 University of Pittsburgh

Participants should leave this session aware that:

1. Their efforts to be honest and complete about their eating habits are more important than picture-perfect Food and Activity records, and
2. Self-monitoring a very important learning tool that everyone is expected to work on during the course of the program.

Participants should never be made to feel that they are unwelcome at group meetings because they did not self-monitor. They should never be asked to leave the program because they do not self-monitor.

To Do (page 19)

Each session ends with a list of activities for you to do during the coming week. They are designed to help you put into practice what you learned in the group meeting. This will help you build the skills you need to make positive changes in your eating and activity behaviors. It will not be collected and graded, but we will discuss it at our next group meeting. This process of learning, doing, and sharing your experience with the group is part of what helps you to succeed in Group Lifestyle Balance.

[Review information on page 19. Include the following:]

- Keep track of your weight.
  - Please let me know if you don't have a bathroom scale.

- Record everything you eat and drink every day.
  - Measure and record everything you eat and drink.
  - Do this every day, as soon as possible after you eat.
  - Keep your Food and Activity record with you during the day so you can record right away.
  - The goal for this week is for you to get used to self-monitoring and to become aware of your eating patterns.

- Start thinking about physical activity. Most participants choose walking but think about other activities you might like to do.

- Talk with your health care provider. It is important that s/he know that you will be making healthy lifestyle changes in your eating, activity, and weight. *[If your program requires health care provider approval to participate, explain your program's policy and procedures.]*

- Bring the completed Food and Activity record to next week's session. I will collect, review, and write comments. It will be returned to you the following week.

Copyright 2017 University of Pittsburgh

Resources (pages 20-25)

Page 20 lists 3 additional resources to include in this session.

You have already used pages 21-22, **Weekly Record**. For easy access, you might want to keep this handout in the front of your notebook. You will use this to record your weight at each of our group meetings. For this first week, please record today's date and your weight, if you haven't done so already. You may leave the "activity minutes" and "steps" columns blank. We will discuss tracking physical activity in an upcoming session. However, if you track your minutes and steps, feel free to record it here.

Many people enjoy tracking their weight loss progress on a graph. A sample graph is on page 23. Pages 24-25 have a graph for you to use, if desired.

If time permits, show the group how to mark their starting weight and goal weight on the graph on page 24 and to draw a line between the two. Explain that the line shows what a perfectly steady and gradual weight loss might look like for them. Qualify this by saying something like:

- Of course, most people's weight varies from week to week, and yours will probably do that, too, sometimes above this line, sometimes below it. Many people lose weight faster at first and then the rate levels off. We won't pay as much attention to each weight but rather to the pattern over time.
- Stay under this line as much as possible and reach your 7% goal weight by about 4-6 months (weeks 16-20). Maintaining your weight loss will be very important which is why we will teach you not only how to lose weight, but also how to keep it off!

[*Thank the participants for attending. Announce the time and place for the next session. Ask them to bring their GLB notebook to each session.]*

After the session:
- Weigh participants who did not do so prior to the group meeting.
- Complete data forms and documentation required in your setting.
- Follow your program's protocol for managing absences.

Copyright 2017 University of Pittsburgh

Session 2:
Be a Calorie Detective

To help you lose weight, we'll help you build healthy eating habits.

Healthy eating involves eating fewer calories, less fat, and less *unhealthy fat*.

- Eating fewer calories. Eating or drinking too many calories from *any type of food or drink* can cause weight gain. Being overweight or obese increases your risk of heart attack, stroke, and diabetes.

- Eating less fat. Fat has more than twice the calories as the same amount of sugar, starch, or protein. Even small amounts of high-fat foods are high in calories. With lower fat foods, you can eat more and feel fuller on fewer calories.

  Note: Low-fat or fat-free foods still contain calories. Read the label.

Compare	Calories*	Fat Grams*
2 cups of cheese curls (2⅜-ounce bag)	370	24
5 cups of 94% fat-free microwave popcorn	110	2

* These values are averages. Always check labels.

- Eating less *unhealthy fat.* Too much unhealthy fat can raise your level of "bad" cholesterol. It may also increase your chances of having a heart attack or stroke or getting diabetes.

Copyright 2017 University of Pittsburgh

Group Lifestyle Balance™
Calorie and Fat Gram Goals

To get started losing weight, stay as close as you can to your calorie and fat gram goals. They are shown in the chart below.

- Find your starting weight.
- Your calorie and fat gram goals are in the same row. Circle them.
- Write them on your Weekly Record.

Starting Weight (pounds)	Daily Calorie Goal	Daily Fat gram Goal
120-174	1,200	33
175-219	1,500	42
220-249	1,800	50
250 or more	2,000	55

- A gram is the way fat in food is measured. It is a unit of weight. A paper clip weighs about 1 gram.

About your calorie and fat gram goals:

- It may be hard to reach your goals at first. Just try to get as close to them as you can.
- Everyone is different. Research has shown that these goals are a good starting point. They are designed to help you lose about 1-2 pounds per week.
- Aiming for a goal of about 25% (one-quarter) of calories from fat means this is a moderate fat, not a low-fat eating plan.
- **It's important not to go too low.** Do NOT try to see how few calories and fat grams you can eat. You need to get enough food for health and good nutrition.

Copyright 2017 University of Pittsburgh

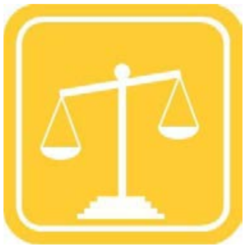

How and Why to Measure Foods

If you measure food at home, you will:

- Know the calories that you eat and drink.
- See what a moderate portion looks like.
- Be better at guessing amounts when eating away from home.

Most people are surprised when they measure foods. **Let's look** at some examples:

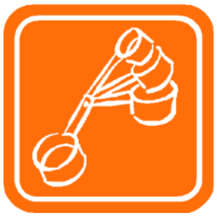

- Write down the name of the food.
- Guess the amount.
- Write down the actual amount.
- Figure the calorie and fat grams for the actual amount.

Food	Guessed Amount	Actual Amount	Calories	Fat Grams

Measuring foods will help you help you stay within your calorie and fat gram goals.

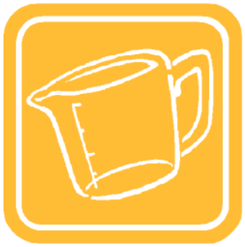

Measuring cups

- For solid foods: Fill and level off.
- For liquids: Fill. Read the line at eye level.

Measuring spoons

- Fill and level off.

Measuring/Estimating Meat and Cheese

- Estimate the portion size.
    - o 4 ounce raw = 3 ounce cooked (about the size of a deck of cards or the palm of your hand).
- Some people find it helpful to use a food scale.
    - o Weigh meats after they are cooked with the fat and bone removed.

Copyright 2017 University of Pittsburgh

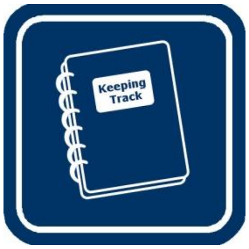

Keep Track of the Calories and Fat You Eat Every Day

1. Record everything you eat and drink. It's the most important part of changing your behavior. Spelling is NOT important. What IS important is to:
   - Be honest. (Record down what you really eat.)
   - Be accurate. (Measure portions, find calorie and fat information.)
   - Be complete. (Include everything.)

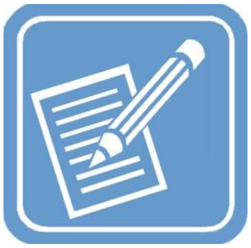

2. Figure out how many calorie and fat grams are in everything you eat and drink. Record it.
   - Find the serving size, calorie, and fat gram information by using:
     - Food labels. Always use label values if you can. They are the most up-to-date and exact.
     - Calorie and fat counter. Use a book or digital option.
     - Menus, menu boards, or displays at some restaurants, bakeries, and coffee shops.
   - Compare the amount YOU ate or drank with the amount listed on the label, Calorie and Fat Counter, or menu.
   - Figure out how many calorie and fat grams are in the amount you ate or drank.

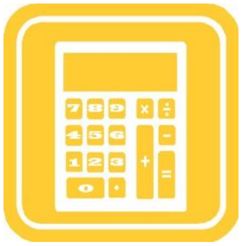

3. Add up the calorie and fat grams you eat and drink during the day.
   - Subtotal the calorie and fat grams in each meal and snack. Doing so will help you stay on track with your goals.
   - Transfer the daily totals of calories and fat grams to the back cover of your Keeping Track book.

Copyright 2017 University of Pittsburgh

Eat a Packaged Food?
Read the Nutrition Facts Label

Look at the Serving size.

Look at the Calories per serving.

Look at the Total Fat Grams per serving.

Question: What if you eat a larger serving than is listed on the label?

Answer: You will be eating more calories and fat grams than are listed on the label.

Nutrition Facts

8 servings per container
Serving size **2/3 cup (55g)**

Amount per serving
Calories **230**

	**% Daily Value***
**Total Fat** 8g	**10%**
Saturated Fat 1g	**5%**
Trans Fat 0g	
**Cholesterol** 0mg	**0%**
**Sodium** 160mg	**7%**
**Total Carbohydrate** 37g	**13%**
Dietary Fiber 4g	**14%**
Total Sugars 12g	
Includes 10g Added Sugars	**20%**
**Protein** 3g	
Vitamin D 2mcg	10%
Calcium 260mg	20%
Iron 8mg	45%
Potassium 235mg	6%

* The % Daily Value (DV) tells you how much a nutrient in a serving of food contributes to a daily diet. 2,000 calories a day is used for general nutrition advice.

Copyright 2017 University of Pittsburgh

When You Use the Calorie and Fat Counter

Can't find a food?

- Use the calories and fat grams for a food that's the most like it. For example, use nut bread for zucchini bread.

- Or write down the name of the food. Ask your Lifestyle Coach about it next week.

Having trouble figuring calories and fat grams?

- Just write down the food or drink and the amount.

- Your Lifestyle Coach will help you next week.

Making a recipe?

- For many recipes, you can simply record how much of each ingredient you ate.
  - For example, in a stew, how much meat did you eat? Carrots? And so on.
  - Have you made the recipe yourself? Add the calories and fat grams in all the ingredients. Divide the sum by the number of servings. That will give you the calorie and fat grams for each serving.

- Another option is to find a similar food in the Calorie and Fat Counter. Use that information.
  - For example, use the calorie and fat grams for beef stew.

Copyright 2017 University of Pittsburgh

Know Where Your Calories and Fat Grams Come From

Let's uncover the calories and fat grams in a sample meal:

Food	Calories	Fat Grams	Teaspoons of Fat
Quarter pound hamburger sandwich with cheese	730	44	11
French fries, large	500	22	5 ½
Chocolate milkshake (large, 20 ounces)	980	24	6
Total:	2210	90	22 ½<br>About 1 *stick* of butter

What kinds of foods do you eat that are high in calories and fat?

1.
2.
3.
4.
5.

Copyright 2017 University of Pittsburgh

Three Ways to Eat Fewer Calories

1. Eat high-calorie foods less often.

Example: Eat high-calorie desserts (cake, pie, premium ice cream) less often.

2. Eat smaller amounts of high-calorie foods.

Cutting back even a little on the amount you eat can make a big difference.

Example: Use 2 tablespoons instead of 4 tablespoons of salad dressing on your salad. This could save 145 calories and 15 grams of fat.

3. Eat lower-calorie foods instead.

Example: Eat broiled fish instead of fried fish.

Make a plan to eat fewer calories.

- Write down three foods you eat often that are high in calories.
- What are you willing to do to eat fewer calories?

Your Top Three High-Calorie Foods	The Three Ways to Eat Fewer Calories		
	Eat it only this (less) often:	Eat only this (smaller) amount:	Eat this (lower calorie) food instead:

Through trial and error you will find products and recipes you like.

Ask friends, family, and group members for ideas.

Copyright 2017 University of Pittsburgh

Practice Keeping Track

For each food below:

- Look up the food in the Calorie and Fat Counter.
- Figure out the calorie and fat grams in the amount you ate or drank.
- Subtotal each meal and snack.
- Total the calorie and fat grams for the day.

Time	Food: Description and Amount	Calories	Fat Grams
	Daily Totals		

Would you prefer to keep track using a digital option? No problem.

Talk to your coach about the best way to share your weekly record. Your coach can still give you feedback.

Copyright 2017 University of Pittsburgh

Practice Keeping Track

If you are using the GLB Keeping Track book, be sure to fill out the back page. Why?

1. It will let you review the week as a whole. Ask yourself:
   - Which days were higher or lower in calories, fat grams, and activity than usual? What happened on those days?
   - On average, are you meeting your goals?
   - Most important, how has your eating and physical activity affected the trend in your weight? What changes, if any, do you want to make next week?

2. It will help your coach to see your overall eating pattern and give you feedback.

My totals for the week

	Calories	Fat Grams	Minutes of Activity	Steps	Weight
Day 1					
Day 2					
Day 3					
Day 4					
Day 5					
Day 6					
Day 7					
Total					
Average					

Copyright 2017 University of Pittsburgh

To Do:

Check the boxes when you complete each item:

☐ Keep track of your weight.

- Weigh yourself at home at least once a week, at the same time of day. Use the same scale.
- Record your weight on the Food and Activity record.

☐ Record everything you eat and drink every day.

New things to practice:

☐ Come as close as you can to your calorie and fat gram goals.

- Use the Nutrition Facts label, Calorie and Fat Counter, and menus, menu boards or displays to figure out the calories and fat in what you eat and drink. Record this.
- Subtotal your calories and fat grams after each meal and snack.
- If you are using the GLB Keeping Track books, transfer the daily totals of calories and fat grams to the back page.

☐ Follow your plan to eat fewer calories (see page 8).

How did it go?

____

____

☐ Keep thinking about physical activity.

- What do you do now?
- What might you like to do? (You will track this later.)

Copyright 2017 University of Pittsburgh

Session 2 Leader Guide:
Be a Calorie Detective

Objectives
In this session, the participants will:

- Review self-monitoring skills, and learn in more detail how to measure and record foods and beverages.
- Determine their calorie and fat gram goals.
- Practice finding foods in the Calorie and Fat Counter and figuring out the number of calories and fat grams in foods and beverages.
- Practice finding information on the Nutrition Facts label.
- Review the importance of measuring foods.
- Learn to calculate running calorie and fat gram totals for the day.
- Learn the three ways to eat fewer calories.
- Discuss how making small changes in their food choices can make a big difference in their daily calorie intake.
- Make a plan to eat fewer calories.

To Do Before the Session:

- If providing Food and Activity books; one per participant.
- If providing a Calorie and Fat Counter; one per participant.
- Have measuring cups and spoons, liquid measuring cup, ruler, and food scale (optional) for display.
- Prepare Session 2 handout for participant notebook. Determine your preferred procedure for distribution (i.e. at the scale, in the classroom, etc.).
- Have food models or actual foods for measuring demonstration.
- (Optional): Have visuals to show graphically the fat content of common high fat foods. Relate to foods that you know the participants eat often. Useful visuals are teaspoons of fat on small plates or test tubes containing measured amounts of shortening.
- Log into the GLB Providers Portal to review additional program implementation resources: https://www.diabetesprevention.pitt.edu/my/login.aspx

Additional resources:

- 2015 Dietary Guidelines for Americans https://health.gov/dietaryguidelines/2015/
- Changes to the Nutrition Facts Label https://www.fda.gov/Food/GuidanceRegulation/GuidanceDocumentsRegulatoryInformation/LabelingNutrition/ucm385663.htm
- Menu and Vending Machines Labeling Requirements https://www.fda.gov/food/ingredientspackaginglabeling/labelingnutrition/ucm217762.htm

Copyright 2017 University of Pittsburgh

Available in the GLB Providers Portal:
https://www.diabetesprevention.pitt.edu/my/login.aspx
- **Guidelines for Reviewing Food and Activity Records**
- **The Secret to Serving Size is in Your Hand** handout
- **Portion Control: Size Matters** handout
- **Electronic Tools for Self-Monitoring** handout
- **Find the Calories and Fat Grams in a Recipe** handout
- Ordering information for Calorie and Fat Counter (**Calorie King®)**

General Session Procedures:
- Weigh each person privately. This may occur in the classroom, but scale display and participant weight record should not be visible to others.
- Record each participant's weight.
- Ask the participant to update his or her own **Weekly Record**. (Optional) Participants may also graph their weight.
- Check to make sure that the participant has correctly calculated his or her 7% weight loss goal and written it on his or her **Weekly Record.**
- Document each participant's 7% weight goal in the lifestyle coach's records.
- Maintain safety of participants as they step on and off the scale. Make sure weighing area is accessible and chairs are available for those needing extra assistance.
- If the participant has lost weight, express positive comments, but not excessively. Emphasize that s/he must already be making behavior changes.
- If the participant has not lost weight, be encouraging. Emphasize that little by little as s/he makes behavior changes, it will show up on the scale.
- Ask if the participant weighed himself or herself at home. Mention that home weights and weights taken at the session may differ because scales differ, but the trend in weight change over time should be the same.
- Collect completed Food and Activity records. If providing Food and Activity records, distribute blank ones.

Group Sharing Time (allow at least 5 minutes)

[Every class should begin with a discussion of the previous week and a review of the "To Do" home assignment. Use an optimistic approach. Strive for a positive and supportive atmosphere.]

In our last session you were introduced to Group Lifestyle Balance™. You learned that keeping track of everything you eat and drink will help you be aware of your food intake.

Copyright 2017 University of Pittsburgh

Let's take a few minutes to talk about your experience with self-monitoring this past week.

Were you able to record anything about your eating?
What did you learn by self-monitoring? What difficulties did you have?
What are your thoughts about weighing yourself during the week?
Did you start a new Food and Activity record this morning?

[Praise all efforts to self-monitor. Be positive and nonjudgmental. Encourage group discussion.]

[*Emphasize the importance of recording every day, even if they felt they had a "bad day". It will help them stay aware and learn about their eating behaviors.]*

Remind participants that you will review any Food and Activity record they provide. Indicate that you will not be grading them but rather encouraging and supporting their efforts at lifestyle change. Records will be returned at the next group meeting. Suggest that they keep them for future reference.

Make clear that the Food and Activity records are for their benefit. However, they should never feel unwelcome at a meeting or that they would be asked to leave the program for not completing a Food and Activity record.

Session 2: Be a Calorie Detective (page 1)

As we discussed last week, we will help you build healthy eating habits that will help you lose weight and improve health.

Healthy eating involves three key strategies: eating fewer calories, less fat, and less unhealthy fat. [*Review page 1 and include the following information:]*

Review the example on page 1 (a lot of calories in the higher-fat cheese curls versus fewer calories in more than twice the amount of lower-fat popcorn).

You may use other examples that are relevant to the participants' eating patterns.

Optional: Use food models of fat, fat in a test tube, or shortening measured onto a plate to show the amount of fat in 2 cups of cheese curls and 5 cups of 94% fat-free popcorn.

In addition, reduced/low or sugar-free foods may still contain calories as well.

Today we will talk about how to find the calorie information for the foods and beverages you consume.

Copyright 2017 University of Pittsburgh

Eating less unhealthy fat. In addition to eating less fat overall as a way to eat fewer calories, you will learn how to replace unhealthy types of fat with healthier choices. This is very important for your health. We will talk more about how to choose healthier types of fat in future lessons.

In our last session we discussed that one of the goals in GLB is to lose 7% of your weight. You know your 7% weight loss goal. Now we're going to talk about how to do this in a way that is slow, safe, and doable. First, let's talk about how many calories you should eat each day in order to lose weight.

Group Lifestyle Balance™ Calorie and Fat Gram Goals (page 2)

To help with losing weight and improving health, you are asked to try to stay as close as possible to a certain calorie and fat gram goal. The fat and calorie goals are based on body size and the number of calories needed to lose weight.

[Review directions for finding calorie and fat gram goals.]

Note: The following explanation is for only those participants who express interest in how their calorie goal has been calculated. Do not give this text to participants.

Question: How did you determine the number of calories I should be eating to lose weight?

Answer: The number of calories you need for weight loss depends on many factors, including your weight, how active you are, how old you are, and so on. But we can make a good guess and then see how the scale responds.

[*Review the information in the text box, "About your calorie and fat gram goals". Include the following:]*

- We don't expect you to reach your calorie and fat gram goals right away or be able to stay as close as possible to them every day. For now, just try to **get as close to your goals as you can.** During the next few sessions, we will teach you to make healthier food choices and how to plan and prepare lower calorie meals and snacks so that it is easier for you to reach your goals. Over time we'll work together so that you can consistently stay as close as possible to your calorie and fat goals.

- Staying within your calorie and fat gram goals should help you lose about 1-2 pounds per week. This is an average but the actual amount of weight lost will vary from week to week. Most people do not lose the same amount of weight each week. People often lose weight faster at first and then the rate levels off.

- Eating less fat is one way to reduce the number of calories you eat. The type of fat is very important and will be discussed in upcoming group meetings.

Copyright 2017 University of Pittsburgh

[The GLB fat gram goal is in line with the Institute of Medicine recommendation of 20-35% of total calories per day from fat.]

- We recommend that you don't go below 1200 calories per day in order to get enough food for health and good nutrition. Eating too few calories is not something you can stick with for a lifetime. Remember that GLB is NOT a diet; it is a lifestyle change program where the goal is for you to create a healthy eating pattern that you can stick with for a lifetime.

You can think of your calorie and fat goals as a budget because you need to stay as close as possible to these goals every day. Be consistent. This will help you establish eating behaviors that will help with losing weight and improving health.

How and Why to Measure Foods (page 3)

Measuring foods is very important. *[Review "If you measure food at home, you will" and include the following:]*

- Measuring will help you know the calories you eat and drink. This will give you a clear picture of what you are doing well and what changes you want to make. Even a small difference in portion size can mean a big difference in calories and fat grams.
- We live in a world of extremely large portions such as triple hamburgers, 64-ounce soft-drinks, and massive tubs of popcorn, marketed and sold as one serving. This is called "portion distortion". Measuring will help you see what a moderate portion looks like.
- We certainly don't expect you to measure food items when you are at parties and other social events. If you regularly measure foods at home, you will get better at judging food amounts just by looking.

Most people are surprised when they begin to measure foods. Our eyes can play tricks on us.

Copyright 2017 University of Pittsburgh

Show the participants the food models for common foods (or actual foods). Suggest a 3-ounce hamburger patty, a bowl of cereal, a serving of rice, one Tablespoon of peanut butter, or other foods that your participants frequently consume.

Review the instructions for how to complete the table on page 3.
Either hold up each food model or ask the participants to come up to the display and briefly guess the amounts, and return to their seats and guess the amount of calories and fat grams.
Tell participants the actual amounts of each food.
Then tell everyone the actual calories and grams of fat for each food.

Note: If you are short on time, focus only on having the participants guess the portion size and them tell them the actual amount. Omit looking up calories and fat grams.

Please continue to measure everything you eat and drink, using measuring cups and spoons, a liquid measuring cup, and a ruler. You may use a food scale that weighs in ounces, but that is optional.

Let me go over some details about how to use the measuring tools. You may be doing these things already.

[*Review the information in the text box at the bottom of page 3. Include the following information:]*

Measuring cups

- For solid foods: Leveling can make a big difference. A heaping measuring cup or spoon that hasn't been leveled will add calories which will really add up with all foods throughout the day.

[Discuss or demonstrate how to level measuring cups and/or measuring spoons.]

- For liquids: Use a liquid measuring cup for items such as milk, juice, or soup. Pour the liquid in the cup then read the line at eye level while the cup is resting on a flat surface (such as the kitchen counter). If you read it from above, your eyes can fool you.

[Optional: Demonstrate measuring liquids and reading the amount at eye level while the cup is resting on a flat surface. Use any liquid. Water is fine.]

Measuring spoons are used to measure both solids (such as sugar) and liquids (such as salad dressing, oil, honey). Fill and level off.

Copyright 2017 University of Pittsburgh

Measuring/Estimating Meat and Cheese: You may either estimate the portion size or use a food scale.

- To estimate portion sizes of meat:
  - Three ounces of cooked meat is about the size of a deck of cards or your palm, minus the fingers.
  - A 3-4 ounce serving of a thin type of fish is about the size of a checkbook.

- If you use a food scale:
  - Buying a food scale is not required. But if you use a food scale, it is important to **weigh meats after they are cooked**. They lose about a quarter of their weight in cooking. So, 4 ounces of raw meat weighs about 3 ounces when it's cooked.
  - When you weigh cheese, you'll notice that one slice might look like another but not weigh the same. For prepackaged slices, you can check the label for the weight and nutrition information.

[Optional: Additional measuring handouts available in the GLB Provider Portal may be used, if appropriate for your group.]

We have talked about the importance of carefully measuring all foods and drinks. The next step is finding and recording the calories and grams of fat for each. This will give you a more accurate idea of the total number of calories you're taking in each day.

Keep Track of the Calories and Fat You Eat Every Day (page 4)

The best way to learn how many calories and fat grams are in food is to **keep track of the number of calories and amount of fat you eat every day.**

[You will need to adapt the following section depending on the participants' literacy level, willingness to self-monitor, and comprehension of the self-monitoring process.]

[Review information in the first text box. Include the following information:]

The first step is to:

1. **Record everything you eat and drink.** You may use a Food and Activity book or a digital tracking option of your choice.
   - You had an opportunity to practice this first step by measuring and recording everything you ate and drank last week.
   - As we mentioned last week, this is something we're going to do throughout the program. It is the **most important part of changing your eating behavior.**

Keeping track of what you eat will help you and me see, in black and white:

- What foods you eat
- How much you eat

Copyright 2017 University of Pittsburgh

- When and where you eat, and
- How your eating behaviors (habits, patterns) change over time

We discussed at the beginning of today's session how much you learned about yourself and your eating behaviors during your first week of self-monitoring. Each week you record, you will continue to become aware of your eating behaviors and see how you are making healthy changes.

Your self-monitoring records will be the very basis for our working together. Each week you will turn in the Food and Activity records you completed the night before. I will review it and write comments. I will return the Food and Activity record to you next week. If you want to use a digital tracking tool, please talk with me about how you will share your record with me.

Remember that only you and I will see them, so spelling is not important. You can make up abbreviations or use your own shorthand if that makes it easier and faster for you to keep track, just so we both know what you mean.

What is important is to:
- **Be honest**. Record what you really eat and drink, not just what you think will please me or yourself.
- **Be accurate.** It's best to record what you eat as soon as possible after you eat it, because it's easy to forget. Measure portions and read labels.
- **Be complete**. Include everything. Butter on toast, sugar and/or cream in coffee, and the mayonnaise on the sandwich.

It may seem hard to record all of your foods, especially at first. It does take some time. But it is worth its weight in gold. Being aware of what you are eating is the first step toward changing your eating behaviors. Remember that research shows that people who self-monitor lose more weight than those who don't.

You had practice this past week with this first step in recording. What's new this week is step two. *[Review information in the second text box. Include the following information:]*

2. **Figure out how many calories and fat grams are in every food and drink and record it.** To do this, you will need to:
   - Measure the amount of food and beverages you consume.
   - There are three places to look for the serving size, calorie and fat gram information.
     - Food labels have a Nutrition Facts label.
     - You could use a Calorie and Fat Counter or digital option. We'll talk more about these in a minute.
     - A third place to find calorie information is on menus, menu boards and displays. Federal regulations require that places that sell prepared foods and have 20 or more locations are required to post the calorie content of food on their menus, menu boards, and displays. This

Copyright 2017 University of Pittsburgh

applies to restaurants, supermarkets that sell prepared foods, convenience stores, bakeries, coffee shops, and movie theaters. Have you noticed calories on menus and menu boards? We will talk more about healthy eating away from home in a future lesson.

And the third step:

3. **Add up the calories and fat grams you eat and drink during the day.**
   [Review information in the third text box. Include the following information:]

   If you are using the GLB Keeping Track book, please transfer the total calories and fat grams to the back page at the end of each day.

Eat a Packaged Food? Read the Nutrition Facts Label (page 5)

One place you will find the calorie and fat information for foods and drinks is on the Nutrition Facts label on packaged food.

[The Nutrition Facts label pictured on page 5 shows the format the U.S. Food and Drug Administration requires manufacturers to use.]

Let's talk about the three important pieces of information to find on the label.
[Review page 5.]

Be sure the participants are able to locate the three most important pieces of information; the serving size, the calories per serving, and the total fat grams per serving. The most common error is using the "% Daily Value" from fat rather than the grams of "Total Fat".

Be sure that the participants understand that the serving size on a label may be very different from what most of us consider a serving. Also explain that many packages look like one serving but may contain more than one serving. Stress the importance of reading the label.

Use the calorie and fat gram information from the label if you can. Even if this item is listed in the Calorie and Fat Counter, the calories and fat grams on the label are more accurate.

The Nutrition Facts label includes additional useful information that we will discuss in upcoming group meetings.

When You Use the Calorie and Fat Counter (page 6)

Another place to find calorie and fat gram information is by using a Calorie and Fat Counter. There are many books and digital options available.

Copyright 2017 University of Pittsburgh

Page 6 lists some things to keep in mind **when you use the Calorie and Fat Gram Counter.** *[Review page 6. Include the following information:]*

If you make a **recipe,** there are websites available that will make it easy to calculate the calories and fat grams in your favorite recipes.

*[Optional: Distribute the handout, **Find the Calories and Fat Grams in a Recipe** for participants to use at home.]*

The bottom line for now is to **just get started and do your best**. If you run into any problems, I'll help you with them at the next session.

Know Where Your Calories and Fat Grams Come From (page 7)

Many people aren't aware of the calories and fat they eat.

Here's an example. *[Review the fast-food meal example.]* That is a lot of calories in one meal. It also has a lot of fat, a total of 22 ½ teaspoons or about 1 entire stick of butter.

What kinds of foods do you eat that are high in calories and fat?

Ask participants to share their answers. They may want to jot down their personal list of foods high in calories and fat.

Make some general points about the food groups or types of food that tend to be high in calories, such as:

- Meats (contain both fat that you can see and fat that you can't see)
- Dairy foods (whole milk, regular cheese, ice cream) ((**Many fat-free and low- fat flavored yogurts are high in calories because they contain a lot of sugar.)**
- Snacks (such as potato chips)
- Butter, margarine (Many people add fat to foods to flavor them.)
- Gravy, mayonnaise
- Baked goods (such as cookies, cake, muffins) (**Many fat-free and low fat baked goods are high in calories because they contain a lot of sugar.)**
- Fat added in cooking (oil, lard, shortening) such as deep-fat frying (fried chicken, French fries, doughnuts)
- Beverages (sweet tea, **soft drinks, sweetened coffee beverages, fruit drinks)**

Keep in mind that the purpose of this list is **not** to give the participants detailed information about where calories and fat are found in foods. Rather, the purpose is to begin to show them that many different foods that they eat are high calories and to provide a rationale for self-monitoring. The facts about where calories are found in foods should come later as a byproduct of their own discovery through self-monitoring.

Copyright 2017 University of Pittsburgh

These are the kinds of foods you will have to be aware of as you become a "calorie detective." They are also the kind of foods that are widely available and they may even be traditional foods in your family or culture. Once you know where the calories and fat in your diet come from, you can learn to make better choices.

Three Ways to Eat Fewer Calories (page 8)

There are no "forbidden foods" in GLB. To help you stay within your calorie and fat gram goals, while still including your favorite foods, let's talk about the three ways to eat fewer calories. [*Review the three ways and include the following information:]*

In the coming months, you'll discover a number of ways to eat fewer calories by using these three strategies.

Let's make a plan to eat fewer calories right now. *[Review Make a plan to eat fewer calories.]*

- Look at your list of high calorie foods on page 7.
- Select 3 foods and write them in the box on the bottom of page 8. Please select foods that you eat often (not, for example, birthday cake that you eat only rarely).
- Decide which of the three ways to eat fewer calories you are willing to use for each. Make sure it is specific, realistic, and something you will do.

For example: Sweet Tea (or any sugar-sweetened beverage). *Ask the group to provide a suggestion for each of the three ways to eat fewer calories.*

- Less often: Drink sweet tea only with dinner on Saturday and Sunday.
- Smaller amount: Measure and drink just 1 cup rather than unlimited amounts.
- Lower-calorie food: Try tea without sugar. Or drink water with lemon instead.

[Explain that there are many options so encourage participants to choose what they think will work best for them and to be open-minded as they experiment with ways to eat fewer calories.]

You will make your own food choices to reach your calorie and fat gram goals
There are no "good" or "bad" foods, just as there are no "forbidden foods" in GLB.
Using the three ways to eat fewer calories will help you make small changes that will make a big difference in the calories you are eating.

Through trial and error you will find what works best for you. Ask family members, friends, and group members for suggestions and ideas.

Changing the way we eat is a gradual process and it will take time. I don't expect you to be perfect. This program is about making progress toward your goals; not perfection.

Copyright 2017 University of Pittsburgh

Lifestyle change is an ongoing process. During the coming months you will learn many different ways to help you make healthier food choices and eat fewer calories. For now, focus on being the best calorie detective you can be, looking for calories everywhere. And just do your best to **come as close to your calorie and fat gram goals as you can.**

Practice Keeping Track (page 9)

Let's practice Keeping Track. If providing a Calorie and Fat Counter, distribute one to each participant at this time.

[Optional: Provide participants who are interested in using a digital self-monitoring tool with the ***Electronic Tools for Self-Monitoring*** *handout found in the GLB Providers Portal.]*

Either ask for a volunteer to report what s/he ate and drank for breakfast today or describe a simple breakfast meal. Write this menu on the board and ask participants to write it on **page 9).** In the first column, write down the time the meal was eaten. In the second column, record the amount and write down the item's name and a description. Skip the columns for calories and fat grams for now.

Give the participants time to record the foods. Taking one food at a time, ask the group to find each item in the Calorie and Fat Counter and to state the page number on which it is found. Make sure all participants can find the food and beverages in the Calorie and Fat Counter. Next, calculate the calories and fat grams in the amount eaten.

Address any areas of difficulty the group may have.

Some participants may find calculations difficult and confusing. Assure everyone that you will continue to help them with this in the upcoming sessions and that the **important thing for now is to begin finding foods in the Calorie and Fat Counter and getting an idea of the number of calories and amount of fat in different foods and in various serving sizes.**

If both high and low calorie foods with portion sizes that require some calculation were not included in the sample meal, give examples for the participants to find in the Calorie and Fat Counter and calculate calories and fat grams.

Ask the participants to add up the total calories and the total fat grams eaten during this meal. Write the calorie and fat gram totals on the practice page and label it "subtotal". Encourage participants to keep a running subtotal after each meal and snack.

Copyright 2017 University of Pittsburgh

A running total is like a subtotal or running balance in a checkbook. The purpose of keeping a running total is so you know just how many calories and fat grams you've eaten as you go through your day.

You can use this subtotal to plan what foods you choose for the rest of the day in order to stay on track with your goals. This is like using a budget to manage how much money you spend. For example, "What should I have for lunch? Well, I've eaten X calories and X grams of fat so far today. My daily calorie goal is X and my fat gram goal is X grams. So, I'd better eat less than X calories and X grams of fat for dinner to stay within my goals for the day." This is like using a budget to manage how much money you spend. Continue to add calories and fat grams after each meal and snack and record it.

You have the choice of using the Food and Activity books or using a digital tracking option. If you choose to use a digital food and activity tracking option, please talk with me so we can make a plan for how you will share your weekly record with me. [*Discuss your program's preferred tracking option(s).]*

Practice Keeping Track (page 10)

If using the GLB Keeping Track book, the final step of self-monitoring is to transfer the total calories and fat grams for each day to the back page.

[*Review page 10.]*

This will help us both to see at a glance how you've done during the entire week.

At the end of the week, you may want to add the daily calorie and fat totals to get a weekly total and divide it by seven to get your daily average calorie and fat intake. Looking at your weekly average calorie intake will help you see that one day of going a bit over your calorie goal will not ruin the entire week. But staying within your calorie goal only on Monday – Wednesday and then overeating on Thursday-Sunday will make it difficult to change your eating behaviors and lose weight.

Participants should leave this session aware that:

- We are more interested in their efforts to be honest and complete about their eating habits than to present us with picture-perfect self-monitoring records, and
- We consider self-monitoring a very important tool and expect everyone to self-monitor.

Copyright 2017 University of Pittsburgh

To Do (page 11)

Turn to page 11 and let's focus now on what you can do between now and the next session.

[*Review the first two check boxes.]*

As you did last week:

- Please continue to keep track of your weight and record it. This is a very important behavior for both losing weight and keeping it off.
- Please continue to keep track of what you eat and drink. Measure all foods and beverages and record them.

Notice the "New things to practice" section. These are designed to help you apply what you learned in today's group meeting. This will help you develop and practice skills you need to make positive changes in your eating behaviors. *[Review each item.]*

[*Announce that each week you will collect their completed Food and Activity record, review it and make comments and suggestions, and return it the following week. Explain that this is an important way for you to communicate with each person individually and for you to help them be successful in their efforts to create a healthier lifestyle.]*

[Encourage participants to talk with their health care provider about GLB and the changes they will be making in their eating, activity, and weight.]

[If your program requires health care provider approval to participate, explain your program's policy and procedures.]

[Announce the day, time, and place for the next session. Ask them to bring their GLB notebook to each session.]

After the session:

- Weigh participants who did not do so prior to the group meeting.
- Complete data forms and documentation required in your setting.
- Follow your program's protocol for managing absences.
- Review the self-monitoring records from the previous week. Write brief comments. Be positive and nonjudgmental. Comment on the mechanics of recording, not the nutritional value of their diet.
    - Praise all efforts to self-monitor, no matter how small, and any level of accuracy or completeness (e.g., descriptions of foods, methods of food preparation, additions to foods at table, or details about portion size).
    - Refer to **Guidelines for Reviewing Food and Activity Records** available in the GLB Providers Portal.

Copyright 2017 University of Pittsburgh

Session 3: Healthy Eating

Staying close to your calorie goal is key to losing weight. With practice, you will learn what foods, drinks, meals, and snacks work best *for you.*

Increasing physical activity will also help with losing weight. We will talk more about this in the next session.

First, let's talk about some important parts of healthy eating:

The way you eat

- When you eat
  - Plan ahead for healthy meals and snacks.
  - A regular pattern of meals or snacks is important. Some people do best with 3 meals per day; others with 3 meals plus 1 or 2 healthy snacks. Find what works best for you.
  - Eating something about every 3 to 5 hours will help you manage your hunger and blood glucose levels.

- How you eat
  - Eat slowly. This will help you:
    - Digest your food better.
    - Be more aware of **what you're eating** and enjoy the taste of your food.
    - Be more aware of **when you're full.** It takes about *20 minutes* for the brain to know that your stomach is full.
  - Pause between bites. Put down your fork or spoon.
  - Serve yourself smaller portions to begin with. Don't worry about cleaning your plate.

... and what you eat overall.

A model for planning healthy meals is MyPlate.

Copyright 2017 University of Pittsburgh

MyPlate

MyPlate was developed by the United States Department of Agriculture (USDA).

- It is a simple guide to building a healthy eating style.
- Helps you make a shift to healthier food and beverage choices.
- Based on science to promote health and prevent disease now and in the future.
- A healthy way to eat for the whole family.

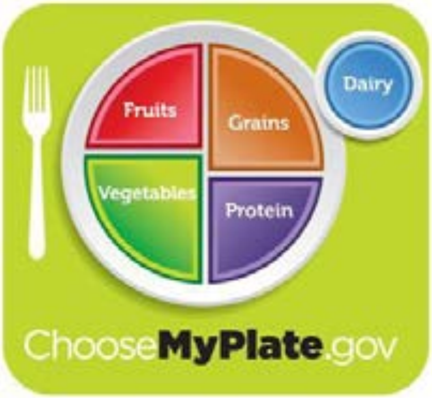

MyPlate shows you what foods to eat and in what amounts:

- Make about ½ your plate fruits and vegetables.
- Make about ¼ of your plate grains. At least half your grains should be whole grains.
- Make about ¼ of your plate protein foods.
- Include 3 servings of fat-free or low-fat dairy every day.

The 2015 Dietary Guidelines tell you to "follow a healthy eating pattern over time to help support a healthy body weight and reduce the risk of chronic disease". A healthy eating pattern:

- Includes all MyPlate groups; Fruit, Vegetables, Protein, Dairy, Grains, and Oils.
- Limits saturated fats and trans fat, added sugars, and sodium.

Go to www.ChooseMyPlate.gov for more information.

Copyright 2017 University of Pittsburgh

Healthy Food Choices from MyPlate

**Fruits**<br>(fresh, frozen, canned, or dried)	Focus on whole fruits.<br>• Choose fresh, canned, frozen, dried, 100% fruit juice.<br>• Eat whole fruit more often than fruit juice.<br>• Limit pastries, fruit drinks with added sugar, fruit canned in syrup.
**Vegetables**<br>(raw or cooked)	Vary your veggies.<br>• Choose fresh, frozen, canned, dried or 100% juice.<br>• Limit fried vegetables and those with added fat, cream, cheese, gravy, salt.
**Grains**<br>(bread, cereal, pasta, rice, tortillas, pita, muffins, oatmeal, barley, quinoa, couscous)	Make half your grains whole grains.<br>• Look for a "whole" grain as the first ingredient.<br>• Limit refined grain foods (those that are not whole).<br>• Limit those with added sugar and fat. Examples: white bread, pasta, and rice, pastries, donuts, muffins, biscuits, high-fat crackers, tortilla chips, fried tortillas, sweetened and granola type cereals.
**Protein Foods**<br>(meat, poultry, fish, legumes (beans/peas), eggs, nuts, seeds)	Vary your protein routine.<br>• Eat fish at least twice a week (*not fried*).<br>• Eat plant proteins often: tofu, soy products, cooked dried beans, rinsed/drained canned beans, split peas, lentils, hummus, and *small amounts* of nuts, seeds, peanut butter and other nut butters.<br>• Select lean cuts of meat. Trim or drain fat and remove poultry skin.<br>• Use low-fat cooking methods (bake, grill, steam, roast, microwave without added fat). Do not fry.<br>• If using processed meats, choose those with less fat, saturated fat, and sodium. Examples: sausage, bacon, hot dogs, ham, deli luncheon meats.
**Dairy**<br>(milk, yogurt, cheese, soymilk)	Move to low-fat and fat-free dairy.<br>• Most choices should be fat-free or low-fat milk, lactose-free milk, soymilk, yogurt, or cheese.<br>• Limit whole milk or cheese or yogurt, cream, cream cheese, sweetened yogurt/frozen yogurt/smoothies, ice cream.

Copyright 2017 University of Pittsburgh

10 tips
Nutrition
Education Series

MyPlate MyWins

Based on the
Dietary Guidelines
for Americans

Choose MyPlate

Use MyPlate to build your healthy eating style and maintain it for a lifetime. Choose foods and beverages from each MyPlate food group. Make sure your choices are limited in sodium, saturated fat, and added sugars. Start with small changes to make healthier choices you can enjoy.

1 Find your healthy eating style

Creating a healthy style means regularly eating a variety of foods to get the nutrients and calories you need. MyPlate's tips help you create your own healthy eating solutions—"MyWins."

2 Make half your plate fruits and vegetables

Eating colorful fruits and vegetables is important because they provide vitamins and minerals and most are low in calories.

3 Focus on whole fruits

Fruits

Choose whole fruits—fresh, frozen, dried, or canned in 100% juice. Enjoy fruit with meals, as snacks, or as a dessert.

4 Vary your veggies

Vegetables

Try adding fresh, frozen, or canned vegetables to salads, sides, and main dishes. Choose a variety of colorful vegetables prepared in healthful ways: steamed, sauteed, roasted, or raw.

5 Make half your grains whole grains

Grains

Look for whole grains listed first or second on the ingredients list—try oatmeal, popcorn, whole-grain bread, and brown rice. Limit grain-based desserts and snacks, such as cakes, cookies, and pastries.

6 Move to low-fat or fat-free milk or yogurt

Dairy

Choose low-fat or fat-free milk, yogurt, and soy beverages (soymilk) to cut back on saturated fat. Replace sour cream, cream, and regular cheese with low-fat yogurt, milk, and cheese.

7 Vary your protein routine

Protein

Mix up your protein foods to include seafood, beans and peas, unsalted nuts and seeds, soy products, eggs, and lean meats and poultry. Try main dishes made with beans or seafood like tuna salad or bean chili.

8 Drink and eat beverages and food with less sodium, saturated fat, and added sugars

Limit

Use the Nutrition Facts label and ingredients list to limit items high in sodium, saturated fat, and added sugars. Choose vegetable oils instead of butter, and oil-based sauces and dips instead of ones with butter, cream, or cheese.

9 Drink water instead of sugary drinks

Water is calorie-free. Non-diet soda, energy or sports drinks, and other sugar-sweetened drinks contain a lot of calories from added sugars and have few nutrients.

10 Everything you eat and drink matters

The right mix of foods can help you be healthier now and into the future. Turn small changes into your "MyPlate, MyWins."

Center for Nutrition Policy and Promotion
USDA is an equal opportunity provider, employer, and lender.

Go to ChooseMyPlate.gov
for more information.

DG TipSheet No. 1
June 2011
Revised October 2016

Choose Healthy Fats

Some fat in the diet is needed for health. MyPlate recommends that you replace unhealthy fats with *healthy* ones. This can help:

- Lower the levels of "bad" cholesterol and triglycerides in your blood. This lowers your risk of heart attack and stroke.

- Reduce your risk of type 2 diabetes and other health problems.

Important: Both healthy and unhealthy fats contain the same number of calories per gram. So always keep your fat gram goal in mind.

Unhealthy fats are solid at room temperature.

Limit:

- Saturated fat: found in animal foods, such as meat, meat drippings and high-fat dairy foods, and tropical oils, such as palm, palm kernel, and coconut oils.

- *Trans* fats: found in foods made with "hydrogenated" or "partially hydrogenated" oils (stick margarine, shortening), processed snack foods (crackers and chips), and baked goods (muffins, cakes, and cookies). Food companies are taking steps to remove trans fats from their products.

Healthy fats are liquid at room temperature. MyPlate calls them **"Oils."**

Choose:

- Monounsaturated and polyunsaturated fats: found mainly in non-tropical vegetable oils such as olive and canola, nuts, seeds, avocados, olives, peanut and other nut butters, mayonnaise, and fatty fish such as salmon, albacore tuna, herring, mackerel, and rainbow trout.

Copyright 2017 University of Pittsburgh

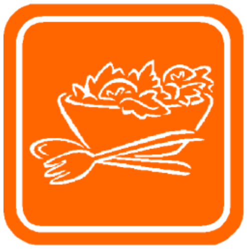

Healthy Eating for a Healthy Body

A healthy eating pattern and regular physical activity can improve health and reduce the risk of many diseases throughout life.

Experiment and find your own best ways to get there. Note that this means you will either "replace", "limit" or "include" certain types of foods.

To help keep blood glucose stable and hunger in check:

Build a regular pattern of healthy eating.

- Eat about the same amount of food at meals and snacks each day.
- Eat meals and snacks at about the same times every of day.
- Try not to skip meals and snacks.
- For healthy meals:
    - Include a variety of foods from all groups of MyPlate.
    - Include a food from the Protein and/or Dairy group at each meal. Protein will help you feel fuller longer.
    - Replace highly processed foods with those that are as close to nature as possible.

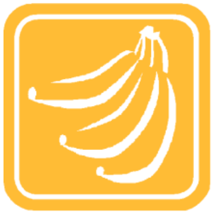

- For healthy snacks:
    - Limit "junk foods" such as chips and soda or a candy bar.
    - Replace with healthy snacks such as yogurt with a banana or an apple with a low-fat cheese stick or peanut butter.

A change I can make: ___________________________________

Choose healthier carbohydrates (sugar, starch, and fiber).

- Sugar:
    - Limit added sugars, high-sugar foods (candy, cakes, pies, cookies, and ice cream), and sugar-sweetened beverages (regular soda, fruit drinks, sweet tea, fancy coffee drinks).
    - Replace foods with added sugars with those that have natural sugar (fruit, vegetables, and milk).
    - Replace sugar-sweetened drinks. At least half of your daily fluid intake should be water.

Copyright 2017 University of Pittsburgh

- Starch:
    - o Limit sugar-sweetened cereals, white bread, rice, and pasta.
    - o Replace with whole grain bread and pasta, brown rice, legumes (beans and peas), unsweetened cereals high in fiber.

- Fiber:
    - o Include high fiber foods: legumes (beans and peas), nuts, seeds, whole grains, high fiber cereal, fruits and vegetables.
    - o Foods with 3 grams of fiber/serving are a good source of fiber; those with 5 grams or more are an excellent source.
    - o Drink water and increase fiber slowly to avoid constipation.

A change I can make: ___________________________________

To help reduce your risk of heart disease:

Replace unhealthy saturated and trans fats with healthier unsaturated fats. Include monounsaturated and polyunsaturated oils instead.

- Replace shortening or stick margarine with oil.
- Replace bacon or cheese in a salad with nuts or seeds.

Include foods with omega-3 fatty acids.

- Fatty fish, walnuts, ground flaxseed, chia seeds, flaxseed oil, and canola oil.
- The American Heart Association recommends:
    - o Eat fish (mainly fatty fish) at least two times per week. The serving size is 3.5 ounces cooked. Deep fried seafood is *not* recommended.
    - o Talk with your health care provider before taking omega-3 supplements.

Include high fiber foods.

- Fiber helps lower blood cholesterol and triglycerides.

A change I can make: ___________________________________

Copyright 2017 University of Pittsburgh

To help manage your blood pressure:

Limit your sodium intake. The 2015 Dietary Guidelines recommend you aim for less than 2300 milligrams (mg) per day.

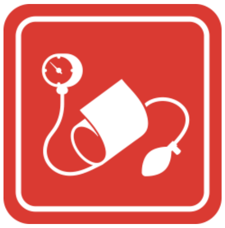

Limit the use of table salt.

- Don't add salt to your food at the table.
- Use half (or less) the amount of salt called for in a recipe.
- Replace salt with herbs, spices, peppers, lemon juice, or vinegar.

Limit the number of foods you eat that are high in sodium. Read food labels (you may be surprised at the sodium content).

- Seasonings like soy sauce and barbeque sauce.
- Salty snacks such as potato chips and crackers.
- Salty or smoked meats and fish, lunch meats.
- Foods prepared in brine such as pickles and sauerkraut.
- Processed foods such as frozen entrées canned or dried soups, baked goods, and pizza.
- Eat out less often. Limit fast food.

A change I can make: ____________________________________

To help maintain healthy bones:

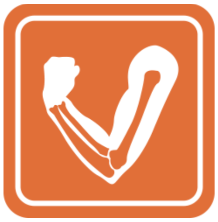

Include foods high in calcium:

- Low-fat or fat-free milk, soymilk, yogurt, or cheese.
- Salmon, broccoli, and leafy green vegetables.
- Fruit juices, bread, and cereal with added calcium.

Note: Talk to your health care provider before taking a calcium supplement.

A change I can make: ____________________________________

Don't let all the details overwhelm you.
You will find ways to make your own healthy eating pattern.
We are here to help.

Copyright 2017 University of Pittsburgh

MyPlate Makeover

Let's practice a meal makeover.

1. Write each item on the plate or cup below. If some of the items don't fit into any of the MyPlate groups, write them off to the side.

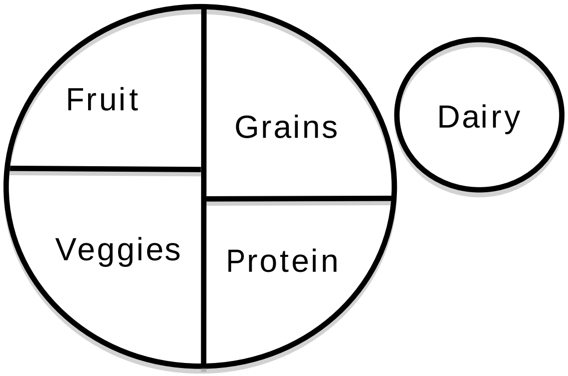

2. What changes could you make to this meal to better match MyPlate?

Vegetables ____

____

Fruits ____

____

Grains ____

____

Protein ____

____

Dairy ____

____

To eat fewer calories and limit unhealthy types of fat:

____

Copyright 2017 University of Pittsburgh

To Do:

Check the boxes when you complete each item:

☐ Keep track of your weight. Weigh yourself at home at least once a week. Record it.

☐ Record everything you eat and drink every day. Come as close as you can to your calorie and fat gram goals.

New Things to Practice:

☐ Follow MyPlate as a model for planning healthy meals and making better food choices. What changes did you make?

____

____

☐ "Replace", "limit", or "include" certain types of foods to meet your calorie and fat gram goals. What changes did you make?

____

____

☐ Be aware of your physical activities in the coming week and about how much time you spend doing them.

____

____

Copyright 2017 University of Pittsburgh

Session 3: Resources

	Page
Build a Better Recipe	12
Making Healthier Food Choices	13-14

Copyright 2017 University of Pittsburgh

Build a Better Recipe

Look for high-calorie foods in your recipes.
Use low-calorie foods instead.

Instead **of…**	**Use…**
Regular ground beef or pork sausage	Ground turkey breast (breast meat only, lean only, no skin), seafood, soy crumbles, tofu
Regular cheese	Low-fat or fat-free cheese (less than 2 grams of fat per ounce)
Sour cream	Low-fat or fat-free sour cream or plain, fat-free yogurt
Margarine, oil, or butter	Trans fat free tub margarine, vegetable oil spray
Chocolate	Cocoa powder plus a small amount of tub margarine (look on the back of the cocoa box for directions)
Pork or bacon fat to season foods	Small amount of trimmed pork loin chop, extra lean trimmed ham, turkey ham
Cream soup	Low-fat cream soups or flavored white sauce made without fat
Canned milk	Canned fat-free milk
Whole eggs	2 egg whites, egg substitute
Mayonnaise or salad dressing	Low-fat or fat-free mayonnaise or salad dressing, plain fat-free or low-fat yogurt
Whole milk or heavy cream	Low-fat or fat-free or canned fat-free milk

Tip: In recipes for cakes, cookies, muffins, and quick breads:

- Try cutting the amount of shortening, margarine, butter, or oil by ⅓ or ½.
- Replace the fat with the same amount of unsweetened applesauce, pureed prunes, or fat-free milk. It works!
- Keep in mind that these recipes may still be high in calories. Eat them less often and take small portions.

Copyright 2017 University of Pittsburgh

Making Healthier Food Choices

Instead of high-calorie foods, pick low-calorie foods.

Choose fresh fruit and vegetables for snacks.
Try vegetarian dinners several times a week.
Eat fruit for dessert.
Other:

Instead of high-calorie foods, use lower-calorie substitutes

Use low-fat:

- Soft (tub) margarine
- Frozen yogurt
- Salad dressing
- Cheese, cream cheeses
- Mayonnaise
- Sour cream

Fat-free or Low-fat milk

Other:

Instead of flavoring foods with fat, use low-fat flavorings.

To flavor these foods:	Use these low-fat flavorings:
Potatoes, vegetables	• Soft (tub) margarine (small amount), fat-free or low-fat sour cream, defatted broth, low-fat or fat-free plain yogurt, salsa.<br>• Herbs, mustard, lemon juice.
Bread	• Low-fat or fat-free cream cheese, soft (tub) margarine (small amount), all fruit jams.
Pancakes	• Fruit, unsweetened applesauce, crushed berries.
Salads	• Low-fat salad dressing, lemon juice, vinegar.<br>• Use nuts or seeds instead of bacon or cheese.
Pasta, rice	• Tomato based spaghetti sauce made with lean meat, poultry, seafood, or soy products, chopped vegetables, and no added fat.<br>• White sauce made with fat-free or low-fat milk.
Other:	

Copyright 2017 University of Pittsburgh

Ways to lower the calories, total fat, and saturated fat in meats.

Buy lean cuts (round, loin, sirloin, leg).
Trim all the fat you can see.
Use low-fat cooking methods:<br>• Bake, roast, broil, barbecue, stir-fry or grill instead of fry.<br>• Use a slow cooker without adding fat.
Remove skin from chicken and turkey. This can be done before or after cooking. Choose white meat.
Drain off fat after cooking. Blot with a paper towel. For ground beef, put in a colander or strainer after cooking and rinse with hot water.
Flavor meats with low-fat flavorings, such as BBQ, Tabasco, catsup, lemon juice or Worcestershire.
Other:

Avoid frying foods. Use other, healthier ways to cook.

Eggs:<br>• Poach, boil, or scramble eggs (or egg whites) with vegetable cooking spray.<br>• Use two egg whites instead of a whole egg.
Vegetables:<br>• Microwave, steam, stir-fry or boil vegetables in a small amount of water.<br>• Oven roast: Spray an oblong pan with cooking spray. Place cut vegetables (broccoli, cauliflower, onion, carrots, asparagus, Brussels sprouts, etc.) in pan. Drizzle with a small amount of olive or canola oil or use cooking spray. Season with pepper, garlic powder or other herbs/spices. Roast 450° for about 30 minutes, stirring occasionally, until brown.
Cook meats without adding fat (see ideas above).
Other:

Copyright 2017 University of Pittsburgh

Session 3 Leader Guide:
Healthy Eating

Objectives
In this session, the participants will:
- Discuss how eating fewer calories fits into the overall context of a healthy eating pattern.
- Explain MyPlate as a model of healthy eating, including an emphasis on plant foods and lower calorie choices.
- Review the recommendations of MyPlate and the 2015-2020 Dietary Guidelines for Americans.
- Practice comparing a meal to the recommendations of MyPlate.
- Discuss the types of fat and how to replace unhealthy fats with healthy ones while staying within the recommended calorie and fat gram goal.
- Discuss how and why to limit saturated fat, trans fat, sodium, and added sugars.
- Discuss factors to consider when planning healthy meals.
- Learn the importance of including vegetables, fruits, and whole grains in a healthy eating pattern.
- Find a personal pattern of meals and snacks.
- Discuss how following a healthy eating pattern can improve weight, health, and reduce the risk of chronic disease.
- Discuss ways to make a shift to healthier food choices.

To Do Before the Session:
- If providing Food and Activity books; one per participant.
- Review Food and Activity records from last session; add comments.
- Prepare Session 3 handout for participant notebook.
- (Optional): Have visuals to show graphically the fat content of common high fat foods. Relate to foods that you know the participants eat often. Useful visuals are teaspoons of fat on small plates or test tubes containing measured amounts of shortening.
- Make a plan for the MyPlate Makeover activity (Session handout, page 9). You can use the sample menu provided (Leader Guide, page 13) or create another example that includes foods typically eaten by your participants.
- (Optional): Poster or slide of MyPlate.
- (Optional): 9-inch plate for display.
- (Optional): MyPlate materials are available in Spanish and many other languages.

Additional resources:
- The Academy of Nutrition and Dietetics website also has information on ways to "Be Budget Friendly". http://www.eatright.org/resource/food/planning-and-prep/eat-right-on-a-budget/eat-right-affordably
- **Good and Cheap** cookbook, Leanne Brown. http://www.leannebrown.com/cookbooks

Copyright 2017 University of Pittsburgh

Available in the GLB Providers Portal:
https://www.diabetesprevention.pitt.edu/my/login.aspx

- **Guidelines for Reviewing Food and Activity Records**

General Session Procedures:

- Weigh each person privately. This may occur in the classroom, but scale display and participant weight record should not be visible to others.
- Record each participant's weight.
- Ask the participant to update his or her own **Weekly Record**. (Optional) Participants may also graph their weight.
- Make sure that the participant has selected the correct calorie and fat gram goal and written it on his/her **Weekly Record**.
- Write each participant's calorie and fat gram goals in the lifestyle coach's records.
- Maintain safety of participants as they step on and off the scale. Make sure weighing area is accessible and chairs are available for those needing extra assistance.
- If the participant has lost weight, express positive comments, but not excessively. Emphasize that s/he must already be making behavior changes.
- If the participant has not lost weight, be encouraging. Emphasize that little by little as s/he makes behavior changes, it will show up on the scale.
- Ask if the participant weighed himself or herself at home. Mention that home weights and weights taken at the session may differ because scales differ, but the trend in weight change over time should be the same.
- Collect completed Food and Activity records. If providing Food and Activity records, distribute blank ones and those with your comments.

You will continue with these general session procedures for the remainder of the program.

Copyright 2017 University of Pittsburgh

Group Sharing Time (allow at least 5 minutes)

In our last session, you learned how to "Be a Calorie Detective" by reading food labels, using a Calorie Counter, and looking at menus/menu boards and displays. You saw how using these tools would help you stay within your calorie and fat gram goals in order to lose weight. We also talked about **three ways to eat fewer calories** and you made a plan for the high calorie foods you eat.

Let's take a few minutes to talk about your experiences this past week. *[Choose only a few of the following prompts to guide discussion.]*

- Overall, how did it go last week? What went well? What problems did you have? What could you do differently?
- What did you learn by self-monitoring? What difficulties did you have?
- Were you surprised by the number of calories and amount of fat in some foods?
- How was your experience with using a Calorie and Fat Counter?
- Did you find the necessary information on the Nutrition Facts labels?
- Did you see nutrition information listed on any menu/menu boards in restaurants? Grocery stores? Other places that sell prepared foods?
- Did you keep a running total of calories and fat grams?
- Did you total your calories and fat grams for the week and find the daily average? What did you learn?
- Did you follow your plan to eat fewer calories?
- Did you try any new food products (e.g.: whole wheat pasta, brown rice)?
- How close did you come to your calorie and fat gram goal?
- What changes did you make in your eating in order to come as close as you can to your calorie goal? What change did you make in your eating pattern this past week that you are most proud of?

[Address and discuss barriers. Problem-solve barriers with the group.]

[Praise all efforts to self-monitor. Be positive and nonjudgmental. Encourage group discussion.]

Healthy Eating (page 1)

In the last session, we talked quite a bit about eating fewer calories. Staying close to your calorie goal is key to losing weight. Today we're going to talk about some of the *other* parts of healthy eating.

Copyright 2017 University of Pittsburgh

[Review page 1 and include the following information:]

Some of the other parts of healthy eating include:

The *way you eat*:

- **When you eat**
  - **Plan ahead for healthy meals and snacks**. Make a grocery list based on your plan and take it with you when you shop.
  - **A regular pattern of meals or snacks is important.** Spread your calories through the day in a pattern that fits your lifestyle and eating preferences. There is not one "right way" to divide calories through the day. Some people do best with 3 meals per day; others with 3 meals plus 1 or 2 healthy snacks. Find what works best for you. If you have been eating 3 meals per day without snacks but find you get overly hungry and then overeat at meals, consider adding a healthy snack. On the other hand, if you "graze" all day and lose track of the calories you're eating, consider eating more structured meals and snacks. Be open-minded and experiment to find what works best for you.
- **How you eat**
  - **Eat slowly.**
    - You will digest your food better if you take small bites and chew your food well.
    - It also can provide a sense of self-control.
    - Enjoy the company who may be present while you're eating.
  - D**on't worry about cleaning your plate.** The greatest waste of food is to eat more than you want or need.

Another part of healthy eating is *what you eat overall.*

- GLB emphasizes healthy food choices so you will lose weight ***and*** improve your health.
- One way to describe a healthy meal is in terms of food groups, in which foods are placed that have a similar amount and type of nutrients important to health. The United States Department of Agriculture (USDA) developed MyPlate as the model for healthy meals. MyPlate is a **general guide to healthy eating** that's based on the latest findings about nutrition and health.

Copyright 2017 University of Pittsburgh

MyPlate (page 2)

Have you heard of MyPlate? Have you seen this model pictured on page 2?

[Tailor the following discussion based on what the participants already know about MyPlate as you review the information on pages 2-4. The intent is to give participants a general overview of MyPlate, not an in-depth, detailed description. Participants interested in additional or more detailed information should be encouraged to visit the MyPlate website listed on the bottom of pages 2 and 4.]

Pages 2-4 of your handout give information about MyPlate.

Page 2 shows the design of MyPlate. *[Review the information on page 2. Include the following information:]*

Foods are placed into one of 5 food groups based on what nutrients they contain. These 5 groups are Vegetables, Fruits, Grains, Protein, and Dairy. *[Explain the format of the plate model, the different sections, and foods that belong in each.]*
Notice that half the plate should be filled with fruits and vegetables. If this would be done at each meal, people would easily reach the recommended 5-9 servings of produce each day.

MyPlate illustrates that you should be eating a mostly plant-based diet. A generation ago, many families built their meals around meat: the "meat and potatoes" eating style. Now we know that most Americans eat too much unhealthy saturated fat and much of it comes from big servings of meat. A healthy diet does include high protein foods. But the foundation of a healthy eating pattern is not meat, but rather vegetables, fruit, and grains (especially whole grains).

The 2015-2020 Dietary Guidelines recommend that we include foods from all groups of MyPlate to create a healthy eating pattern. The Guidelines also give recommendations about what to limit. Throughout GLB, we will continue to focus on sharing nutrition information and helping you create a healthy eating pattern that works for you. Something you can stick with for a lifetime.

The 2015 Dietary Guidelines focus on the entire diet as a whole rather than looking at a single nutrient or food. They report that *under-consumed* food groups include Vegetables, Fruits, Whole grains, and Dairy; *over-consumed* nutrients include saturated fat, sodium, refined grains, and added sugars.

The healthy eating pattern recommended in GLB is in line with the 2015 Dietary Guidelines.

Copyright 2017 University of Pittsburgh

[Participants with a 1200 or 1500 calorie goal may feel that trying to fit a food from each MyPlate group in one meal could be difficult. Explain the option of splitting the food into one meal + one snack. For example: Protein, Grain, and Vegetable (chicken, brown rice, green beans) for a meal and save Dairy and Fruit (vanilla yogurt + banana) for a snack.]

Healthy Food Choices from MyPlate (page 3)

Although there are no "forbidden foods" in GLB, page 3 gives more information about the foods in each group of MyPlate and suggestions for the healthiest choices. This page may give you some ideas of how to make some shifts to healthier food and beverage choices.

[Quickly review page 3. Again, the purpose is to give a general explanation of MyPlate. Participants who are interested in more information about foods in each group, portion sizes, etc. should be encouraged to visit the MyPlate website.]

[Participants may ask about plant-based beverages such as almond, cashew, or coconut milk. To help them determine if they wish to use these products, convey the following information; they are not considered as a serving from the Dairy group because they are much lower in protein than cow's milk or soymilk.]

Choose MyPlate (page 4)

Page 4 provides additional information about MyPlate. *[Encourage participants to read this page again at home. Point out the website address, www.choosemyplate.gov, as a source of additional information. Discuss the following information with your group:]*

Tip #2 reminds us to make half our plate fruits and vegetables. Eat a variety of different colored fruits and vegetables. The major health organizations recommend that we move toward a more plant-based diet. The goal is to eat about 5-9 servings of fruits/vegetables per day.

Tip #9 recommends drinking water instead of sugary drinks. This may be a way to cut calories that will work for you.

Another consideration is the size of the dinner plates you use at home. Plate sizes in the United States have increased over the years, as has the incidence of overweight and obesity. Measure the diameter of the dinner plates you use at home. Try using a 9-inch plate. This will help manage portion sizes while making it look like you are eating a satisfying amount of food. *[Optional: Show participants a 9-inch plate.]*

Copyright 2017 University of Pittsburgh

Participants may ask your advice about using sugar substitutes. All sugar substitutes have been approved by the Food and Drug Administration, are safe for human consumption, and are on the GRAS list (Generally Recognized as Safe), just as all other ingredients added to food in the United States

Research does not support the general recommendation to either consume or to avoid sugar substitutes. Thus, using foods and drinks that contain these sweeteners is a personal choice. Some people use them as a way to help stay within their calorie goals; others may choose to not include them.

GLB does not encourage nor discourage the use of sugar substitutes, but asks participants to decide for themselves if they want to use them or not.

Choose Healthy Fats (page 5)

MyPlate does not include a separate section for "Fat". The MyPlate website does have information on healthy types of fat to include in your healthy eating pattern in the section labeled "Oils".

Let's talk about **both** the **amount of fat** and **the type of fat** to include in a healthy eating pattern.

In Session 2 we discussed that one way to eat fewer calories is to eat less fat. Eating less fat overall can help stay within your calories goals so you lose weight. Remember that the goal of GLB is NOT to eat as little fat as possible. Some fat is needed for health. To include the correct **amount of fat** in a healthy eating pattern, stay within your fat gram goal.

In addition to being aware of the total **amount of fat** in your diet, it is important to use the **healthier types of fats** to improve your health. *[Review page 5 about the importance of and reasons for replacing unhealthy fats with healthy ones.]*

Please remember that all fats contain 9 calories per gram, so it is important to carefully watch portion sizes so you stay within your calorie and fat gram goals. We will continue to talk about making healthier food choices, including fats, throughout GLB. In fact, a future lesson is totally devoted to Heart Health. If you want more information, visit the "Oils" section on the MyPlate website.

[Note: Based on the latest scientific evidence, the 2015 Dietary Guidelines do not include the previous recommendation to limit cholesterol intake to less than 300mg/day. Many foods that contain cholesterol are also high in saturated fat, so limiting saturated fat may also limit cholesterol intake.]

Copyright 2017 University of Pittsburgh

[Participants may ask for a definition of "triglycerides". Explain that this is a type of fat found in the blood. Blood levels of triglycerides can be managed by exercising regularly, losing weight, and eating a diet that is low in unhealthy types of fat (saturated and trans fats), alcohol, and sugary foods and drinks.]

[In June 2015, the Food and Drug Administration gave food manufacturers three years (until 2018) to eliminate partially hydrogenated oils from their food products. This is the main source of trans fat in the American diet.]

Healthy Eating for a Healthy Body (pages 6-8)

The GLB goal is for you to improve both your weight and your health.

The first step continues to be to focus on ways to eat fewer calories in order to reach and maintain your weight loss goals.

The next step is to help you focus on making healthy food choices to improve your overall health. Creating a healthy eating pattern that you can follow for a lifetime will improve your health and reduce your risk of certain diseases. You may need to experiment to find the healthy eating pattern that works best for you. Again, there are no "forbidden foods", but you may need to **"replace"**, **"limit"** or **"eat more of"** certain types of foods.

Let's looks at all the ways that a healthy eating pattern, along with being physically active, can improve your health and reduce the risk of many diseases now and in the future.

[Review page 6. Include the following information:]

To help keep blood glucose stable and hunger in check:

Build a regular pattern of healthy eating:
As we discussed, you will find a healthy eating pattern that works best for you. This includes discovering if you want 3 meals per day or 3 meals with one or two healthy snacks. Try to eat at about the same times every day and avoid skipping meals and snacks. This usually causes a person to get overly hungry which can lead to overeating. Use MyPlate to plan healthy meals.

The GLB message is to **limit and replace highly processed foods** with those that are as close to nature as possible.

- An example of "eat as close to nature as possible" would be a baked potato vs. French Fries vs. potato chips. Another example would be an apple versus applesauce versus apple pie. Clearly the baked potato or apple are the best

choices because they are lower in calories, fat and sodium because they are very close to nature.

- **Note: Minimally** processed foods can be healthy and convenient. For example, bags of frozen vegetables, pre-washed salad greens, chicken that has pre-cut into boneless/skinless breasts, canned tuna.

Several times on pages 6-8 participants are asked to write down **"A change I can make".** These should be shifts to healthier food and beverage choices that the participant is willing to make. For example, trying whole grains, eating protein at each meal, shifting to healthier snacks, and drinking a beverage with no-added sugar.

As you review each section, ask participants to share their responses, if they are comfortable doing so.

GBL follows the recommendations from:

- The Institute of Medicine. It recommends 45-65% of total calories should come from carbohydrates in a healthy eating pattern.
- The 2015 Dietary Guidelines and MyPlate. Both recommend the intake of carbohydrates from vegetables, fruits, whole grains, dairy, and legumes.

Some participants may think that "all carbs are bad".

The GLB messages are that we look at the entire diet as a whole, no food is "demonized" (not even sugar), there are no forbidden foods, and our emphasis is on choosing healthier carbohydrates.

Choose healthier carbohydrates (pages 6 & 7):

A healthy diet includes carbohydrate.

- Just as you have been encouraged to choose healthier types of fat, there are healthier carbohydrates you should choose to include in your healthy eating pattern.
- Healthy carbohydrates come from fruits, vegetables, whole grains, beans and peas, and low-fat or fat-free dairy foods. These foods provide many healthful nutrients your body needs.

You are not being asked to monitor your carbohydrate intake, just calories and grams of fat, which was done in DPP.

Copyright 2017 University of Pittsburgh

The 2015 Dietary Guidelines recommend that we consume less than 10% of calories per day from added sugar. This does NOT include sugar that is naturally present in fruit, vegetables, and milk.

- The reason is that foods and beverages with added sugars contribute calories, but no healthful nutrients. Evidence suggests that eating patterns with less added sugars are associated with a reduced risk of cardiovascular disease, obesity, type 2 diabetes, and some types of cancers in adults.
- This does NOT mean that every food must contain less than 10% of calories from added sugars; look at the entire day's intake of added sugars. The suggestions listed here will help you reduce the added sugars in your meals and snacks.

Fiber: The amount of fiber in a food is listed on the Nutrition Facts label in both grams of fiber and %Daily Value. The %Daily Value is based on 28 grams of fiber per day.

Note that at least half of your daily fluid intake should be water. There is no set guideline for how much water people should drink each day. How many of you have heard that we're all supposed to drink 8 cups of fluid per day? That isn't supported by research. Fluid requirements vary among individuals based on age, sex, activity level, medications, environment (temperature/humidity), and what you're eating that day. Instead, the goal is to drink enough water so you have light colored urine. Urine that is very dark yellow can be a sign that you are dehydrated. People often experience hunger when dehydrated and eat even though their body is really just lacking fluid, not food. Staying well-hydrated may help you stay within your calorie goals.

To help reduce your risk of heart disease:

[Review page 7. Include the following:]

Healthy unsaturated oils should replace solid fats.

[Participants may ask about using coconut oil. The current recommendation, based on the available scientific evidence, is that coconut oil is considered a solid fat (high in saturated fat) and should be limited. There is ongoing research to determine if the saturated fat found in plant foods (e.g. coconut oil) has the same negative effects as solid fats from animal foods.]

A healthy eating pattern limits saturated and trans fat. The 2015 Dietary Guidelines recommends that less than 10% of daily calories should come from saturated fat. This recommendation is based on scientific evidence that replacing saturated fat with unsaturated fat is linked to a reduced risk of cardiovascular disease.

Copyright 2017 University of Pittsburgh

Participants may have questions about Omega 3 fatty acids. Explain that they are a type of unsaturated fat that is found mostly in fish and other sources listed on page 5.

Emphasize that fish should be prepared using low-fat cooking methods, not deep-fried.

Be sure to remind participants that they should consult their health care provider before beginning any kind of supplement.

We will be discussing fats again in future lessons. Participants who are interested in learning more about omega-3 fatty acids may access the American Heart Association website.

[Review page 8. Include the following:]

To help manage your blood pressure:

Most Americans get 50% more sodium than recommended.

As sodium intake goes up, so does blood pressure. This relationship between sodium intake and blood pressure is well documented. Diets high in sodium are also associated with heart disease.

[Participants may have already been told by their health care providers to reduce sodium intake due to prehypertension or hypertension.]

We have covered a lot of information today about healthy eating. Don't let all the details overwhelm you. Throughout GLB, we will continue to talk about healthy eating and how to create a healthy eating pattern than works for you.

The goal of GLB is to eat fewer calories, but healthier calories as well. Keep making shifts to healthier food and beverage choices. These small changes will lead to improvements in your weight and health. Focus on progress, not perfection.

Copyright 2017 University of Pittsburgh

Some participants express concern that healthy eating is too expensive. Acknowledge any concerns participants may raise. Use the opportunity to problem-solve with the group. The following points may help address concerns:

- Grains: Sugared cereals are usually more expensive than unsweetened. Reduced fat crackers are typically the same price as the full-fat crackers. Bagels are usually cheaper than donuts. You pay more for convenience. For example, instant oatmeal packaged in individual servings is about twice the price per ounce than buying a box of quick oats that you measure yourself.
- Vegetables: Buy fresh produce in season. Take advantage of local farmers markets. Use canned and frozen vegetables without sauces or cheese. Buying organic produce usually increases the cost. Buying prewashed or precut vegetables adds to the cost. Consider growing a garden!
- Fruit: Buy fresh produce in season. Take advantage of local farmers markets. Use canned or frozen fruit without added sugar or packed in its own juice. Watch the packaging: frozen orange juice that requires reconstituting with water is less expensive per ounce than the cartons that are ready to drink. Juice packaged in a single serving container is more expensive, yet.
- Dairy: Whole milk is usually more expensive than skim milk. Low fat or fat free cottage cheese is the same price as the full-fat.
- Protein: Leaner cuts of meat can be more expensive than the higher fat cuts. Remember that you will probably be eating a smaller portion size than you did before you started the program. Consider adding some meatless meals. Legumes (beans and peas) are an inexpensive source of protein and fiber.
- Remind participants that they will be eating less food overall than they did before they started the program.
- They will be saving money by buying less (or none) of the expensive "junk foods" like candy, chips, etc.
- To save money, plan meals ahead of time, make a shopping list, watch the ads for sales, clip coupons for healthy foods, and try store brands.
    - A cookbook designed for cooking on a limited income (including the SNAP budget of about $4 per day) is **Good and Cheap** by Leanne Brown. The recipes are healthy and very simple. http://www.leannebrown.com/cookbooks
    - The Academy of Nutrition and Dietetics website also has information on ways to "Be Budget Friendly".

http://www.eatright.org/resource/food/planning-and-prep/eat-right-on-a-budget/eat-right-affordably

Copyright 2017 University of Pittsburgh

MyPlate Makeover (page 9)

Let's practice using MyPlate. Turn to page 9.

Write a sample dinner menu on the board. It is suggested that the menu include a very large serving of meat, small serving of vegetable, and dessert without a serving from Fruit or Dairy groups.

Example: 8 ounce steak, 1 cup rice, ½ cup green beans, 8 ounce glass of wine, chocolate cake.

Or come up with a menu that includes foods typically eaten by your participants.

Draw MyPlate on the board or flipchart. Encourage group participation during this activity.

Look at the first item on the menu. In which food group of MyPlate does this food belong? *[Write the name of the food in the correct section of the plate.]*

Let's look at the next item on the menu. In which food group does this belong? *[Write the name of the food in the correct section of the plate. Repeat for each food and beverage on the menu.]*

Now let's do a "Meal makeover" by comparing this meal with the recommendations of MyPlate.

- Is there a food in each section of the plate?
- Is there a food high in protein? Is the Protein potion size about 3 ounces? Is it a low-fat protein choice?
- Is the food from the Grain group a whole grain?
- Are fruits/vegetables included?
- Are there a variety of colors of fruits and vegetables?
- Do fruits and vegetables make up half the plate?
- Is the Dairy item low-fat or fat-free?
- How were the foods prepared? (Discuss low-fat versus high-fat cooking methods.)
- Overall, are the food choices low or high in calories?

[If your menu includes alcohol, explain that if you choose to drink alcohol, do so in moderation. Moderation is defined as no more than 1 drink per day for women and no more than 2 drinks per day for men. Consider one serving to be one can (12 fluid ounces) of beer (150 calories), one glass (5 fluid ounces) of dry wine (115 calories), or 1.5 fluid ounces (one "shot") of liquor (105 calories). Alcoholic beverages are high in calories. Mixers, such as tonic or a regular soft drink, add more calories.]

Copyright 2017 University of Pittsburgh

What changes could be made to this meal to better follow the recommendations of MyPlate? (For example: more vegetables, smaller portion of meat, add fruit and/or dairy, etc.)

The 2015 Dietary Guidelines recommend eating less added sugar, salt, and saturated fat. Does this meal follow the recommendations? What could be done to eat less total fat and limit unhealthy fat? Less salt? Less added sugars?

[Emphasize the importance of reading Nutrition Fact labels, menus and menu boards and using the Calorie Counter. Discuss if this meal would be easy or difficult to fit into the calorie and fat gram goals for the day.]

For weight loss, it is important to stay within your calorie and fat gram goals.
To improve your health while you are losing weight, it is important to make the healthiest food choices possible within your calorie and fat gram goals.

MyPlate will help you to include many healthful nutrients in your meals while staying within your goals. MyPlate will help you eat healthy, balanced meals, include more plant foods, and practice portion control.

Choose a healthy eating style that meets your nutrition and calorie goals.

Healthy eating is important. Don't forget about physical activity. Regular physical activity is one of the most important things you can do to improve your health. We will talk more about physical activity in our next group meeting.

To Do (page 10)

Turn to page 10 and let's focus now on what you can do between now and the next session.

[*Review the first two check boxes.]*

As you did last week:

- Please continue to keep track of your weight and record it. This is a very important behavior for both losing weight and keeping it off.
- Please continue to measure and record everything you eat and drink every day. Self-monitoring is also a very important behavior.

Notice the "New things to practice" section. These are designed to help you apply what you learned in today's group meeting. This will help you develop and practice skills you need to make positive changes in your eating behaviors. *[Review each item.]*

Use MyPlate as a model for healthy meals. Try it at least once this week.

Copyright 2017 University of Pittsburgh

Next week we'll begin to talk in detail about the GLB activity goal. For this coming week, please be aware of your physical activity. Write down the activities you do.

[Encourage participants to talk with their health care provider about GLB and the changes they will be making in their eating, activity, and weight.]

[If your program requires health care provider approval to participate, explain your program's policy and procedures.]

[*Announce that each week you will collect their completed Food and Activity records, review it and make comments and suggestions, and return it the following week. Explain that this is an important way for you to communicate with each person individually and for you to help them be successful in their efforts to create a healthier lifestyle. Encourage participant to save for future reference the Food and Activity records that you return to them.]*

Resources (pages 11-14)

The two extra handouts provide a lot of useful information. I encourage you to read them at home and to try some of the suggestions. You can give us your feedback at the next group meeting.

Page 12: **Build a Better Recipe**. This is for those of you who do a great deal of cooking from recipes. It has some helpful information to help you make recipes lower in calories.

Pages 13-14: **Making Healthier Food Choices**. These pages give additional suggestions for making healthier food choices. Let me point out two sections that might be particularly useful:

- On page 14, the fifth box in "Ways to lower the calories, total fat, and saturated fat in meats" gives directions for how to rinse cooked ground beef. Have any of you ever tried this? Most of us are used to cooking ground beef and draining off the fat, but rinsing it removes even more fat. Usually we add the cooked ground beef to things like spaghetti sauce, chili, tacos, etc., so you won't notice any change in flavor.
- In the box, "Avoid frying foods" there is an easy recipe for how to oven roast vegetables. Have any of you tried this?

[Announce the day, time, and place for the next session. Ask them to bring their GLB notebook to each session.]

Copyright 2017 University of Pittsburgh

After the session:

- Weigh participants who did not do so prior to the group meeting.
- Complete data forms and documentation required in your setting.
- Follow your program's protocol for managing absences.
- Review the self-monitoring records from the previous week. Write brief comments. Be positive and nonjudgmental.
    - Praise all efforts to self-monitor, no matter how small, and any level of accuracy or completeness (e.g., descriptions of foods, methods of food preparation, additions to foods at table, or details about portion size).
    - Highlight especially any positive changes made that relate to the session topic of the week before the records were collected: recording calorie and fat gram information, getting close to the recommended calorie and fat gram goals, and use of the "Three Ways to Eat Fewer Calories". Praise all efforts to change eating behaviors.
    - Refer to **Guidelines for Reviewing Food and Activity Records** available in the GLB Providers Portal.

Session 4: Move Those Muscles

It's all about *<u>moving more</u>*.

Progress has led to less overall daily movement. Many people now do their day-to-day tasks with little physical effort.

- The good news: You get more done in a given amount of time.
- The bad news: The need to move is being removed from your daily routine.

What tasks do you do now with less movement than years ago?

Moving more is one of two main goals of GLB. It is key to your overall physical health, well-being and independence. An active lifestyle can also help you manage your weight. To add movement back into your daily routine, ***focus on the two important types of activity***.

1. **Planned activity:**
   - Moderate intensity <u>physical activity (similar to a brisk walk) that you add to your schedule</u>
   - The eventual goal is to reach at least **150 minutes of moderate intensity activity/week.** Example: 30 minutes of brisk walking on 5 days per week.
   - Bouts of activity should last at least 10 minutes at a time
   - Build up at a pace that is safe and do-able
2. **Spontaneous or unstructured activity:**
   - Activity <u>choices you make throughout the day</u>
   - Hard to keep track of easily but makes a big difference in your physical activity level at the end of the day
   - Often these activities are of light intensity (such as housework or gardening)
   - Research suggests that replacing your sitting time with physical movement is <u>important for your overall health</u>

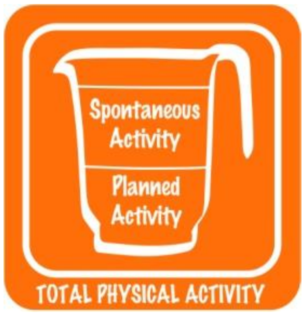

It is ***the sum of both planned and spontaneous activity*** that makes up your total physical activity level.

Copyright 2017 University of Pittsburgh

Think back. Were there times when you were more physically active? What sort of activities did you do? What worked best for you?

____

____

____

Most people begin this program with their own thoughts and feelings about what it is like to be active or inactive (see common examples below). *What are yours?*

	What's good about it	What's hard about it
**Being Inactive**	*"I like to lie on the couch and watch TV"*<br>____________________<br>____________________	*"I feel stiff and sluggish"*<br>____________________<br>____________________
**Being Active**	*"I feel strong and healthy"*<br>____________________<br>____________________	*"I am really busy"*<br>____________________<br>____________________

Copyright 2017 University of Pittsburgh

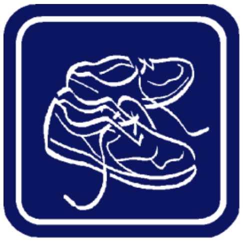

What a Bargain: The Many Benefits of an Active Lifestyle

Physical activity is one of the biggest “bargains” you can get. It has many rewards. Here are just a few:

Being more active may:

- Help you have more energy, feel better and sleep better.
- Make you more physically fit. Being more fit will make it easier for you to do your daily tasks (like climbing stairs, keeping up with your children, grandchildren or friends).
- Improve your bone density and muscle strength. This leads to better joint health, flexibility, and balance.
- Lower your risk for diabetes, heart disease and certain types of cancer.
- Improve your mental alertness and brain-function. This can lower risk of dementia and Alzheimer’s disease.
- Help you lose weight and keep it off.

Being more active may:

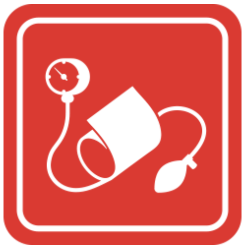

- Raise your HDL (“good”) cholesterol.
- Lower your triglycerides.
- Lower your blood pressure.
- Lower your blood glucose by making your body more sensitive to insulin.

All of these physical activity benefits can lead to better overall physical function and more independence as you age.

What are the main reasons ***you*** want to have a more active lifestyle?

____

The DPP showed that being active was an important part of reducing the risk of developing type 2 diabetes.

It also improved other risk factors for heart disease.

Imagine having a chance to prevent or delay such serious conditions.

Copyright 2017 University of Pittsburgh

What You Can Expect from the GLB Physical Activity Program

Rest assured that **you can do it.** There are many reasons the physical activity part of GLB is likely to work for you.

- **Realistic starting point:** Begin where you are right now. No judgment, no questions asked. Add small amounts of activity over time.

- **Choose your own types of activities:** Most people choose brisk walking as their main form of activity. It's your choice.

- **Keep track:** Being aware of your activity habits is important. It's key to changing your activity behavior. It also lets you see your progress over time.

- **Plan ahead:** A physically active lifestyle does not happen by chance. Look at your typical week and schedule blocks of time that are most likely to work for you.

- **Progression:** Should be slow and safe. Increase around 30 minutes per week (less than 5 minutes per day). For safety reasons, we recommend that you increase <u>duration</u> of activity (total time) first, not intensity (how hard you are working). Any increase in either duration or intensity should be gradual.

- **Safety:** This program is all about safety. Let your health care provider know that you will be making changes in your eating, activity, and weight. You may be advised to limit or avoid certain types of activity.

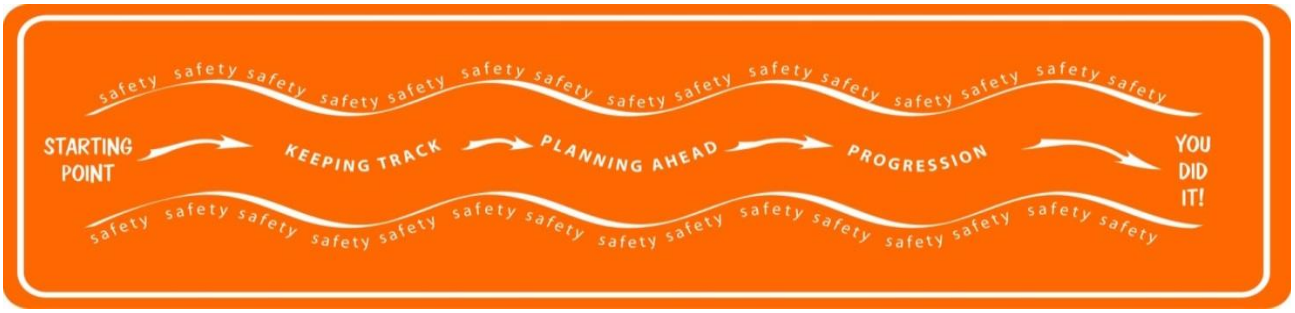

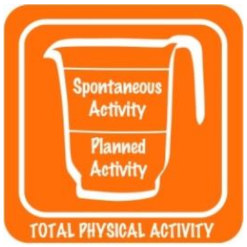

Your Physical Activity Goal: Planned Activity

Aim for at least 2½ hours (that's 150 minutes) of physical activity each week.

This is the "planned" part of your total activity goal. It is safe, gradual, and do-able. It is also in line with many national public health recommendations as well as the DPP itself.

- Choose forms of physical activity that are of **moderate intensity**, like brisk walking.
- Aim to do at least 10 minutes at a time.
- Work up to this goal *slowly*.
- Spread the activity over 3 or more days per week.
- Pick activities you LIKE.

You have thought about activities you used to do and what worked best. What will you do to get started with your activity progression now? Many people choose walking and that is great. List below some of the things that you think will help you put your best foot forward.

____

____

____

____

Copyright 2017 University of Pittsburgh

Physical Activity Progression: Where should I start?

How many total minutes did you spend doing moderate intensity physical activity this past week?

- If **less than 60 minutes**, aim for 60 minutes this week.
- If **60 minutes or more**, aim for the same amount this week.
- Track your activity minutes this week.

How to progress: Gradually increase the amount of physical activity you do. A good rule of thumb is to increase by 30 minutes each week. For example:

Week 4:	Walk 60 minutes per week.<br>(12 minutes on 5 days per week)
Week 5:	Walk 90 minutes per week.<br>(18 minutes on 5 days per week)
Week 6:	Walk 120 minutes per week.<br>(24 minutes on 5 days per week)
Week 7:	Walk 150 minutes per week.<br>(30 minutes on 5 days per week)
Week 8+:	Walk at least 150 minutes per week.

Types of Aerobic Activities

Aerobic physical activities when done at a moderate intensity work your heart and lungs. They elevate your heart rate and keep it up for the duration of the activity. Most people choose to do **brisk walking** because it is relatively easy to do and good for you.

- Aerobic dance (step aerobics)
- Bicycle riding (outdoors, or indoor stationery bike)
- Dancing (square dancing, line dancing)
- Hiking
- Rowing (canoeing, rowing machine)
- Skating (ice skating, roller skating, rollerblading)
- Skiing (cross-country, NordicTrack)
- Elliptical, StairMaster®
- Swimming (laps, snorkeling)
- Tennis
- Volleyball
- Walking (treadmill, outdoor, indoor mall or fitness center)
- Water Aerobics

Note: Resistance training is not considered aerobic but is highly recommended; more on this later.

Copyright 2017 University of Pittsburgh

Plan for an Active Week

"A goal without a plan is just a wish"
-Antoine de Saint-Exupery, French writer

Last week we asked you to be aware of the physical activities that you were doing. Now you will **make a plan** for a more active week and keep track of your physical activity minutes.

<u>Planned Activity</u>: You *can* find the time to be active:

- Set aside blocks of time this week to be active.
- Record bouts of activity that last 10 minutes or longer and are of "moderate" intensity (like a brisk walk).

	**What will you do?**	**When?**	**Where?**	**Minutes**
Monday				
Tuesday				
Wednesday				
Thursday				
Friday				
Saturday				
Sunday				
Total planned physical activity this week				

Don't just wait for physical activity to happen.

Make it happen by ***devoting blocks of time*** to be active. Look ahead each week, plan dates with yourself and others, and mark it on your calendar. Try not to schedule others things during those times.

Once you complete your activity, record your minutes and <u>give yourself credit</u>.

Copyright 2017 University of Pittsburgh

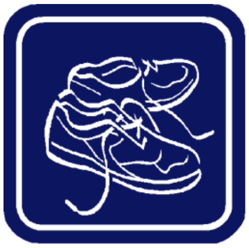

The Smart Way to Be Active: Keep It Safe

Being active is usually quite safe. However, in rare cases, problems can arise. *Prevention* is the best approach. **Here are 5 key ways to be sure that you can keep it safe when being active.**

1. WARM-UP and COOL-DOWN

Warm-up

- Slowly increases blood flow and prepares your body for exercise.
- This is even more important if you have high blood pressure or a history of heart conditions.
- Do whatever activity you plan to do but at a lower intensity.
- Take about 5 to 10 minutes to warm up. For example, walk at a slow pace before picking up to a brisk pace.

Cool-down

- Helps keep your heart rate and blood pressure from dropping too quickly. If you stop abruptly, you may feel light-headed.
- Do whatever activity you have been doing, but at a lower intensity (go back to a slow walking pace).
- Take about 5 to 10 minutes to cool-down.
- Once you cool down, you may choose to do a few minutes of mild stretching.

2. KEEP IN CONTROL

- Stay in the driver's seat and don't let gravity be in control.
- Jerky unstable movements (like "bouncing") can lead to injury.
- When doing stretches, or any activity, use your body (or stable object like a chair) to help support yourself and keep in control.
- See page 14, exercise 2 for an example of a supported side bend.

Copyright 2017 University of Pittsburgh

3. KEEP BALANCED

- Always work BOTH sides of your body equally.
- When you stretch or strengthen on one side, don't forget to do repeat on the other side.
- If you walk laps on a small track, be sure to change direction.

4. BREATHE

- Keep breathing while exerting yourself during physical activity. (Swimming underwater is the exception to the rule).
- Holding your breath during exercise can increase blood pressure.
- Count out loud if that helps.
- Pace yourself.

5. STRETCH

- Stretching may prevent muscle soreness, cramps, and injuries.
- Stretching may also help you become more flexible and feel relaxed.
- WARM-UP before stretching (Example: gentle walking).
- Move slowly until you feel the stretch.
- Hold for 15 to 30 seconds.
- See stretching exercises on pages 13-16.

Important Message from Your Coach:

NO PAIN *<u>IS</u>* GAIN. *Always listen to your body.*

- Make small, gradual increases in how often, hard, and long you are physically active.
- You may feel sore when you start or increase an activity. Some soreness is normal, but it should ease up over time.
- Physical activity ***should not be painful.*** If a certain activity is quite painful—STOP. If pain persists, seek medical advice.

Copyright 2017 University of Pittsburgh

When to stop exercising…

Being more physically active is usually quite safe. But in rare cases, problems can arise. **The following may be signs of something serious like a heart problem, so should NEVER be ignored.**

Chest pain or discomfort:

What: Uncomfortable feeling of pressure, pain, squeezing, or heaviness. Especially if it comes on with increased effort or when trying a new activity.

Where: Possibly in the:

- Center of the chest,
- Spread throughout the front of the chest, or
- Radiating to, or even starting, in the shoulder(s), arm(s), neck, jaw, and back.

What to do: Stop and sit or lie down.

If it doesn't go away after 2-4 minutes, call 9-1-1.

If it does go away, let your health care provider know this happened.

Severe nausea, shortness of breath, cold sweats, feeling lightheaded or irregular pulse/palpitations:

What to do: Stop and sit or lie down.

If it doesn't go away in 5-10 minutes, call your health care provider.

If it does go away, let your health care provider know this happened.

We want you to stay safe while you exercise. Following the guidelines above will lessen the risk of exercise-related injury.

Copyright 2017 University of Pittsburgh

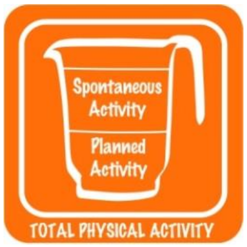

Your Physical Activity Goal: Spontaneous/Unstructured Activity

Every day you have many chances to make "the more active choice". Every time you move it adds up to a "more active you".

1. **Start by becoming more aware** of these opportunities.
    - These activity moments will usually be brief and of light intensity.
    - They will often be spontaneous and last a few minutes.
    - These minutes will add up and make a big difference in your total physical activity level.
    - Experts suggest you pay closer attention to this "light intensity activity". It makes up the largest portion of your total physical movement each day.

2. **Increase your spontaneous activity**. When you have the chance, <u>choose to move</u>.
    - Walk instead of driving when possible (called "active transport").
    - Get off the bus a few stops sooner and walk.
    - Take the stairs instead of the elevator, even a few flights.
    - Be active while you watch TV. Get up and move during commercial breaks.

3. **Decrease the time you spend sitting**. Be aware of how much time you sit (at work, at home, during social activities).
    - Cut down on your TV and screen time.
    - **Break up long bouts of sitting**.

By replacing sitting time with movement of any intensity, you will increase your overall physical activity level. This behavior change may benefit your health.

Name some ways **you** could make an active choice or replace your sitting time. Study your routine. Find strategies that will *work for you*.

____

____

Copyright 2017 University of Pittsburgh

To Do:

Check the boxes when you complete each item:

☐ **Keep track of your weight.** Weigh yourself at home at least once a week. Record it.

☐ **Record everything you eat and drink every day.** Come as close as you can to your calorie and fat gram goals.

New things to practice:

☐ **Be active for ____ minutes this week.**

- How many minutes were you active last week? _______________
  - If **less than 60 minutes**, your goal for this week is 60 minutes.
  - If **60 minutes or more**, aim for the same amount this week.

☐ **Follow your Activity Plan**, see page 8.

- Warm up, cool down, and follow the guidelines for safe stretching.

☐ **Keep track of your minutes of physical activity.**

- Record only the time when you're **doing** physical activity.
- Only record activities that last 10 minutes or more.
- Begin to fill in the "Year-at-a-Glance" chart on page 21 (optional).

☐ **Make active choices throughout the day.**

- List some of the ways you increased your spontaneous physical activity.

____

____

Copyright 2017 University of Pittsburgh

Session 4: Resources Page

Stretches and Warm-up Activities	14-17
Footwear information: Wear a Good Pair of Shoes	18
In Case of Injury	19
Beat the Heat	20
Keep Warm in the Cold	21
Tracking Activity: Year-at-a-Glance	22-23

References:

United States Department of Health & Human Services. Physical activity and Health: a Report of the Surgeon General. (1996): DIANE Publishing.

Haskell, William L., I-Min Lee, Russell R. Pate, et al. "Physical activity and public health: updated recommendation for adults from the American College of Sports Medicine and the American Heart Association." *Circulation* 116; 9 (2007): 1081.

Centers for Disease Control and Prevention, Department of Health and Human Services, Physical Activity Guidelines for Americans (2008): www.health.gov/paguidelines

American Heart Association: Warning Signs of a Heart Attack: http://www.heart.org/HEARTORG/Conditions/HeartAttack/WarningSignsofaHeartAttack/Warning-Signs-of-a-Heart-Attack_UCM_002039_Article.jsp

Copyright 2017 University of Pittsburgh

Stretches

Stretching may prevent muscle soreness, cramps and injuries. It may help you become more flexible and feel relaxed. REMEMBER:

- Always WARM-UP before stretching (such as gentle walking).
- Stretching should not feel painful. Move slowly until you feel the stretch.
- Hold the stretch for 15 to 30 seconds.
- Breathe.

Here is a stretching program for you to try. Some stretches show two options. The second image shows a more advanced option for each specific muscle group. Be sure to listen to your body and choose the stretches that work best for you.

1a. Corner Chest Stretch

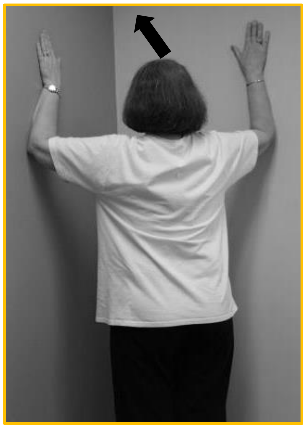

- Put your hands up in a corner, keep your shoulders relaxed.
- Gently step forward until you feel a stretch in your chest.
- Hold for 15 to 30 seconds. Repeat 3 to 5 times.

1b. Chest Stretch (more advanced)

Note: *<u>Do not do this stretch if you have a shoulder injury.</u>*

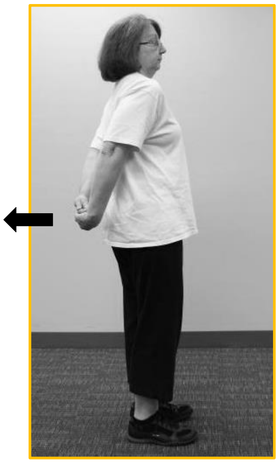

- Clasp your hands behind your back with your palms facing up.
- Pull your hands down and press your shoulder blades together. Your chest should stick out.
- Gently lift arms up, moving hands aways from your back.
- Hold for 15 to 30 seconds. Repeat 3 to 5 times.

Copyright 2017 University of Pittsburgh

2. Waist Bends

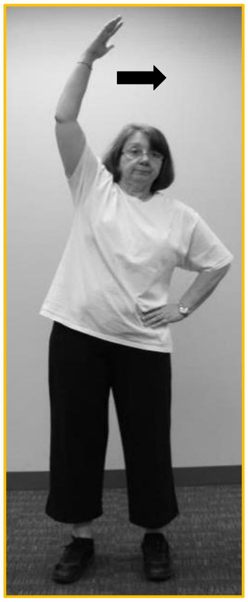

- Stand up tall with your feet shoulder-width apart.
- Bringing your left arm over your head and keep your right arm on your waist. Bend **gently** to the right.
- Look straight ahead.
- Hold for 15 seconds. Repeat 3 to 5 times.
- Repeat the exercise, bending to the left side.

3a. Sitting Hamstring Stretch

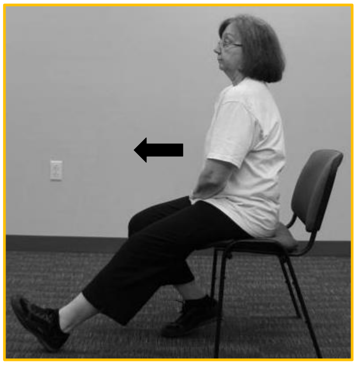

- Sit at the edge of a chair with your knees slightly bent. Straighten one leg out in front. Sit up straight.
- Gently bend forward at the waist, **keeping your shoulders back and your back straight.**
- Hold for 15 to 30 seconds. Repeat 3 to 5 times on both legs.

3b. Hamstring Stretch on Floor (more advanced)

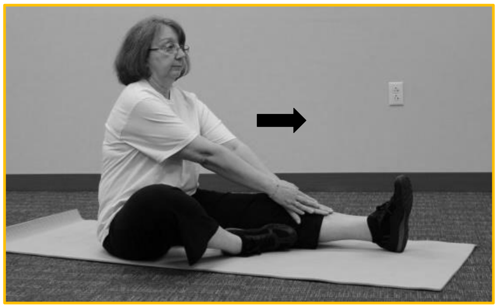

- Sit on the floor with one knee bent.
- Reach out your hands towards the extended leg and bend forward at the waist, **keeping your shoulders back and your back straight.**
- Hold for 15 to 30 seconds. Repeat 3 to 5 times on both legs.

Copyright 2017 University of Pittsburgh

Note: *Do not do these stretches if you have had a hip replacement.*

4a. Figure 4 Stretch –lower intensity

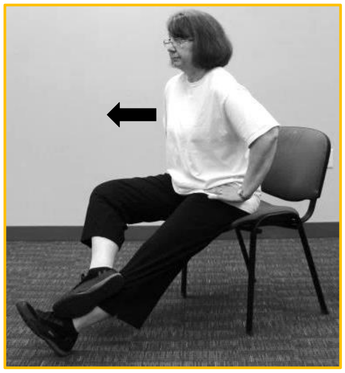

- Cross one ankle **below** the opposite knee and allow knee to fall out.
- To deepen the stretch, gently bend forward at the waist, **keeping your shoulders back and your back straight.**
- Hold 15 to 30 seconds. Repeat 3 to 5 times on both legs.

4b. Figure 4 Stretch (more advanced)

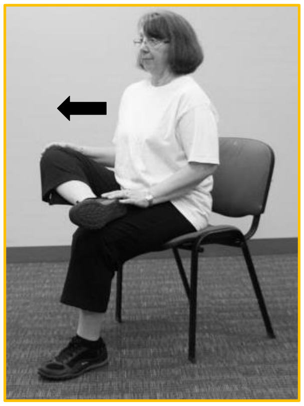

- Keeping your back straight, cross one ankle over the opposite knee and allow knee to fall out.
- To deepen the stretch, bend forward at the waist, **keeping your shoulders back and your back straight**.

5a. Lower Back Stretch

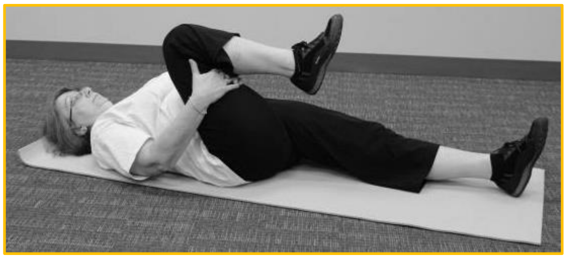

- Can be done on bed or floor.
- Lay on your back, with legs extended.
- Bring one knee into your chest, clasp hands behind thigh.
- Gently pull knee into chest.
- Hold for 15 to 30 seconds. Repeat 3 to 5 times on both legs.

5b. Lower Back Twist (more advanced)

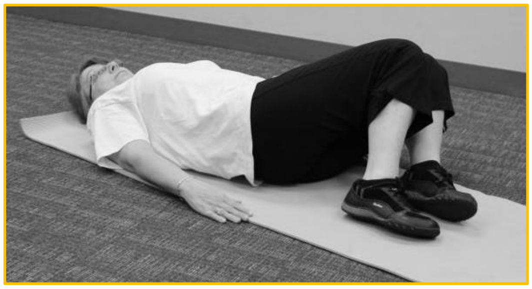

- Lay on your back, bring both knees to 90 degrees with feet on floor.
- Keeping shoulders flat in the ground, let both knees fall gently to same side.
- Hold for 15 to 30 seconds.
- Repeat 3 to 5 times on both sides

Copyright 2017 University of Pittsburgh

6a. Hip Flexor Stretch

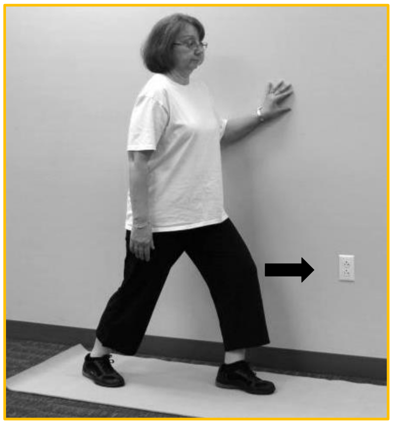

- Take a big step forward. Gently bend the front knee until you feel a stretch in the front of your hip of the extended leg.
- Keep both toes pointing forward. **Do not allow front knee to go over toes.**
- Hold for 15 to 30 seconds. Repeat 3 to 5 times on both legs.

6b. Kneeling Hip Flexor Stretch (more advanced)

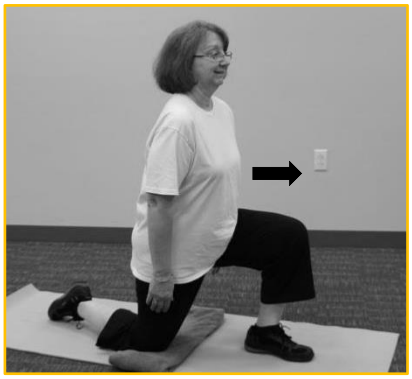

- From kneeling, take a big step forward.
- Keep your upper body straight, while you gently increase the weight on your front foot, **do not allow front knee to go over toes.**
- You should feel the stretch in the front of the hip of the leg that is kneeling.
- Hold for 15 to 30 seconds.
- Repeat 3 to 5 times on both legs.

7. Calf Stretch

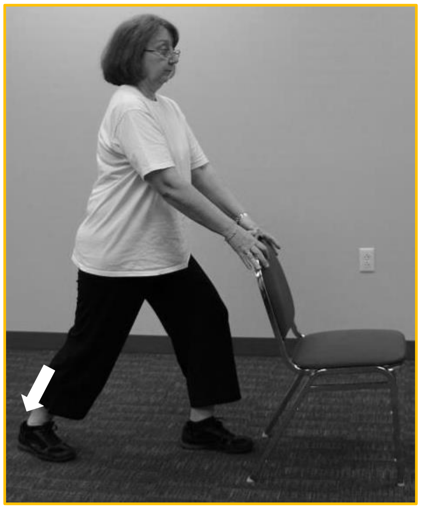

- Stand straight with feet shoulder-width apart.
- Holding on to a **stable** chair or countertop, step forward with your right foot, slightly bending your right knee.
- The front of your knee should be in line with the front of your toes. Your left leg should stay relatively straight and your left heel should remain on the floor. Slightly bend your front knee.
- Hold for 15 to 30 seconds. Repeat on the opposite side.

Copyright 2017 University of Pittsburgh

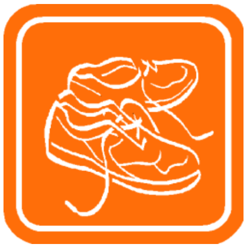

Wear a Good Pair of Shoes

You don't need to buy new or special shoes if the ones you have now fit well and support your feet. Keep these tips in mind when shopping for shoes:

Visit one or more stores you trust. Try on various styles and brands.

You may be able to ask the store staff to help you find shoes that fit well, give the support you need, and suit the kind of activity you do.

Look for a good fit.

- Wear the kind of socks you'll wear when you're active.
- Shop right after you've been active. That way, your feet will be the size they are when you're warmed up.
- Shoes should be comfortable right away. Try them on and walk around. They should NOT need to "stretch out" later.
- There should be one thumb's width of space between your longest toe and the end of the shoe.
- The heel should NOT pinch or slip around when you walk.
- Shoes should bend easily at the ball of your feet (behind your toes).

Get the support you need for the kind of activity you plan to do.

- Your shoes should match the shape of your foot and the way your feet strike the ground.
- Take your old shoes with you to the store. The pattern of wear can show the kind of support you need. For example, is the back of the shoe worn down unevenly on the bottom (that is, does it slant toward the inside or outside)? If so, you may need extra support for arches or flat feet.
- You may want to look for shoes that suit the activity you plan to do. Stores offer many types of shoes for activity, such as running or aerobic dance. They are made to give you the kind of support needed for that activity.

The bottom line is good fit, comfort, and support. You may want to visit a store with expert staff who will know how to help you.

Copyright 2017 University of Pittsburgh

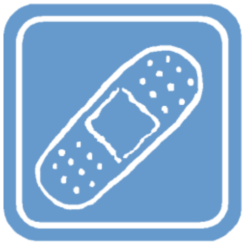

In Case of Injury

Call your health care provider when you suspect a serious injury. If you can't move the injured area, or if there is immediate and severe swelling, these may be signs of a broken bone.

If you get a sprain, strain, "pull" or bruise: Think **R.I.C.E.**

What R.I.C.E stands for	What to do
**Rest (restrict movement)**	• Stop doing the activity.<br>• Rest for a few days.<br>• Sometimes splints, tapes, or bandages are needed.
**Ice**	• Apply ice or cold compresses for the first 24 to 36 hours after the injury. This reduces pain and swelling.<br>• Schedule: 10 minutes on, 10 minutes off.<br>• *Always* wrap ice or compresses in an absorbent towel or cloth. Don't apply directly or wrapped in plastic. That can cause frostbite and more injury.
**Compression (Pressure)**	• Apply pressure by wrapping the injury with an elastic bandage. This helps to reduce swelling and blood flow (pressure) to the area.<br>• The bandage should be tight enough to reduce blood flow but not cut it off completely. Loosen the bandage if your toes or fingers begin to feel numb or lose their color.
**Elevation**	• Lift the injured area above heart level. Keep it elevated whenever possible, not just during icing.<br>• This helps reduce the pooling of blood in the area that can cause pain and throbbing.

If *ever* in doubt about your physical condition, call your health care provider.

Copyright 2017 University of Pittsburgh

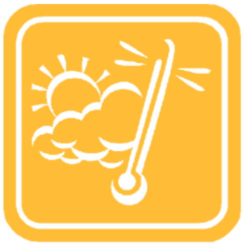

Beat the Heat

- CHECK THE WEATHER ADVISORY. On rare occasions, it may be too hot to exercise outdoors.
- Drink water before, during, and after being active.
- On hot, humid days, be active early or late in the day when it is the coolest.
- Don't overdo it. Go at your own pace.

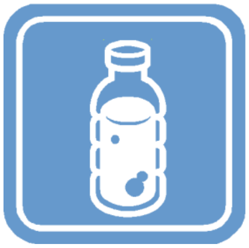

- Wear light, loose-fitting clothes.
- Wear a hat to shade your face from the sun.
- Know the signs of heat illness: paleness, dizziness, nausea, vomiting, fainting, warm and flushed skin, or not sweating in situations in which you would normally expect to sweat.
- Tell someone your route and when you'll be back.
- Better yet, go with a friend.
- Carry a cell phone and some form of ID.

Copyright 2017 University of Pittsburgh

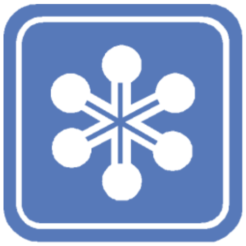

Keep Warm in the Cold

- CHECK THE WEATHER ADVISORY. On rare occasions, it may be too cold and/or icy to exercise outdoors.
- Be active in the middle of the day, when it's the warmest.
- Drink plenty of water, even though you may not be sweating.
- Dress warmly from head to toe.
  - Head: Wear a hat or ear muffs.
  - Body:
    - Dress in several thin layers. Remove or add layers as needed.
    - Full-length long-underwear or tights on your legs.
    - T-shirt, long-sleeved turtleneck.
    - Well-vented wind jacket with a zipper.
  - Hands: Wear mittens or gloves.
  - Feet: Wear well-insulated socks. Wear shoes or boots that keep your feet warm and dry and give you good traction.

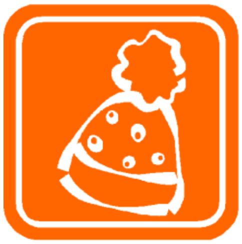

- Go out against the wind and come home with the wind.
- Avoid patches of ice.
- Know the signs of frostbite: numbness, tingling or a burning feeling and/or changes in skin color.
- Tell someone your route and when you'll be back.
- Better yet, go with a friend.
- Carry a cell phone and some form of ID.

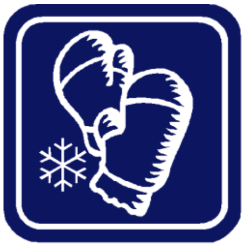

Copyright 2017 University of Pittsburgh

HOW AM I DOING? Tracking Activity: Year-at-a-Glance

Name: _________________ Year: _______

Please enter the date each week and mark an X beside the number of minutes of activity achieved that WEEK.
The MINIMUM physical activity minute goal starting in Week 7 is shaded below.

Min/week																							
Other:																							
300																							
290																							
280																							
270																							
260																							
250																							
240																							
230																							
220																							
210																							
200																							
190																							
180																							
170																							
160																							
150																							
140																							
130																							
120																							
110																							
100																							
90																							
80																							
70																							
60																							
50																							
40																							
30																							
20																							
10																							
Week	4	5	6	7	8	9	10	11	12	13	14	15	16	17	18	19	20	21	22	23	24	25	26
Date																							

HOW AM I DOING? Tracking Activity: Year-at-a-Glance

Name: ________________ Year: ______

Please enter the date each week and mark an X beside the number of minutes of activity achieved that WEEK. The MINIMUM physical activity minute goal is shaded below.

Min/week																										
Other:																										
300																										
290																										
280																										
270																										
260																										
250																										
240																										
230																										
220																										
210																										
200																										
190																										
180																										
170																										
160																										
150																										
140																										
130																										
120																										
110																										
100																										
90																										
80																										
70																										
60																										
50																										
40																										
30																										
20																										
10																										
Week	27	28	29	30	21	32	33	34	35	36	37	38	39	40	41	42	43	44	45	46	47	48	49	50	51	52
Date																										

Session 4 Leader Guide: Move Those Muscles

Objectives
In this session, the participants will:

- Receive the Group Lifestyle Balance™ physical activity goal.
- Discuss the differences between planned and spontaneous/unstructured activity.
- Understand the need to obtain the approval of their health care provider before beginning this program (if required by your program).
- Recognize safety issues related to physical activity and when to stop exercising.
- Identify the many benefits of an active lifestyle.
- Consider time spent sitting and ways to sit less.
- Discuss the recommended gradual progression of this physical activity program.
- Identify other types of moderately intense aerobic activities equivalent to brisk walking that the participants may enjoy.
- Understand the importance of planning ahead for an active week.
- Learn different ways to find the time to be active.
- Understand techniques for safe exercising and stretching to prevent injury including warm up and cool down, staying in control and balance, and avoiding bouncing and holding one's breath.
- Review information on safe stretches to do at home, proper footwear, what to do for a musculoskeletal injury and how to prepare for exercising in hot/cold weather.
- Develop an activity plan for the next session.

To Do Before the Session:

- If providing Food and Activity books; one per participant.
- Review Food and Activity records from last session; add comments.
- Prepare Session 4 handout for participant notebook.
- (Optional) Collect information about local walking trails (with maps/addresses), parks, fitness centers, pools, and dance classes, etc. to distribute to your participants.
- (Optional) Make a plan for supervised activity sessions for your group if your organization is able to do so. This would require a modification to the letter to the health care provider.

If required by your program, make sure all participants have their physician's approval prior to beginning the activity portion of the program

Available in the GLB Providers Portal:
https://www.diabetesprevention.pitt.edu/my/login.aspx

- **Guidelines for Reviewing Food and Activity Records**

Copyright 2017 University of Pittsburgh

Group Sharing Time (allow at least 5 minutes)

In the last session, we talked about healthy eating and the importance of planning ahead for healthy meals and snacks. We discussed MyPlate and how it can be used as a guide to building a healthy eating style. We also talked about how to making shifts to healthier food and beverage choices can promote health and prevent disease now and in the future.

Let's take a few minutes to talk about your experiences this past week. *[Choose only a few of the following prompts to guide discussion.]*

- Overall, how did it go last week? What went well: What problems did you have? What could you do differently?
- What did you learn by self-monitoring? What did you learn about your overall eating pattern? What did you learn about your food choices?
- Were you surprised by the amount of calories and fat in some foods?
- Did you try to eat at regular meal times? How did it work for you?
- Did you experiment with a new eating pattern? Some people do best with 3 meals; other with 3 meals plus snacks. Have you found the pattern that works best for you?
- Were you aware of how long it takes you to eat a meal? Did you try any strategies to help you eat slowly so meals last about 20 minutes?
- Did you make any changes to better match the recommendations of the MyPlate model? How did it work for you?
- Did you make any changes to eat a healthier eating pattern?
- What changes did you make to "replace", "limit", or "include" certain foods in order to meet your calorie, fat, and health goals?
- Did you complete any of the "change I can make" you identified to help you shift to healthier food and beverage choices? How did it go?
- Did you rinse cooked ground beef? If so, please give us your feedback.
- Did anyone try the roasted vegetable recipe? If so, please give us your feedback.
- What problems did you have? How did you solve them?
- What change did you make in your eating pattern this past week that you are most proud of?
- How close did you come to your calorie and fat gram goal?
- Were you able to do something active this past week? What did you do? How did that go?

[Address and discuss barriers. Problem-solve barriers with the group.]

[Praise all efforts to self-monitor and to change eating behaviors. Be positive and nonjudgmental. Encourage group discussion.]

Note: The Leader's Guide for this session is written as if the participants have been relatively sedentary before this time. Use your judgment to adapt your presentation of the session for those participants who are already significantly active.

Copyright 2017 University of Pittsburgh

Session 4: Move Those Muscles (pages 1-2)

So far, you've focused on losing weight through healthy eating. Starting with this session, we will also begin to focus on the other key part of Group Lifestyle Balance™: achieving and maintaining an active lifestyle.

[*Review the information on the top of page 1. Include the following information:]*

What tasks do you do now that require less physical movement than years ago?
[Sample answers: There weren't elevators in every building. Cars were harder to come by as many people could not afford them. Many more people would walk to wherever they needed to go. They had no phone or only one phone and so they ran up or down the stairs to answer it.]

In general, our grandparents performed more movement and physical activity in their typical day than we do now. Activity was a big part of life in past generations. By contrast, most of us now have so many conveniences that our lives are almost guaranteed to be inactive unless we **consciously add movement and activity to our day.**

The overall goal of the activity portion of GLB is to help you find ways to add movement to your daily routine in ways that can be counted (planned) and as well as in ways that would be difficult to count (spontaneous/unstructured). At the end of the day it is the sum of the planned and spontaneous (or unstructured) physical activity that is important.

Let's talk about these two ways to Move Those Muscles*: [Review information in the box on page 1 and include the following:]*

1. **Planned Activity.**
   This physical activity goal is in line with the recommendations of the major health organizations including the Surgeon General, The American College of Sports Medicine, and the Centers for Disease Control.

2. **Spontaneous or Unstructured Activity** consists of activity choices that we make during the day that are too brief to keep track of, but can make a difference in your total activity level.

[Ask participants to think back and answer the questions listed at the top of page 2. Ask participants to share their answers, if they are comfortable doing so.]

Now please think about your thoughts and feelings about what it is like to be active or inactive.

[Review and discuss the 4 questions in the box on page 2. Ask participants to share their answers, if they are comfortable doing so. Encourage discussions about what they might have to give up to be more active. These are all important points to consider.]

Copyright 2017 University of Pittsburgh

What a Bargain: The Many Benefits of an Active Lifestyle (page 3)

I want to be sure you understand why keeping up with your planned physical activity program is so very important.

Physical activity is one of the biggest "bargains" you can get. It has many rewards, some of which are listed on page 3.

[Review page 3. Include the following information:]

Many people report that they simply **feel good** when they're more active, and they really miss it if they've been active for a while and then stop or miss a few days.

Help you lose weight and keep it off.   Research has shown that both diet and physical activity are important to lose weight and keep it off. Diet is the more important of the two for weight loss, whereas physical activity is a relatively more important predictor of successful weight maintenance. A good balance of the two is needed for a healthy lifestyle.

To summarize, physical activity may help you lose weight, be more fit, and feel better in general.

These are some of the many benefits of an active lifestyle. It's the gift that keeps giving.

Which of the benefits of physical activity listed on page 3 are the most important to you? [*Note: Ask participants to share their responses with the group, if they are comfortable doing so). Encourage a group discussion about the many important health benefits of physical activity.]*

Can you see why physical activity is one of the biggest "bargains" you can get?

What You Can Expect from the GLB Physical Activity Program (page 4)

What can you expect from the physical activity program in GLB?

Please be assured that you can succeed with this program. There are several keys to success: *[Refer to graphic on bottom of page 4 as you review the 6 key reasons this program can and will work. Include the following information:]*

- **Realistic starting point**: It does not matter about your current activity level. We will start at the beginning with you, wherever that may be, no matter how inactive, and help you to increase your activity level slowly, steadily, and safely.

Copyright 2017 University of Pittsburgh

- **Keep track**: Just like we asked that you keep track of your food intake, we will now ask you to track your physical activity. This is very important in helping you understand and improve upon your physical activity behavior.
- **Plan ahead**: Making plans to include activity in your week is also very important in reaching your activity goal.
- **Progression**: We'll talk more about progression in a few minutes.
- **SAFETY:** One of the most important keys to success is making sure that you are safe while being physically active. It is important to keep your health care provider informed about your progress in the program and changes you have made in activity, eating, and weight as he or she is very interested in your health and well-being. *[If required by your program, explain the physician's approval from and discuss the policy/procedures for your program.]*

There are some important issues to consider regarding your safety:

- As your GLB coach, I am not able to provide medical advice. I am NOT your health care provider and GLB does NOT replace your regular appointments with your health care provider.
- In all cases of illness, pain, or medication adjustment, you need to go to your health care provider.
- If you get sick, put your activity on hold until you are well again and your health care provider has given you the OK to start exercising again.
- If you feel pain (not muscle aches but pain), especially new pain, stop doing what you are doing and have it checked out by your health care provider.

Your Physical Activity Goal: Planned Activity (page 5)

Let's talk about the first way to Move Those Muscles and add movement back into your daily routine by developing a planned physical activity program that will work for you.

[Review the information on page 5. Include the following information:]

As discussed in Session 1, the Group Lifestyle Balance™ activity goal is to **reach and maintain a minimum of 2-1/2 hours (that's 150 minutes) of physical activity each week.**

[Review the 5 bullet points that describe the GLB physical activity goal. Include the following:]

- Choose forms of physical activity that are of moderate intensity, like brisk walking. You will not be asked to do anything extreme.

- This may sound like a lot right now, but you will work up to this goal slowly. For example, you might start by walking 10 minutes a day on 6 days a week and slowly build up to 25 minutes on 6 days a week. Starting slowly and spreading it out over the week is important so you don't get hurt and so you enjoy yourself.

Copyright 2017 University of Pittsburgh

- **Pick activities you LIKE.** The goal is for you to develop a lifelong habit of being active, so choose activities that you enjoy and ones that are also appropriate and feasible for you and your lifestyle.

  A good example of this is when you encourage "active" family time. *[Ask participants to give name some examples of what they have done or could do with the family that is active such as a walking/hiking, biking, bowling, going to the pool, or playing miniature golf.]*

The bottom line is that in GLB you will be designing your own personal physical activity plan that will work for you.

For participants who want more information: Over time we will increase your Group Lifestyle Balance™ activity goal to 2-1/2 hours (150 minutes) of brisk activity per week. This goal is very similar to the national physical activity recommendations for adults. The Surgeon General's Report on Physical Activity and Health recommends that American adults participate in moderately intense physical activity, such as brisk walking, for 30 minutes on most or all days of the week which is quite similar to the Group Lifestyle Balance™ physical activity goal.

Some participants may have their own personal goals. Some may already be very active. Some may wish to do less than 150 minutes of activity each week. Briefly address their concerns. Here are some examples of ways to address individual concerns:

- If the participant *wants to be less active* than the GLB goal: "We'll work toward this goal slowly, one step at a time. It's a safe and reasonable goal for you, and I'm very confident that you can do it."
- If the participant *wants to be more active* than the program goal: "Let's work toward this goal first. When you reach this goal, we'll talk about going further."

[Discuss the final question. Encourage group discussion.]

(Optional) In the Diabetes Prevention Program, supervised activity sessions were provided several times a week. These sessions were found to be helpful to many participants in reaching their activity goal. Offer them, if possible, at your location. If you are able to offer group activity sessions at your location, make certain that the activity sessions are appropriate for all. Announce this to your group and be sure to encourage all to participant. Describe the sessions and distribute a schedule and map as needed.

(Optional) Another helpful tip is to provide your participants with maps and addresses of walking trails, parks, fitness centers, pools, dance classes, etc., in your area. Distribute this information to your group.

Copyright 2017 University of Pittsburgh

Physical Activity Progression: Where should I start? (page 6)

As you know, the activity goal in this program is a minimum of 150 minutes per week. You will work up to this goal *slowly* to prevent injury and to establish a routine.

Let's talk about where you should start. *[Review information at the top of page 6. Make sure participants understand their activity goal for this coming week.]*

The box on page 6 shows an example of how to gradually and safely increase your minutes of physical activity. *[Review "How to progress" information outlined in the box. Make it clear to participants that the goal of the program is to build "time over intensity". This means that, until participants are regularly performing 150 minutes of moderate intensity activity, they should work on building up the* ***time****, before increasing the* ***intensity*** *of the sessions. For safety reasons, we do not recommend doing high intensity bouts of activity when beginning an activity program.]*

Types of Aerobic Activities *[Review the information and include the following:]*

Aerobic activities are the types of activity recommended in this program.

Most people choose to do **brisk walking.** It's easy to do and good for you. All you need is a good pair of shoes, and it can be done almost anywhere. (By "brisk" walking, we mean more than a stroll. **Walk fast enough to breathe heavier than usual and to note that you are working hard, but not so fast that you can't carry on a conversation or that you have trouble breathing.**)

Page 6 lists many other kinds of activity that are good, too. **What other activities might you like to do?** Circle the activities that you are interested in trying.

Discuss activities participants enjoy and **check whether these activities are similar to brisk walking.** Make the point that **how hard an activity is performed will have a big impact on whether it is similar to brisk walking** (e.g., the difference between swimming laps and simply moving slowly in the water.

It is very important to plan activities you LIKE. After all, the point is to make physical activity a regular part of your lifestyle, and that will never happen unless you enjoy the activities you do.

Please note that resistance training is not considered an aerobic activity but is highly recommended. This will be discussed in an upcoming session.

Copyright 2017 University of Pittsburgh

Plan for an Active Week (page 7)

Last week you were asked to be more aware of your physical activity. You were also asked to write down any physical activities you did during the week.

What is new this week is for you to start recording your minutes of physical activity. To help you reach your goal for physical activity each week, it is important to make a plan for a more active week. That will help you find time to be active.

Here are two different ways to plan your activity.

1. **Set aside one block of time for planned activity on most days.** Make being active a predictable part of your daily routine, like taking a shower may be a predictable part of your morning. Think of **when** you'll be active; not **if** you'll be active today.

> If possible, use an example that is particularly relevant to the participants' lifestyles. For example, people who work in office jobs may want to experiment with holding group meetings where everyone stands for a period of time. Or, they may try conducting a meeting with just a few people while "walking and talking".

Some people can't find one big block of time in a day to be active. Either their schedules vary a lot from day to day, or they're so busy that there isn't a 20-30 minute period that's free on most days. For some people, this might be the case during certain seasons of the year, for example, during the fall when after-school schedules begin to get hectic for their kids. In these situations, it's usually easier to use a different approach.

So, another way to plan your activity is to split up the time into bouts of exercise that last at least 10 minutes.

2. **Set aside 2-3 smaller, blocks of time during the day to be active. These should last 10 minutes or longer.** For example, you might be able to take a 10-15 minute break every day at lunchtime and go for a brisk walk. Then later, take another 10-15 minute walk right after dinner. If you can figure out a third 10-15 bout somewhere in your day, you will have accumulated 30-45 minutes of activity throughout your day. Remember to write this activity and the minutes you were active in your Food and Activity Record.

Your **goal for between now and the next session** will be to get a total of at least **60 minutes of activity** per week. Think about your typical day. **When can you set aside 15 to 20 minutes to do an activity you like?** Are you a morning person? Or would you enjoy getting out for a walk during lunch? How about after dinner? *[Those who did more than 60 minutes of activity last week are asked to do the same amount of activity this week.]*

[Give participants time to complete the chart on page 7.]

Copyright 2017 University of Pittsburgh

Whether you are able to perform activity in one longer block of time or split it up into several times throughout the day, remember to record the activity in your Food and Activity record. Please record both the type of activity and the minutes you were active.

To stay healthy, you'll want to make being active a part of your regular routine so that you can establish it as a habit for a lifetime. Granted, being active takes time out of your day, but it is time well spent. Commit to finding the **time to be active.**

In contrast to planned activity, "**spontaneous or unstructured activity**" involves **making last minute, spontaneous, short-duration activity choices throughout the day.** It's hard to record this kind of activity, so **we aren't asking you to record it in your Food and Activity record.** But it's an important way to add movement in your day and it does make a difference.

An example of an **inactive choice** is when you shop, park your car as close as you can to the entrance to the store. An example of an **active choice** is to walk up the stairs rather than taking the elevator. This may only take a few minutes to do and would be annoying to keep track of, but every minute of activity has an impact on your overall health and adds up to a "more active you."

The Smart Way to be Active: Keep It Safe (pages 8-9)

Being active is usually quite safe. Injury due to a moderate activity program like the one recommended in GLB is not common. The best approach is prevention. Practice safe activity techniques that will help keep you safe and prevent injuries when being active.

5 key ways to keep it safe when being active: *[Review pages 8-9. Include the following information:]*

1. WARM-UP and COOL-DOWN before each activity session.
2. Be sure to KEEP IN CONTROL. Maintain stability.
3. Be sure to KEEP BALANCED. Work both sides of the body equally.
4. Remember to BREATHE. Do not hold your breath.
5. STRETCH. We'll talk more about stretching exercises as we review the handout.

[Review "Important Message from Your Coach" in the box on page 9. Include the following information:]

No Pain IS Gain: Always listen to your body. If you have sharp or intense pain during a particular movement, stop doing it.

Copyright 2017 University of Pittsburgh

Refer participants to the information in the "Resources" section: **Stretches and warm-up activities (pages 14-17), footwear information (page 18), handling an injury (page 19) and exercising in the heat and cold (pages 20-21). On pages 22-23 is a chart that can used to track minutes of activity each week for a year.** That is optional, but many participants enjoy doing it**.**

Demonstrate a few of the stretches if possible. Refer to page 15, exercise 2 (Waist Bends) as an example in which the individual has introduced control to their movement. Again, **it's very important not to intimidate the participants or make them worry that being active is unsafe. These are precautionary measures to reduce injuries.**

And please remember, GLB is not taking over any medical care of the participant. If a participant complains about any medical problem whatsoever, including injuries, the participant should be directed to his/her health care provider.

When to stop exercising (page 10)

Although being *physically active is usually* very safe, there are some times when you should stop exercising. Let's review some of these times:

[Review the information on page 10. Include the following information:]

All of these signs and symptoms are important because they could be signs of something serious like a heart attack and should never be ignored.

Remember that all cases of chest pain and/or discomfort should be brought to the attention of your health care provider as soon as possible, especially if they come on with new activity or increased exertion.

Even if your symptoms go away, it is important to let your health care provider know about the incident as soon as possible.

Your Physical Activity Goal: Spontaneous/Unstructured Activity (page 11)

Now let's talk about the second way to Move Those Muscles and add movement back into your daily routine by adding more spontaneous/unstructured activity.

Copyright 2017 University of Pittsburgh

Ask participants to name some examples of ways to add spontaneous/unstructured activity to their day. Encourage group discussion.

Examples include:

- Park your car further away from work or from the entrance to a shopping mall and grocery store.
- Get off the bus one stop early and walk the rest of the way.
- Walk down the hall to talk with a coworker instead of calling or emailing.
- Walk to a nearby store rather than driving.
- Do yard work rather than hiring someone else to do it.
- Take the stairs rather than an elevator.
- Go for a 2-minute walk during TV commercials (especially food commercials!).
- Do stretching exercises while watching TV.

[Review page 11. Include the following information:]

Decrease the time you spend sitting. There is increasing evidence that it's important not only to be active, but to shorten and break up periods of sitting.

The first important step is to be aware of how much time you spend sitting each day. Then you can start to find ways to sit less during the day and to break up long bouts of sitting. This will help to add more movement to your day and may benefit your health.

Many people say that they have no time to take a walk but yet, they make time to watch several hours of television in the evening. **Try cutting down some of your TV time and take a walk instead.**

At first, you may not see walking as a way to relax after a long day. But when you get used to it, you'll discover that walking is a great way to relax and unwind, and you may feel much more rested and refreshed than you would have had you spent that time sitting in front of the TV.

Look at the question at the bottom of the page. Can you think of some ways you could make an active choice or to decrease the time you spend sitting? *[Examples: Be active while you watch TV. Ride an exercise bike or walk on a treadmill. Encourage participants to share examples to list a few activities they are willing to try.]*

To Do (page 12)

Turn to page 12 and let's focus on **what you can do between now and the next session.**

[Review page 12 and include the following information. If required by your program, explain the physician's approval from and discuss the policy/procedures for your program.]

Ask participants to fill in the "Be active for ___ minutes" with "60 minutes" or the greater amount in the case they are already doing more than 60 minutes of activity.

For example, you could do 12 minutes of activity on 5 different days of the week. We'll gradually increase this over the coming months until you're up to 150 minutes per week.

- **Include a friend or family member if you would like.** Some people like to be active alone and enjoy this time to do something special for themselves. But many people find it helpful to be active with someone else. Is there anyone you would like to invite to walk with you?

- **Plan activities you LIKE to do. Follow your Activity Plan on page 7.** Warm up, cool down, and follow the guidelines for safe stretching. And ALWAYS PRACTICE SAFE EXERCISE HABITS.

If using the GLB Food and Activity book, show participants where they should record physical activity. Record the time of day they were active, the type of activity, and the number of minutes they were active.

You may also record the distance in miles that you are walking, if you want to do so.

Use one line for each time you're active, even if it's the same kind of activity. For example, if you take a walk at 8:00 in the morning and another one at 7:00 in the evening, write both down separately.

It's also important to **record only the amount of time you were actually *doing* the activity.** Don't include the time when you may have been taking a short break.

- For example: You walked for 10 minutes, then you ran into a friend and stopped to talk for 5 minutes, then walked 10 more minutes. How many minutes of activity should you record? *[Answer is 20 minutes of walking, not 25.]*

- Another example: If you were in the pool for 60 minutes but only swam laps for 10 of those minutes, how many minutes of activity should you record? *[Answer is 10 minutes of swimming.]*

Let's look at a few examples and discuss how you would record this activity:

- Strolling around the mall for an hour while shopping with a friend. *[Answer is 0 due to low intensity. However, it is a great choice for increasing overall movement rather than choosing a sitting activity.]*

- Doing light household chores (dust, vacuum, wash dishes) for 20 minutes. *[Answer is 0 since light intensity. Again, this is a great option for increasing overall movement rather than choosing a sitting activity.]*

Copyright 2017 University of Pittsburgh

[Announce the day, time, and place for the next session.]

After the session:

- Weigh participants who did not do so prior to the group meeting.
- Complete data forms and documentation required in your setting.
- Follow your program's protocol for managing absences.
- Review the self-monitoring records from the previous week. Write brief comments. Be positive and nonjudgmental.
  - Praise all efforts to self-monitor, change eating behaviors, and add activity.
  - Highlight any positive changes made that relate to the session topic of the week before the records were collected: using MyPlate recommendations, making shifts in food choices in order to use healthy fats and more fiber and less saturated fats, trans fat, sodium, and added sugars.
  - Refer to **Guidelines for Reviewing Food and Activity Records** available in the GLB Providers Portal.

Copyright 2017 University of Pittsburgh

Session 5: Tip the Calorie Balance

The Group Lifestyle Balance Program™ involves two lifestyle changes:

1. Healthy eating.
2. Being physically active.

Both are part of calorie balance:

- Calories In
    - Calories are a unit of measure. Calories are used to measure the energy value of foods and beverages. When you eat and drink, you take in calories.
    - The number of calories in a food or beverage depends on the amount of fat, carbohydrates (starches and sugars), protein, and/or alcohol it contains.
    - Fat has the most calories per gram. A gram is a unit of weight. Fat has more than twice the calories as the same amount of protein or carbohydrate. Note: Alcohol is high in calories too.

Calories per Gram*	Carbohydrates (Starches and Sugars)	Protein	Alcohol	Fat
	4	4	7	9

- Calories Out
    - Calories are also used to measure the energy you spend. You use calories for just staying alive (for bodily functions like breathing) and by being physically active.

Be aware of what you eat and drink and how active you are.

Copyright 2017 University of Pittsburgh

Calorie Balance: Calories In and Calories Out

Your weight is a result of the balance between:

- Calories in from what you eat and drink
- Calories out from moving more (planned and spontaneous activity during your day)

If you want to change your weight, you need to "tip the balance".

The best way to "tip the balance" is to both eat and drink less and be more physically active.

"Tipping the balance" will help you lose weight and improve your health.

Calories In   Calories Out

1. Your weight stays about the same.

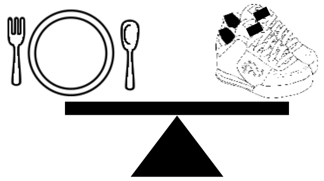

---

2. Tipping towards weight gain.

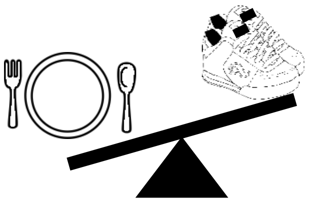

---

3. Tipping towards weight loss.

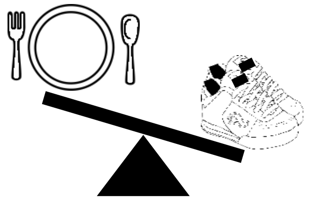

---

4. You can reach a new balance at a lower weight.

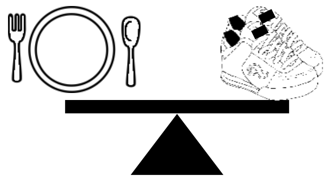

Copyright 2017 University of Pittsburgh

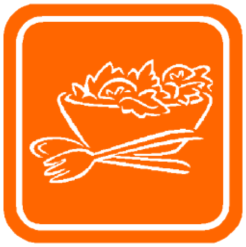

Healthy Eating and Being Active Work Together

To lose weight and improve health, it's best to eat less *and* be more physically active. That way, you change both sides of the balance at once.

Tip the calorie balance to lose weight.

Work to find a new balance at a new, lower weight.

Make the eating and activity changes part of your lifestyle, to keep the weight off.

How much does it take to tip the balance?

- Eating 500 fewer calories per day should result in losing about 1 pound per week. Research shows that this varies from person to person.
- Slow, steady weight loss (an average of 1 to 2 pounds per week) is the best way to lose weight.
- Your calorie goal was set to help you "tip the balance" enough to reach your goal weight.
- Beware of falling into the "because I exercised" trap. Physical activity uses calories, but not as many as people think.
  For example, 1 mile of brisk walking (15-20 minutes) uses about 100 calories.

To lose:	Tip the balance by this number of calories:
1 pound per week	Roughly 3,500 per week (or 500 each day for 7 days)

Eat at least 1200 calories per day to have a healthy, well-balanced diet.

Copyright 2017 University of Pittsburgh

Changes You Have Made So Far

To be more active:

Planned Activity: ____

Spontaneous Activity: ____________________________________

To change your eating pattern:

Fewer calories: ____

Healthier food choices: ___________________________________

Have these changes *tipped the calorie balance*?

Look on your Weekly Record for the following:

- Your goal of 7% weight loss: _____________
- Your Session 1 weight: _____________
- Your Session 5 weight (today): _____________

Change in weight so far: _____________

☐ No, I weigh as much or more than I did at Session 1.

- To lose weight, try something else to tip the balance.
- We'll work together to find what will work better for you.

☐ Yes, I have lost 1-5 pounds so far.

- You have made some progress.
- To lose more weight, try something else to tip the balance further.

☐ Yes, I have lost more than 5 pounds so far.

- You have tipped the balance.
- Keep tipping the balance, and you'll keep losing weight.

Copyright 2017 University of Pittsburgh

New Ways to Tip the Balance

Keep Track

- Record everything. Watch portion sizes.
- Try a new way to spread your calories throughout the day.
- Try a digital option for tracking eating and activity.

Be Active

- Move more throughout the day.
- Borrow an exercise DVD from your local library.
- Join an exercise class or gym.
- Find ways to be more active using TV or the internet.
- Find an activity buddy.

Try New Recipes

- Look for healthy recipes online.
- Borrow low-calorie cookbooks from your local library.

Be Aware of Liquid Calories

- Drink more water.
- Replace sugar-sweetened beverages (soft drinks, sweetened coffee drinks, sweet tea, etc.) with ones that are calorie-free.
- Limit liquid calories from alcohol.
    - Alcohol increases appetite and lowers self-control.
    - Calories in alcohol are called "empty calories" because they don't have healthy nutrients.
    - Health experts recommend no more than two drinks per day for men and no more than one drink per day for women. If you don't consume alcohol, don't start.
- Limit liquid calories from coffee drinks.
    - Be aware of added sugars and/or fat.
    - Health experts say that moderate coffee intake is not associated with an increased risk of disease. This is defined as no more than 3 to 5 cups (8-ounce each) per day (or up to 400 mg/day of caffeine). If you don't consume caffeine, don't start.

Copyright 2017 University of Pittsburgh

Try a New Eating Pattern

Eating patterns provide ideas for healthy meals and snacks.

For example:

- USDA. These websites list 12 calories levels with the daily amount needed from each food group.
  - Healthy U.S.-Style Eating Pattern. https://health.gov/dietaryguidelines/2015/guidelines/appendix-3/
  - Healthy Mediterranean-Style Eating Pattern. This eating pattern is based on the positive health outcomes seen in studies of Mediterranean-Style diets. https://health.gov/dietaryguidelines/2015/guidelines/appendix-4/
  - Healthy Vegetarian Eating Pattern. This eating pattern does not contain meat, poultry, or seafood but does include dairy and eggs. It can be adapted for vegan diets. https://health.gov/dietaryguidelines/2015/guidelines/appendix-5/
- MyPlate. Go to "Online tools" on this website.
  - Choose the "Daily Checklist" option if you want general information about how much to eat for your calorie level.
  - Choose the "SuperTracker" option if you would like to create a personalized plan.

  https://www.choosemyplate.gov/
- The GLB Healthy Menu Ideas are on pages 9-11. These menu ideas are based on your calorie and fat gram goals and give you an example of how you might spread those calories throughout the day. Menus for meals and snacks, recipes, and a shopping list are included.

What can you see yourself doing with your eating and activity behaviors to "tip the balance" further?

____

Increasing physical activity and eating fewer calories helps tip the calorie balance towards weight loss. These two lifestyle changes are key to improving your health and well-being.

Copyright 2017 University of Pittsburgh

To Do:

Check the boxes when you complete each item:

☐ Keep track of your weight. Weigh yourself at home at least once a week. Record it.

☐ Record everything you eat and drink every day. Come as close as you can to your calorie and fat gram goals.

New things to practice:

☐ Tip the balance (see pages 5 and 6). What changes did you make?

____

☐ Be active for _______ minutes this week. Record what you do.

- The suggested activity goal for last week was 60 minutes.
- If you reached 60 minutes, try adding 30 minutes this week.
- If you were active for less than 60 minutes, that's okay. Start at your current activity level and try adding 30 minutes more.
- If you are doing more, great work and keep it up.

Look for blocks of time when you could be active. Have these blocks of time last for 10 minutes or more. Plan activities you LIKE to do.

	What You Will Do	When	Minutes
Monday			
Tuesday			
Wednesday			
Thursday			
Friday			
Saturday			
Sunday			
Total minutes for the week (My goal: ___ minutes)			

☐ Make active choices throughout the day. List some of the ways you increased your spontaneous activity.

____

Copyright 2017 University of Pittsburgh

Session 5: Resources

Session 5: Resources	Page
The Group Lifestyle Balance™ Healthy Menu Ideas	9
Menu Ideas for 1200 Calories	10
Menu Ideas for 1,800 Calories	11
Breakfast Food List	12
Light Meal Food List	13-14
Main Meal Food List	15-16
Snack List	17
Shopping List	18
Lentils Ole	19

Copyright 2017 University of Pittsburgh

The Group Lifestyle Balance™ Healthy Menu Ideas

Using these menu ideas may:

- Provide structure. You have fewer choices and decisions to make.
- Help you stay away from high calorie foods in the store and not bring them into your house.
- Make it easier and faster to shop for food. We give you a simple shopping list.
- Make it easier to keep track. The calorie and fat grams are listed for meals and snacks.
- Show you how to spread your calories throughout the day. This helps you feel fuller and be less likely to make unhealthy food choices or eat too much.

About the GLB Healthy Menu Ideas:

- The calories are spread over the day as follows:

	Breakfast	Light Meal	Main Meal	Snacks
1,200 calories:	200-300	300-400	500-550	200
1,800 calories:	250-400	400-500	600-650	200-400

- If your calorie goal is 1,500 calories:
  Begin with the Menu Ideas for 1,200 Calories. Add 300 calories by adding more servings from one or more food groups.
- If your calorie goal is 2,000 calories:
  Begin with the Menu Ideas for 1,800 Calories. Add 200 calories by adding more servings from one or more food groups.
- The calories and fat grams on the food lists are averages. If you eat a packaged food, use the values found on the package's Nutrition Facts label.

Note: Over time, you will come up with your own healthy eating pattern that suits your lifestyle. You may want to return to these menu ideas if you feel you need more structure.

Copyright 2017 University of Pittsburgh

Menu Ideas for 1,200 Calories

Mix and match the menus below for breakfast, a light meal, a main meal, and snacks. Choose foods from the attached lists. Make sure to follow the portion sizes on the lists.

Breakfast<br>200-250 calories	• Cold or hot cereal<br>• 1 cup milk<br>• ½ cup fruit juice or 1 fruit serving	• Peanut butter or low-fat cheese<br>• Toast (1 slice), or ½ English muffin/bagel<br>• ½ cup fruit juice or 1 fruit serving	• Egg or egg substitute<br>• Toast (1 slice), or ½ English muffin, or ½ bagel, or 1 tortilla<br>• ½ cup fruit juice or 1 fruit serving	• Yogurt<br>• Fruit<br>• Chopped walnuts
Light Meal<br>300-400 calories	• Sandwich:<br>• 1 protein serving (chicken, turkey, salmon, or tuna)<br>• 1 slice low-fat cheese<br>• 2 bread servings<br>• Mayonnaise<br>• Raw vegetables<br>• 1 cup milk or yogurt	• Salad: Salad vegetables, 1 serving of turkey, chicken, tuna, or salmon, with olive oil vinaigrette<br>• 1 pita or ½ bagel or whole grain crackers<br>• 1 cup milk or yogurt	• Cottage cheese<br>• Fruit<br>• 1 pita or ½ bagel or whole grain crackers	• Low-calorie frozen entrée<br>• Salad with balsamic vinaigrette<br>• Fruit
Main Meal<br>500-550 calories	• Fish or poultry (baked or broiled)<br>• Pasta, potato, rice, barley, couscous, yam, or quinoa<br>• Vegetable with soft (tub) margarine<br>• Fruit	• Low-calorie frozen entrée<br>• Salad with avocado and low-fat dressing<br>• Vegetable with soft (tub) margarine<br>• Fruit	• Lean beef or pork<br>• Pasta, potato, rice, barley, couscous, yam, or quinoa<br>• Salad with sunflower seeds and low-fat dressing<br>• Vegetable<br>• Fruit<br>• 1 cup milk or yogurt	• Chicken stir-fry<br>• Rice<br>• Fruit<br>• 1 cup milk or yogurt
Snack<br>200 calories	Mix and match choices from the Snack List to total 200 calories.			

Copyright 2017 University of Pittsburgh

Menu Ideas for 1,800 Calories

Mix and match the menus below for breakfast, a light meal, a main meal, and snacks. Choose foods from the attached lists. Make sure to follow the portion sizes on the lists.

Breakfast 250-400 calories	• Cold or hot cereal<br>• 1 cup milk<br>• ½ cup fruit juice or 1 fruit serving<br>• Toast (1 slice) or ½ English muffin/bagel	• Peanut butter or low-fat cheese<br>• Toast (2 slices), or 1 English muffin/bagel<br>• ½ cup fruit juice or 1 fruit serving	• Egg/Egg substitute<br>• Toast (2 slices), or 1 English muffin, bagel, or 1 tortilla<br>• ½ cup fruit juice or 1 fruit serving	• Yogurt<br>• Fruit<br>• Chopped walnuts<br>• Toast (1 slice), or ½ English muffin or ½ bagel
Light Meal 400-500 calories	• Sandwich:<br>• 1 protein serving (chicken, turkey, salmon, or tuna)<br>• 1 slice low-fat cheese<br>• 2 bread servings<br>• Mayonnaise<br>• Raw vegetables<br>• Fruit<br>• 1 cup milk or yogurt	• Salad: Salad vegetables, 1 serving of turkey, chicken, tuna, or salmon, with olive oil vinaigrette<br>• Pita, bagel, or whole grain crackers<br>• Fruit<br>• 1 cup milk or yogurt	• Cottage cheese<br>• Fruit<br>• Pita, bagel, or whole grain crackers	• Low-calorie frozen entrée<br>• Salad with balsamic vinaigrette<br>• Fruit
Main Meal 600-650 calories	• Fish or poultry (baked or broiled)<br>• Pasta, potato, rice, barley, couscous, yam, or quinoa<br>• Vegetable with soft (tub) margarine<br>• Dinner roll<br>• Fruit	• Low-calorie frozen entrée<br>• Salad with avocado and low-fat dressing<br>• Vegetable with soft (tub) margarine<br>• Dinner roll<br>• Fruit	• Lean beef or pork<br>• Pasta, potato, couscous, rice, barley, yam or quinoa<br>• Salad with sunflower seeds & low-fat dressing<br>• Vegetable<br>• Fruit<br>• 1 cup milk or yogurt	• Chicken stir-fry<br>• Rice<br>• Fruit<br>• 1 cup milk or yogurt
Snack 200 - 400 calories	Mix and match choices from the Snack List to total 200-400 calories.			

Copyright 2017 University of Pittsburgh

Breakfast Food List

Grains	1 Serving	Calories	Fat (g)
Bran flakes	¾ cup	100	0
High-fiber cereal	¾ cup	100	1
Oatmeal, cooked	1 cup	120	2
Whole wheat bread	1 slice	80	1
Tortilla, whole wheat	1(6″ diameter)	95	2
Tortilla, corn	.7 ounce	40	1
English muffin, whole grain	½ muffin	65	.5
Bagel, whole grain	½ (3" diameter)	100	1
**Dairy**			
Fat-free milk (or lactose free)	1 cup	90	0
Low-fat or light soy milk	1 cup	105	2
Low-fat cheese	1 ounce	60	2
Fat-free yogurt, no sugar added	6 ounces	100	0
**Protein**			
Egg substitute	½ cup	60	0
Large egg	1	70	4
Egg whites	3	50	0
Nuts, chopped (unsalted)	2 Tablespoons	95	9
Peanut butter or other nut butters	1 Tablespoon	95	8
**Fruit**			
Fruit juice with no added sugar	½ cup	50	0
Banana	½ (8″ long)	60	0
Fresh fruit	1 small/medium	80	0
Fruit canned in juice	½ cup	70	0
Grapefruit	½ medium	41	0
Berries, any kind, fresh or frozen	1 cup	70	0
Melon, any kind	1 cup	60	0
Dried fruit	2 Tablespoons	60	0
**Miscellaneous**			
Soft (tub) margarine	1 Tablespoon	70	7
Regular jam or jelly, any flavor	1 Tablespoon	50	0
Low sugar jam or jelly, any flavor	1 Tablespoon	25	0

Copyright 2017 University of Pittsburgh

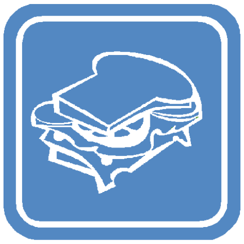

Light Meal Food List

Frozen Low-Calorie Entrées:
Choose any which have less than 300 calories and 10 grams of fat.

Protein	1 Serving	Calories	Fat (g)
Tuna, canned in water	3 ounces	99	1
Salmon, canned in water	3 ounces	130	6
Turkey or chicken breast, roasted	3 ounces	140	3
Lean beef or pork	3 ounces	100	3
Tofu	½ cup	94	6
Soy burger	1 patty	95	2.5
Hummus	2 Tablespoons	50	2
Cooked dried beans or canned	½ cup	110	.5
Nuts, chopped (unsalted)	2 Tablespoons	95	9
Seeds (unsalted)	2 Tablespoons	93	8
**Grains**			
Pita , whole grain	1 (4″ diameter)	75	1
Bagel, whole grain	½ (3″ diameter)	100	1
Whole wheat bread	1 slice	80	1
Tortilla, 6-inch, whole wheat	1 (6″ diameter)	95	2
Tortilla, corn	.7 ounce	40	1
Whole grain fat crackers	6	100	3
**Fruit**			
Fresh Fruit	1 small/medium	80	0
Banana	½ (8" long)	60	.5
Fruit canned in juice	½ cup	70	0
Dried fruit	2 Tablespoons	60	0
Berries, any kind, fresh or frozen	1 cup	70	0
Melon, any kind	1 cup	60	0
**Dairy**			
Fat-free milk (or lactose free)	1 cup	90	0
Low-fat or light soy milk	1 cup	105	2
Low-fat cheese	1 ounce	60	2
Fat-free yogurt, no sugar added	6 ounces	100	0
Cottage cheese, low-fat	½ cup	80	1

Copyright 2017 University of Pittsburgh

Light Meal Food List (continued)

Vegetables	1 Serving	Calories	Fat (g)
Avocado	1/4	80	7.5
Tomato or vegetable juice	1 cup	40	0
Salad greens (lettuce, spinach)	1 cup	7	0
Raw vegetables (carrot, celery, bell pepper)	½ cup	20	0
**Oils**			
Light salad dressing	2 Tablespoons	70	5
Balsamic vinaigrette	2 Tablespoons	60	5
Olive oil vinaigrette	2 Tablespoons	70	5
Mayonnaise	1 Tablespoon	90	10
Light mayonnaise	1 Tablespoon	35	3
Soft (tub) margarine	1 Tablespoon	70	7

Copyright 2017 University of Pittsburgh

Main Meal Food List

Frozen Low-Calorie Entrées:
Choose dinners which have less than 400 calories and 12 grams of fat.

Protein	1 Serving	Calories	Fat (g)
Halibut	3 ounces	125	3
Cod	3 ounces	95	1
Tuna, yellow fin, fresh	3 ounces	115	1
Salmon	3 ounces	175	10
Turkey or chicken breast, roasted	3 ounces	140	3
Lean beef or pork	3 ounces	100	3
Tofu	½ cup	94	6
Soy burger	1 patty	95	2.5
Cooked dried beans or canned	½ cup	110	.5
Nuts, chopped (unsalted)	2 Tablespoons	95	9
Seeds (unsalted)	2 Tablespoons	93	8

Grains			
Pasta (cooked.), whole wheat	1 cup	175	1
Brown rice	½ cup	110	1
Barley, cooked	½ cup	97	0
Couscous, cooked	½ cup	88	0
Quinoa, cooked	½ cup	110	2
Dinner roll	1	84	0

Vegetables			
Tomato or vegetable juice	1 cup	40	0
Cooked non-starchy vegetable (Brussels sprouts, cabbage, carrots, cauliflower, broccoli, green beans, spinach, zucchini, beets)	1 cup	60	0
Cooked starchy vegetables (Corn, peas, lima beans)	½ cup	70	.5
Potato, baked in skin	Medium	180	0
Sweet potato (yam)	Medium	105	0
Salad greens (lettuce, spinach)	1 cup	7	0
Raw vegetables (carrot, celery, bell pepper)	½ cup	20	0
Avocado	1/4	80	7.5

Copyright 2017 University of Pittsburgh

Main Meal Food List (continued)

Dairy	1 Serving	Calories	Fat (g)
Fat-free milk (or lactose free)	1 cup	90	0
Low-fat or light soy milk	1 cup	105	2
Low-fat cheese	1 ounce	60	2
Fat-free yogurt, no sugar added	6 ounces	100	0

Fruit			
Fresh Fruit	1small/medium	80	0
Banana	½ (8" long)	60	.5
Fruit canned in juice	½ cup	70	0
Dried fruit	2 Tablespoons	60	0
Berries, any kind, fresh/frozen	1 cup	70	0
Melon, any kind	1 cup	60	0

Oils			
Light salad dressing	2 Tablespoons	70	5
Balsamic vinaigrette	2 Tablespoons	60	5
Olive oil vinaigrette	2 Tablespoons	70	5
Mayonnaise	1 Tablespoon	90	10
Light mayonnaise	1 Tablespoon	35	3
Soft (tub) margarine	1 Tablespoon	70	7

Copyright 2017 University of Pittsburgh

Snack List

Feel free to include healthy snacks during the day, if desired. Choosing snacks that are less than 200 calories will help you to stay close to your daily calorie goal.

Snack time is a great time to enjoy veggies, fruit, and foods high in calcium. Snacks with protein and fiber may help you feel fuller longer.

Snacks	Calories	Fat Grams
1 cup fat-free milk with 1 graham cracker sheet/rectangle	150	1
100 calorie pack of almonds	100	9
1 container fat-free Greek yogurt with 1 cup sliced strawberries	140	1
1 sliced apple with 1 Tablespoon peanut butter	180	8
1 low-fat cheese stick or 1 ounce low-fat cheese with ½ cup grapes	140	5
4-ounce fat-free vanilla pudding snack with ½ banana	150	0
¼ cup hummus with raw veggies (carrot, bell peppers, celery)	100	6
8-ounce can low-sodium vegetable or tomato juice with 1 low-fat cheese stick	130	6
½ cup low-fat cottage cheese with ½ cup fresh or canned fruit in juice	140	2
100 calorie bag of microwave popcorn	100	2
2 Tablespoons guacamole with raw veggies (carrot, bell peppers, celery)	50	4.5

Copyright 2017 University of Pittsburgh

Shopping List

Check (✓) the items you need to follow the GLB Healthy Menu Ideas.

Produce

☐ Fresh fruit
☐ _______________
☐ _______________
☐ _______________
☐ Dried fruit
☐ _______________
☐ _______________
☐ Avocado
☐ Salad greens
☐ Potatoes, white
☐ Potatoes, sweet
☐ Raw vegetables
☐ _______________
☐ _______________
☐ _______________

Dairy

☐ Milk/soy milk
☐ Yogurt
☐ Low-fat cheese
☐ Low-fat cottage cheese
☐ _______________

Salad Dressings

☐ Low-fat salad dressing
☐ Vinaigrette dressing
☐ Mayonnaise
☐ _______________

Cereals/Grains

☐ High-fiber cereal
☐ Oatmeal
☐ Whole wheat bread
☐ Dinner rolls
☐ English muffins
☐ Bagels
☐ Pita
☐ Whole grain crackers
☐ Brown rice
☐ Whole grain pasta/couscous
☐ Barley
☐ Quinoa
☐ Tortilla, whole wheat or corn
☐ _______________
☐ _______________
☐ _______________

Frozen Entrées

☐ Light meals (less than 300 calories & 10 fat grams)

☐ Main meals (less than 400 calories & 12 fat grams)

Protein Foods

☐ Chicken/turkey white meat
☐ Lean beef or pork
☐ Fish
☐ Canned tuna or salmon, in water
☐ Eggs or egg substitute
☐ Peanut butter
☐ Walnuts
☐ Sunflower seeds
☐ _______________
☐ _______________

Miscellaneous

☐ Soft (tub) margarine
☐ Vegetable/olive oil
☐ Garlic
☐ Nonstick spray
☐ Jam or Jelly
☐ Canned or frozen fruit
☐ Canned or frozen vegetables
☐ 100% fruit or vegetable juice
☐ _______________
☐ _______________

Copyright 2017 University of Pittsburgh

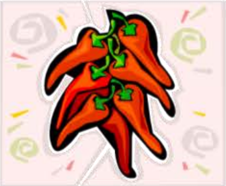

Lentils Ole´

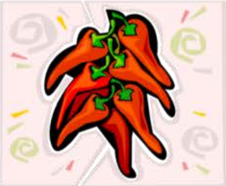

This is one of the best tasting lentil dishes I've ever prepared. I found it in the *Minnesota Heritage Cookbook*, produced in a state not known for haute cuisine but one that harbors quite a number of wonderful cooks.

Serving suggestions: These lentils go well with rice, plain pasta, baked potato or corn bread. Or serve them as a taco filling or on a flour tortilla with chopped fresh vegetables (tomato, lettuce, cucumber, peppers, onion) and shredded cheese for garnish or as the base for tostadas (prepared with softened corn tortillas) with toppings that might include shredded carrots and zucchini, chopped fresh spinach or lettuce, mashed avocado, plain yogurt, salsa, and sunflower seeds.

Preparation tip: This recipe freezers well. It holds up well, and the flavor improves upon reheating.

Lentils Ole´

7 ½ cups water
1 pound lentils
1 Tablespoon olive oil
4 cloves garlic, minced
2 bunches green onions, chopped (including green tops)
2 cups chopped sweet red or green pepper
2 ½ Tablespoons chili powder
1 teaspoon ground cumin
28 ounce can tomato sauce or crushed tomato
2 ½ Tablespoons molasses
2 ½ Tablespoons red-wine vinegar

1. In a large saucepan, bring the water and the lentils to a boil. Reduce the heat to medium-low, cover the pan, and simmer the lentils for 25-30 minutes or until the lentils are tender but not mushy. ***Do not overcook the lentils***. Drain the lentils.

2. While the lentils cook, sauté the garlic, onion and pepper in olive oil in a large pot for about 2 minutes or until the vegetables are tender-crisp.

3. Add the chili powder, cumin, tomato sauce, molasses and red-wine vinegar and simmer.

4. When the lentils are cooked and drained, add the lentils to the vegetable mixture. Heat through before serving it.

½ cup serving = approximately 155 calories, 1.5 grams fat, 7 grams fiber

Source: Jane Brody's Good Food Gourmet 1990

Note: Use no-salt-added tomato sauce or crushed tomatoes to lower the sodium content.

Copyright 2017 University of Pittsburgh

Session 5 Leader Guide:
Tip the Calorie Balance

Objectives
In this session, the participants will:
- Discuss how "calories in" (eating) and "calories out" (being active) are related in terms of calorie balance.
- Review the sources of calories and the calorie value (energy value) of each.
- Discuss how calorie balance relates to weight loss.
- Discuss how healthy eating and being active work together to tip the balance.
- Review their progress so far in terms of changes made to be more active, changes in their eating pattern, and change in weight.
- Discuss additional ways to tip the calorie balance.
- Discuss the purpose and benefits of using the GLB Healthy Menu Ideas or other healthy eating patterns.
- Make a plan to further tip the balance if weight loss is less than expected.
- Develop an activity plan for the upcoming week.

To Do Before the Session:
- If providing Food and Activity books; one per participant.
- Review Food and Activity records from last session; add comments.
- Prepare Session 5 handout for participant notebook.
- Optional: Revise the GLB Healthy Menu Ideas ahead of time if needed to tailor to ethnic or regional eating preferences.
- Optional: Make Lentils Ole and bring to class for participants to taste test.

Available in the GLB Providers Portal:
https://www.diabetesprevention.pitt.edu/my/login.aspx
- **Guidelines for Reviewing Food and Activity Records**

Group Sharing Time (allow at least 5 minutes)

In the last session, we talked about being active. We talked about the two ways to add movement into your day by doing both planned and spontaneous/unstructured activity.

[*You may want to spend a few minutes reviewing the two ways to add movement. One option is to ask participants to define each and explain the differences between them.]*

Let's take a few minutes to talk about your experiences this past week. *[Choose only a few of the following prompts to guide discussion.]*

Copyright 2017 University of Pittsburgh

- Were you able to follow the plan you made to reach your goal for minutes of planned activity during the week? How did it go? What did you learn?
- Were you able to make the active lifestyle choices to increase spontaneous/unstructured activity and to decrease the time you spent sitting? How did it go? What did you learn?
- Did anyone use the “How am I Doing?” Tracking Activity: Year-at-a Glance graph found on page 21 of the Session 4 handout? You can record your activity minutes every week. You can use the graph to see at a glance your progress over time and how you are doing compared to your activity goals. *[Answer any questions about how to use the graph.]*
- Overall, how did it go last week? What went well? What problems did you have? What could you do differently?
- What did you learn by self-monitoring? What did you learn about your overall eating pattern? What did you learn about your food choices?
- What change did you make in your eating pattern this past week that you are most proud of?
- How close did you come to your calorie and fat gram goal?

[Problem solve with the participants to address any barriers.]

[Praise all efforts to self-monitor and to change eating and activity behaviors. Be positive and nonjudgmental. Encourage group discussion.]

Session 5: Tip the Calorie Balance (page 1)

Everything we've covered so far fits together. It fits together because of what's called the "calorie balance." That's what we'll talk about today.

We've said many times that Group Lifestyle Balance involves **two lifestyle changes:**
1. Healthy eating and
2. Being physically active.

Both of these changes are important. They may prevent diabetes and lower your risk of other diseases. They are also important because they're **both related to weight loss** due to what is called "**calorie balance.**"

Calorie balance is the balance between the calories (or energy) you take in by eating and drinking and the calories (or energy) you use by being physically active.

First, let’s talk about “calories in”.

[Review the information on page 1 and include the following information.]

Calories are a unit of measure. We use pounds as the unit of measure for body weight, feet and inches as the unit of measure for height, and miles as the unit of measure to

Copyright 2017 University of Pittsburgh

determine the distance between places. Calories are used to measure the energy value of foods and drinks.

Calories in food come from **fat, carbohydrates (starches, sugar), protein, or alcohol.** Other ingredients in food, like vitamins, minerals, water, and fiber, don't have calories. For example, green leafy vegetables are mostly water, vitamins, minerals, and fiber--so they have very few calories.

The **number of calories in any food you eat depends on what's in that food.** *[Refer to the box on page 1.]*

Fat is the most concentrated in calories, with 9 calories per gram. That's more than twice the number of calories in starches, sugars, or proteins, and even more than alcohol. So, foods that are high in fat are high in calories. Eating less fat is one way to eat fewer calories.

Now let’s talk about “calories out”.

[Review the information in this section and include the following information:]

Your body burns calories even while at rest for just staying alive. You also burn calories during physical activity.

Calorie balance is important for weight management. So be aware of what you eat and drink (calories in) and how active you are (calories out).

Calorie Balance: Calories In and Calories Out (page 2)

Your **weight** is determined by the **balance of calories in from what you eat and drink and calories out from moving more (both planned and spontaneous activity).**
Let's look at four ways the calorie balance can work. [*Refer participants to the 4 diagrams on page 2. Include the following information:]*

1. Your weight can **stay about the same**. In this case, "calories in" from food equal "calories out" from activity. Food and activity are at about the same level on both sides of the scale.

2. Second, you can **gain weight**. In this case, "calories in" from food are higher than "calories out" from activity. Either calories have increased or activity has decreased or both. The balance has tipped this way *[indicate direction of balance]*.
3. Third, you can **lose weight.** "Calories in" from food are less than "calories out" from activity. You’ve eaten fewer calories or you’ve done more activity, or both. The best way to lose weight is to do **both** at the same time to **tip the balance** this way *[indicate direction]*.

4. And finally, you can reach a **new balance at a new lower, healthier weight. During the GLB program, y**ou will create a healthier lifestyle with new eating and activity behaviors that keep your "calories in" and "calories out" in balance. We will work together to help you **make the changes part of your lifestyle, so you will keep the weight off.** This is what happens when you lose weight and keep it off. You've reached a new balance at a lower weight.

The important things to **remember are** that:

- **Eating and physical activity work together** to determine how much you weigh.
- To lose weight, it's **best to eat fewer calories *and* be more active.**
  That way, you are changing both sides of the energy balance at once.

TIPPING the balance will help you lose weight and improve your health.

Healthy Eating and Being Active Work Together (page 3)

[Review page 3 and include the following information:]

How much does it take to tip the balance?

The number of calories you need to eat, or the amount of activity you need to do, varies from person to person.

In DPP the participants "tipped the balance" by finding ways to eat about 500 fewer calories per day than they had been. This resulted in a weight loss of about 1 pound per week. [*Refer to the box at the bottom of the page. Emphasize that research shows that this varies from person to person, so is only a rough estimate.]*

Your GLB calorie and fat gram goals were set up to help you "tip the balance" enough to create a s**low, steady weight loss (about 1 to 2 pounds or so a week. No one loses the same exact amount of weight each week.). This the best way to lose weight.**

Quick losses of large amounts of weight can mean that muscle is being lost, which is not healthy. Quick weight loss can be difficult to maintain because it often involves very restricted eating and/or excessive amounts of exercise that can't be sustained for a lifetime. GLB aims to help you make changes in your eating and activity behaviors that you can stick with long term.

Be careful not to fall into the "because I exercised" trap and think that you can overeat and still lose weight.
To summarize how healthy eating and being active work together:

- Body weight is a balance between "calories in" and "calories out".

Copyright 2017 University of Pittsburgh

- Even though there are hundreds of diet books, infomercials, and ads on the internet, there is no "magic bullet" to weight loss. Research shows that all the weight loss programs recommend a way to eat fewer calories.
- For losing weight and improving health, the best way to tip the balance is to change both eating **and** activity.
- Following your GLB calorie goal will lead to about a 1-2 pound weight loss per week. Be consistent. Aim to get close to your calorie goal each day.
- Eat at least 1200 calories per day to have a healthy, well-balanced diet. It is very hard to get all the healthful nutrients you need each day if you're eating less than 1200 calories. Also, a very low calorie level is difficult to maintain. It can also cause you to be overly hungry which can lead to overeating.

Changes You Have Made So Far (page 4)

In GLB we will sometimes pause and give you a chance to think about the changes you have made so far in your eating and activity behaviors. This allows you to celebrate the positive lifestyle changes you have made. It also allows you to think about your focus for the upcoming month. You have been working on creating a healthier lifestyle for about a month, so today is a good time to evaluate your progress.

Let's take a minute now to review some of the **changes you've made so far** on both sides of the balance.

First, **what changes have you made to be more active**? We've talked about increasing both structured/planned activity, the kind you've been recording, and spontaneous/unstructured activity, like taking the stairs instead of an elevator, that add more total movement to your day.

[Give the participants some time to briefly record on page 4 some of the changes they've made. Ask volunteers to share. Praise and encourage the maintenance of these changes.]

Now let's talk about the **changes have you in your eating pattern.** Think about changes you have made to eat fewer calories and to make healthier food choices.

[Again, have participants briefly record some of the changes they've made. Ask volunteers to share. Praise and encourage the maintenance of these changes.]

Lifestyle change is a process. The purpose is to look at progress toward reaching your goals. No one is looking for perfection.

Have these changes tipped the calorie balance?
The scale reflects the changes you are making in your eating and activity behaviors.

Let's look at how the scale has responded.

Copyright 2017 University of Pittsburgh

- In Session 1 you found your 7% weight loss goal. [*Ask participants to refer to their **Weekly Record** and record this goal weight in the box on page 4.]*

- At the **start of GLB, you weighed** ... [*Ask participants to refer to their **Weekly Record** and record their starting weight.]*

- **Your weight today is... [***Ask participants to record their current weight.]*

- Change in weight so far. [*Ask participants to record their weight change during the past month in GLB.]*

[Emphasize that it's important for the participants to focus on their accomplishments so far, no matter how small.]

> Ask participants to check one of the three boxes on page 4.
>
> Encourage attendance at group meetings, even if they have a time when they are struggling. This is when they most need to group support, suggestions, and encouragement.
>
> Express your confidence in the participants' future success.

New Ways to Tip the Balance (pages 5-6)

You've looked at the changes in your eating and activity behaviors and how these changes were reflected on the scale.

Let's talk about some new ways to "tip the balance".

[Review information on page 5. Encourage discussion by asking participants to give feedback on any of the suggestions they have tried such as a local exercise class they enjoyed or their favorite website for low-calorie recipes.]

Be Aware of Liquid Calories:
In Session 3 we discussed the 2015 Dietary Guidelines recommendation to consume less added sugar. One way to decrease sugar intake is to follow the guideline of MyPlate and to drink water instead of sugary drinks.

Have you been experimenting with drinking more water? Have you noticed a difference in how you feel? Water does not have any magical weight loss properties. But it can help with weight loss because people often mistake their body's thirst signal as a hunger signal and overeat. Staying well hydrated can help prevent this from happening.

[After reviewing the information, ask participants to share ways they have reduced the calories they are drinking. Encourage a discussion about limiting sugar-sweetened beverages, alcohol, and/or coffee drinks.]

> Note: Caffeine was not mentioned in the 2010 Dietary Guidelines but it was included in the 2015 guidelines. Cautions were given about mixing alcohol and caffeine, high-caffeine energy drinks, and mixing energy drinks with alcoholic beverages.

Some people find it useful to follow an eating pattern for meals and snacks. These can help to eliminate decisions regarding food choices and simplify the self-monitoring process.

Page 6 lists a variety of healthy eating patterns that you may find useful.

The USDA provides a general healthy eating pattern as well as ones that are specific to Mediterranean-Style and Vegetarian eating.

The MyPlate website offers a general eating pattern but also allows you to create a personalized plan.

The GLB Healthy Menu Ideas are on pages 9-11. *[Ask participants to turn to page 9. Review the benefits of using these menu ideas and suggestions for how to spread calories throughout the day.]*

The GLB Healthy Menu Ideas include 4 sample menus for breakfast, lunch, and dinner.

To help you put together your own meals and to make it easier to self-monitor, there are lists of suggested foods for each meal on pages 12-16. A list of snack ideas is on page 17.

A shopping list and a recipe are included as well on page 18.

[Optional: Taste test Lentils Ole. The recipe is on page 19.]

For those of you who do not want this much structure, I encourage you to look at the menu ideas and to find one or two meals or snacks that you are willing to try. You can share your feedback with the group next week.

Of the ways to further tip the balance that we just discussed, what one or two of these strategies can you see yourself doing? Write them on the bottom of page 6.
[Ask participants to share their answers with the group, if they feel comfortable doing so. Encourage group discussion.]

Are there any additional strategies we haven't discussed that you are using to help tip the balance? [*Encourage group discussion.]*
[*Summarize the key messages of this lesson by reviewing the information in the text box.]*

To Do (page 7)

Turn to page 7 and let's focus on what you can do between now and the next session.
[*Review information on page 7]*

As you have been doing:

Copyright 2017 University of Pittsburgh

- Please continue to **keep track** of your weight and record it.
- Also continue to measure and record everything you eat and drink every day. Come as close as you can to your calorie and fat gram goals.

Let's look at the "new things to practice".

Make a change in eating or activity to "tip the balance". Look at the suggestions on pages 5 and 6 and decide what you are willing to try.

One choice may be to lower your calorie goals. You may be eating more calories and fat than you realize, or your goals may simply be too high for you. Everyone is different. The way to know whether your goals are right for you is whether or not you are losing 1-2 pounds per week on average. Lower your calorie goal by 300 calories per day. Refer back to Session 2, page 2 for the calorie and fat gram goals used in Group Lifestyle Balance (1200 calories/33 grams of fat; 1500 calories/42 grams of fat; 1800 calories/50 grams of fat; 2000 calories/55 grams of fat). It is important that you do NOT go below 1200 calories per day.

Or you may choose to follow one of the healthy eating patterns listed on page 6 in today's handout, try a new exercise class, or experiment with ways to limit your liquid calories.

Whatever change you make to "tip the balance", write it down. You can give the group feedback about your efforts at the next meeting.

Let's talk about your activity goal for this coming week.
The suggested activity goal for last week was 60 minutes.

- If you reached 60 minutes, try adding 30 minutes this week. Your new activity goal of **90 minutes.** *[Note: This is the standard GLB exercise progression. Many participants will be able to comply. However, flexibility is included based on health or other barriers to exercise.]*
- If you were active for less than 60 minutes last week, that's okay. Start at your current activity level and try adding 30 minutes more this week.
- If you were already doing more than 60 minutes of activity, great work. Keep it up.

[Note: You should make exceptions for individuals with serious health problems that limit their mobility. For them, they should start where they are and gradually increase as much as they can.]

As before, make a plan to be active this week. Remember how important it is to make a plan for activity…it doesn't just happen.
[Give participants time to complete the chart on page 7.]

Make active lifestyle choices throughout the day. As we've discussed, every minute of movement is helpful. Standing uses more calories than sitting; walking uses more calories than standing; and so on. So, keep moving as much as you can.

Copyright 2017 University of Pittsburgh

What are some of the active choices you plan to make? Jot them down on the bottom of page 7.

For the rest of the program, we'll keep working together to bring you closer to your goals for healthy eating, being active, and losing weight. **We'll keep trying to tip the calorie balance and see how the scales respond.** Over time, you'll reach a new balance at your goal weight and then we'll work together to help you maintain that weight.

Resources (pages 8-19)

[Participants were directed to these pages when discussing page 6. Encourage participants to read these pages at home.]

[Announce the day, time, and place for the next session.]

After the session:

- Weigh participants who did not do so prior to the group meeting.
- Complete data forms and documentation required in your setting.
- Follow your program's protocol for managing absences.
- Review the self-monitoring records from the previous week. Write brief comments. Be positive and nonjudgmental.
    - Praise all efforts to self-monitor and to change eating and activity behaviors.
    - Highlight especially any positive changes made that relate to the session topic of the week before the records were collected: recording minutes of physical activity, being active on several days, and coming close to their calorie, fat gram, and activity goals.
    - Refer to **Guidelines for Reviewing Food and Activity Records** available in the GLB Providers Portal.

Copyright 2017 University of Pittsburgh

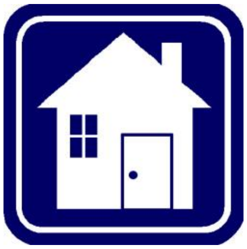

Session 6: Take Charge of What's Around You

The first step to a healthier lifestyle is to look closely at your day-to-day routines. To take charge, learn the “cues” (or “triggers”) for how you eat and how you move.

What "cues" you (or makes you want) to eat?

- Hunger
- What you're thinking or feeling
- What other people say and do
- Sight and smell of food
- Certain activities that make you think about food, like watching TV or reading magazines

Examples:

**“Cue”**	Makes You Want to Eat This
You see a carton of ice cream.	Ice cream 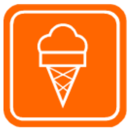
You turn on the TV. 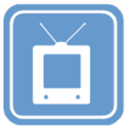	Potato chips
You go to the movies.	Popcorn 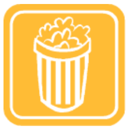

When you respond to a food cue in the same way, over and over again, you build a habit.

How can you change problem food cues and habits?

1. Stay away from the cue. Or keep it out of sight.
2. Build a new, healthier habit.
   - Practice responding to the cue in a healthier way.
   - Add a new cue that helps you lead a healthier life.

Keep in mind: It takes time to break an old habit and build a new one.

Copyright 2017 University of Pittsburgh

Common Problem Food Cues

At home

- Living room: TV, computer, phone, candy dishes
- Kitchen: Ready-to-eat foods (ice cream, chips, cookies), preparing food, putting away leftovers
- Dining room: Serving dishes on table, large dinner plates, long amount of time spent at the table

Away from home

- Commuting: Bakeries, coffee shops, fast food drive-thru windows
- Public areas: High-calorie foods and beverages (doughnuts, pastries, candy) in cafeteria, vending machines
- Workplaces: Candy and snack foods always in view

My problem food cues: ____________________________________

Tips

- Keep problem foods out of your house and work place. Or keep them out of sight. Out of sight is out of mind.

- Keep healthy choices easy to reach, in sight, and ready to eat. Examples: Fresh fruits, raw vegetables (already washed and prepared), water, and other calorie-free drinks.

- Limit your eating to one place (or fewer places).

- Limit other activities when you are eating.

Shopping Tips

- Become an expert on where you shop (name those places): ____
- Plan meals and make a shopping list ahead of time. *Stick to the list*.
- Don't go shopping when you're hungry.
- Avoid sections in the store that are a problem for you.
- Buying in bulk can help your budget but be sure to have a plan for portion control.
- Ask the store manager to order healthy food you want.
- Don't let coupons tempt you to buy junk food.

Copyright 2017 University of Pittsburgh

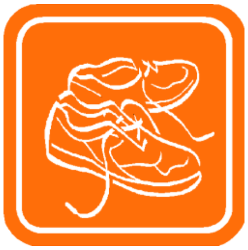

Common Activity Cues

Now look at your cues for moving and sitting.

1. Add positive activity cues to your life.

- Keep these in sight:
  - Equipment such as shoes, bag, mat, bike
  - Self-monitoring record, exercise calendar
  - Exercise video
  - Motivational photos, posters, magazines
- Set up a regular "activity date" with a friend or family member.
- Set a timer or alarm on your watch or other device to remind you to be active.
- My positive activity cues: ___________________________________

2. Decrease your cues for being inactive.

- Limit TV watching. Or be active while you watch TV.
- Limit any form of screen time (such as videos, games, shopping, and social media).
- Limit "over scheduling". Having too much on your calendar makes it hard to find time to be active.
- My cues for being inactive:__________________________________

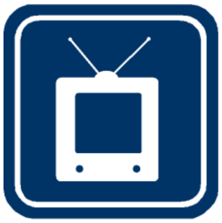

3. Learn to use feelings of being low on energy or stressed as cues for being active.

- Try building a new habit. Go for a walk instead of watching TV when feeling stressed, bored, or sluggish.

Copyright 2017 University of Pittsburgh

To Do:

Check the boxes when you complete each item:

☐ Keep track of your weight. Weigh yourself at home at least once a week. Record it.

☐ Record everything you eat and drink every day. Come as close as you can to your calorie and fat gram goals.

New Things to Practice:

Make food and activity cues work for you, not against you.

☐ Get rid of one problem food cue.

What problem food cue will you get rid of? ____________________

What will you need to do to get rid of it?

____

What problems might you have? What will you do to solve them?

____

☐ Add one positive cue for being more active.

What activity cue will you add? _____________________________

What will you need to do to add it?

____

What problems might you have? What will you do to solve them?

____

☐ Be active for _______ minutes this week. Record what you do.

- The suggested activity goal for last week was 90 minutes.
- If you reached 90 minutes, try adding 30 minutes this week.
- If you were active for less than 90 minutes, that's okay. Start at your current activity level and try adding 30 minutes more.
- If you are doing more, great work and keep it up.

Copyright 2017 University of Pittsburgh

Look for blocks of time when you could be active. Have these blocks of time last for 10 minutes or more. Plan activities you LIKE to do.

	What You Will Do	When	Minutes
Monday			
Tuesday			
Wednesday			
Thursday			
Friday			
Saturday			
Sunday			
Total minutes for the week (My goal: ___ minutes)			

☐ Make active choices throughout the day. Name some of the ways you increased your spontaneous activity.

____

☐ Before the next session, answer these questions:

- Were you able to change any food or activity cues?

  ___Yes   ___No   ___ Almost

- What problems did you have?

  ____

- What could you do differently?

  ____

  ____

Copyright 2017 University of Pittsburgh

Session 6 Leader Guide:
Take Charge of What's Around You

Objectives
In this session, the participants will:
- Learn about common food and activity cues and ways to change them to make it more likely that they will reach their lifestyle goals.
- Mentally search their homes, work places, and food shopping places, looking for problem food cues and discussing ways to change them.
- Learn ways to add positive cues for activity and limit cues for inactivity.

To Do Before the Session:
- If providing Food and Activity books; one per participant.
- Review Food and Activity records from last session; with comments.
- Prepare Session 6 handout for participant notebook.

Available in the GLB Providers Portal:
https://www.diabetesprevention.pitt.edu/my/login.aspx
- **Guidelines for Reviewing Food and Activity Records**

Group Sharing Time (allow at least 5 minutes)

In the last session, you learned that your weight is, in part, the result of the balance between "calories in" from what you eat and drink and "calories out" from moving more. We talked about how the best way to "tip the balance" for weight loss is to eat and drink less and be more physically active.

Let's take a few minutes to talk about your experiences this past week. *[Choose only a few of the following prompts to guide discussion.]*

- Overall, how did it go last week? What went well? What problems did you have? What could you do differently?
- What change did you make in your eating pattern this past week that you are most proud of?
- How close did you come to your calorie and fat gram goal?
- Were you able to follow your plan to reach your goal for minutes of planned activity during the week? How did it go? What did you learn? Were you able to

Copyright 2017 University of Pittsburgh

make active lifestyle choices to increase spontaneous/unstructured activity and to decrease the time you spent sitting? How did it go? What did you learn?

- Did you try something new in order to “tip the balance”? How did it go? What did you learn?
- Did you try a meal or snack from the GLB Meal Plans? If so, please share your feedback with the group.
- Did anyone look at any of the websites for additional meal plans? If so, please share you feedback with the group.

[Problem solve with the participants to address any barriers.]

[Praise all efforts to self-monitor and to change eating and activity behaviors. Be positive and nonjudgmental. Encourage group discussion.]

Session 6: Take Charge of What’s Around You (page 1)

[Do not distribute the Session 6 handout yet.]

Today we're going to talk about how to **take charge of what's around you**, or **how to make what's around you support your GLB goals** to lose weight by eating healthfully and being more active.

First, we'll talk about cues for eating, and later, go on to activity cues.

- **What "cues" you (or makes you want) to eat?** *[Optional: Write responses on the board.] Be sure to note when “hunger” is mentioned.* Of course, one reason we eat is because of **hunger** and learning how to respond to hunger cues in a healthy way is one of the goals of the program**.** But what about those times when you have an “appetite” or desire to eat without physically being hungry?
- You might eat because of **what you're thinking or feeling**. For example, you might eat some ice cream because you feel lonely, bored, or happy.
- You might eat because of **what other people say and do.** You might eat chips at a party because a friend offers them to you.
- Or you might eat because of **the sight or smell of food.**
- **Certain activities that make you think about food** (like watching TV or reading magazines). This is what we'll focus on today. In later meetings, we'll talk about eating in response to thoughts, feelings, or what other people say and do.

[Distribute the Session 6 handout. Review page 1 and include the following information:]

Look at the “cues” listed on page 1. How does our list compare?

Copyright 2017 University of Pittsburgh

When people list their food cues, hunger is often mentioned last. *[Point out where "hunger" is on the list the group generated.]* The goal is to have hunger move to the top of the list, as shown on page 1. We will help you notice and respond more to the cues that come from your body and less to those from the environment. That is what it means to "take charge of what's around you".

Please look at the box for some examples.

- The **sight of food** is one of the most powerful food cues. For example, you may see a carton of ice cream in the freezer and soon you'll be eating ice cream, even though you're not hungry.
- The **activity of watching TV** is also a powerful food cue for many people. You may turn on the TV and find yourself eating potato chips, even though you're not hungry.
- Another example is **eating popcorn at the movies.** Do you eat popcorn when you go to the movies?

If not, probe for another example that is relevant for the participants, such as eating hot dogs or nachos at a sporting event or buying cookies after passing a bakery. Use the example in the discussions that follow.

Why do you eat popcorn in that situation? Do you think it's because you're hungry? Most likely, it's because eating popcorn at the movies is a **habit** for you.

When you respond to a food cue in the same way over and over again, you build a habit. The food cue becomes paired with the way you respond, and your response becomes more and more automatic.

Let's say that since childhood, you've gone to the movies many, many times, and you've eaten many boxes of popcorn there. Now you find yourself eating popcorn whenever you go to the movies, even though you're not hungry. You responded to the cue (going to the movies) in the same way (buying popcorn), over and over again. Buying popcorn became a habit. And since it's a habit, it may be hard for you to sit through a movie and not have popcorn.

Food cues and eating habits are not harmful by themselves. But they can be a problem if they get in the way of your efforts to eat fewer calories. Food cues may also lead you to make unhealthy food choices or eat when you're not physically hungry.

How can you change problem food cues and habits?

1. One of the best things you can do is to **stay away from the food cue. Or keep it out of sight.** For example, you may not be willing to stop going to the movies, but you can stay away from the concession stand. If you keep going to the movies and don't let yourself have popcorn, slowly you will stop thinking about popcorn. The connection between the movies and the popcorn will have been broken.

Copyright 2017 University of Pittsburgh

2. Or you can **build a new, healthier habit. Practice responding to the cue in a healthier way.** An excellent way to support yourself as you do this is to **add a cue that helps you lead a healthier life.** For example, you might take a package of sugar-free gum with you when you go to the movies. When you enter the theater, take out a piece of gum. After a while, you will connect going to the movies with chewing gum.

It's important to remember that **it takes time to break an old habit or build a new one.**

Change doesn't happen overnight. If you wanted to stop eating popcorn at the movies, you would need to see a lot of movies without popcorn. Eventually, you will enjoy the movie and forget about the popcorn.

Note: Some participants may need more examples to grasp the idea that cues in the environment make them want to eat certain things, or that they can learn to manage these cues. Find some ways to show your participants that there are many food cues around all the time and that this phenomenon is so common that we are usually unaware of how powerful it is.

These ideas are powerful, and they work. Also, they're nothing new. People use them every day. Some examples:

- Food companies deliver samples of new breakfast cereals right to your door by mail. They know that if they can get the food into your house, you'll eat it and be more likely to buy it.
- Food stores put new products on the shelves that are the easiest to see and reach. They also put out samples to taste.

For generations, mothers have put leftovers in the front of the refrigerator so their children are more likely to eat them.

In this session, we want to help you learn to make changes in what's around you to encourage healthy eating and being more active.

Common Problem Food Cues (page 2)

[Note: A few common food cues are listed at the top of page 2. Do not turn to this yet.]

Let's talk about **some of the problem food cues in your life and some ways you can change them.**

Let's start with **where you live.** Imagine that we've just opened the front door. A film crew is recording what's in the room. Which room would it be? Do you see any actual food in the room? Do you see anything else that might make you think about eating, like

Copyright 2017 University of Pittsburgh

a TV or a comfortable chair? What is a change you could make to stay away from that cue or to build a new, healthier habit?

**Move from room to room** ("Are there other rooms that are a problem for you?"), asking for cues and discussing possible ways to either stay away from the cue or to build a new, healthier habit. If the participants have no response, ask them to refer to their previous Food and Activity records and/or turn page 2 of the handout to the list of common problem food cues and ask if one or two of the examples apply to them (possible solutions are given below). Use just a few examples, and make sure they are relevant to your group. Food cues will come up at future sessions and can be addressed in detail at that time.

[Review the "At home" section. Include the following information if not already discussed in your group.]

Living Room

Cue: *TV (or computer, telephone).*

Solution(s): One way to break the connection between eating and the TV is to make it a rule for yourself not to eat in front of the TV (or while on the computer or phone).
Keep an exercise bike, exercise mat, or other equipment nearby.
Keep a pack of sugar-free gum nearby. Allow yourself only calorie-free beverages, not food.

Cue: *Candy dishes (for serving candy, chips, and nuts) on an end table.*

Solution(s): Don't buy candy, chips, or nuts. If you do buy these foods, store them out of sight.

Kitchen

Cue: *High-calorie foods,* ***especially those that are ready to eat*** *(*in the freezer (e.g., ice cream), refrigerator (e.g., cheese, lunch meats, pie), kitchen cupboards (e.g., cookies, chips), or on counter tops (e.g., cookie jar, food packages).

Solution(s): Stop bringing these foods into your home altogether. Store them out of sight, in a brown bag or other opaque container.
Make them hard to reach. Store on highest shelf or in the basement.
Keep healthy food choices easy to reach, in sight, and ready to eat.

Examples: Fresh fruits, raw vegetables (already washed and prepared), pretzels, low-fat popcorn.
Limit high-calorie choices to those that require preparation.

Copyright 2017 University of Pittsburgh

Cue: *Foods you are cooking or leftovers, on the stove or counter.*

Solution(s): Make it a rule not to eat while cooking. Taste foods only once, then drink water or a take a breath mint immediately.
Ask someone else to taste the food.
Rinse off any utensils used in food preparation immediately after each use.
Put leftovers away **before** meals.
Ask someone else to put the leftovers away.
Put leftovers in individual serving containers right away, and freeze them for future meals.

Dinner Table

Cue: *Serving dishes on table.*

Solution: Serve foods from the kitchen.
Store food only in the kitchen. Put packages away immediately after use.

Cue: *Large dinner plates (or large glasses, bowls, serving spoons and forks).*

Solution(s): Serve yourself small portions using a smaller plate or bowl. Or ask someone who is supportive to do so. Spread the food attractively over the plate.

Cue: *Leftovers on plates.*

Solution(s): Remove your plate from the table as soon as you're finished.
Don't eat the food that your children leave on their plates.

Let's do the same thing with when you are **away from home**. Are there any things you encounter while commuting, in public areas, or at your workplace that have become paired with eating high calorie foods? What can you do to change these problem food cues? *[Review the "Away from home" section. Include the following information. Encourage group discussion.]*

Cue: *Bakeries, coffee shops, food restaurants on your commute.*

Solution(s): Take a different way.
Make it a rule not to eat in the car.

Cue: *High-calorie foods in public areas* (doughnuts, pastries or candy in the cafeteria or in vending machines).

Solution(s): Stay away from those areas.
Bring a low-calorie snack to share with others.
See if there's a way to keep these foods out of sight (other people may appreciate it, too).

Copyright 2017 University of Pittsburgh

Cue: *Vending machines.*

Solution(s): Stay away from the vending machines.
Bring a low-calorie snack from home. Or buy juice or pretzels, if available in the machine. Ask a friend to go get them for you, so you won't be tempted by the other foods.

Cue: *High-calorie foods always in view in the workplace.*

Solution(s): Don't bring high-calorie foods to work. Keep low-calorie snacks like apples, raw carrots, pretzels, low-fat popcorn, or calorie-free beverages on hand instead.
Make it a rule not to eat at your desk.

Please turn to page 2 of your handout. At the top of the page you'll see common problem food cues we just discussed.

Please take a minute think of your problem food cue(s). Write it on the line.

Look at the "Tips" box. It lists four strategies to manage food cues to help you take charge of your eating environment. The important things to **remember**, whether you are at home or away from home are:

[Review the information in the box on page 2. Include the following information:]

- **Limit your eating to one place.** Where do you eat most of your meals at home? Limit all eating to this place. When you are hungry, go to this place to eat. This will help you to distinguish between hunger and other cues to eat.

  At work, a particular table in the cafeteria or kitchen area may be a good choice. Do not eat at your desk or computer. This is an open invitation to become distracted from eating.

- **When you eat, limit other activities**. The rule is simple: No TV, driving, or talking on the phone while you are eating. Focus on enjoying the meal. In the future, these other activities will not cue you to eat.

Finally, let's take the film crew to **where you shop for food.** Walk around the store as you usually do. What do you see that's a problem for you?

The grocery store can be a "high risk" situation because of the sheer number of cues. Your shopping habits are directly linked to the foods that end up in your home (and in your stomach). But you do have some control and you can learn to become a healthier shopper. Let's look at the "Shopping Tips" that are listed at the bottom of the page.

Copyright 2017 University of Pittsburgh

[Review information and include the following information:]

- **Become an expert on where you shop.** Know the best places (including farmers markets) that fit your budget, routine, and healthy eating goals.
- **Plan meals and make a shopping list ahead of time.** Make it a rule not to buy anything that's not on the list.
- **Don't go shopping when you're hungry.** Have a low-calorie meal or snack first.
- **Avoid sections in the store that are tempting** to you, if possible. For example, walk down a different aisle to avoid the bakery.
- **Buying in bulk:** Some people make individual serving packets as soon as they get home. What are some ideas for portion control?
- **Ask the grocery store manager to order healthy foods t**hat you want to buy. You are the customer and it is their business to please you.
- **Don't be a slave to coupons.** Only use the coupons that are for healthy foods, paper products, and cleaning/laundry supplies.

Common Activity Cues (page 3)

Now let's turn to cues for **moving and sitting**. For most people, there are many things around them that lead to being inactive. For example, after dinner, you may automatically sit down in front of the TV. This is because the end of dinner and TV have been paired together many times in the past. But remember, you do have a choice. You could just as easily choose to take a 15- minute walk after dinner.

If you have been inactive, you probably have many cues around you that are associated with inactivity and few that would cue you to be active. To be active regularly, it's important to add positive activity cues to your life. Over time, the cues will become paired with being active, and you will develop new activity habits that will become more and more automatic.

1. **What are some positive activity cues that you could add to your life?** Let's pick up our imaginary film crew again, and start with **where you live.** What could you add to the living room that would prompt you to be active?

Move from **room to room**. Mention a few examples from the handout and add some that are particularly relevant to the participants.

In the living room or bedroom:

- Keep exercise shoes, bag, and equipment in sight, not in the closet.
- Keep home exercise videos handy (training apps, channels, DVDs).
- Hang an activity calendar and graph of your activity in a visible place.
- Keep a stationary bicycle or exercise mat in front of the TV. Now TV will become a cue for moving not sitting.

Copyright 2017 University of Pittsburgh

- Hang a photo or poster of people being active or of outdoor scenes in a visible place.
- Subscribe to a health or exercise magazine. Keep it in a visible place.
- Put a note on the TV reminding you that a half hour of TV time could be used for a walk instead.

In the kitchen:
- Post motivational photos, outdoor scenes, or reminders to be active on the refrigerator.

In the bathroom:
- Post a reminder on the mirror to be active.

In the garage:
- Keep the car in the garage. Maybe you'll choose to walk to the neighbor's house, rather than get the car out to drive short distances.
- Keep exercise shoes, bag, and equipment on the front seat of your car.
- Keep a bicycle in working condition. Store it along with a helmet in a visible place and ready to ride.

We've talked about some ideas of how to add positive cues for activity at home. What are some positive cues for activity that you could add to your work place?

Let's pick up our imaginary film crew again. *[Encourage group discussion. Examples include:]*
- Put your walking shoes in a visible place in your office.
- Put a note on your office door or computer reminding yourself to take a walk during your lunch break before eating.
- Set an alarm on your watch or other device to remind you to take a walk.
- Make a regular, daily appointment with yourself to be active. Put it in your calendar. Keep your appointments with yourself--they are as important as your other appointments.

Look at the positive cues listed on page 3. *[Review and include the following information:]*
- Set up a **regular "activity date" to be active with a friend or family member.** When she or he arrives at 7:00 for a walk, you'll probably go even if you don't feel very energetic.

What are some other ideas?

2. **Manage your cues for being inactive.** Another approach is to remove the cues for being inactive. *[Review and include the following information:]*
   - **Limit TV watching.** Keep the TV behind closed doors in a cabinet. Or get rid of your TV. Or be active while you watch TV (for example, ride an exercise bike or

Copyright 2017 University of Pittsburgh

get up and move during commercials.). What are some things that you do during TV commercials in order to break up long periods of sitting?

- **Don't pile things at the bottom of the stairs that need to go upstairs.** They remind you to keep leaving more things there, rather than climbing the stairs. Take separate trips upstairs instead.

What are some other ideas?

3. **Learn to use feelings of being low on energy, sluggish, or stressed as cues for being active.** People often become inactive in response to feeling tired or stressed. Experiment with being active instead. For example, go for a walk instead of laying on the couch and watching TV. Try it this week and see how it goes. If you continue to choose to be active in response to these feelings, you will build a new habit.

In summary:

- It takes time to break old habits and build new, healthier ones, but it can be done.
- Be patient with yourself and keep experimenting with ways to take charge of what's around you.
- One of the most important steps you can take is to get rid of problem cues and add new ones that will help create an environment that will support healthy eating and being active.
- **You can make food and activity cues work FOR you, not against you. That's what it means to "take charge".**

To Do (pages 4-5)

Turn to page 4 and let's focus on what you can do between now and the next session.

Please continue to keep track of your weight and eating. Come as close as you can to your calorie and fat gram goals.

To help you practice what you learned today, focus on ways to make food and activity cues work for you, not against you.

First, **get rid of one problem food cue** in your life. Let's make a plan for that. *(Ask the participants the questions on page 4 and have them fill in the blanks).*

Also, **add one positive cue for being more active.** *(Ask the participants the questions on and have them fill in the blanks).*

There is a section on page 5 where you can write how things went with your plan for food and activity cues. You can share your experience/give feedback on this next week.

Copyright 2017 University of Pittsburgh

Let's talk about your activity goal for this coming week.

- If your goal for last week was 90 minutes, your goal is to add 30 minutes for a new activity goal of **120 minutes.** *[Note: This is the standard GLB exercise progression. Many participants will be able to comply. However, flexibility is included based on health or other barriers to exercise.]*
- If you were active for less than 90 minutes last week, that's okay. Start at your current activity level and try adding 30 minutes more this week.
- If you were already doing more than 90 minutes of activity, great work. Keep it up.

[Note: You should make exceptions for individuals with serious health problems that limit their mobility. For them, they should start where they are and gradually increase as much as they can.]

As before, make a plan to be active this week. Remember how important it is to make a plan for activity…it doesn't just happen.

[Give participants time to complete the chart on page 5.]

[Announce the day, time, and place for the next session.]

After the session:

- Weigh participants who did not do so prior to the group meeting.
- Complete data forms and documentation required in your setting.
- Follow your program's protocol for managing absences.
- Review the self-monitoring records from the previous week. Write brief comments. Be positive and nonjudgmental.
    - Praise all efforts to self-monitor and to change eating and activity behaviors.
    - Highlight especially any positive changes made that relate to the session topic of the week before the records were collected: any changes in eating and activity to help to "tip the balance", experimenting with use of all or part of meal plans, recording minutes of physical activity, being active on several days, and coming close to their calorie, fat gram, and activity goals.
    - Refer to **Guidelines for Reviewing Food and Activity Records** available in the GLB Providers Portal.

Copyright 2017 University of Pittsburgh

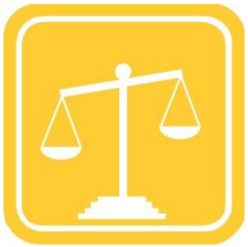

Session 7: Problem Solving

Many things can get in the way of being more active and achieving your healthy eating, physical activity and weight goals. But problems can be solved.

The five steps to problem solving:

1. Describe the problem *in detail*, as a chain of actions.

- Give a specific example of one time you have had that problem.
- Try to **find "the action chain" —** a series of actions (or "links") that led up to the problem. Look for:
  - Things that "cued" you (made you want) to eat or be inactive.
  - People who didn't support you.
  - Thoughts or feelings that got in your way.

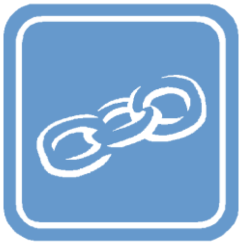

Example:

- Sarah says, "I'm a mess. I seem to end up eating sweets every afternoon. And then I don't care about my food choices the rest of the day."
- A specific example: Last Saturday afternoon she ate a lot of cookies.

Sarah's action chain:

- Skipped lunch while running errands. Traffic was bad.
- Said to herself, "I have no energy to go for a walk today". Came home tired, stressed, grumpy and hungry.
- Went right to the kitchen.
- Talked with her son on the phone. He was critical, which upset her.
- Saw cookies on counter.
- Ate cookies.

Copyright 2017 University of Pittsburgh

2. Brainstorm your options for each link in the chain.

Links in<br>**Sarah's Action Chain**	Some of Sarah's Options
Skipped lunch while running errands. Traffic was bad.	• Never run errands again. (In our dreams....)<br>• Pack a healthy snack.
Skipped her walk. Came home tired, stressed, grumpy and hungry.	• Go for a walk to unwind.
Talked with her son on the phone. The conversation upset her.	• Take a few deep breaths.<br>• Remind herself not to take what he said personally.<br>• Try to avoid difficult conversations when she's tired, stressed, grumpy or hungry.
Went into the kitchen. Saw cookies on the counter.	• Avoid the kitchen. Go out in the yard or sit in a favorite chair for a few minutes to calm down.<br>• Don't buy cookies.<br>• Keep cookies out of sight.<br>• Keep fruit in sight.

3. Pick one option to try.

- Weigh the pros and cons.
- Choose one that is very likely to work and that you can do.
- Try to break as many links as you can, as early in the chain as you can.

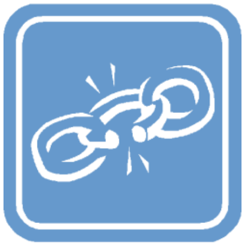

Let's say Sarah chose to pack a healthy snack.

Copyright 2017 University of Pittsburgh

4. Make a positive action plan.

Example for Sarah:

She will:	• Pack a 200-calorie snack.
When?	• The next time she runs errands.
She will do this first:	• Shop for the healthy snack foods.<br>• Put one in her purse.
Roadblocks that might come up:	• Might forget to pack her snack.
How she'll handle them:	• Will go to a healthy quick-serve place.<br>• Will buy a light and healthy meal or snack.
She will do this to make her success more likely:	• Brainstorm with her group about healthy snacks that work in these situations and fit her lifestyle.

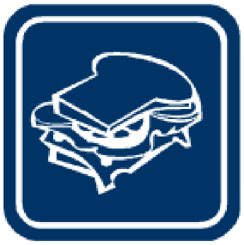

5. Try it. See how it goes.

Did it work? If not, what went wrong? Problem solve again.

Problem solving is a process. Don't give up!

Copyright 2017 University of Pittsburgh

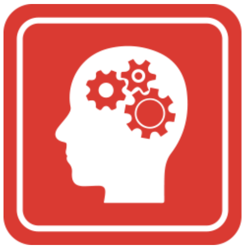

Practice:
Help Sam Solve His Problem

To practice problem solving, help Sam (this page) or Sadie (next page).

Sam's problem: Nighttime snacking

Sam has been working hard to lose weight. He has lost 7 pounds since Session 1. He takes a brisk 40-minute walk, three or four times a week.

At today's session, he told the group that he's frustrated. "Here's one of my biggest problems," he said:

- I eat pretty healthy most of the time, and I feel good.
- But for the last couple of nights, I've been back into my old habit of snacking at night. I'm worried I won't be able to stop.
- I eat dinner around 6. By 8:30 I'm prowling around in the kitchen, looking in the cupboards and the refrigerator. Sometimes I go back to look two or three times.
- The more I try not to do this, the more I seem to want SOMETHING.
- If I just let myself go, I can end up eating over 600 calories.
- It's really discouraging. I want to stop but I'm not sure I can.

Help Sam solve his problem. Turn to the attached worksheets.

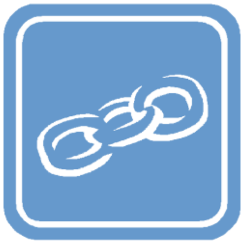

1. In the left column of the Problem Solver for Sam or Sadie, **write down some of the important "links"** in the chain of Sam's day. You can "make believe" and fill in details from your own life. The goal is to spell out more about what might be going on with Sam.

2. In the right column, brainstorm options for each link. What could Sam do differently along the way? The more ideas you have for each link, the better. Be creative and have fun.

3. Choose one option. Fill in the blanks on the Make a Positive Action Plan for Sam or Sadie worksheet.

Copyright 2017 University of Pittsburgh

Practice:
Help Sadie Solve Her Problem

Sadie's problem: Too tired to exercise

On most weeks, she walks for about 20 minutes on the weekend.

"At least part of the problem," she says, "Is not getting enough exercise."

- I've never liked to exercise, even when I was young.
- One of the reasons I joined GLB was to get help fitting in exercise. I know I need to be more active. But it's a real struggle for me.
- It's not that I do nothing. I'm always busy! I spend a lot of time visiting friends and family. I love to cook—it seems like I'm cooking all the time. And I work part-time and do a couple of volunteer jobs too.
- At the end of a busy day, I'm bushed. I get home and fix dinner and then have a glass of wine to relax. The idea of going back outside to take even a short walk is not very appealing. I like to read after dinner too because it's a quiet time just for me.
- This is a long-standing pattern. But I really do want to find a way to fit in more exercise.

Help Sadie solve her problem. Turn to the attached worksheets.

1. In the left column of the Problem Solver for Sam or Sadie, write down **some of the important "links"** in the chain of Sadie's day. You can "make believe" and fill in details from your own life experiences. The goal is to spell out more about what might be going on with Sadie.

2. In the right column, brainstorm options for each link. What could Sadie do differently along the way? The more ideas you have for each link, the better. Be creative and have fun.

3. Choose one option. Fill in the blanks on the Make a Positive Action Plan for Sam or Sadie worksheet.

Copyright 2017 University of Pittsburgh

The Lifestyle Balance
Problem Solver for Sam or Sadie

Describe the problem in detail. Be specific.

____

____

____

Find the action chain.<br>Links	Brainstorm your options.<br>Options

Pick one option. Is it very likely to work? Can you do it?

____

____

____

Copyright 2017 University of Pittsburgh

Make a Positive Action Plan for Sam or Sadie

Pretend to be Sam or Sadie

I will:

____

When?

____

I will do this first:

____

Roadblocks that might come up:	I will handle them by:
__________________________	________________________
___________________________	________________________
___________________________	________________________

I will do this to make my success more likely: _________________

____

____

How can I get the support I need? __________________________

____

____

Copyright 2017 University of Pittsburgh

To Do:

Check the boxes when you complete each item:

☐ Keep track of your weight. Weigh yourself at home at least once a week. Record it.

☐ Record everything you eat and drink every day. Come as close as you can to your calorie and fat gram goals.

New things to practice:

☐ Choose a problem of your own that is getting in the way of meeting your weight, calorie, fat gram, and/or activity goals.

☐ Complete the "**My Lifestyle Balance Problem Solver"** and "My **Positive Action Plan"** worksheets (pages 10 and 11). Follow your action plan.

☐ Be active for ______ minutes this week. Record what you do.

- The suggested activity goal for last week was 120 minutes.
- If you reached 120 minutes, try adding 30 minutes this week.
- If you were active for less than 120 minutes, that's okay. Start at your current activity level and try adding 30 minutes more.
- If you are doing more, great work and keep it up.

Look for blocks of time when you could be active. Have these blocks of time last for 10 minutes or more. Plan activities you LIKE to do.

	What I Will Do	When	Minutes
Monday			
Tuesday			
Wednesday			
Thursday			
Friday			
Saturday			
Sunday			
Total minutes for the week (My goal: ___ minutes)			

Copyright 2017 University of Pittsburgh

Session 7: Resources — Page

My Lifestyle Balance Problem Solver & Action Plan 10-11

Copyright 2017 University of Pittsburgh

My Lifestyle Balance Problem Solver

Describe the problem in detail. Be specific.

____

____

____

Find the action chain.<br>Links	Brainstorm your options.<br>Options

Pick one option. Is it very likely to work? Can you do it?

____

____

Copyright 2017 University of Pittsburgh

My Positive Action Plan

I will: ____

When? ____

I will do this first: ____

Roadblocks that might come up:	I will handle them by:
____________________________	____________________________
____________________________	____________________________
____________________________	____________________________

I will do this to make my success more likely: _________________

____

____

How can I get the support I need? ___________________________

____

____

Copyright 2017 University of Pittsburgh

Session 7 Leader Guide: Problem Solving

Objectives
In this session, the participants will:
- Learn the five steps to problem solving.
- Practice the steps using a problem the participants are experiencing now with eating less fat/calories or being more active.

To Do Before the Session:
- If providing Food and Activity books; one per participant.
- Review Food and Activity records from last session; add comments.
- Prepare Session 7 handout for participant notebook.

Available in the GLB Providers Portal:
https://www.diabetesprevention.pitt.edu/my/login.aspx
- **Guidelines for Reviewing Food and Activity Records**

Group Sharing Time (allow at least 5 minutes)

In the last session, you learned how managing food and activity cues can help you to "take charge of what's around you". This will help to support your efforts to make healthy food choices and be physically active.

- Were you able to get rid of one problem food cue? Please share your experience.
- Were you able to add one positive cue for being more active? Please share your experience.
- Did taking charge of your food and activity cues help you create an environment that supported healthy eating and being active?
- Are there additional food and activity cues you would like to focus on this coming week?

Let's take a few minutes to talk about your experiences this past week. *[Choose only a few of the following prompts to guide discussion.]*

- Overall, how did it go last week? What went well: What problems did you have? What could you do differently?
- How close did you come to your calorie and fat gram goal?

Copyright 2017 University of Pittsburgh

- What change did you make in your eating pattern this past week that you are most proud of?
- Were you able to follow your plan to reach your goal for minutes of planned activity during the week? How did it go? What did you learn? Were you able to make the active lifestyle choices to increase spontaneous/unstructured activity and to decrease the time you spent sitting? How did it go? What did you learn?

[Problem solve with the participants to address any barriers.]

[Praise all efforts to self-monitor and to change eating and activity behaviors. Be positive and nonjudgmental. Encourage group discussion.]

Session 7: The 5 Steps to Problem Solving (pages 1-3)

In the first six sessions of GLB, you learned *how* to eat healthy and be more active. Healthy eating and being active will help you lose weight, prevent diabetes, and be healthier in general.

But healthy eating and being more active means changing your habits, and making the changes a permanent part of your lifestyle. Many things can get in the way of changing habits. That's what we'll focus on in the next several sessions. We will discuss:

- Negative thoughts,
- Slips and your reactions to slips (a slip is when you don't follow your eating or activity plan),
- Stress, and
- What people say and do (or "social cues").

All of these things can get in the way of healthy eating and being more active.

What are some examples of things that get in the way for you?

Name several problems that the participants have already discussed at earlier meetings if possible, e.g., you wanted to go out for a walk, but it was too cold or you wanted to eat fewer calories and make healthy food choices, but your family/roommates wanted you to buy potato chips.

It's inevitable that problems like these will come up.

But problems can be solved. Today we're going to talk about the *process* of problem solving. This is the process that we will be working on together throughout the program. If fact, we have been working the problem-solving process to some extent already. Each time you discuss with each other the ways you have handled challenges and brainstormed solutions, this is problem-solving. Now we will talk about the steps to problem-solving in more detail.

Copyright 2017 University of Pittsburgh

[*Distribute Session 7 handout. Review the information and include the following:]*

The five steps to problem solving:

1. The first step is to **describe the problem in detail, as a chain of actions.**

   For example, instead of defining the problem as "I eat more calories than I should," be specific about the kinds of foods you eat that are high in calories - maybe high-calories desserts and candy. Be specific about when you eat them, and describe these situations in detail. For example, you may eat high-calorie desserts when you go to your mother's house and she offers them to you.

   Also, **look at what led up to the problem**. Many problems involve a chain of actions: one action leads to another and then another and eventually this leads to inactivity or overeating. This is called an "**action (or behavior) chain.**"

   Try to see the steps (or "links") in the action chain, including:
   - **Things that "cued" you (made you want) to eat or be inactive.** We've talked about food and activity cues before. Examples are a bakery near where you work, television watching, or a carton of ice cream in your freezer.
   - **People in your life who don't support your efforts** to lose weight, eat a healthy diet, and be more active. Examples are a co-worker who offers you doughnuts every morning, family who insist that you deep-fry chicken rather than baking it, or a spouse who wants you to watch TV in the evening rather than go for a walk.
   - **Thoughts or feelings that get in your way.** Examples are self-defeating thoughts like, "I'll never be disciplined enough to walk every night." Or eating in response to feelings of boredom, stress, loneliness, or anger.

   Here is an example of an action chain. *[Refer to the "Example" and the text box with "Sarah's action chain".]*

   - It may seem complicated to look at a problem in this much detail. But actually, it makes problem solving much, much simpler.
   - You see that the real problem may not be the last step (eating the cookies) but rather all of **the things that led up to it** (like not eating lunch and so on).
   - Uncovering the action chain will help you to **find the "weakest links" in the chain to break.** There's a saying that a chain is only as strong as its weakest link. By naming all of the links in the chain, you will be able to find the weakest ones, the places where you can make a change most easily.

Copyright 2017 University of Pittsburgh

2. Step 2 is to **brainstorm your options for each link in the chain** (page 2). What are all of the possible solutions to the problem? "Brainstorming" means to create a storm of ideas in your brain. Let the ideas pour out, no matter how crazy they may seem. Anything goes. The more ideas the better. And it's actually helpful to include some crazy, extreme ideas because it helps open your mind and stir up your creative juices.

   By brainstorming, **you'll see that you aren't at all helpless and powerless to change your situation.** You have many options. Here are some possible solutions for Sarah. *[Review and discuss the "Links in Sarah's Action Chain" and "Some of Sarah's Options".].*

3. Third, **pick one option to try (page 2).** Weigh the pros and cons of each option, and choose one (or it might be a combination of several) that is **very likely to work** and that **you can do**. In other words, be realistic. You should be confident that you will succeed.

   It's also helpful to try to **break as many links as you can, as *early* as you can** in the chain.

   For example, it will be much easier for Sarah to control her eating in the evening if she eats some lunch and doesn't arrive home hungry. It will be easier for Sarah to avoid eating too many cookies if she doesn't buy the cookies in the first place. Another reason to try to break an action chain as early as possible is that **you will have more links to work with.** If eating lunch doesn't help Sarah and she still arrives home tired, stressed, grumpy, and hungry, she can still choose low-calorie snacks like fruit or yogurt when she gets home.

   Let's say that Sarah chooses the option of packing a healthy snack.

4. Fourth, **make a positive action plan (page 3).** This is where you spell out exactly:
   - What you will do,
   - When you will do it, and
   - What you need to do first.
   - Also, make a plan for any roadblocks that might come up,
   - And build in steps that will make success more likely. For example:
     - Involve someone else.
     - Find ways to make it more fun and enjoyable.
     - Write your plan down and post it on your refrigerator or calendar or add a reminder to your phone or computer.
     - Tell your plan to someone else, so you're committed to following it.
     - Join an exercise class or club so you're more committed.
     - Make a date with someone to go for a walk.

   GLB recommends planning ahead for healthy eating, being active, and now planning ahead for trying the option that will solve the problem. Note that part of

Copyright 2017 University of Pittsburgh

the planning process is to think about any roadblocks that might get in the way and to come up with a couple of back-up plans to deal with them.

Sometimes if you build in a step to get yourself over the first "hump," then everything begins to snowball and the rest is much easier.

For example, here is Sarah's action plan *[Review and discuss Sarah's action plan.]*

5. The fifth step of problem-solving is to **try it. See how it goes.** Did it work? If not, what went wrong? Use what you have learned to problem solve again and make a new action plan.
   - If the first option works, great. If not, keep trying different options until a solution is found.
   - Don't ignore a problem and let it cause frustration.
   - Problem-solving, like lifestyle change, is a process. Don't give up. It often takes many tries to find a solution.
   - Remember your purpose. Why is losing weight and improving health important?

Now let's practice by helping Sam and Sadie solve their healthy lifestyle change problems.

Practice (pages 4-7)

Divide your participants into an even number of groups (or with a partner). Have at least two groups.

Ask half the group to read Sam's problem on page 4. They should work together to complete "The Lifestyle Balance Problem Solver for Sam or Sadie" on page 6 to solve Sam's nighttime snacking problem. They should then complete page 7, "Make a Positive Action Plan for Sam or Sadie".

Ask the other half of the group to read Sadie's problem on page 5. They should work together to complete "The Lifestyle Balance Problem Solver for Sam or Sadie" on page 6 to solve Sadie's problem with not getting enough physical activity. They should then complete page 7, "Make a Positive Action Plan for Sam or Sadie".

Allow time for the small groups/partners to work through the steps.

Bring the entire group back together.

- Ask a participant to read "Sam's problem: Nighttime snacking" on page 4.
- Ask someone from each group who worked on Sam's problem to read their problem solving steps listed on page 6 and his action plan listed on page 7.
- Repeat this process for "Sadie's problem: Too tired to exercise" on page 5.
- Ask for comments/input/feedback from the entire group.

Copyright 2017 University of Pittsburgh

[Note: Usually each group comes up with a different solution for Sam and Sadie. Every problem has many different solutions. The goal is for each participant to find what works best for them.]

To Do (page 8)

Turn to page 8 and let's focus on what you can do between now and the next session.

Please continue to keep track of your weight and eating. Come as close as you can to your calorie and fat gram goals.

New things to practice:

It's normal to have problems in life. It's also normal to run into a problem(s) while working to change your eating and activity behaviors. Remember that problems can be solved.

Life's more challenging problems usually require a lot of trial and error to find solutions that work for you. Keep reminding yourself that each day, you are doing your own personal research to find what works best for you. Don't give up.

Between now and the next session, work on solving a particular food or activity problem. Name a problem that is getting in the way of meeting your weight, calorie, fat gram, and/or activity goals.

Work through the 5 steps to problem solving by using the worksheet on page 10, "My Lifestyle Balance Problem Solver". Then use page 11 to make a positive action plan. *[If time allows, participants can start working on the worksheet on page 10.]*

Follow your plan. You can share your experience/give feedback next week.

Let's talk about your activity goal for this coming week.

- If your goal for last week was 120 minutes, your goal is to add 30 minutes for a new activity goal of **150 minutes.** *[Note: This is the standard GLB exercise progression. Many participants will be able to comply. However, flexibility is included based on health or other barriers to exercise.]*
- If you were active for less than 120 minutes last week, that's okay. Start at your current activity level and try adding 30 minutes more this week.
- If you were already doing more than 120 minutes of activity, great work. Keep it up.

[Note: You should make exceptions for individuals with serious health problems that limit their mobility. For them, they should start where they are and gradually increase as much as they can.]

Copyright 2017 University of Pittsburgh

As before, make a plan to be active this week. Remember how important it is to make a plan for activity…it doesn't just happen.

[Give participants time to complete the chart on page 8.]

[Announce the day, time, and place for the next session.]

After the session:

- Weigh participants who did not do so prior to the group meeting.
- Complete data forms and documentation required in your setting.
- Follow your program's protocol for managing absences.
- Review the self-monitoring records from the previous week. Write brief comments. Be positive and nonjudgmental.
    - Praise all efforts to self-monitor and to change eating and activity behaviors.
    - Highlight especially any positive changes made that relate to the session topic of the week before the records were collected: coming close to their calorie, fat gram, and activity goals and any efforts to change a food or activity cue.
    - Refer to **Guidelines for Reviewing Food and Activity Records** available in the GLB Providers Portal.

Copyright 2017 University of Pittsburgh

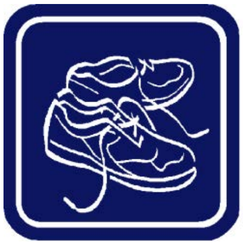

Session 8:
Step Up Your
Physical Activity Plan

In Session 4 you learned that both planned and spontaneous physical activities are important. Together they make up your total day-to-day activity level.

How are you doing with your physical activity? What has worked for you so far?

____

____

What are your challenges?

____

____

Balancing your day with a solid physical activity plan takes some effort and sometimes you will find yourself in a slump. You may need a boost to "step up" your activity plan.

Step Up Your Physical Activity with a Pedometer

So far you have been focusing mostly on your planned activity and tracking your weekly minutes. Now you will add in a pedometer.

The pedometer is a tool that can help you learn *a lot* about your total physical activity level.

Your total physical activity includes spontaneous activity and your planned activity.

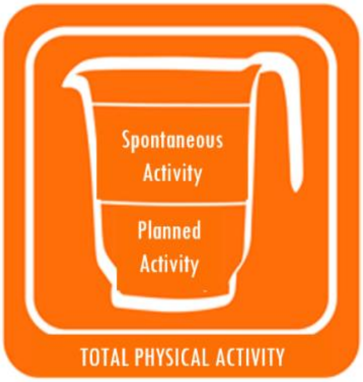

Copyright 2017 University of Pittsburgh

What is a Pedometer?

- A pedometer is a fun and simple tool that has been shown to help people be aware of and improve their activity levels.
- Pedometers measure your movement such as walking or climbing stairs throughout the day. They capture that movement in the form of steps.
- Wearing a pedometer as part of your daily routine will begin to give you a better idea of how much total movement you get. The pedometer estimates total movement by capturing most of your planned and spontaneous activity.

How Does a Pedometer Work?

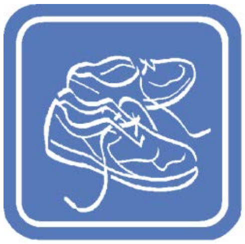

- It records a "step" each time your foot hits the ground.
- If you wear it correctly, it gives you credit for *most steps you take* throughout the day including:
  - during spontaneous activities such as household chores, or simply walking around the house
  - during planned activity such as brisk walking outside
- It does not matter where the steps are coming from. All steps are added together to give you a total count for the day.
- Pedometers do best counting steps in movement that resembles walking. You can't wear it swimming, and it will not measure biking or arm activities very well.

Why Wear a Pedometer? What Are Some Benefits?

- It can help you keep track of your total daily physical activity.
- It can help you monitor your activity when your routine changes (such as weekdays to weekends, or season to season).
- You can try fun activity challenges and learn new ways to add steps into your daily routine. The next pages will help you get to know your pedometer.

Copyright 2017 University of Pittsburgh

Pedometer Instructions

Wear your pedometer correctly to get a more accurate step-count.

When you first get up in the morning:

- Clip the pedometer to your waistband, on your side, in line with your hip.
- Reset it to zero.
- Make sure it is securely attached and worn firmly against your body so that it does not bounce.
- Do not let it flop around in a pocket and never wear it on a jacket.
- Keep it upright.
- Do not let the pedometer get wet. Don't wear it bathing or swimming. Keep it under your coat when you walk in the rain or snow.

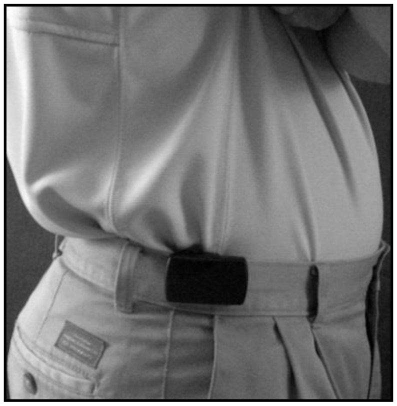

Before you go to sleep at night:

- Take it off. Put it where you can find it easily in the morning.
- Write down the number of steps you took that day in your Food and Activity record.

Important: If you think the batteries need to be changed, return the pedometer to your Lifestyle Coach.

Copyright 2017 University of Pittsburgh

Get to Know Your Pedometer and <u>Your</u> Activity Levels

Test your new pedometer with the **"100 Step Test".**

Complete this simple test to see if you're wearing the pedometer correctly and if it is measuring your steps accurately:

1. Clip the pedometer firmly at your waist.
2. Reset it to zero and close the cover.
3. Walk 100 steps.
4. Open the pedometer and check how many steps were recorded.

100 steps = Perfect
95 or 105 steps = Good (±5% error)
90 or 110 steps = Acceptable (±10% error)
85 or 115 steps = Unacceptable (±15% error)

You can repeat this test from time to time to feel confident about the accuracy and placement of your pedometer.

Pedometer Problem Solving:

If you suspect it is not working properly:

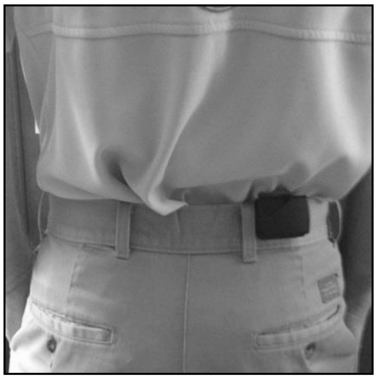

- Make sure that it is attached firmly against your torso and does not flop around.
- Try pants with a belt or a better fitting waistband.
- If it won't stay upright, or you are getting unacceptable readings, try securing the pedometer to *the back* of your waistband (see photo). Recount your steps.
- Major increases or decrease in weight may change how you should wear the pedometer. If this occurs, you can do the 100-Step Test again to test the accuracy/placement.

Bottom line: Wear your pedometer in the spot that gives you the most reliable results

Copyright 2017 University of Pittsburgh

Use Your Pedometer to Learn About Your Activity Patterns

Key Challenge #1:

<u>How many steps do you take in an average week?</u>

1. Wear your pedometer every day for seven days in a row. Record your daily steps here (or use your own self-monitoring record).

Mon	Tue	Wed	Thu	Fri	Sat	Sun

2. Add up the daily steps to get your total number of steps per week.

   My total steps per week: _______________.

3. Divide the total number of steps per week by 7 to get your average steps per day. Write this number in the box below.

My average steps per day = ______________

Are your average steps per day about what you expected?

____

Copyright 2017 University of Pittsburgh

Use Your Pedometer to Develop an "Active Head"

Key challenge #2:

1. Wear your pedometer on two days during the week when you would typically get about the same level of physical activity or movement.

2. On the first day, go about your routine activities as you normally would do. This is your Normal Day.

3. On the second day, include as many short bursts of *spontaneous activity* as you can. Do not make any major changes to your day. Rather, turn any spare minute into an active minute.

   Examples: take the stairs instead of using the elevator, walk across the hall or street to talk to your neighbor instead of using the phone.

   Think of other quick and simple ways that might work for you. This is your Active Head Day.

   Record your steps below.

Day 1: Normal Day	Day 2: Active Head Day
Total steps= ____________	Total steps= ____________

Compare the number of step taken on these two days.

- Do they differ? ☐ YES ☐ NO
- If yes, what do you think explains the difference?

  ____

- If no, what could you have added to your day to make a difference?

  ____

  ____

Subtle increases in movement throughout the day can lead to an increase in your total physical activity levels. Developing an Active Head is one way to make this happen. Your pedometer can help you measure these small increases in movement.

Copyright 2017 University of Pittsburgh

Other pedometer challenges to try:

Measure Your Mile in Pedometer Steps

- Wear your pedometer to a local school track, or any fairly level surface (a sidewalk or street) where you can walk one mile. Choose a location where the exact distance is known.
- Reset your pedometer to zero, walk the mile, and record the number of steps you took. Now you can use this "personal steps per mile" number in the future to estimate distances (miles) walked.

It takes me _________ pedometer steps to walk one mile.

Keep in mind:

- It takes, on average, about 2000 steps to walk *one mile.*
- This step count varies from person to person. A taller person will take fewer steps per mile compared to someone who is shorter because of longer stride length.
- For a moderate walking pace of 3 miles/hour, it takes about *20 minutes to walk one mile*.

Measure Your Weekday vs. Weekend Pedometer Steps

Some people tend to be more active during the weekdays while others are more active on the weekends. What is your pattern?

- Wear your pedometer and record the number of steps taken each weekday (Monday-Friday). Add up your Monday-Friday steps and divide by 5. This is your weekday average.
- Next, record the number of steps taken each day on Saturday and Sunday and divide by 2. This is your weekend average.

Average weekday steps ___________.
Average weekend steps ___________.

When are you more active? Did your answer surprise you?

____

Copyright 2017 University of Pittsburgh

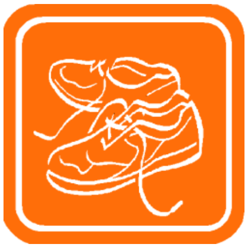

Adding Steps *Throughout* Your Day

Try some of the simple ideas and tips below.

At Home

- Make an after-dinner walk a family tradition.
- Walk your dog.
- Try to take half of your goal steps by noon.
- Get up and move around once every 30 minutes.
- Walk while you talk on the phone.
- Reward your family for meeting step goals with fun activities.
- Walk around your house during TV commercials (they average 17 minutes per one hour of TV programming)
- Walk while your kids play sports.
- Walk to your neighbor's house instead of calling.
- Start a walking club with your neighbors or friends.
- Turn off the TV and do something active with family.
- Take a walk and pick up litter in your neighborhood or in a park.
- Plan active weekends (longer walks, scenic hikes, playing in the park).
- Plan walks into your day (a friend at the beginning, with your family at the end).

On the Town

- Park farther away in parking lots.
- Limit use of elevators and escalators – use the stairs instead.
- Plan active vacations.
- Walk at the airport while waiting for your plane.
- Avoid people movers at the airport.
- Walk your grocery cart back to the store.
- Walk, don't drive, for trips less than one mile.
- Take several trips to unload groceries from your car.
- Avoid the drive-through at the bank. Instead, walk inside.

At Work

- Get off the bus earlier and walk farther to work.
- Take several 10-minute walks during the day.
- Host "walking" meetings.
- Start a break-time walking club with your coworkers.
- Walk a few laps on your floor during breaks, or go outside and walk around the block.
- Get up and move at least once every 30 minutes.
- Choose the farthest entrance to your building, then walk the long way to your office.
- Take a longer route to your meeting.
- Walk during your lunch break.
- Take 5 minute walking breaks from your computer.
- Take the stairs rather than the elevator or the escalator.
- Walk to a colleague's office rather than calling or sending an email.
- Park farther away in morning or when you go to lunch.
- Walk to a restroom, water fountain, or copy machine on a different floor.

Copyright 2017 University of Pittsburgh

Mixing It Up and Staying Active

There are many ways to add variety to your activity routine. "Mixing it up" can make activity more fun and help to prevent boredom.

Using a pedometer is one way to help you to mix it up.
Can you think of any other ways?

Ways to Mix It Up:	Ideas:
Add Variety:<br>• Do something new.<br>• Do the same activity in a new place or at a new time.<br>• Be active as a way to be social.<br>• Be active with someone new.	
Make Being Active Fun:<br>• Dance.<br>• Listen to music or audio books while being active.<br>• Look for active events such as a walking tour or a group bike ride.	
Challenge yourself:<br>• Find ways to add steps to your day.<br>• Train for an organized event such as a charity walk.<br>• Take a nature hike.	

Changes in the weather/seasons can be a barrier to keeping up with your activity plan. Can you think of ways to stay active in bad weather?

____

____

Copyright 2017 University of Pittsburgh

The F.I.T.T. Principle

Physical activity is made up of several components. It is not just about what type of activity you do, but how long you do it each time, how often you do it, and how hard you perform the activity.

One way to remember all of these components is the acronym, F.I.T.T: Frequency of activity, Intensity of activity, Type of activity and Time of activity.

So far, we have discussed frequency, type of activity and time (or duration) of your physical activity program. Intensity is the final component to consider.

F.I.T.T.	What to Do:
Frequency<br>How often are you active?	• Try to be active on most days of the week (at least 3 days per week is recommended, 5 to 7 days are even better).
Intensity<br>How hard are you working while being active? How fast is your heart beating?	• Two methods that will be described in this session are:<br>1) Rating of Perceived Exertion<br>2) Estimated Target Heart Rate
Type of Activity<br>Aerobic activity is the foundation of this program.<br>*Note: We will discuss resistance training in a later session.*	• For heart fitness, do aerobic activities that challenge your heart and lungs.<br>• Use larger muscles such as your legs. Examples: Brisk walking, swimming
Time<br>How long are you active?	• Stay active for at least 10 minutes at a time.<br>• Increase slowly.

Copyright 2017 University of Pittsburgh

Physical Activity Intensity Level

So far, your GLB activity focus has been on frequency (most days of the week), type (aerobic activities that strengthen your heart) and time (at least 150 minutes per week). Now let's focus on intensity.

Increasing physical activity intensity can improve how well your heart works. Remember, your heart is a muscle, too. If you exercise your heart by doing aerobic activity, it will become stronger and more fit over time. This is just like the muscles in your arm becoming stronger if you lift weights.

As your heart becomes stronger over time, that means your aerobic fitness is improving. "Aerobic fitness" refers to how well your heart can pump oxygen in your blood to your muscles, like those in your arms and legs. When you perform aerobic activity regularly, your aerobic fitness improves and your heart does not need to beat as fast for the same effort. As your heart becomes stronger, you may notice that it's easier for you to do things like walking up stairs and hills.

After you have reached the 150 minutes per week activity goal, consider kicking up the intensity of your activity a bit. For example, walk a little faster, just enough to notice that you are breathing a little bit harder, or add hills to your walking route.

How to Measure your Physical Activity Intensity

There are several ways that you can measure your physical activity intensity. Below are two common ways:

1. Rating of Perceived Exertion or RPE (see next page)
2. Estimated Target Heart Rate (see page 15).

In GLB, we use the RPE but both are good options.

> Please check with your health care provider if you plan to make major increases in the intensity of your activity.

Copyright 2017 University of Pittsburgh

How Hard are You Working? Estimating Rating of Perceived Exertion

Rating of Perceived Exertion (RPE) is:

- A tool that allows you to measure how hard you feel you are working while performing your physical activity.
- Based on a scale of 1 to 10 with a 1 being that the activity was very easy and a 10 being the hardest you've ever worked.
- Aim for a rating between 4 and 6. This is considered a good training range for making your heart stronger.

Most people have a good sense of how hard they are working when they're being active. Listen to your body.

Rate yourself on the following RPE scale while you are being active.

How Hard are You Working?

1	2	3	4	5	6	7	8	9	10
☐	☐	☐	☐	☐	☐	☐	☐	☐	☐
Very easy	Easy		Moderate	Somewhat hard	Hard		Very hard		Very, very hard
				Examples:					
"I'm not working hard at all. I can talk and even sing easily."			"I'm working and breathing harder than usual. I can still talk easily."	"I'm working and breathing somewhat hard. I can talk fairly easily."	"I'm working hard and breathing deeply. I can still talk."	"I'm working very hard. I can't catch my breath or talk."			
				← Stay in this range →					

Copyright 2017 University of Pittsburgh

To Do:

Check the boxes when you complete each item:

☐ Keep track of your weight. Weigh yourself at home at least once a week. Record it.

☐ Record everything you eat and drink every day. Come as close as you can to your calorie and fat gram goals.

New things to practice:

☐ Wear your pedometer every day this week. Calculate your average steps per day (see page 5).

- My average step counts per day _______________

☐ Complete the Active Head challenge on page 6.

☐ Estimate activity intensity using the rating of perceived exertion (RPE). Record it each time you are active.

☐ Optional: Calculate your estimated target heart rate (see pages 15-16).

☐ Be active for ______ minutes this week. Record what you do.

- The suggested activity goal for last week was 150 minutes.
- If you reached 150 minutes, congratulations. This will continue to be your minimum goal throughout GLB. If you are doing more, great work and keep it up.
- If you were active for less than 150 minutes, that's okay. Start at your current activity level and try adding 30 minutes more.

Copyright 2017 University of Pittsburgh

Session 8: Resources Page

Estimating your target heart rate 15

Step credit calculation 17

Copyright 2017 University of Pittsburgh

Estimating the Intensity of Your Workout by Taking Your Heart Rate

You can calculate your estimated target heart rate. This range is a guide that you can use during your aerobic activity workout. But, always listen to your body first.

Several things can affect your heart rate, such as:

- Stress
- Sickness
- Heat
- Medications (in particular blood pressure medications such as beta-blockers)

If you are interested, follow these steps:

First you need to measure your resting heart rate. You will need a clock, watch, or stopwatch with a second hand.

- Use your index and middle fingers. Don't use your thumb, which has a pulse of its own.
- Place your fingers on your wrist, just above the base of the thumb.
- Or place the tips of the fingers on your neck, on either side of the Adam's apple. Do not press too hard on the neck or you may feel dizzy or light headed. This option is less preferred.

Next, find your estimated target heart rate.

Copyright 2017 University of Pittsburgh

To calculate your estimated target heart rate:

1) Maximum heart rate: Subtract your age from 220.

220 - ___ = ____ (estimated maximum heart rate)

2) Resting heart rate: Measure your heart rate at rest by counting beats for 30 seconds and multiplying by two.

Resting beats in 30 seconds ___ X 2 = ___ (resting heart rate)

3) Target heart rate =

((maximum heart rate − resting heart rate) X % intensity) + resting heart rate.

Lower range 50% = (maximum heart rate − resting heart rate) X 0.5) + resting heart rate = ______

Upper range 70% = (maximum heart rate − resting heart rate) X 0.7) + resting heart rate = ______

Your target heart rate range is _______ to _______ beats per minute (bpm).

Finally, measure your heart rate while you are exercising.

- Take your pulse as directed above while you are in the middle of your activity, long after your warm up. Keep moving, rocking side-to-side.
- Stay within your target heart rate range.

Example for a 40-year old:

This person has an estimated maximum heart rate of 180 bpm and a resting heart rate of 70 bpm.

- 50% Target Heart Rate: [(180 – 70) × 0.50] + 70 = 125 bpm
- 70% Target Heart Rate: [(180 – 70) × 0.70] + 70 = 147 bpm
- The Target Heart Rate range = 125 − 147 bpm

Copyright 2017 University of Pittsburgh

Step Credit Calculation

For activities that are not recorded by your pedometer

Some types of physical activity aren't recorded by the pedometer or there may be situations when the pedometer cannot be worn.

The two most common examples are biking and water activities such as swimming and rowing. However, you can estimate a "step credit" for these activities and add it to your daily pedometer count.

Determine your step credit:

1. Figure out how many steps you take to walk one mile (see page 7). At a normal walking pace (3 miles/hour), it takes about 20 minutes to walk a mile.

2. Divide your number of steps by two. This will give you the number of steps you took in 10 minutes. Don't worry if you took a little more or less than 20 minutes to walk the mile. This is only an estimate.

3. Credit yourself the number of steps you calculated above for every continuous 10 minutes "non-pedometer" activities you do.

Step Credit Example

1. It takes Jane 2,000 steps to walk one mile.
2. 2,000 divided by 2 = 1,000
3. 1,000 is the number of steps that Jane will record for every 10 minutes of "non-pedometer" activities like biking, swimming, or rowing.

Copyright 2017 University of Pittsburgh

Session 8 Leader Guide:
Step Up Your Physical Activity Plan

Objectives
In this session, the participants will:

- Receive a pedometer and become familiar with it.
- Understand how to wear a pedometer, determine average daily steps, and use the pedometer as a tool to monitor physical activity levels and overall movement.
- Understand the concept of aerobic fitness and the F.I.T.T. Principle (frequency, intensity, type of activity, and time) as related to their activity goal.
- Learn two ways to estimate their physical activity intensity: by rating their perceived exertion and or by taking heart rate.

To Do Before the Session

- If providing Food and Activity books; one per participant.
- Review Food and Activity records from last session; add comments.
- Prepare Session 8 handout for participant notebook.
- Have pedometers (one per participant).
- Become familiar with the pedometer. For example, wear it for a week or two, record steps, and calculate the daily average. Also complete the 100-Step Test.

Available in the GLB Providers Portal:
https://www.diabetesprevention.pitt.edu/my/login.aspx

- **Guidelines for Reviewing Food and Activity Records**
- **2017 GLB Supplies Ordering Information**

Group Sharing Time (allow at least 5 minutes)

Let's take a few minutes to talk about your experiences this past week. *[Choose only a few of the following prompts to guide discussion.]*

- Overall, how did it go last week? What went well? What problems did you have? What could you do differently?
- What did you learn by self-monitoring?
- Were you able to stay close to your calorie and fat gram and calorie goals?
- Were you able to follow your plan for physical activity, including lifestyle activity? How did you do with reaching your physical activity goal?

[Remind participants to graph their activity if they have not yet done so.]

Copyright 2017 University of Pittsburgh

The last session was "Problem Solving". Were you able to use the five steps to problem solving and follow your action plan? What went well? What was challenging? What could you do differently?

[Problem solve with the participants to address any barriers.]

[Praise all efforts to self-monitor and to change eating and activity behaviors. Be positive and nonjudgmental. Encourage group discussion.]

Session 8: Step Up Your Physical Activity Plan (pages 1-2)

[Note: Participants may already be using some type of an activity monitor. Above all, acknowledge and encourage their efforts. Suggest that they try using the pedometer provided to the group as an additional learning exercise and to see how it compares to their current activity monitor.]

[Review the information at the top of page 1. Ask participants to answer the questions and to share their answers, if they feel comfortable doing so.]

Step Up Your Physical Activity with a Pedometer

[Review the information on the bottom of page 1 and include the following:]

So far in the physical activity portion of Group Lifestyle Balance, the focus has been on increasing the amount of time you are doing moderate intensity physical activities. Most of you have gradually increased your activity and have reached or exceeded the program goal of at least 150 minutes of moderate intensity activity. Some of you are still working toward your goal. Many of you are walking because it's easy to do and doesn't require any special equipment. Other activities of at least moderate intensity, of course, are acceptable. It is just a matter of preference.

Now let's see how you can get an estimate of your overall movement (of moderate intensity (or greater) as well as light intensity activity). This will be done through the use of pedometers.

[Review the information on page 2 and include the following:]

Pedometers are great tools that measure steps taken throughout the day. Not only do they capture the steps that you take when you do planned activities, but they also capture steps that you do in more spontaneous or unplanned activity and many light intensity activities.

Therefore, if you wear your pedometer throughout the day, you can get a better idea of how much total movement (both planned and spontaneous/light intensity movement that we discussed in Session 4) you have done in that day. Keep in mind that the pedometers will not capture activities such as arm work, or biking, (because there isn't the impact of

Copyright 2017 University of Pittsburgh

your foot hitting the ground that the pedometer counts) or water activities like swimming (because you can't wear the pedometer in the water).

Pedometer Instructions (page 3)

Before we go into the various ways to use it, let's talk about how the pedometer works and how to wear it.

[Distribute one pedometer per participant. Review page 3.]

Show participants how to wear the pedometer. Demonstrate how and where to clip on the pedometer and how to use the safety strap.

Emphasize that it is important to **reset the pedometer every night. Also emphasize that the pedometer is to be worn at the waist and must be kept in an upright position. Illustrate this by holding the pedometer upright and moving it up and down to hear the clicking sound of the pedometer recording that movement. Now move it side-to-side. You will notice that you don't hear the clicking sound in side-to-side movement. The pedometer will only register a step in the upright position.**

Participants should record their steps each day. If using the GLB Keeping Track books, explain that they should record steps in the same area where activity minutes are recorded.

Make it clear to participants that pedometers record their planned moderate intensity physical activity plus all their unplanned activity and lower intensity activity. They should **continue to self-monitor and hand record their minutes of planned activity every day**.

Get to Know Your Pedometer and Your Activity Levels (page 4)

It's important to make sure your pedometer is accurately recording your steps.

[Review page 4.]

Copyright 2017 University of Pittsburgh

Review the instructions for the **100 Step Test**. Demonstrate how to complete the test and, **where possible, have everyone do it as part of the group meeting**. In situations where the pedometer steps fall in the unacceptable range, discuss reasons why this may have occurred and solutions for fixing it.

There are several reasons why a pedometer may under-report steps. The most common reason is that the pedometer may not be staying upright in the vertical plane. If this seems to be the reason, instruct the participant to move the pedometer around the pants/belt line to their back, aligned with the rear of the knee cap. This position helps to keep the pedometer upright when placement in the front does not seem to work correctly.

Other reasons why the pedometer may not accurately reflect the number of steps taken include wearing it on loose clothing or if the participant shuffles as he/she walks without picking up his/her feet.

Use Your Pedometer to Learn About Your Activity Patterns (page 5)

Please wear your pedometer every day this coming week and record your steps each day on page 5. Then you can determine your total steps per week and your average steps per day.

[Review ***Key Challenge #1****.]*

As we discussed, there are some types of physical activity that aren't recorded by the pedometer or situations where the pedometer cannot be worn since the activity happens in water. The three most common examples are biking, resistance training, and water activities like swimming and rowing. However, you can crudely estimate a "step" credit for these activities to add to your daily pedometer count. This information is on page 17, **Step Credit Calculation**.

Next week you will set a goal that is challenging yet reasonable based on your starting average steps.

[Note: This is not the time to tell them about pedometer step goals or national step count averages. You do not want to influence their step count recording for this week. Next week you will provide them with this information.]

Use Your Pedometer to Develop an "Active Head" (pages 6 and 7)

It is also important to try to develop what we call an "Active Head". This means trying to incorporate as much spontaneous activity into your day as possible. Every little bit of movement will add up.

Copyright 2017 University of Pittsburgh

*[Review **Key Challenge #2.**]*

*[Review **Measure Your Mile in Pedometer Steps** on page 7 and include the following information:]*

When measuring a mile, keep in mind that the number of steps it takes to complete a mile will vary from person to person. This is because everyone's stride length is different. Someone shorter will likely have a shorter stride length and therefore require more steps to complete a mile than someone much taller with a longer stride length.

*[Review **Measure Your Weekday vs. Weekend Pedometer Steps** on page 7 and include the following information:]*

Sometimes it can be helpful to figure out when you are most active. One way to do this is to wear your pedometer for a week. At the end of the week, compare the number of steps taken during the week to those taken over the weekend.

[Encourage participants to try these challenges at home this coming week.]

Adding Steps Throughout Your Day (page 8)

Please review this page at home. It may give you some great ideas for ways to add steps throughout your day. You can give us feedback next week if you tried any of these suggestions.

Mixing It Up and Staying Active (page 9)

[*Review the information on page 9. Encourage group discussion while completing the chart. Include the following:]*

There are many ways to boost your activity.

Add variety.

- **Do something new and different** now and then. Mixing it up is a good strategy for you. It may get stale doing the same activity, day in and day out, every season of the year, in the same way.

  Remember, you are making life-long changes, and being active is something you will be doing for the rest of your life. So, build in some variety. For example, if you usually walk during the week, try riding a bike or swimming on the weekend. For the winter, take up cross-country skiing or perhaps indoor cycling. A great idea for all year around is to take up strength (resistance) training. We will be talking about resistance training in an upcoming session.

Copyright 2017 University of Pittsburgh

Can you think of some ways to vary what you do for activity? *[Make sure participants understand to self-monitor only the physical activities that are similar (or higher in) intensity than brisk walking.]*

- **Do the same activity in a new place.** For example:
  - Walk a different path through the park.
  - Walk in a different neighborhood after work.

  What are some ways you can vary where you do your activity?

- **Be active as a way to be social.**
  - Instead of going out for a cup of coffee, go out for a "walk and talk" with a friend or family member
  - Plan a weekend hike with a group of friends
  - Go biking with a cycling club.
  - Join a basketball or softball league
  - Sign up with a group of friends for a walk for charity.

  What are some activities you could do with a friend, family member, or group as a way to socialize?

M**ake being active fun.**

- Plan walking tours of cities when you travel.

  What would be fun for you?

Another way to prevent boredom is to **challenge yourself**.

- Train for a challenging mountain hike on your vacation.
- Set up a friendly competition with a friend (whoever walks the most miles before a certain date gets taken out for a healthy lunch by the other).

  What would make activity more challenging for you?

Have you been bored at times with your activity in the past?
Have you found anything to be particularly helpful for you at those times?

[Discuss the final question on the page. Encourage group discussion.]

The F.I.T.T. Principle (page 10)

[So far, participants have focused on the "duration" part of their aerobic activity program. Other than mentioning that they do activities similar to a brisk walk, there has been no detailed discussion about the intensity of their effort.]
Let's discuss the "F.I.T.T." principle as a great summary of both the activity components that we have discussed so far and those that we still need to consider. *[Review page 11 and include the following:]*

"F" stands for **frequency**, or **how often you are active.**

Copyright 2017 University of Pittsburgh

"I" stands for **intensity,** or **how hard you are working while being active.** (Which we will discuss in more detail in a minute).

"T" stands for **type of activity.**

- To improve your fitness, you should do **"aerobic"** activities. As we said before, these are moderate (or greater) intensity activities that **challenge your heart.** Brisk walking, swimming, and cycling are all examples of aerobic activities.

The final **"T"** stands for **time,** or **how long you are active**

- To improve your aerobic fitness, you should **stay active continuously for at least 10 minutes at a moderate intensity (or greater) and do activities that require the use of large muscle groups such as those in your legs.** Activities done briefly and/or that use small muscle groups (such as the muscles in your arms when pitching a softball or washing a window) will not greatly improve your aerobic fitness.
- The **total number of minutes per week should reach or exceed your GLB activity goal for that week.**

Physical Activity Intensity Level (page 11)

We have discussed all of the components of F.I.T.T, with the exception of intensity. So far in GLB, you have been focusing on the type, frequency, and time portion of your physical activity program with the goal of attaining 150 minutes of aerobic activity.

However, it is very important to assess the intensity of your physical activity, in other words, how hard you are working when you are active.

[Review page 11 and include the following:]

How to Measure your Physical Activity Intensity

How hard you are working is usually reflected in two ways….**how fast your heart beats and how hard you breathe.**

1. We want your heart to beat faster than it usually does so that it will become stronger, but we don't want to push you out of the moderate intensity range.

2. Your breathing should also increase to the point **that you can talk but not sing.** You should be able to have a conversation with a friend while walking, but **if you can break into song, speed it up!** On the other hand, **if you have trouble breathing and talking while you walk, slow it down.**

Both are good options but we use the RPE in GLB. If you are interested in learning more about how to estimate target heart rate, the steps are on the handout on pages 15-16 for you to review at home. Please note that certain medications can affect the heart rate so using the target heart rate range would not be appropriate.

Copyright 2017 University of Pittsburgh

How Hard are You Working? Estimating Rating of Perceived Exertion (page 12)

Let's talk a little more about how to estimate your physical activity intensity level through measuring your perceived exertion.

First let's look at the **Rating of Perceived Exertion (RPE) Scale.**

This is a tool that estimates how hard you are working by determining how you feel while performing physical activity. Rate yourself on this scale while you're being active. How hard are you working? Your goal is to achieve an activity intensity around "Somewhat Hard", which is between 4-6 on the RPE scale.

Review this page with the participants. The scale provided in the box is a modified version of the Borg's Rating of Perceived Exertion (RPE) Scale. This modified numerical scale ranges from 1 to 10, with 1 corresponding to very easy, 5 to somewhat hard, and 8 to very hard. The activity intensity goal for this program is to achieve and maintain ratings between 4 and 6.

To Do (page 13)

Turn to page 13 and let's focus on what you can do between now and the next session.

Please continue to keep track of your weight and eating. Come as close as you can to your calorie and fat gram goals.
New things to practice:

*[Review this section. Encourage participants wear the pedometer every day and to try some of the ways for "**Adding Steps Throughout Your Day**" listed on page 8. In addition to completing the "100 Step" (page 4) in class, ask participants to occasionally do this at home.]*

You may also find it interesting to complete the two pedometer challenge activities listed on page 7, **Measure Your Mile in Pedometer Steps** and **Measure Your Weekday vs. Weekend Pedometer Steps.** You can share your experience/feedback with the group next week.

Focus on your activity intensity each time you are participating in your planned activity this week. You may need to adjust how hard you are working during an activity so that you stay within your ideal rating of perceived exertion range.

Let's talk about your activity goal for this coming week.

Copyright 2017 University of Pittsburgh

- If your goal for last week was 150 minutes and you reached it, congratulations. This will continue to be your minimum goal throughout GLB. If you are doing more, great work and keep it up. *[Note: This is the standard GLB exercise progression. Many participants will be able to comply. However, flexibility is included based on health or other barriers to exercise.]*

- If you were active for less than 150 minutes last week, that's okay. Start at your current activity level and try adding 30 minutes more this week.

[Note: You should make exceptions for individuals with serious health problems that limit their mobility. For them, they should start where they are and gradually increase as much as they can.]

[Announce the day, time, and place for the next session.]

After the session:

- Weigh participants who did not do so prior to the group meeting.
- Complete data forms and documentation required in your setting.
- Follow your program's protocol for managing absences.
- Review the self-monitoring records from the previous week. Write brief comments. Be positive and nonjudgmental.
    - Praise all efforts to self-monitor and to change eating and activity behaviors.
    - Highlight especially any positive changes made that relate to the session topic of the week before the records were collected: working on solving an eating or activity problem and coming close to their calorie, fat gram, and activity goals.
    - Refer to **Guidelines for Reviewing Food and Activity Records** available in the GLB Providers Portal.

Copyright 2017 University of Pittsburgh

Session 9:
Manage Slips and
Self-Defeating Thoughts

Progress Review

Changes you have made so far:

To be more active:

Planned physical activity: ________________________________

Spontaneous activity: ___________________________________

To change your eating pattern:

Fewer calories: ____

Healthier food choices: __________________________________

Have you reached the 7% weight loss goal? ☐ Yes ☐ No

Are you on track with your personal weight loss goal? ☐ Yes ☐ No

Have you reached the goal of 150 minutes per week of physical activity? ☐ Yes ☐ No

Are you on track with your personal activity goal? ☐ Yes ☐ No

How will you continue to make progress? Describe the eating and activity changes that you are willing to focus on right now:

____

____

____

Copyright 2017 University of Pittsburgh

What is a Healthy Weight for You?

If you want to lose more than 7% of your Session 1 weight, keep the following in mind:

- Health care providers often use the Body Mass Index or BMI (see page 13) to help them decide what weight is healthy for someone. It is only one of many tools. It is not perfect.
- The DPP advised participants not to go below a BMI of 21.
- Talk with your health care provider about what weight is right for you, given your age and overall health.
- After talking with your health care provider, choose a 5 pound weight range you would like to reach and maintain.

My personal weight goal range is _____ - _____ pounds

Copyright 2017 University of Pittsburgh

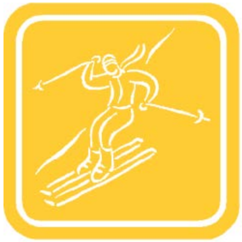

The Slippery Slope of Lifestyle Change

"Slips" are:

- Times when you do not follow your plans for healthy eating or being physically active.
- A normal part of lifestyle change.
- To be expected.

> Slips do not hurt your progress.
> What hurts your progress is the way you *react* to slips.

What things cause you to slip from healthy eating?

____

____

What things cause you to slip from being physically active?

____

____

What causes you to slip is learned. It is a habit. The way you react to slips is also a habit.

> You can learn a *new way to react* to slips
> to get back on your feet again.

Copyright 2017 University of Pittsburgh

What to Do After a Slip

First, remember two things:

1. Slips are normal and to be expected.

   99.99% of all people on their way to losing weight and being more active have slips.

2. No one time of eating too much or being inactive, no matter how extreme, will ruin everything.

   The slip is not the problem. The problem occurs if you do not get back on your feet again and keep going toward your goals.

So after you slip:

1. Talk back to self-defeating thoughts with positive thoughts.

   Self-defeating thoughts, such as "I'm a failure," can be your worst enemy. Talk back. "I'm not a failure because I've slipped. I can get back on my feet again."

2. Ask yourself what happened.

   Learn from the slip. Can you avoid it in the future? Manage it better?

3. Regain control the very next time you can.

   Do <u>not</u> tell yourself, "Well, I blew it for the day." Make your very next meal a healthy one. Get back on schedule with your activity plan right away.

4. Talk to someone supportive.

   Talk with family, friend, group members or your coach. Discuss your new strategy for handling slips. Commit yourself to renewed effort.

5. Focus on all the positive changes you have made.

   You are making life-long changes. Slips are just one part of the process.

Copyright 2017 University of Pittsburgh

Practice: Help Sadie Manage the Slippery Slope of Lifestyle Change

Take turns reading the cards numbered 1 to 6.

After hearing Sadie's story discuss the questions at the bottom of the page.

1. Sadie lost 15 pounds over a 4 month period, slowly but steadily. She felt better than she had in years and was proud of herself. Then, she decided to go away for a long weekend to visit her sister.	2. After doing so well in the program, Sadie felt she deserved a break from her hard work on healthy eating and activity. She decided to go away without her self-monitoring tools and to eat and drink whatever she wanted.
3. Sadie weighed herself as soon as she got home and couldn't believe that she gained 5 pounds. She knew that she had slipped and got right back on track with recording and meeting her eating and activity goals. It took 3 weeks to lose the 5 pounds she gained over one weekend, but she did it.	4. Sadie kept thinking about how little time it took for her to regain 5 pounds and how long and hard it was to lose it again. She thought, "If I can't just enjoy myself for a few days, why do I even bother?" She felt sometimes like there was no use in continuing to try to stick to her long term goals.
5. Sadie found it harder to stay on track. She rarely completed her self-monitoring books and became lax in planning ahead for healthy meals. She started watching more TV. She gained 5 pounds, again. Sadie thought, "I don't want to face the weigh-in at the next class…it's too upsetting."	6. One week of avoiding the group turned into 4 missed sessions. Sadie was now close to her baseline weight. Sadie realized she needed the group support and coaching now more than ever. She felt embarrassed and uneasy but she pushed herself go to the next class.

- What are Sadie's "high risk situations"? ________________________
- What does Sadie do well? Not so well? _______________________
- What parts of Sadie's story do you relate to? __________________
- How could her slips be managed better? ______________________

Copyright 2017 University of Pittsburgh

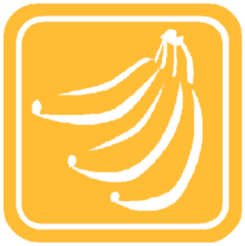

Slips from Healthy Eating

Describe one thing that has caused you to slip from healthy eating:

____

Can you avoid it in the future? If so, how? __________________________

Make a plan for how to get back on your feet when you have a slip:

There will be roadblocks, how will you handle them?

____

How can I get the support I need? ____________________________

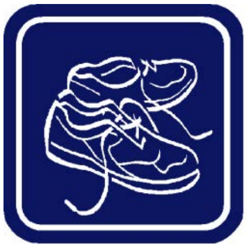

Slips from Being Physically Active

Describe one thing that has caused you to slip from being physically active:

____

Can you avoid it in the future? If so, how? __________________________

Make a plan for how to get back on your feet when you have a slip:

Roadblocks that might come up, how will you handle them?

____

How can I get the support I need?

____

Copyright 2017 University of Pittsburgh

Self-Defeating Thoughts

Self-defeating thoughts can lead you to overeat or be inactive. A vicious cycle can result. For example:

Thought: "I'm tired of working so hard. I'm sick of being in this program. I can never eat what I want."

Result: You eat potato chips.

Thought: "I did it again. I'll never lose weight."

Result: You feel discouraged and eat more.

Common Kinds of Self-Defeating Thoughts		Examples
Good or Bad	Divide the world into:<br>• Good or bad foods<br>• Seeing yourself as a success or failure<br>• Being on or off the program	"Look at what I did. I ate that cake. I'll never be able to succeed in this program."
Excuses	Blame something or someone else for our problems.<br>Do not mean to go off the program, but we "can't help it."	"But I'm really enjoying myself."<br>"I have to buy these cookies just in case company drops in."
Should	Expect perfection.<br>A set-up for disappointment.<br>Leads to anger and resentment.	"I should have eaten less of that dessert."
Not As Good As	Compare ourselves to someone else.<br>Blame ourselves for not measuring up.	"Mary lost two pounds this week. I only lost one."
Give Up	Tempted to stop trying.<br>(Often follow other kinds of self-defeating thoughts.)	"This program is too hard. I might as well forget it."

Copyright 2017 University of Pittsburgh

How to Talk Back to a Self-Defeating Thought

1. Catch yourself. Think, "I'm doing it to myself."
2. Imagine shouting, "STOP!" to yourself. Picture a huge, red stop sign.
3. Talk back with a positive thought.

Self-Defeating Thought	Talk Back with a Coping Statement
Good or Bad<br>• "I can never eat dessert again."<br>• "Look at what I did. I ate that cake. I'll never succeed."	Work Toward Balance<br>• "I can eat that dessert and then cut back on something else."<br>• "One slip-up isn't the end of the world. I can get back on track."
Excuses<br>• "It's too cold to take a walk."<br>• "I don't have the willpower."	**It's Worth a Try**<br>• "I can try going for a walk and stop if it gets too cold."<br>• "It's hard to change old habits. But I'll give it a try and see how it goes."
Should<br>• "I should have eaten less dessert."<br>• "I have to write down everything I eat."	**It's My Choice**<br>• "It was my choice. Next time I can decide not to eat so much."<br>• "I'm writing down everything I eat because it helps me eat better."
Not As Good As<br>• "Mary lost two pounds this week, and I only lost one."	Everyone Is Different<br>• "It's not a race. Mary and I can lose weight at different rates and both succeed."
Give Up<br>• "This program is too hard. I might as well forget it."<br>• "I'll never get it right."	One Step at a Time<br>• "I've learned something about what's hard for me."<br>• "I'll try something different next time."

Copyright 2017 University of Pittsburgh

Practice: Help Sam Talk Back

Work together in small groups to practice talking back to self-defeating thoughts (you can also do this, on your own, at home).

1. Say each of Sam's self-defeating thoughts out loud. Then say, "Stop"
2. Talk back, out loud, with a positive thought. Write it down.
3. Imagine self-defeating thoughts that you might have.
4. Talk back, out loud, with a positive thought. Write it down.

Self-Defeating Thought	STOP	Positive Thought
Sam: "I am letting go this weekend…I will eat and drink whatever I want."	STOP	
Sam: "If I can't enjoy *myself for a few days,* ***maybe I don't want to do*** *this program."*	STOP	
Sam: "All I really want to *do is lie on the couch with* ***my remote and watch TV."***	STOP	
Sam: "I would like to *avoid the* ***scale this week."***	STOP	
My self-defeating thoughts:	STOP	

Managing slips (and preventing relapse) means finding ways to cope with negative or self-defeating thoughts and behaviors not just once, but many times. Practicing talking back can help you become stronger and more confident in managing slips.

Copyright 2017 University of Pittsburgh

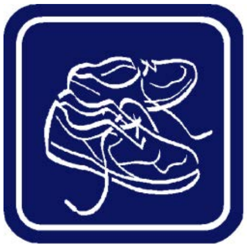

Checking In On the Pedometer

Last week you learned that a pedometer is a fun, simple tool to keep track of your steps throughout the day.

One of the Key Challenges was to calculate your average steps per day.

What were your *average steps* per day? ____________________

How does my step count compare to others?

There is no clear "national step goal" for all adults, although national health organizations have provided some guidelines. These guidelines are based on research and show that age and health status influences the average amount of steps a person takes per day.

What is the recommended GLB step goal?

GLB recommends that you work up to the same step goal that was used in DPP.

- Aim for about 7,000 steps per day, or about 50,000 steps per week.
- This is just a guideline; your personal goal may vary.
- It is important to be aware of what your average steps per day are *right now*, then increase safely and gradually.
- When adding more steps, try adding about 250-500 steps per day.
- Talk back to self-defeating thoughts about your activity and keep trying.

Copyright 2017 University of Pittsburgh

To Do:

Check the boxes when you complete each item:

☐ Keep track of your weight. Weigh yourself at home at least once a week. Record it.

☐ Record everything you eat and drink every day. Come as close as you can to your calorie and fat gram goals.

☐ Record your minutes of physical activity. Come as close as you can to the GLB goal of at least 150 minutes per week.

New things to practice:

☐ Catch yourself thinking self-defeating thoughts. Record them.

☐ Practice STOP-ping them and talking back with positive thoughts.

After that, did you think, feel, or act in a new way? _____________

____

☐ Try your two action plans for handling slips. See page 6.

☐ Answer these questions:

Did your action plans work? _______________________________

If not, what went wrong? What could you do differently? ________

____

☐ Add at least 250 steps per day.

Last week, what was your average step count per day? _________

_________________ plus 250 steps = ____________________

Average steps per day (last week) — Daily step goal (next week)

Copyright 2017 University of Pittsburgh

Session 9: Resources — Page

Session 9: Resources	Page
Body Mass Index (BMI) Chart	13
Build a Better Morning Meal	14-15
Build a Better Light Meal	16-7
Build a Better Main Meal	18-19
About Your Snacking Habits	20-21
Satisfying Snacks	22

Copyright 2017 University of Pittsburgh

Body Mass Index Table for Adults Age 20 and Over

Find your height in the first column. Move across that row to find the weight that is closest to yours. Your BMI will be at the top of that column.

	Normal						Overweight					Obese										Extreme Obesity														
BMI	19	20	21	22	23	24	25	26	27	28	29	30	31	32	33	34	35	36	37	38	39	40	41	42	43	44	45	46	47	48	49	50	51	52	53	54
Height (inches)	Body Weight (pounds)																																			
58	91	96	100	105	110	115	119	124	129	134	138	143	148	153	158	162	167	172	177	181	186	191	196	201	205	210	215	220	224	229	234	239	244	248	253	258
59	94	99	104	109	114	119	124	128	133	138	143	148	153	158	163	168	173	178	183	188	193	198	203	208	212	217	222	227	232	237	242	247	252	257	262	267
60	97	102	107	112	118	123	128	133	138	143	148	153	158	163	168	174	179	184	189	194	199	204	209	215	220	225	230	235	240	245	250	255	261	266	271	276
61	100	106	111	116	122	127	132	137	143	148	153	158	164	169	174	180	185	190	195	201	206	211	217	222	227	232	238	243	248	254	259	264	269	275	280	285
62	104	109	115	120	126	131	136	142	147	153	158	164	169	175	180	186	191	196	202	207	213	218	224	229	235	240	246	251	256	262	267	273	278	284	289	295
63	107	113	118	124	130	135	141	146	152	158	163	169	175	180	186	191	197	203	208	214	220	225	231	237	242	248	254	259	265	270	278	282	287	293	299	304
64	110	116	122	128	134	140	145	151	157	163	169	174	180	186	192	197	204	209	215	221	227	232	238	244	250	256	262	267	273	279	285	291	296	302	308	314
65	114	120	126	132	138	144	150	156	162	168	174	180	186	192	198	204	210	216	222	228	234	240	246	252	258	264	270	276	282	288	294	300	306	312	318	324
66	118	124	130	136	142	148	155	161	167	173	179	186	192	198	204	210	216	223	229	235	241	247	253	260	266	272	278	284	291	297	303	309	315	322	328	334
67	121	127	134	140	146	153	159	166	172	178	185	191	198	204	211	217	223	230	236	242	249	255	261	268	274	280	287	293	299	306	312	319	325	331	338	344
68	125	131	138	144	151	158	164	171	177	184	190	197	203	210	216	223	230	236	243	249	256	262	269	276	282	289	295	302	308	315	322	328	335	341	348	354
69	128	135	142	149	155	162	169	176	182	189	196	203	209	216	223	230	236	243	250	257	263	270	277	284	291	297	304	311	318	324	331	338	345	351	358	365
70	132	139	146	153	160	167	174	181	188	195	202	209	216	222	229	236	243	250	257	264	271	278	285	292	299	306	313	320	327	334	341	348	355	362	369	376
71	136	143	150	157	165	172	179	186	193	200	208	215	222	229	236	243	250	257	265	272	279	286	293	301	308	315	322	329	338	343	351	358	365	372	379	386
72	140	147	154	162	169	177	184	191	199	206	213	221	228	235	242	250	258	265	272	279	287	294	302	309	316	324	331	338	346	353	361	368	375	383	390	397
73	144	151	159	166	174	182	189	197	204	212	219	227	235	242	250	257	265	272	280	288	295	302	310	318	325	333	340	348	355	363	371	378	386	393	401	408
74	148	155	163	171	179	186	194	202	210	218	225	233	241	249	256	264	272	280	287	295	303	311	319	326	334	342	350	358	365	373	381	389	396	404	412	420
75	152	160	168	176	184	192	200	208	216	224	232	240	248	256	264	272	279	287	295	303	311	319	327	335	343	351	359	367	375	383	391	399	407	415	423	431
76	156	164	172	180	189	197	205	213	221	230	238	246	254	263	271	279	287	295	304	312	320	328	336	344	353	361	369	377	385	394	402	410	418	426	435	443

Source: Adapted from *Clinical Guidelines on the Identification, Evaluation, and Treatment of Overweight and Obesity in Adults: The Evidence Report.*

Copyright 2017 University of Pittsburgh

Build a Better Morning Meal

Keep breakfast simple. Use MyPlate as a model. Include healthy food choices, such as:

Vegetables

- Vegetables added to egg dishes
- Tomato, vegetable, or carrot juice

Fruit

- Fresh fruit or canned fruit packed in water or juice (not syrup)
- Eat whole fruit more often than fruit juice
- Eat less often: sweetened juice, fruit drinks, fruit canned in syrup

Grains

- Oatmeal or cereals that are low in sugar and high in fiber
- Whole -grain toast, English muffin, or bagel
- Eat less often: "frosted" or sweetened cereals, granola, cereals with nuts or coconut, pastries, croissants, biscuits, and most muffins

Protein

- Eggs, egg substitutes, seafood, cooked dried beans, soy products, or peanut butter
- Eat less often: bacon (except Canadian bacon) and sausage

Dairy

- Fat-free or low-fat (1%) milk/soymilk/cheese
- Fat-free or low-fat yogurt, with no added sugar
- Eat less often: Whole or 2% milk, high-fat/sugar yogurt, regular cheese

Limit saturated fat and added sugar. Eat these foods less often: sugar, honey, high-calorie coffee beverages, regular syrup, cream cheese, pastries, coffeecake, and doughnuts.

Copyright 2017 University of Pittsburgh

My Best Breakfasts

Look through your completed Food and Activity records. Find some breakfasts that most closely follow MyPlate.

- What food choices work well for you?
  ____

  ____

  ____

- What calorie and fat gram goals would work well for you at breakfast?

Calorie goal for breakfast: ___________ calories
Fat gram goal for breakfast: ___________ grams

Use the above to build **three "standard" breakfast menus** for yourself.

1.____

____

____

2.____

____

____

3.____

____

____

Copyright 2017 University of Pittsburgh

Build a Better Light Meal

Make one meal each day a "light" meal. Use MyPlate as a model. Think simple and quick. Examples:

- Sandwich, carrot sticks, fruit, milk
- Salad, tuna, whole-grain crackers
- Pasta, veggies, cooked dried beans
- Brown rice, stir-fried veggies, chicken
- Burrito, salad, fruit
- Frozen entree, salad, fruit, milk

Keep healthy, "quick-to-fix" food choices on hand, such as:

Vegetables

- Peeled carrots, prepared raw vegetables from the salad bar, pre-washed salad greens in a bag, canned tomatoes, frozen mixed vegetables

Fruit

- Fresh fruit or canned fruit in water or juice

Grains

- Whole grain breads, rolls, bagels, English muffins, pita bread, tortillas
- Whole grain crackers, unsweetened hot or high fiber cold cereals, quick cooking brown rice, whole-wheat couscous, other types of whole grain pasta

Protein

- Water-packed tuna, salmon, chicken; sliced turkey or chicken breast; sliced extra lean ham; canned or cooked dried beans (garbanzos, black beans, navy, kidney beans, black eye peas, etc.); vegetarian refried beans

Dairy

- Fat-free or low-fat (1%) milk/soymilk/cheese
- Fat-free or low-fat yogurt, with no added sugar

Other: Canned, low-fat soups and broth; low-fat sauces, including spaghetti sauce; nonfat or low-fat salad dressings; salsa; flavored vinegars; spicy mustard; low-calorie frozen entrees

Copyright 2017 University of Pittsburgh

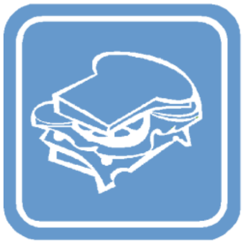

My Best Light Meals

Look through your completed Food and Activity records. Find some light meals that most closely follow MyPlate.

- What food choices work well for you?

  ____

  ____

  ____

- What calorie and fat gram goals would work well for you at light meals?

Calorie goal for light meal: ___________ calories

Fat gram goal for light meal: ___________ grams

Use the above to build **three "standard" light meal** menus for yourself.

1. ____

   ____

   ____

2. ____

   ____

   ____

3. ____

   ____

   ____

Copyright 2017 University of Pittsburgh

Build a Better Main Meal

Use MyPlate as a model for your main meals. Make healthy food choices, such as:

Vegetables

- Cooked vegetables without added fat as side dishes
- Vegetables added to spaghetti sauce, lasagna, chili, stew, grain dishes
- Colorful salads with low-fat or vinaigrette dressing

Fruit

- Fresh, dried, or canned fruit (in juice) added to vegetable salads
- Fruit for dessert

Grains

- Whole-grain pasta, bread, tortillas, and pita bread
- Brown rice, barley, couscous, wild rice, quinoa, and bulgur

Protein

- Lean cuts of meat, poultry, or seafood, cooked without fat
- Hummus, canned or cooked dried beans, split peas, or lentils, cooked without fat or fatty meats (Serve in place of meat more often. Try them in casseroles, soups, or salads. Make chili with more beans and veggies.)
- Nuts and seeds.
- Watch portion sizes

Dairy

- Fat-free or low-fat (1%) milk/soymilk/cheese
- Fat-free or low-fat yogurt, with no added sugar

Limit saturated fat and added sugar. Drink fat-free or 1% milk. Save calories by drinking water instead of sugary drinks.

Copyright 2017 University of Pittsburgh

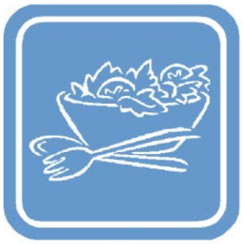

My Best Main Meals

Look through your completed Food and Activity records. Find some main meals that most closely follow MyPlate.

- What food choices work well for you?
  ____

  ____

  ____

- What calorie and fat gram goals would work well for you at main meals?

Calorie goal for main meal: __________ calories

Fat gram goal for main meal: ___________ grams

Use the above to build **three "standard" main meal menus** for yourself.

1. ____

   ____

   ____

2. ____

   ____

   ____

3. ____

   ____

   ____

Copyright 2017 University of Pittsburgh

About Your Snacking Habits

Think about your snacking habits.

A Snack You Eat Often, Amount	Fat Grams, Calories	Where and When You Eat This Snack
1.		
2.		
3.		
4.		
5.		

Are most of your snacks planned or unplanned? _______________

Unplanned snacks are often triggered by (check what applies to you):

- ☐ Being too hungry
- ☐ Being tired or overworked
- ☐ Feeling stressed, anxious, bored, or angry
- ☐ Seeing or smelling food, or others eating
- ☐ Doing certain things or being in certain places (such as watching TV or a movie)
- ☐ Holidays or family events
- ☐ Other:

____

____

How could you avoid these triggers? Or handle them in a healthier way? (Example: Do you eat potato chips while you watch TV? You could stop buying chips. You could keep a bowl of fresh fruit in the TV room.)

____

____

Copyright 2017 University of Pittsburgh

My Best Snacks

Look through your completed Food and Activity records. Find some healthy snacks.

- What food choices, times, and places for snacks work well for you?

  ____

  ____

- What calorie and fat gram goals would work well for you for snacks?

Calorie goal for snacks: __________ calories

Fat gram goal for snacks: ________ grams

Use the above and the list of snacks on the next page to build seven **"standard" snacks** for yourself.

1. ____
2. ____
3. ____
4. ____
5. ____
6. ____
7. ____

Copyright 2017 University of Pittsburgh

Satisfying Snacks

Choose snacks with a taste and texture you enjoy.

- Use MyPlate as a guide for snack choices.
- Watch portion sizes. Aim for less than 200 calories.
- Limit snacks with empty calories from added sugars, saturated fat, and alcohol.

Crunchy Snacks:

- Fresh fruit (such as apple, pear)
- Raw vegetables (such as broccoli, carrots, cauliflower, green pepper, celery) with hummus
- Low-fat crackers (such as oyster, Melba, matzo, rice crackers, crisp bread), with low-fat cheese or peanut butter
- Popcorn, air-popped or light
- Cereal and low-fat or fat-free milk
- Pretzels
- Baked tortilla chips and salsa
- Rice cakes, popcorn cakes
- 100-calorie package of nuts

Sweet Snacks

- Fresh fruit (such as orange, banana, cherries, kiwi, grapes, blueberries, strawberries, peach, plum, pineapple, melon)
- Gelatin
- Low-fat cookies (such as ginger snaps, graham crackers, vanilla wafers)

Chewy Snacks

- Dried fruit (raisins, apricots)
- Chewy breads (such as English muffin, bagel, pita, breadsticks)
- Low-fat cheese sticks

Chocolate Snacks

- Fudgesicle, regular or sugar-free
- Low-fat chocolate pudding
- Chocolate nonfat milk
- Chocolate graham crackers
- Low-fat ice cream sandwich

Smooth or Frozen Snacks

- Applesauce, unsweetened
- Low-fat cottage cheese with fruit
- Low-fat or fat-free pudding
- Yogurt, low-fat or fat-free, with no added sugar
- Fruit smoothie
- Frozen yogurt, nonfat
- Sherbet, sorbet, frozen fruit bars, lemon ice, or popsicles
- Frozen grapes, bananas, berries

Copyright 2017 University of Pittsburgh

Session 9 Leader Guide:
Manage Slips and Self-Defeating Thoughts

Objectives
In this session, the participants will:

- Review their progress since Session 5.
- Identify some things that cause them to slip from healthy eating or being active.
- Discuss what to do after a slip to "get back on your feet again."
- Practice identifying high risk situations and strategies for managing a slip.
- Make a plan to get back on their feet after slips from healthy eating or being active.
- Recognize that everyone has self-defeating thoughts. Identify examples.
- Learn how to stop self-defeating thoughts and talk back to them.
- Practice stopping negative thoughts and talking back to them.
- Discuss their experience with the pedometer.
- Compare their steps with the recommended GLB step goal.
- Learn how to gradually and safely increase steps using the pedometer.

To Do Before the Session:

- If providing Food and Activity books; one per participant.
- Review Food and Activity records from last session; add comments.
- Prepare Session 9 handout for participant notebook.
- Make a list of resources for behavioral and mental health referrals. Some participants may raise problems outside the expertise of the Lifestyle Coach, such as a significant clinical depression, anxiety, or a clinical eating disorder. Lifestyle Coaches should be prepared to make referrals to address such problems.
- Optional: Have a large Stop sign to use as a prop in class.

Available in the GLB Providers Portal:
https://www.diabetesprevention.pitt.edu/my/login.aspx

- **Guidelines for Reviewing Food and Activity Records**

Copyright 2017 University of Pittsburgh

Group Sharing Time (allow at least 5 minutes)

In the last session, you learned how to step up your activity plan by using a pedometer and how to have an active head. You also learned about the FITT principle and how to use the RPE scale to measure your exercise intensity.

- Did you wear your pedometer? How did it go? Did it help you to move more in your daily life? Did it help you reach your activity goal? What are some things you did this past week to add steps?
- Did you complete the Active Head challenge? Please share your experience.
- Were you able to use the RPE scale to estimate how hard you were working during exercise? Please share your experience.

Let's take a few minutes to talk about your experiences this past week. *[Choose only a few of the following prompts to guide discussion.]*

- Overall, how did it go last week? What went well? What problems did you have? What could you do differently?
- How close did you come to your calorie and fat gram goal?
- What change did you make in your eating pattern this past week that you are most proud of?
- Were you able to follow your plan to reach your goal for minutes of planned activity during the week? How did it go? What did you learn?

[Problem solve with the participants to address any barriers.]

[Praise all efforts to self-monitor and to change eating and activity behaviors. Be positive and nonjudgmental. Encourage group discussion.]

Progress Review (pages 1-2)

In GLB we will sometimes pause and give you a chance to think about the changes you have made so far in your eating and activity behaviors. This allows you to celebrate the positive lifestyle changes you have made. It also allows you to think about your focus for the upcoming month.

First, let's take some time to review your progress since Session 5, which is the last time we formally looked at how you were doing. *[Distribute Session 9 handout.]*

First, **what changes have you made to be more active**? We've talked about increasing both structured/planned activity, the kind you've been recording, and spontaneous/ unstructured activity, like taking the stairs instead of an elevator, that add more total movement to your day.

Copyright 2017 University of Pittsburgh

[Give the participants some time to briefly record on page 1 some of the changes they've made. Ask volunteers to share, if they feel comfortable doing so. Praise and encourage the maintenance of these changes.]

Now let's talk about the **changes you have made in your eating pattern.** Think about changes you have made to eat fewer calories and to make healthier food choices.

[Again, have participants briefly record some of the changes they've made. Ask volunteers to share, if they feel comfortable doing so. Praise and encourage the maintenance of these changes.]

Lifestyle change is a process. The purpose is to look at progress toward reaching your goals. No one is looking for perfection.

Let's look at how the scale has responded.

Look at your **Weekly Record**. Have you reached the GLB 7% weight loss goal? Check yes or no.

Are you on track with your personal weight loss goal? Check yes or no.

- Some people have the goal of losing 7% while others would like to continue losing until they reach a personal weight loss goal.
- Let's talk about what is a healthy weight for you. *[Review the information on page 2,* ***What is a Healthy Weight for You****. Explain how to read the BMI chart on page 13. Emphasize that this is not a perfect tool. Stress the importance of talking with their health care provider about what weight is right for them, given age and overall health. However, any "shift to the left" on the chart is a sign of progress toward improving health.]*
- No one weighs the same every day so we ask that you determine a 5 pound weight range you would like to stay within. This applies to those of you who want to reach and maintain the 7% goal and those who would like to continue losing more weight.

Now let's look at your physical activity. *[Participants should refer to the* ***How am I Doing?*** *graph.]*

- Have you reached the goal of 150 minutes of physical activity per week?
- Some people have the own personal activity goal. Are you on track with this goal? *[Ask for volunteers to share, if they feel comfortable doing so.]*

Copyright 2017 University of Pittsburgh

Emphasize that it's important for the participants to focus on their accomplishments so far, no matter how small, and express your confidence in the participants' future success.

Encourage attendance at group meetings, even if they are struggling. It is not uncommon for participants to want to avoid the meetings if they feel they are not doing well. However, this is usually when they most need the group support, suggestions, and encouragement.

Pages 14-22 provide information about how to "build a better" breakfast, lunch, dinner, and snack. These pages will be helpful as you continue to shift to healthier food choices and a healthy eating pattern.

Finally, let's look at the box at the bottom of the page. Think about how you will continue to make progress toward reaching and maintaining your weight, eating, and activity goals. Write down something that you are willing to focus on right now. *[Ask for volunteers to share, if they feel comfortable doing so.]*

The Slippery Slope of Lifestyle Change (page 3)

Today we're going to talk about "slips," or times when you don't follow your plans for healthy eating or being active. *[Review page 3 and include the following:]*

Let's use skiing as an example. Everyone who learns to ski knows that they will "slip" and fall down. It's a natural part of learning to ski. What a skiing instructor does is to help beginning skiers anticipate when they might fall down and show them how to get up again. That's what we'll do today--talk about when you might "slip" from your eating and activity plans, and how you can get back on track again after you slip.

Note: Throughout this session, try to use analogies in addition to skiing that are meaningful to the participants. (For example, one analogy is how we handle fires. First, we try to identify high-risk situations in which fires are likely to occur. Second, we try to take steps to avoid these situations if we can. Third, in case a fire does occur, we plan ahead for a way to put out the fire and/or escape. We make a plan that is as simple and easy to remember as possible so that we are more likely to follow it while under stress.) You will also want to use a meaningful analogy for how the participants have developed other skills by making mistakes and learning from them, such as learning to drive a car, bake a cake, and so on.

Now let's move on to the topic for today, **"slips."** *[Review page 3 and include the following:]*

Slips are:

- **A normal part of lifestyle change.** Just like falling down is a normal part of skiing. If you are going to ski, you are going to fall. All skiers will fall. And

Copyright 2017 University of Pittsburgh

everyone who sets out to lose weight and be more active will have slips.

- **To be expected.** If you haven't already had some slips, you most certainly will have them in the future. Slips are **inevitable.**

Does this sound discouraging? Well, it doesn't have to be. Because **slips don't hurt your progress. What hurts your progress is the way you *react* to slips.** So today we'll talk about the best way to react to slips when they happen.

Different people have different things that lead to a slip. For example, many people may respond to **moods or feelings** with slips from healthy eating or being active.

Some tend to overeat when **happy**. Imagine that:

> Your **family is celebrating.** Maybe it's a holiday, a birthday, or a vacation. There is plenty of everyone's favorite foods, from appetizers to desserts. And for years, your family's custom has been to "take it easy," have fun and just relax during these times. **What would this situation be like for you? Would you tend to slip in this kind of situation?**

Some people are more vulnerable to overeating when **bored**. Imagine that:

> You're **at home alone, watching a favorite TV program.** You're feeling okay, pretty relaxed, but a little bored. A commercial comes on at the end of the program, and you find yourself wandering into the kitchen. **What would this be like for you?**

Other people overeat when **upset**. Imagine that:

> You are settling down for a relaxing evening at home. Someone in your family starts to talk about something that's been part of an **ongoing argument** between the two of you. You both get angry and he or she stomps out of the house, slamming the door. You head for the kitchen. **What would this situation be like for you?**

Or here's another example:

> You're **behind on a project at work.** The boss has been looking in on you every 10 minutes and glaring at you impatiently. You feel pressured and very tense. You go get yourself a cup of coffee and see a delicious snack that someone brought in that morning. **What would this be like for you?**

Which is the *most* difficult for you in terms of slipping from healthy eating: feeling happy, bored, or upset? Are there other things that cause you to slip from healthy eating?
[Give the participants time to name a few examples. Have them record the examples on page 3, and then ask volunteers to share.]

What things cause you to slip from being active?

[Ask the participants to name several examples, such as vacations, holidays, feelings or moods, cold or hot weather, and to record on page 3. Ask volunteers to share.]

The situations that lead to slips differ from person to person. For example, you may tend to eat when you're bored, whereas someone else may get involved in a hobby. Or when you are at a party, you may be so busy talking and laughing that you forget to eat, whereas someone else may find the goodies are just too tempting. **What causes you to slip is learned. It is a habit.**

The way you react to slips is also a habit. You can learn a new way to react to slips that will get you back on your feet again.

What to Do After a Slip (page 4)

The good news is that slips usually do not ruin your chances of success or undo the progress that has been made. It simply means that more practice and learning are necessary.

[Review page 4 and include the following:]

First, **remember two things:**

- **Slips are normal and to be expected.**
  A slip doesn't need to lead to giving up completely. Slips can and should be useful learning experiences.

- **No one time of overeating or not being active, no matter how extreme, will ruin everything.** You won't gain more than a few pounds of weight even after the biggest eating binge imaginable--unless you *stay* off track and keep overeating time and time again.

So, after you have a slip:

1. **Talk back to negative thoughts with positive thoughts.**
   The negative thoughts that come after a slip can be your worst enemy. They can lead to feeling discouraged, guilty and angry and undermine your ability to handle the slip effectively. Talk back to the negative thoughts with positive ones.

2. Next, **ask yourself what happened.**
   Use the opportunity to look closely at the situation and ask yourself what happened. Was it a special occasion? If so, is it likely to happen again soon? Did you overeat in response to feeling lonely, bored, or depressed? Did you eat because of social pressure? Did you skip activity because you were too busy with other things, or because of work and family pressures? Use these questions to review the situation and think about it objectively.

Copyright 2017 University of Pittsburgh

Learn from the slip. Then you can plan a strategy for handling the situation better next time.

Can you avoid this situation in the future (for example, by not sitting near the food or by not walking past the candy machine)? If you can't avoid it, **can you manage it in a better way** (for example, by making sure you have low-calorie foods available at home)?

3. **Regain control the very next time you can.**
   Do **not** tell yourself, "Well, I blew it for the day," and wait until the next day to start following your healthy eating plan. **Make your very next meal a healthy one. Get back on schedule with your activity plan right away**. You will not have set yourself back very much if you follow this suggestion.

4. **Talk to someone supportive.** ("Talk it through, don't eat it through.")

5. Finally, **focus on all of the positive changes you have made** and realize that you can get back on track. The same person who "blew it" today is the same person who has been successful during many previous weeks. Slips do not reveal "real you" (hopeless, lacking willpower, etc.). They are simply another occasion of behavior. **Remember, you are making life-long changes. Slips are just one part of the process.**

Practice: Help Sadie Manage the Slippery Slope of Lifestyle Change (page 5)

[Ask six participants to each read a section out loud from Sadie's story. Initiate a group discussion.]

Can anyone relate to Sadie's story? How?

Now let's look at Sadie's story more closely.

[Ask the participant to reread card 1 and discuss the following:]

- This is a high-risk situation. Why? *[Answer: This is a change in her usual routine. She has a self-defeating towards taking a weekend off.]*

[Ask the participant to reread card 2 and discuss the following:]

- What happened? *[Answer: She has a self-defeating thought and she does not have a plan.]*
- Remember that GLB is a new healthier lifestyle, not a 'diet' that she is "on' or "off".
- Why is it important to plan ahead?
- What could Sadie have done differently?

[Ask the participant to reread card 3 and discuss the following:]

Copyright 2017 University of Pittsburgh

- What happened? *[Answer: She had a slip and gained weight.]*
- How did Sadie respond to the slip? *[Answer: She responded in a good way. She got back to working on her plan as soon as she got home and was able to lose the weight she gained.]*

[Ask the participant to reread card 4 and discuss the following:]
- What happened? *[Answer: She had self-defeating thoughts.]*
- What could she have done differently? *[Answer: Talk back with positive thoughts, use it as a learning experience and focus on the positive, all the weight she has lost.].*

[Ask the participant to reread card 5 and discuss the following:]
- What happened? *[Answer: She had another slip, is now experiencing a slide and avoiding group meetings.]*
- What could Sadie have done differently? *[Answer: She could talk to someone supportive, come back to group meetings right away to help refocus and to use the group support.]*

[Ask the participant to reread card 6 and discuss the following:]
- What is happening? *[Answer: She continues with a slip.]*
- What did she do to get back on her feet? *[Answer: She came back to group.]*

The Slippery Slope tends to happen in a chain of events. It is possible to break the chain in many places, but the earlier the chain is broken the better. This is the goal of problem-solving.

Slips from Healthy Eating/Slips from Being Physically Active (page 6)

You see from Sadie's example that it is possible to manage the slippery slope.
Now, it's time to apply what you learned to your own lifestyle efforts.

[If time allows, guide participants through the questions about slips. Ask participants to share their response, if comfortable doing so. If you don't have class time to complete this activity, suggest to the group that they should complete this page at home.]

Self-Defeating Thoughts (page 7)

Sadie had some self-defeating thoughts. Now we're going to talk in more detail about self-defeating thoughts.
Everyone has self-defeating thoughts at times. These thoughts **can lead you to overeat or be inactive**. Then afterwards you may **feel even worse** about yourself. A **vicious cycle of self-defeat** can result.

Copyright 2017 University of Pittsburgh

Let's look at the example on page 7. *[Review the example and include the following:]*

- This self-defeating thought might lead you to eat some potato chips.
- This is followed by another self-defeating thought. Next, you're discouraged and go on to eat more of them.

Sometimes we aren't aware we are having self-defeating thoughts. Self-defeating thoughts can become such a habit for most of us that we tend to believe and act on our self-defeating thoughts without even hearing them.

One goal of this session is to help you hear your self-defeating thoughts and teach you to talk back to them.

Let's look at some **common examples** of self-defeating thoughts.

[Review each category and the example(s), then ask a question or two to get the participants thinking about their own experience with self-defeating thoughts. Include the following information:]

1. **Good or Bad Thoughts**
    - Sometimes this is called "all or nothing" or "light bulb" thinking (either on or off) with nothing in between.
    - Do you have some foods you consider "good," and some foods you consider "bad?"
    - What happens when you eat a little of what you consider to be a "bad" food?
    - Can you think of some problems with considering a food "bad?"

2. **Excuses (or Rationalizations) Thoughts**
    - These thoughts blame something or someone else for your problems. You act as if they have so much power that you have no choice but to overeat or be inactive.
    - Can you think of a time when you bought some high calorie food "for someone else"? Did they really need the food, or do you think you used them as an excuse to buy the food for yourself?

3. **Should Thoughts**
    - These "should" thoughts assume that someone is standing over you, forcing you to do what you don't want to do.
    - What kind of things do you think you "should" or "should not" do to lose weight and be more active?
    - What do you expect yourself to do perfectly (for example, self-monitoring)?
    - What happens when you expect perfection of yourself? How do you feel? How does it affect your future decisions and choices?
4. **Not As Good As Thoughts**
    - These are thoughts when you compare yourself to someone else and then blame yourself for not measuring up.
    - Do you compare yourself to someone else? Who?

Copyright 2017 University of Pittsburgh

- How does comparing yourself to that person make you feel? How does it affect your decisions and choices about eating and being active?

5. **Give Up Thoughts**
   - These thoughts often follow a chain of other kinds of self-defeating thoughts.
   - Do you ever want something good to eat and think, "I'm sick of GLB and this healthy lifestyle stuff"?

   [Note: These are the thoughts that GLB most wants to help participants manage.]

How to Talk Back to a Self-Defeating Thought (page 8)

[Review the three steps on the top of page 8 and include the following information:]

Once you are aware of a self-defeating thought, you can "talk back to it." Here's how:

1. First, catch yourself having the self-defeating thought. Ask yourself, "Is this thought moving me forward or bringing me down?" As soon as you're aware of a self-defeating thought, say to yourself, "I'm doing it to myself."

2. Then imagine shouting "STOP!" to yourself. Picture a huge, red stop sign. *[You may want to hold up the STOP! sign prop at this point.]* The stop sign is so big that it takes up all the room in your mind. This should startle you and get rid of the self-defeating thought.

3. Talk back with a positive thought. No matter how well you've stopped a self-defeating thought, it will probably return again. It may be a habit for you. So, it's important to begin to build a new habit: positive thinking.

Let's look at some examples of how to "talk back" to a self-defeating thought.

> This session may be an appropriate time to review with participants the work sheet "Remember Your Purpose" (Session 1, page 4) on which they recorded their personal reasons for joining the program and so on. Details from this work sheet may provide images and words for the participants to use in talking back to negative thoughts with positive ones.
>
> Any imagery that is significant to the participants may help make the process of "talking back" more meaningful and fun; for example, some participants might find it enjoyable to imagine a devil on one shoulder and an angel on the other, and to see the task of positive thinking as, "letting the angel talk."

[Review the categories and the examples in the box on page 8 and include the following information:]

- **Good or Bad: Talk back with "Work Toward Balance".** Don't expect perfection of yourself, but don't indulge yourself either. Work toward an overall balance.

Copyright 2017 University of Pittsburgh

- **Excuses: Talk back with "It's Worth a Try".** Instead of looking for something or someone else to blame, why not give yourself a chance? Try something. You just might succeed.

- **Should: Talk back with "It's My Choice".** You are in charge of your eating and activity. No one else is responsible for your choices or standing over you with unrealistic expectations.

- **Not As Good As: Talk back with "Everyone is Different".**

- **Give Up: Talk back with "One Step at a Time".** Problem-solving is a process. It takes time to make life-long changes. Learn from what doesn't work and try another option. Learning is always a success.

Practice: Help Sam Talk Back (page 9)

Now let's practice by helping Sam to stop self-defeating thoughts and talk back with positive thoughts.

> Note: This activity may be done with the entire group or you by dividing the participants into several small groups. If time is short, encourage participants to work on this page at home.
>
> Review the directions.
>
> First, say the negative thought aloud. Then say "Stop!" Use a stop sign prop if you find it helpful.
>
> Generate examples of positive thoughts. Ask participants to record the positive thoughts on page 9.

[Emphasize the "bottom line" message at the bottom of the page.]

Checking In On the Pedometer (page 10)

Last week you received your pedometer. You were asked to wear it every day last week and then calculate your average steps per day.

What were your average steps per day? Write it in the box on page 10.

Now you can look at your daily step count average in light of national averages and the GLB step goal.

Copyright 2017 University of Pittsburgh

[Review the information on page 10 and include the following information.]

To give you some perspective, the average adult tends to get about 5,000 steps per day or 35,000 steps for the week.

In GLB, your goal is to **slowly increase your steps to an average of about 7,000 steps per day or 50,000 steps for the week** and to maintain this level. This goal was also used in the DPP.

Don't forget to keep recording your activity minutes per week. Reaching your goal for activity minutes each week will help you reach your goal for steps. The two work hand in hand.

As a best guesstimate, someone who is doing 150 + minutes of moderate intensity physical activity per week and is not completely inactive in their normal day will achieve about 50,000 steps for the week.

To Do (page 11)

Turn to page 11 and let's focus on what you can do between now and the next session.

Please continue to keep track of your weight, eating, and activity minutes. Come as close as you can to your calorie, fat gram, and activity goals. *[Remind participants that the GLB activity goal is to reach and maintain at least 150 minutes per week.]*

New things to practice:

To help you practice what you learned today, let's apply what you learned about slips and self-defeating thoughts to your lifestyle change efforts.

First, between now and the next session, catch yourself thinking self-defeating thoughts and record them. Practice stopping them and talking back with positive thoughts. Notice any change in how you thought, felt, or acted. You can share your experience/feedback next week.

In addition, try to apply what you learned about slips. If you have a slip this week, use the worksheet on page 6 to make an action plan. Or think of something that has caused a slip in the past. Follow your plan. You can share your experience/feedback next week. *[If time allows, participants can start working on the worksheet on page 6.]*

[Review the step goal that is adding steps slowly and safely.]

[Note: This is the standard GLB step progression. Many participants will be able to comply. However, you should make exceptions for individuals with serious health

problems that limit their mobility. For them, they should start where they are and gradually increase as much as they can.]

Session 9: Resources (pages 12-22)

Page 12 lists the additional resources.

Page 13 is the BMI chart discussed earlier in this session.

Pages 14-22 provide information about how to build a better" breakfast, lunch, dinner, and snack. These pages will be helpful as you continue to shift to healthier food choices and a healthy eating pattern.

[Announce the day, time, and place for the next session.]

After the session:

- Weigh participants who did not do so prior to the group meeting.
- Complete data forms and documentation required in your setting.
- Follow your program's protocol for managing absences.
- Review the self-monitoring records from the previous week. Write brief comments. Be positive and nonjudgmental.
    - Praise all efforts to self-monitor and to change eating and activity behaviors.
    - Highlight especially any positive changes made that relate to the session topic of the week before the records were collected: recording RPE, wearing the pedometer and recording steps per day and weekly average, coming close to their calorie, fat gram, and activity goals.
    - Refer to **Guidelines for Reviewing Food and Activity Records** available in the GLB Providers Portal.

Copyright 2017 University of Pittsburgh

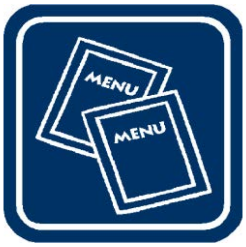

Session 10:
Four Keys to Healthy Eating Out

Not every meal will be prepared and eaten at home. It is important to develop skills and have a plan for healthy eating out.

1. Plan ahead.

- Carefully select where you will eat. Choose places that offer lower calorie healthy options.
- Start thinking about what you might order. Before you go, view the menu and nutrition information on the restaurant's website, look at a calorie and fat counter, or call ahead to ask about the menu.
- Eat fewer calories during other meals and snacks that day.
- Eat a small healthy snack before you go out. Or drink a large, calorie-free or low-calorie beverage.
- If available at the restaurant, use the nutrition information listed on the menu, menu board, or displays to help you make healthy choices.
- You may want to limit alcohol. Alcohol adds calories, stimulates the appetite, and may make it harder to stick to your plan.
- For parties: Bring a healthy, low-calorie food to share with others.

2. **Don't be afraid to ask for what you want**. Be firm and friendly.

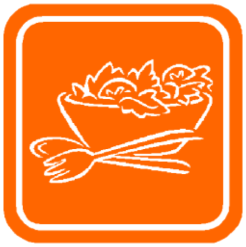

Ask for the foods you want:

- Ask for lower-calorie foods.
- Ask if foods can be cooked in a different way.
- Don't be afraid to ask for foods that aren't on the menu.

Ask for the amounts you want:

- Ask how much is usually served.
- Order salad dressing, gravy, sauces, or spreads "on the side."
- Ask for less cheese or no cheese.
- Split a main dish or dessert with someone.
- Order a small size (appetizer, senior citizen's, children's size).
- Have the amount you don't want to eat put in a container to take home.

Copyright 2017 University of Pittsburgh

How to Ask For What You Want

Ask for what you want:

- Begin with "I," not "You."
- Use a firm and friendly tone of voice that can be heard.
- Look the person in the eye.
- Repeat your needs until you are heard. Keep your voice calm.

Wishy-washy	"Oh, well. I guess they couldn't broil the fish."
Threatening	"You said you would broil my fish! I'm not paying for this!"
Firm and friendly	"This looks very nice. But I asked for my fish to be broiled, not fried. Would you have some broiled for me, please?"

Copyright 2017 University of Pittsburgh

3. Take charge of what's around you.

- If you can, be the first to order.
- Keep foods off the table that you don't want to eat. Or take one serving and then ask that the item be removed from the table.
- Ask that your plate be removed as soon as you finish eating.

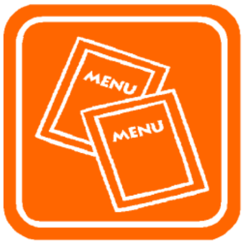

4. Choose foods carefully.

Watch out for these high-calorie words on menus:

- Au gratin
- Hollandaise
- Breaded
- Parmesan
- Buttered or buttery
- Pastry
- Cheese sauce
- Rich, creamed, creamy, in cream sauce
- Sautéed
- Stuffed or loaded
- Fried, deep fried, French fried, batter fried, pan fried
- Scalloped
- Smothered
- Southern style
- Gravy

These words may or may not mean the items are low in calories:

- Baked
- Broiled
- Boiled
- Grilled
- Rubbed, blackened
- Poached
- Steamed
- Roasted
- Stir-fried
- Spiced, seasoned

Copyright 2017 University of Pittsburgh

What's on the Menu?

You can find lower-calorie choices wherever you eat out. Be sure to ask the waiter how the food is prepared.

Go means lower-calorie choices. Caution means high-calorie choices.	
**Pizza**	
Go	Plain cheese pizza (ask for half the cheese or low-fat cheese), onions, green peppers, mushrooms
Caution	Meat toppings (sausage, pepperoni, ham, bacon)
**Burger Place (fast food)**	
Go	Grilled, broiled, or roasted chicken, without skin or sauce; broiled, extra lean burger
Caution	Large hamburger, cheeseburger, French fries, fried fish or chicken, mayonnaise-based sauces, cheese, bacon
**Mexican**	
Go	Heated (not fried) tortillas, grilled chicken or beef fajitas, soft tacos (corn or flour tortillas), salsa
Caution	Enchiladas, chili con queso, fried tortillas, tortilla chips, sour cream, crisp tacos
**Chinese and Japanese**	
Go	Stir-fried chicken, stir-fried vegetables, steamed rice, soup, teriyaki
Caution	Egg foo yung, fried chicken, beef, or fish, fried rice or noodles, egg rolls, fried won ton, tempura
**Italian**	
Go	Spaghetti with tomato sauce, minestrone soup
Caution	Lasagna, manicotti, other pasta dishes with cheese or cream, sausage, fried or breaded dishes (such as veal or eggplant parmesan)
**Seafood**	
Go	Broiled, baked, or boiled seafood with lemon, plain baked potato
Caution	Fried fish, fried vegetables, French fries
**Steakhouses**	
Go	Shrimp cocktail, broiled/grilled chicken or fish, plain baked potato
Caution	Steak (except for lean, trimmed cuts), fried fish or chicken, onion rings, other fried vegetables, French fries

Copyright 2017 University of Pittsburgh

Fast Food *Can* Be Lower in Calories and Fat

The choices below contain less than 370 calories and 15 grams of fat per serving. Values are from restaurant websites (2017).

Food Item	Calories	Fat grams
**Arby's®**		
Farmhouse Salad w/Roast Turkey, Light Italian Dressing	250	14
Turkey and Cheese Slider	200	7
Boston Market®		
Fresh Steamed Vegetables	60	3.5
Rotisserie Potatoes	120	2.5
Turkey Breast, Regular	160	4.5
Burger King®		
Regular hamburger	220	8
Tendergrill® Chicken Sandwich, no mayonnaise	320	6
BK Veggie® Burger, no mayonnaise	310	7
**Domino's Pizza®**		
1/6th 10-inch Hand-Tossed Cheese Pizza	200	7
KFC®		
Green Beans	25	0
Grilled Chicken Breast	210	6
**McDonald's®**		
Fruit and yogurt parfait	150	2
Egg White Delight McMuffin	260	8
Southwest Grilled Chicken Salad	350	9
Hamburger (single)	250	8
Subway®		
6″ FRESH FIT sandwich, no cheese/dressing (Turkey, Roast Beef, Subway Club®, or Oven Roasted Chicken)	280-320	3.5-5
Black bean soup	210	1
**Wendy's®**		
Large Chili	250	7
Power Mediterranean Chicken Salad, Half Size	250	9
Grilled Chicken Go Wrap	270	10
Junior hamburger	240	14
Grilled chicken sandwich	360	8

Copyright 2017 University of Pittsburgh

Practice:
Eating at Fast Food Restaurants

Can you eat low-calorie, healthy meals when
you eat out at fast food restaurants?

Answer the questions below.

1. What problems and challenges might get in the way?

____

____

____

____

____

2. What could you do to overcome those problems and challenges?

____

____

____

____

____

Copyright 2017 University of Pittsburgh

Practice: Eating at Restaurants (not fast food)

Can you eat low-calorie, healthy meals when you eat out at restaurants (not fast food)?

Answer the questions below.

1. What problems and challenges might get in the way?

____

____

____

____

____

2. What could you do to overcome those problems and challenges?

____

____

____

____

____

Copyright 2017 University of Pittsburgh

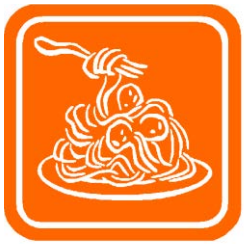

Practice:
Eating at Banquets and Buffets

Can you eat, low-calorie, healthy meals when you eat out at banquets and buffets (such as church dinners, wedding receptions, business events, and reunions)?

Answer the questions below.

1. What problems and challenges might get in the way?

____

____

____

____

____

2. What could you do to overcome those problems and challenges?

____

____

____

____

____

Copyright 2017 University of Pittsburgh

Practice: Eating at the Homes of Family and Friends

Can you eat low-calorie, healthy meals when you eat out at the homes of family and friends?

Answer the questions below.

1. What problems and challenges might get in the way?

____

____

____

____

____

2. What could you do to overcome those problems and challenges?

____

____

____

____

____

Copyright 2017 University of Pittsburgh

To Do:

Check the boxes when you complete each item:

☐ Keep track of your weight. Weigh yourself at home at least once a week. Record it.

☐ Record everything you eat and drink every day. Come as close as you can to your calorie and fat gram goals.

☐ Record your minutes of physical activity. Come as close as you can to the GLB goal of at least 150 minutes per week.

New things to practice:

☐ Work toward solving an eating-out problem.

- Describe a problem you have when you eat out:

  ____

- Make and follow a Positive Action Plan:

I will: ____

When: ____

I will do this first: ___________________________________

Roadblocks that might come up: __________________________

I will handle them by: _________________________________

I will do this to make my success more likely: ______________

How can I get the support I need? ________________________

Did it work? If not, what went wrong? What could you do differently?

____

☐ Add at least 250 steps per day.

Last week, what was your average step count per day? _________

_________________ plus 250 steps = ____________________

Average steps per day (last week) — Daily step goal (next week)

Copyright 2017 University of Pittsburgh

Session 10 Leader Guide:
Four Keys to Healthy Eating Out

Objectives:
In this session, the participants will:

- Learn four basic principles for healthy eating out: **plan ahead, ask for what you want, take charge of what's around you, and choose foods carefully.**
- Identify specific examples of how to apply these principles when eating away from home.
- Learn the importance of having a plan when eating away from home.
- Identify barriers to healthy eating when eating out and problem-solve ways to overcome them.
- Learn how to gradually and safely increase steps using the pedometer.

To Do Before the Session:

- If providing Food and Activity books; one per participant.
- Review Food and Activity records from last session; add comments.
- Prepare Session 10 handout for participant notebook.
- Optional: Bring menus from local restaurants. Or print menus from restaurant websites. Choose restaurants that your participants frequent.
- Optional: Decide if you want participants to invite family members to attend the next meeting on social cues. Limit the number of guests each participant may invite.

Available in the GLB Providers Portal:
https://www.diabetesprevention.pitt.edu/my/login.aspx

- **Guidelines for Reviewing Food and Activity Records**

Group Sharing Time (allow at least 5 minutes)

In the last session, you learned about the slippery slope of lifestyle change and how to respond to a slip. We also talked about self-defeating thoughts and how these can impact your lifestyle change efforts.

- Did anyone have a slip and change how they responded to the slip? If so, please share your experience with us.
- Were you more aware of your thoughts this past week? Did anyone catch themselves having a self-defeating thought, visualize the "stop" sign and say "stop!", and turn it into a positive thought? If so, please share your experience.

Copyright 2017 University of Pittsburgh

Let's take a few minutes to talk about your experiences this past week. *[Choose only a few of the following prompts to guide discussion.]*

- Overall, how did it go last week? What went well? What problems did you have? What could you do differently?
- What change did you make in your eating pattern this past week that you are most proud of?
- Were you able to reach your goal for minutes of planned activity during the week? How did it go? What did you learn?
- Did you wear your pedometer? How did it go? Did it help you to move more in your daily life? Did it help you reach your activity goal? What are some things you did this past week to add steps?
- Were you able to use the RPE scale to estimate how hard you were working during exercise? Please share your experience.

[Problem solve with the participants to address any barriers.]

[Praise all efforts to self-monitor and to change eating and activity behaviors. Be positive and nonjudgmental. Encourage group discussion.]

Session 10: Four Keys to Healthy Eating Out (pages 1-3)

Raise your hand if you have eaten every meal at home since you started this program.

[Use this question to begin the conversation about making healthy choices when eating out. Generally, few participants will respond that they have never eaten out at some point during the program. Point out that it is unrealistic to imagine that they will never eat out: eating out is fun part of life, and can fit into a healthy lifestyle.]

Name some of the places where you eat out.

Do you find it easier to stay within your calorie and fat gram budget when eating at home or away from home? Why?

Today we're going to talk about eating away from home.

[Review the four keys and include the following information:]

There are four basic keys to healthy eating out. *[First, indicate each of the major headings on pages 1 and 3. Then come back and review the specific points under each heading.]*

1. Plan ahead

- The more planning ahead that occurs, the easier it is to make healthy food choices and to stay close to your calorie and fat gram goals.

Copyright 2017 University of Pittsburgh

- Having a plan will help you to anticipate any problems and handle them more easily. You won't run into so many surprises.
- The next three keys will help you carry out your plan.
- Have you used restaurant websites to find nutrition information? A calorie and fat counter? How did this work for you?
- Sometimes people wait until they get home and then look up the calories and fat in what they ate and are frustrated and disappointed when they see the totals. They often say that they would have made different food choices if they had looked at the calorie information ahead of time. Has this ever happened to you?
- Do you think it would be a good idea to skip breakfast and lunch in order to spend all your calories when going out to dinner? Why or why not? *[Answer: This is not a good idea. You would probably be so overly hungry that it would be difficult to stay within your calorie goal.]*
- Have you noticed the nutrition information listed on menus, menu boards, or displays in restaurants and places that sell prepared foods? Have you used this information to help you make healthy food and beverage choices?
- If you have tried any of these strategies, please share your experience.

Federal regulations are pending that will require places that sell prepared foods and have 20 or more locations to post the calorie content of food on their menus, menu boards, and displays. This will apply to restaurants, supermarkets that sell prepared foods, convenience stores, bakeries, coffee shops, and movie theaters.

2. **Don't be afraid to ask for what you want. Be firm and friendly**.
   - Take steps to make what's around you support you and your efforts to make healthy food choices and to stay within your calorie and fat gram goals. Try to get rid of the things that can get in the way, if you can.
   - Psychologists call this "assertiveness".
   - Ask for both the foods you want and the amounts you want. Remember, you are paying for the meal. There is a lot of competition for your restaurant dollars. Restaurants are used to special requests and usually will do all they can to honor these requests. They want you to be happy and to come back. Has anyone ever made a special request? How did it go?
   - Are there any other suggestions? Examples include:
     - Ask for foods that aren't on the menu. For example, if fresh fruit is on the breakfast menu, it may be available as a dessert for dinner.
     - Ask how much is served. Restaurants know how many ounces are in their servings of meat, fish, and poultry. They have this information for portion and cost control.

[Encourage group discussion about how to ask for the foods you want and the amounts you want.]

Some people find it hard at first to ask a waiter or waitress for something special. With practice, it gets easier. Page 2 gives a mini assertiveness training session.
Here are some tips to help you ask for what you want:

Copyright 2017 University of Pittsburgh

[Review page 2 and include the following:]

- **Begin with "I", not "You."**
  Using "I" statements shows that you take responsibility for your own feelings and desires. "I would like my chicken broiled with lemon juice instead of butter," or "I would like a tossed salad with dressing on the side instead of coleslaw, please."

  "You should have," "you said," "you don't understand." Using "you" often puts others on the defensive. "You didn't put the salad dressing on the side!" Better: "I asked to have the salad dressing on the side, please."

- **Look the person in the eye.**
  Eye contact says a lot. Avoiding eye contact often appears as if you don't believe what you are saying.

- **Repeat your needs until you are heard. Keep your voice calm.**
  Sometimes it may take several tries before you are understood. If you need to repeat yourself, keep your voice low but firm. A loud voice can be threatening to others.

[Encourage group discussion about experiences with "asking for what you want".]

[Review page 3 and include the following:]

3. **Take charge of what's around you.**
   - Why do you think it's a good idea to be the first to order when eating out with others? *[Answer: You won't be tempted by what or how much others order, and they may follow your healthy example. You may also set a healthy example for your fellow diners.]*

4. **Choose foods carefully.**
   - You can tell a lot from the words on a menu. Sometimes it's easy to tell if something is going to be high or low in calories. For example, you know by now that a vegetable with cream sauce is going to be higher in calories than a steamed vegetable.
   - Ask the waiter if you're not sure how something is prepared.

What's on the Menu (page 4)

Most restaurants have lower-calorie choices on their menu.

[Encourage participants to read this page at home.]

Copyright 2017 University of Pittsburgh

Fast Food Can be Lower in Calories and Fat (page 5)

If you choose to eat at a fast food restaurant, it is possible to stay within your calorie goals for the day. This page gives some examples from various fast-food restaurants.

You can look at this page more closely at home.

Fast food restaurants typically have nutrition information on their websites. Make a plan of what to order before you go. Have you ever done this? How did it work for you?

Fast food restaurants with 20 or more locations usually post the nutrition information on menu, menu boards, or displays. Have you used this information to help you make healthier food choices?

*[Note: This handout is **not** meant to suggest that GLB recommends eating in fast food restaurants; rather it is meant to be a guide for when participants do choose to eat in this type of establishment.]*

Practice (pages 6-9)

Now let's see how you can use this information.

Activity:

- Divide the class into 4 groups
- Assign each a different eating out situation: Fast Food restaurant (page 6), Restaurant that is not fast food (page 7), Banquets and Buffets such as wedding receptions, church dinners, or reunions (page 8), and Homes of family and friends for a party or a meal (page 9).
- Each group should discuss the challenges or barriers. Then discuss how to overcome these problems or challenges. They may include strategies they have tried or might want to try. Ask 1 person in each group to be the "recording secretary".
- Allow time for the groups to work.
- Come together as a large group.
    - Ask the Fast Food group share their list of barriers to healthy eating.
    - Ask others in the class to add to the list.
    - Acknowledge that eating away from home can present some barriers to healthy eating. But it is possible to overcome these barriers.
    - Ask the Fast Food group to share their list of ways to overcome these barriers to healthy eating.
    - Ask others in the class to add to the list. They can suggest things they have done or could do to make healthy food choices.
    - Repeat this process with the other three small groups.

- Mention that buffets are challenging because of all the food cues. As we discussed in **Session 6, Take Charge of What's Around You**, seeing food is a powerful cue to eat when you're not hungry, overeat, or make unhealthy food choices. This is why

Copyright 2017 University of Pittsburgh

restaurants often have a dessert tray so you can see all the choices. This is also why restaurants often have table tents that feature pictures of desserts and food pictures in the menu.

- Remind participants that alcohol increases appetite and lower self-control. Ask participants to share what they have done to help to limit liquid calories from alcohol.

We have talked about how important it is to make a plan if you know you are going to eat away from home.

But what if it is unexpected? If you keep a running subtotal of calories and fat grams in your Food and Activity record, you will know exactly how many calories you have left to spend if eating out is an unplanned event.

Some people report that they find it helpful to eat out less often when they are starting GLB. This allows time to learn information and practice skills and behaviors at home first. What are your thoughts about this?

GLB is NOT a diet program that you are "on" or "off". It is a healthy lifestyle. You can use the 4 keys to healthy eating out to develop skills and strategies to make it easier to eat a healthy diet when eating out.

Have you ever eaten away from home and felt that you made healthy food choices and stayed within your calorie and fat gram goals? How did that make you feel*? [Examples: It motivates me to continue making healthy changes in my eating behaviors, I feel proud of my efforts, and it shows me that I can eat away from home and enjoy the food and company and choose healthy food.]*

It is possible to eat out, enjoy the food and the company, and still eat a healthy meal. Eating out doesn't have to be an excuse for overeating. Eating out is part of life, so it is important to develop strategies and skills to make it easier to choose healthy meals while eating away from home.

Optional: If time allows, give participants menus from local restaurants.

- Ask participants to review the menu and choose a healthy dinner that would fit into their calorie goals. Without using nutrition information from the restaurant or a Calorie and Fat Counter, select a meal that is about 400-600 calories.
- Distribute nutrition information from each restaurant. Ask participants to quickly look for high and low calorie/fat items. Participants may identify changes they would make to their original "order" to lower the calorie and fat content.
- Remind participants that nutrition information is available online. Encourage them to plan their menu choices and calculate the calories and fat grams before they go to the restaurant. They can also look on the menu, menu board, or display for nutrition information, if available.

Copyright 2017 University of Pittsburgh

To Do (page 10)

Turn to page 10 and let's focus on what you can do between now and the next session.

Please continue to keep track of your weight, eating, and activity minutes. Come as close as you can to your calorie, fat gram, and activity goals.

New things to practice:

Describe a problem you have when you eat out. *[Give participants time to record the problem on page 10.]*

Choose one of the four keys to healthy eating out that is likely to help you solve the problem and that you can do. Make a positive action plan. *[If time permits, give the participants time to complete the Positive Action Plan.]*

Between now and the next session, **follow your action plan** and answer "Did it work?" You can share your experience/feedback next week.

Continue to wear your pedometer and track your steps. *[Encourage participants to look at their average steps per day from last week and to find ways to add 250 more steps/day.]*

[Note: This is the standard GLB step progression. Many participants will be able to comply. However, you should make exceptions for individuals with serious health problems that limit their mobility. For them, they should start where they are and gradually increase as much as they can.]

[Announce the day, time, and place for the next session.]

[If appropriate in your setting, ask the participants to invite family members to the next session, Make Social Cues Work for You. Participants may find this helpful in planning strategies for handling social cues.]

After the session:

- Weigh participants who did not do so prior to the group meeting.
- Complete data forms and documentation required in your setting.
- Follow your program's protocol for managing absences.
- Review the self-monitoring records from the previous week. Write brief comments. Be positive and nonjudgmental.
  - Praise all efforts to self-monitor and to change eating and activity behaviors.
  - Highlight especially any positive changes made that relate to the session topic of the week before the records were collected: handling slips and self-defeating thoughts, adding steps, coming close to their calorie, fat gram, activity, and step goals.
  - Refer to **Guidelines for Reviewing Food and Activity Records** available in the GLB Providers Portal.

Copyright 2017 University of Pittsburgh

Session 11:
Make Social Cues Work *for* You

What other people say or do may have a big impact on your eating and physical activity. These are called social cues.

Problem Social Cues:	Examples:
The sight of other people eating problem foods or being inactive.	
Being offered (or pressured to eat) problem foods or invited to do something that involves sitting for a long time.	
Being with peers who are rarely active.	
Being nagged.	
Hearing complaints.	

Helpful Social Cues:	Examples:
The sight of other people eating healthy foods or being active.	
Being offered healthy foods or invited to do something active.	
Being praised.	
Hearing compliments.	

When you respond to a social cue in the same way, you build a habit.

The other person has *also* learned a habit. This makes social cues even harder to change than other cues.

Copyright 2017 University of Pittsburgh

To Change Problem Social Cues:

1. Stay away from the cue, if you can.

   - Example: Move to a different room.

2. Change the cue, if you can.

   - Discuss the problem with the other person.
   - Brainstorm options.
   - Tell the other person about GLB and your efforts to eat a healthier diet and be more active to lose weight and improve health.

> Ask others to:
>
> - Praise you for your efforts and
> - Ignore your slips.
>
> This is KEY to your success.

3. Practice responding in a healthier way.

   - Say "No" to food offers.
   - Show others you know they mean well.
   - Suggest something they can do to help you.
   - *Example:* "No, thanks. But I'd love a glass of ice water."

Remember, it takes time to change habits.

Copyright 2017 University of Pittsburgh

To Add Helpful Social Cues:

- Spend time with people who are active and make healthy food choices.
- Put yourself in places where people are active.
- Set up a regular "date" with others to be active.
- Ask your friends to call you to remind you to be active or to set up dates to be active.
- Bring a low-calorie food to share when you go to a party.
- When it's appropriate, be the first to order when you eat out at a restaurant.
- Be social by doing something active. Take a walk and talk.
- What are some other social cues you might find helpful?

Copyright 2017 University of Pittsburgh

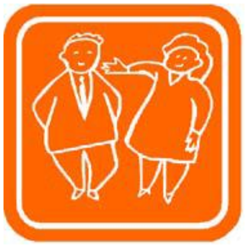

What Social Support Do You Need?

Research shows that people are better able to reach their eating and activity goals if they have some type of social support in place. What support do you need?

For healthy eating: ____

For being more active: ______________________________________

What could others do to help you? Here are some ideas. Check the ones that are important to you. You may need to teach your family and friends how to support your efforts.

Ways to help me eat healthy:	Ways to help me be more active
☐ Serve low-calorie foods for meals.	☐ Go for a walk with me. Or do other physical activities with me.
☐ Eat low-calorie foods when I'm nearby.	☐ Plan social events around being active.
☐ Don't tempt me with problem foods as a reward or gift.	☐ Compromise when my being active conflicts with your schedule.
☐ Clear the table and put food away as soon as the meal is over.	☐ Praise me when I do my scheduled activity. Don't remind me when I don't.
☐ Help with cooking, shopping, or cleaning up after meals.	☐ Do one extra household chore to allow time for me to take a walk.
☐ Don't offer me second helpings	☐ Set up a regular date with me to be active.
☐ Encourage me to cook or try new foods.	☐ Encourage me to go out for a walk when I'm debating whether or not to go.
☐ Praise my efforts to eat healthier foods.	☐ Try to achieve and maintain the GLB goals with me.
☐ Other:	☐ Other:

Copyright 2017 University of Pittsburgh

Social Cues are Powerful at Social Events

Social events:

- Upset our routine.
- Challenge us with unique food and social cues.
- May involve habits that have developed over many years and so can be very powerful.

To handle social events, problem solve. Brainstorm your options.

Options:	Examples:
Plan Ahead.	■ Eat something before an event.<br>■ Plan your meal in advance. Budget your calories for the day.<br>■ Plan to eat the best (in small portions) and leave the rest.<br>■ Bring a tasty, low-calorie dish to share.
Stay Away from Problem Cues.	■ Stand as far away as you can from the table with the food. Keep your hands busy with a glass of water, coffee, tea, or calorie-free beverage.<br>■ Watch the alcohol. It lowers your willpower and increases appetite.<br>■ Clear the table as soon as possible. Put the food away.
Change Problem Cues.	■ Discuss your goals with your family, friends, guests, host or hostess.<br>■ Ask others to praise your efforts and ignore your slips.
Respond to Problem Cues in a Healthier Way.	■ Practice a polite, but firm, "No, thank you."<br>■ Suggest something else they can do to help you.
Add Helpful Cues.	■ Serve healthy foods or bring some to share.<br>■ Use low-calorie ingredients or cooking methods to lower the calories in favorite recipes.<br>■ Try some new, low-calorie recipes.<br>■ Ask a friend or family member for support (split dessert with you, take a walk together, offer you healthy food choices).<br>■ Plan things to do that are active and don't involve food.

Copyright 2017 University of Pittsburgh

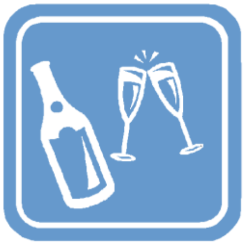

Describe a Social Cue **That's a** Problem for You

Pick one idea from this session for changing that social cue. Choose one that is likely to work and that you can do.

Make a positive action plan.

I will: ____

When? ____

I will do this first: ____________________________________

Roadblocks that might come up	I will handle them by:
_______________________	_____________________
_______________________	_____________________
_______________________	_____________________

I will do this to make my success more likely: ________________

____

____

How can I get the support I need? _________________________

____

____

Copyright 2017 University of Pittsburgh

Positive Social Cues You Would Like to Add

Pick one idea from this session for adding that social cue. Choose one that is likely to work and that you can do.

Make a positive action plan.

I will: ____

When? ____

I will do this first: ____________________________________

Roadblocks that might come up:	I will handle them by:
______________________	______________________
______________________	______________________
______________________	______________________

I will do this to make my success more likely:

____

____

____

How can I get the support I need?

____

____

____

Copyright 2017 University of Pittsburgh

To Do:

Check the boxes when you complete each item:

☐ Keep track of your weight. Weigh yourself at home at least once a week. Record it.

☐ Record everything you eat and drink every day. Come as close as you can to your calorie and fat gram goals.

☐ Record your minutes of physical activity. Come as close as you can to the GLB goal of at least 150 minutes per week.

New things to practice:

☐ Try my two action plans for making social cues work for me.

Answer these questions:

Did my action plans work?

____

If not, what went wrong?

____

____

What could I do differently next time?

____

____

☐ Add at least 250 steps per day.

Last week, what was your average step count per day? _________

__________________ plus 250 steps = _____________________

Average steps per day (last week) — Daily step goal (next week)

Copyright 2017 University of Pittsburgh

Session 11: Resources — Page

Getting Ready for the Holidays	10
Staying Active on Holidays	11
Lifestyle Balance on Vacation	12
Lifestyle Balance on Vacation: Problem Solving	13
Staying Active on Vacation	14

Copyright 2017 University of Pittsburgh

Getting Ready for the Holidays

Planning ahead is the key to staying active and eating healthy during the holidays.

1. Take time to think about potential problems in advance.
2. Write down the problem. List some possible solutions.
3. Choose the best strategy. Write it down.
4. Plan to reward yourself. Write down what you will do to earn the reward. Plan a non-food reward.

Ten Ways to Get Ready for the Holidays

1. Plan pleasures other than food or drink.
2. Hold a family conference well before the holidays.
    - Are we going to have tempting high-calorie foods in the house?
    - Get the family to agree not to nag you about what you eat or your activity plan.
    - Are there positive ways they can help?
    - What changes can be made to the holiday menus?
3. When you decide to eat a special high calorie food, choose quality, not quantity.
4. Plan ahead to be assertive with hosts. Let hosts know, in a clear and direct way, what you are planning to eat and not eat.
5. Eat something before you go to a party.
6. Decide what you want to do about alcohol. Alcohol increases appetite. It also lowers self-control. If you drink, are you aware of the calories involved?
7. Beware of fatigue, rushing, and tension. Look for early warning signals. Plan nonfood ways to cope, including exercise. Also, make plans to simplify.
8. Prepare for old friends or relatives you see only at holidays.
9. Plan for other pleasures *after* the holidays.
10. Make *reasonable* New Year's resolutions.

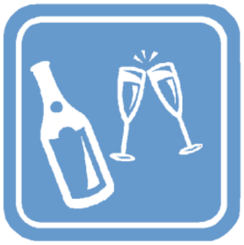

Copyright 2017 University of Pittsburgh

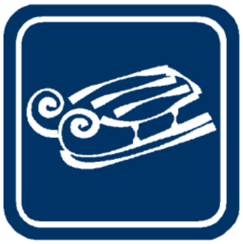

Staying Active on Holidays

For most of us, staying active on holidays isn't easy. Holidays upset our routine. They also challenge us with unique social cues. What's more, holidays may involve inactive habits that have developed over many years.

The key to staying active on holidays is planning.
Take a minute to think ahead about your next holiday.

- Where will you be?
- What might the weather be like?
- What days and times of the day will you have available for exercise?
- Are there nearby places to exercise?
- Who will you be with? Are they supportive of your being active?
- What kind of "holiday mind" thoughts might get in the way of staying active (e.g., I'm off from work now)?
- If you are traveling, what gear or clothing will you need to pack?
- How can you make staying active extra fun and festive?

I will: ____

When? ____

I will do this first: ____________________________________

Roadblocks that might come up: I will handle them by:

________________________ ______________________

________________________ ______________________

________________________ ______________________

I will do this to make my success more likely: ________________

____

Copyright 2017 University of Pittsburgh

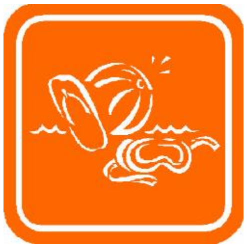

Lifestyle Balance on Vacation

Ways to Handle Vacations

1. Plan pleasures other than food or drink.
   - Plan the kind of vacation *you* want.
   - Think about what you like to do for physical activity. Can you make that a part of your vacation?

2. Hold a family meeting ahead of time.
   - What did we like or dislike about our last vacation? What will we do this time? How will we handle food and eating out?
   - Can we find a fun way to all be physically active this vacation? Get the family to agree not to nag you about your eating or activity. Talk about ways your family can help.

3. Have reasonable expectations for your vacation.
   - Keep track of your eating and physical activity.
   - Weigh yourself often. But remember, scales differ. Plan to *maintain* your weight.
   - Let go a little. Eat something special. (Choose the best. Have a small portion.) Get extra activity.

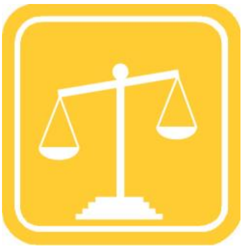

4. If you tend to be tense on vacation:
   - Avoid long periods when you're doing what others want and not what you want.
   - Plan daily times to relax.
   - Plan regular breaks while driving.

5. Decide what you want to do about alcohol.
   - Alcohol may lower self-control. If you drink, be aware of the calories.

6. Balance rest with physical activity.
   - Look for ways to be active that you enjoy.

7. Prepare **for friends or relatives you haven't seen in a while.**

8. Plan for pleasures *after* your vacation.

Copyright 2017 University of Pittsburgh

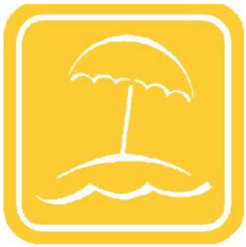

Lifestyle Balance on Vacation: Problem Solving

Healthy eating and being active *are* possible on vacation.
The key is to plan ahead:

1. What problems might come up?

____

____

2. Choose one problem. List some possible solutions.

____

____

3. Choose the best solution. What will you need to do to make that happen? How will you handle things that might get in the way?

____

____

4. Plan to reward yourself. What will the reward be? What will you need to do to earn that reward?

____

____

____

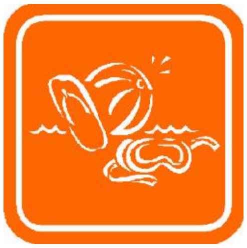

Copyright 2017 University of Pittsburgh

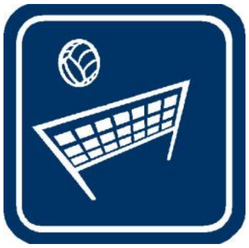

Staying Active on Vacation

Vacation means freedom from the demands of work and home life. So, it's easier to be active, right? Not always. The change of routine and surroundings can bring new challenges.

For most people, the key to staying active on vacation is planning.

Take a minute to think ahead about your next vacation.

- Where will you be?
- What might the weather be like?
- What days and times of the day will you have available for exercise?
- Are there nearby places to exercise?
- Who will you be with? Are they supportive of your being active?
- What kind of "vacation mind" thoughts might get in the way of staying active (e.g., I'm off now)?
- If you are traveling, what gear or clothing will you need to pack?
- How can you make staying active extra fun?

I will: ____

When? ____

I will do this first: ____

Roadblocks that might come up	I will handle them by:
__________________________	________________________
__________________________	________________________
__________________________	________________________

I will do this to make my success more likely: _________________

____

Copyright 2017 University of Pittsburgh

Session 11 Leader Guide:
Make Social Cues Work *for* You

Objectives
In this session, the participants will:
- Review examples of problem social cues and helpful social cues.
- Discuss ways to change problem social cues and add helpful ones.
- Review strategies for coping with social events such as parties, vacations, having visitors, and holidays.
- Make an action plan to change a problem social cue and add a helpful one.
- Learn how to gradually and safely increase steps using the pedometer.

To Do Before the Session
- If providing Food and Activity books; one per participant.
- Review Food and Activity records from last session; add comments.
- Prepare Session 11 handout for participant notebooks.

Available in the GLB Providers Portal:
https://www.diabetesprevention.pitt.edu/my/login.aspx
- **Guidelines for Reviewing Food and Activity Records**

Group Sharing Time (allow at least 5 minutes)

In the last session, we talked about the four keys to healthy eating out.
- Did anyone put into practice any of the four keys? How did it go?
- Did you work toward solving an eating-out problem? Did it work? If not, what went wrong? What could you do differently?

Let's take a few minutes to talk about your experiences this past week. *[Choose only a few of the following prompts to guide discussion.]*
- Overall, how did it go last week? What went well? What problems did you have? What could you do differently?
- Were you able to stay close to your calorie and fat gram goals? Follow your plan for physical activity, including lifestyle activity? *[Remind participants to graph their activity if they have not yet done so.]*
- Did you wear your pedometer? Did you add steps in your day? How?

[Problem solve with the participants to address any barriers.]

[Praise all efforts to self-monitor and to change eating and activity behaviors. Be positive and nonjudgmental. Encourage group discussion.]

Copyright 2017 University of Pittsburgh

Make Social Cues Work for You (page 1)

In an earlier session, we talked about **how to "take charge of what's around you."** We invited an imaginary film crew to walk through your house and where you work, and we looked for problem food or activity "cues," things that would prompt you to think about eating or to be inactive, like a TV or a bag of cookies on a kitchen counter. We planned some ways to get rid of problem cues and add positive cues (for example, watch less TV, keep high-calorie foods out of the house, and keep your walking shoes in sight).

In that session, we focused on the sight and smell of food or certain activities that make you think about food. Today we're going to talk about **social cues**, or **what other people say or do that affects your eating and activity.** Again, we will plan some ways to **reduce problem social cues** and some ways to **add positive ones.**

*[Review and discuss **Problem Social Cues** and include the following:]*

- **The sight of other people eating problem foods or being inactive.** For example, you go to a bar where you see other people eating potato chips and watching TV. Can you think of an example in your own life? Is it difficult for you when you see someone in your family or a friend eat certain foods? *[Ask participants to write brief examples on page 1.]*

- **Being offered (or pressured to eat) problem foods** or **being invited to do something that involves sitting for a long time** are also negative cues. For example, your spouse buys you candy for your birthday or a friend asks you to come over to watch football. What are some examples in your own life?

- **Being with peers who are rarely active.** Can you think of examples in your own life? Does this impact your activity level?

- **Being nagged** is a negative cue. For example, your spouse says, "You shouldn't be eating that bacon. It's too high in calories and fat." Some people may think that nagging is helpful, but often it backfires. Does anyone nag you about your eating or activity? How do you respond?

- **Hearing complaints** is a negative cue, too. For example, your daughter says, "I hate all these vegetables you are serving." Or your spouse says, "You're always outside walking. You don't have any time for your family anymore." Do you hear complaints from anyone about your eating or activity?

Now let's compare problem social cues with **positive or helpful social cues.** *[Review and discuss Helpful Social Cues and include the following:]*

- The **sight of other people eating healthy foods or being active.** For example, you go out to dinner with another participant who orders low-calorie foods or you go to an aerobics class. Can you think of any people who are good examples for you? In what way? *[Ask participants to write brief examples on page 1.]*

Copyright 2017 University of Pittsburgh

- **Being offered healthy foods** or **being invited to do something active.** For example, your mother offers you fruit salad for dessert or asks you to go for a walk. Does anyone do this for you?

- **Being praised.** For example, your spouse says, "The oatmeal was delicious this morning, honey." Who praises you for your efforts and accomplishments?

- **Hearing compliments.** For example, your daughter says, "I like these oven-roasted vegetables you made. Thanks, Mom." Or your spouse says, "You're really committed to walking every day. I'm proud of you." Does anyone compliment you?

When you respond to a social cue in the same way over and over again, you build a habit. The cue becomes paired with the way you respond, and your response becomes more and more automatic. In an earlier session, we used the example of eating popcorn whenever you go to the movies as a food cue that over time becomes a habit for many people. It works the same way with social cues.

Let's say that since childhood, your mother has offered you second helpings of food at the dinner table. You developed a habit of accepting her offer. Now when you return home as an adult and your mother offers you second helpings, it is hard for you to refuse.

It's important to understand that with social cues, the **other person has also learned a habit.** So, in the example we've just used, your mother has learned to offer you second helpings and expects that you will accept the offer. **This makes social cues even harder to change than other cues.**

To Change Problem Social Cues (page 2)

How can you change problem social cues? *[Review page 2 and include the following:]*

1. As with problem food cues, one of the best things you can do is to **stay away from the cue, if you can**. For example:

   Move to a different room if a family member eats problem foods in front of you.
   - Skip certain parties that are just too tempting for you.
   - Socialize with people by going bowling, dancing, or to the movies. Don't go out to eat as a way to socialize.
   - Change the subject when someone starts to criticize your eating, physical activity, or your weight.

2. **Change the cue, if you can.** This means trying to influence the other person's habit, if you can. For example, when someone nags, complains, eats problem foods in front of you, or pressures you to eat:

   - **Discuss the problem. Brainstorm options.** For example, "It's hard for me when you eat ice cream in front of me. It really tempting. Is there a way we could get

Copyright 2017 University of Pittsburgh

together and have fun, but not eat ice cream?" **Be willing to compromise** to find a solution that will work for everyone.

- **Tell people about GLB. Discuss your efforts to eat a healthier diet, be more active, and lose weight. Talk about why this is important to you.** Many people will be willing to help if they understand that you are trying to change your eating and activity habits to improve your health.

- **Ask others to praise you for your efforts and ignore your slips. This is KEY to your success.** Explain to your friends and family what would be most helpful to you. In turn, be sure to thank them when they notice your efforts and overlook your slips.

Note: One option is to role play this with the participants. Either ask one volunteer to role play with you in front of the group using an example that is meaningful to him or her, or break the group into pairs to role play and then ask volunteers to share their experience.

3. If you can't stay away from the problem social cue or change it, **practice responding in a healthier way**. Over time you will **build a new, healthier habit** and **the other person will learn a new habit, too,** because of your new response. For example:

   - **Say "No" to food offers.** If you are consistent and continue to say "No," others will eventually stop offering.

   - One of the most important things you can do is to **show others you know they mean well, and suggest something they can do to help you. Be specific.** Most people mean well when they nag, offer food or pressure someone to eat (for example, many people think that being a good hostess means insisting that guests have second helpings). If you recognize that they mean well and give them a specific, positive alternative, they can still feel helpful and you are more likely to reach your goals, too. For example, when a hostess offers you second helpings, say, "Thanks so much for offering. You know what I'd really enjoy is some coffee." **If you can, give them specific ideas of how to help ahead of time,** before you are confronted by a challenging situation.

Note: One option is to role play saying "No" to food offers,(e.g., "Are you sure you don't want a piece of cake?"), either with one volunteer in front of the group or by breaking the group into pairs to role play and then asking volunteers to share. Illustrate that the participants should be prepared to say "No" several times, and suggest alternatives to someone who continues to offer food.

Remember that **it takes time to break an old habit or build a new one.** Change doesn't happen overnight. And with social cues, there are at least two people involved in making

Copyright 2017 University of Pittsburgh

a change: yourself and someone else. **Don't expect other people to adjust instantly** to a new way of relating, any more than you expect yourself to change instantly.

To Add Helpful Social Cues (page 3)

[Review page 3 and include the following:]

Not all social cues are problems. You can use social cues to *help* you eat healthier and be more active. For example:

- **Spend time with people who are active and make healthy food choices.** For example, at parties stand next to people who spend most of their time talking and dancing instead of eating.
- **Put yourself in places where people are active**. For example, join an exercise club or sports league. Come to this program's activity sessions (if available).
- **Set up a regular "date" with others to be active**. You will be more likely to be active because you won't want to disappoint them by cancelling.
- **Ask your friends to call you to remind you to be active or to set up dates to be active.** Has anyone done this? Is it helpful?
- **Bring a low-calorie food to share.** For example, bring a fruit salad to a potluck dinner. Has anyone done this? How did it go?
- **Be the first to order when you eat out at a restaurant** and order healthy foods. This is much easier than waiting until after others order high-calorie foods and then trying to make a healthier choice. In addition, you will provide a positive social cue for other people.
- **Be social by doing something active.** For example, take a walk and talk. Go out dancing instead of going out to dinner. Start a family tradition of taking a walk after dinner instead of watching TV.
- What are some other social cues you might find helpful?

What Social Support Do You Need? (page 4)

[Review the information at the top of page 4. Ask participants to record and share their answers, if comfortable doing so. Encourage discussion.]

An important way to change negative social cues and add positive ones is to **ask people who want to support you for help.**

What people in your life want to support you?

Copyright 2017 University of Pittsburgh

What could they do to help you? Here are some ideas. Would any of these be helpful to you?

> Review the ideas on page 4. Ask participants to check a few that they think would be helpful and add other ideas at the bottom of the chart. Encourage participants to go over this page with a supportive friend or their family members.

Social Cues are Powerful at Social Events (page 5)

Social cues are especially powerful at social events such as parties, holidays, vacations, and when you have guests in your home or are a guest in someone else's home. These events:

- **Upset our routine.** For example, you usually walk after dinner, so how do you fit walking in on a day when you're going to a party after dinner?
- **Challenge us with unique food and social cues.** For example, you go on vacation to a place you've never been before. There are dozens of appealing restaurants and you're not familiar with any of them.
- **May involve habits that have developed over many years and so can be very powerful.** For example, for the past 30 years on Thanksgiving, your family has spent the entire afternoon watching football on TV and eating.

What are some social events that are difficult for you?

> Note: Get an idea of the kinds of social, cultural, or religious events the participants attend. If it is close to a holiday or vacation, you may want to focus on brainstorming options and making an action plan for these events.
> Additional handouts are in "Resources". Handouts include "Getting Ready for the Holidays," "Staying Active on Holidays," "Lifestyle Balance on Vacation," "Lifestyle Balance on Vacation: Problem Solving," and "Staying Active on Vacation". Review them as appropriate. They may also be used at any time during GLB.

To handle social events well, try to anticipate the problems that will occur. What exactly might be difficult for you? Then brainstorm your options ahead of time.

Here are some ideas *[review the examples on page 5]*:

- **Plan ahead.**
- **Stay away from problem cues when you can.**
- **Change problem cues.**
- **Respond to problem cues in a more healthy way.**
- **Add helpful social cues.**

Stay positive. Think of every social event as an opportunity to learn what works well for you and what doesn't. Remember, you are building healthy habits for a lifetime.

Copyright 2017 University of Pittsburgh

To Do (page 8)

Turn to page 8 and let's focus on what you can do between now and the next session.

Please continue to keep track of your weight, eating, and activity minutes. Come as close as you can to your calorie, fat gram, and activity goals.

New things to practice:

Describe a social cue that's a problem for you. *[Give participants time to record the problem on page 6.]* Make a positive action plan. *[If time permits, give the participants time to complete the worksheet.]*

Think of positive social cues you would like to add. *[Give participants time to record this on page 7.]* Make a positive action plan. *[If time permits, give the participants time to complete the worksheet.]*

Try your **two action plans** for making social cues work for you. And before the next session, answer the questions for both action plans (Did it work? If not, what went wrong?). You can share your experience/feedback next week. *[Note: If it is near a holiday, vacation, or particular social event, include an action plan for that event.]*

Add at least 250 steps per day to your activity this week. [*Have participants fill in the blank.]* What can you do to walk more steps per day?

[Note: This is the standard GLB step progression. Many participants will be able to comply. However, you should make exceptions for individuals with serious health problems that limit their mobility. For them, they should start where they are and gradually increase as much as they can.]

[Announce the day, time, and place for the next session.]

After the session:

- Weigh participants who did not do so prior to the group meeting.
- Complete data forms and documentation required in your setting.
- Follow your program's protocol for managing absences.
- Review the self-monitoring records from the previous week. Write brief comments. Be positive and nonjudgmental.
    - Praise all efforts to self-monitor and to change eating and activity behaviors.
    - Highlight especially any positive changes made that relate to the session topic of the week before the records were collected: making healthy food choices when eating out, coming close to their calorie, fat gram, activity, and step goals.
    - Refer to **Guidelines for Reviewing Food and Activity Records** available in the GLB Providers Portal.

Copyright 2017 University of Pittsburgh

Session 12:
Ways to Stay Motivated

Progress Review

Changes you have made so far:

To be more active:

Planned physical activity: ___________________________________

Spontaneous activity: ______________________________________

To change your eating pattern:

Fewer calories: ____

Healthier food choices: _____________________________________

Have you reached the 7% weight loss goal? ☐ Yes ☐ No

Are you on track with your personal weight loss goal? ☐ Yes ☐ No

Have you reached the goal of at least 150 minutes per week of physical activity? ☐ Yes ☐ No

Are you on track with your personal activity goal? ☐ Yes ☐ No

> If not, *take heart.*
>
> You have learned many skills that will help you move forward.

At the end of this session, you will set goals to carry with you into the next phase of the program.

Copyright 2017 University of Pittsburgh

Ways to Stay Motivated

1. **Stay aware of the benefits you've achieved and hope to** achieve.

What did you hope to achieve when you started Group Lifestyle Balance™? Have you reached these goals?

____

____

What would you like to achieve in the next few months?

____

____

2. Recognize your successes.

What changes in your eating and physical activity do you feel proudest of?

____

____

____

____

Copyright 2017 University of Pittsburgh

3. Keep visible signs of your progress.

- Keep weight and physical activity graphs (minutes or steps) where you can see them. Your graphs will help you stay focused on your goals.
- Measure yourself (waist, belt size) once a month and chart your progress.
- Other ways: ____

4. Do you need to vary your routine?

Is it time for you to vary your physical activity? ☐ Yes ☐ No

____

Can you think of some ways to vary it?

____

Is it time to add more variety to your meals and snacks?

____

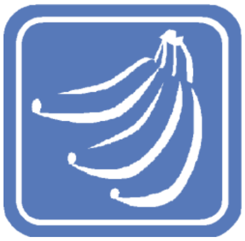

____

Some people do best with structure, others with variety. There is no one right way**.... look** for the things that keep *you* motivated.

Copyright 2017 University of Pittsburgh

5. Create some friendly competition.

Do you like to compete with others or yourself?

How? ____

6. Use others to help you stay motivated.

Who gives you support and encouragement?

____

Are there new connections you can make?

____

7. Manage stress**.... see** pages 5-6.

8. **Set new goals...see page** 7.

Copyright 2017 University of Pittsburgh

Manage Stress

Stress is tension or pressure. It is not uncommon for people to respond to stress by overeating, drinking too much alcohol, or being inactive.

- What kinds of things make you feel stressed? ____
- What is it like for you when you get stressed? ____
- How have you learned to respond to stress in healthy or unhealthy ways? ___________________________________

Use *all* the skills you have learned in this program to prevent or manage stress.

- Practi**ce saying “No.** Try to say, “Yes” only when it is important to *you*.
- Share some of your work with others.
- Set goals you can reach.
- Take charge of your time. Make schedules with the real world in mind. Get organized.
- Use problem solving. Describe the problem in detail. Brainstorm your options. Pick one option to try. Make an action plan. Try it. See how it goes.
- Plan ahead. Think about the kind of situations that are stressful for you. Plan for how to handle them or work around them.
- Keep things in perspective. Remember your purpose. Think of all of the good in your life. Remember why you joined GLB.
- Reach out to people.
- Be physically active.

Copyright 2017 University of Pittsburgh

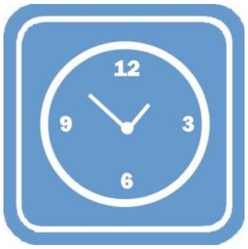

When You Can’t Avoid Stress

Catch yourself feeling stressed as early as you can.

Take a 10-**minute “time out.”**

- Move those muscles.
- Pamper yourself. Just take 10 minutes for YOURSELF.
- Breathe. Try this: Take a full, deep breath. Count to five. Then let go of your breath slowly. Let your face, arms, legs, and body muscles relax.

Group Lifestyle Balance™ may cause stress. For example:

Sources of Stress	To Manage Stress	Examples
It takes extra time to shop for and prepare food.	• Share your work.<br>• Take charge of your time.	• Ask your family to help.<br>• Make double recipes. Freeze part for later.
Your food budget is tight; healthy options are limited.	• Reach out to people.<br>• Problem solve.<br>• Plan ahead.	• Explore community resources.<br>• Stock up on frozen/canned fruits and vegetables from discount markets.
You feel deprived when you can’t eat favorite foods.	• Set goals you can reach.<br>• Keep things in perspective.	• Enjoy favorite foods in small amounts now and then.<br>• Remind yourself why you want to have a healthy lifestyle.
Your family doesn’t like low-calorie foods.	• Reach out to people.<br>• Problem solve.	• Ask your family to support you in trying new foods.<br>• Brainstorm options together.
You feel uneasy at social events where there are high-calorie foods.	• Practice saying “No.”<br>• Reach out to people.<br>• Plan ahead.	• Turn down invitations that aren’t important to you.<br>• Call the host/hostess. Ask if you can bring a low-calorie dish.<br>• Before you go to the party, plan what foods to choose.
It is hard to fit activity into your busy life.	• Plan ahead.<br>• Problem solve.	• Make a date to be active.<br>• Be active while doing other things. Take a walking meeting. Hike with family.
Remembering to make the active choice is hard.	• Try to be more aware of adding in more steps each day.	• Park further away from the entrance.<br>• Take stairs instead of elevator.<br>• Get off the bus a few stops earlier.

Copyright 2017 University of Pittsburgh

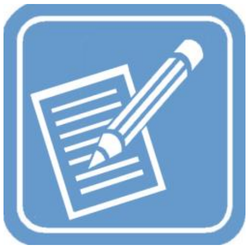

Set New Goals for Yourself

Find ways to reward yourself when you meet each goal.

Goals: Specific, short-term, just enough of a challenge

Rewards: Something you will do or buy if and only if you reach your goal

What are some non-food ways you can reward yourself for reaching a goal?

____

____

____

____

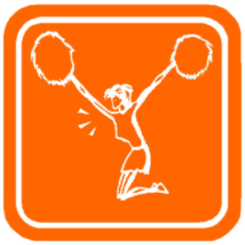

Copyright 2017 University of Pittsburgh

What's Your Pleasure?

It takes time and effort to build new eating and physical activity habits. One way to stay motivated is to be sure there's enough pleasure in your life.

Name three things you enjoy doing but rarely do.

Choose things that are not related to food and that are easy to do. See the next page for ideas.

1. ____

2. ____

3. ____

Schedule the above pleasures into your life.

Make appointments with yourself on a calendar. When you can, schedule your pleasures for:

- **When the "old you" might have rewarded yourself by** overeating or being inactive. For example, do you have a habit of eating ice cream after working hard in the garden? Find a way to relax in the garden (e.g., read a book, talk on the phone with a friend).
- Right after you have taken a challenging step toward change. For example, have you been putting off enrolling in a water aerobics class? Plan to take a long, hot bath on the night after the first class.
- When you feel discouraged. Remember, it takes time to change. You deserve to be good to yourself for all of the efforts you make.
- Keep your appointments with yourself, no matter how odd it feels. Treat them like you would treat appointments with someone else you care about.

Copyright 2017 University of Pittsburgh

50 Ways to Be Good to Yourself

1. Soak in the bathtub.
2. Plan your career.
3. Collect shells.
4. Recycle old items.
5. Go on a date.
6. Buy flowers.
7. Go to a movie in the middle of the week.
8. Walk with a friend.
9. Listen to music.
10. Recall past parties.
11. Buy household gadgets.
12. Read a humor book.
13. Think about your past trips. Or plan future ones.
14. Listen to others.
15. Read magazines or newspapers.
16. Do woodworking.
17. Build a model.
18. Spend an evening with good friends.
19. Plan a day's activities.
20. Meet new people.
21. Remember beautiful scenery.
22. Save money.
23. Go home from work.
24. Practice yoga or tai chi.
25. Think about retirement.
26. Repair things.
27. Work on your car or bicycle.
28. Remember the words and deeds of loving people.
29. Wear sexy clothing.
30. Have a quiet evening.
31. Collect coins.
32. Take care of your plants.
33. Buy or sell stock.
34. Go swimming.
35. Doodle.
36. Collect old things.
37. Go to a party.
38. Think about buying things.
39. Play golf.
40. Play soccer.
41. Fly a kite.
42. Have a discussion with friends.
43. Have a family get-together.
44. Take a day off with nothing to do.
45. Arrange flowers.
46. Have sex.
47. Ride a motorcycle.
48. Go to the beach.
49. Sing around the house.
50. Go skating.

Copyright 2017 University of Pittsburgh

MORE Ways to Be Good to Yourself

50. Paint.
51. Do needle point, crewel, knitting, sewing, etc.
52. Take a nap.
53. Entertain.
54. Go to a club meeting.
55. Go hunting, skiing, or fishing.
56. Sing with a group.
57. Flirt.
58. Play a musical instrument.
59. Make a gift for someone.
60. Collect postcards.
61. Buy a record, tape, or CD.
62. Plan a party.
63. Buy clothes.
64. Sightsee or window-shop.
65. Garden.
66. Go to a beauty parlor.
67. Play cards, chess, etc.
68. Buy a book.
69. Watch children play.
70. Write a letter or card.
71. Write in a diary.
72. Go to a play or concert.
73. Daydream.
74. Take a class.
75. Go for a drive.
76. Listen to music.
77. Refinish furniture.
78. Take a sauna or steam.
79. Make a list of things to do.
80. Ride a bike or a horse.
81. Take a walk in the woods.
82. Buy a gift for someone.
83. Visit a national park.
84. Take photographs.
85. Play with animals.
86. Read fiction or nonfiction.
87. Watch an old movie.
88. Go dancing.
89. Meditate, pray, or go to church.
90. Go bowling.
97. Go the mountains.
98. Think about happy memories.
99. Look at photographs.
100. Play cards, checkers, etc.
101. Do a jigsaw puzzle
102. Solve riddles.
103. Discuss politics.
104. Play softball or volleyball.
105. Do crossword puzzles.
106. Shoot pool.
107. Dress up and look nice.
108. Buy something for yourself.
109. Talk on the phone.
110. Kiss.
111. Go to a museum.
112. Light candles.
113. Get a massage.
114. Say, “I love you.”
115. Start an aquarium.

Adapted from the *Skills Training Manual for Treating Borderline Personality Disorder* by Marsha Linehan. © 1993 The Guilford Press.

What is Your Purpose Now?

You have been working hard to change your lifestyle.

Why is reaching and staying at a healthy weight and being physically active important to you?

____

____

Take a moment to revisit your healthy lifestyle goals.

My Weight Goal

What is your weight goal now?

- ☐ I am working on reaching the 7% weight loss goal.
- ☐ I have reached the 7% weight loss goal.
- ☐ I am working on a personal weight loss goal.

In the next month, I will focus on (check one):

- ☐ Continued weight loss.
- ☐ Weight maintenance.

Copyright 2017 University of Pittsburgh

My Physical Activity Goals

What is your planned physical activity goal now?

- ☐ I am working on reaching the 150 minute per week goal.
- ☐ I have reached the 150 minute per week goal.
- ☐ I am working on a personal physical activity goal of _______ minutes per week.

What are your other activity goals?

- ☐ I am working on adding spontaneous activity as often as I can each day.
- ☐ I am working on adding steps each day to reach at least 7,000 steps per day, or about 50,000 per week.

In the next month, I will focus on (check one):

- ☐ A weekly activity goal of _________ minutes.
- ☐ A weekly step goal of __________.
- ☐ I will add spontaneous physical activity.

My Self-Monitoring Goals

Research shows that people who keep track are better at losing weight, staying active, and maintaining healthy lifestyle behaviors.

Look at page 13. Based on how I am doing right now:

I will commit to self-monitor at this level:

- ☐ Gold
- ☐ Silver
- ☐ Bronze

Copyright 2017 University of Pittsburgh

How I Will Keep Track

Choose and commit to one of the following three levels of self-monitoring based on how you are doing now. You should adjust the level (frequency and detail) based on your progress toward your weight and/or activity goals.

For all levels, continue to record your weight. Also record activity minutes or track your activity in any way that keeps you motivated.

Gold

- Record everything every day.
- Choose this if:
  - You aren't at your weight and/or activity goals yet.
  - You start to regain weight or stop exercising.
  - You know this level of detail helps you stay motivated and in control.

Silver

- Cut back somewhat on recording, but choose wisely. For example, you might decide to record calories/fat on fewer days per week, for the times of day when you need more control, or just write what you eat and drink.
- Choose this if:
  - You *are* at or below your weight and/or activity goals.
  - You are feeling confident about your eating and activity behaviors. You want to do some recording because it helps you stay aware.

Bronze

- Use the Group Lifestyle Balance™ Monthly Calendar (page 16) or other simple tracking method. At a minimum, record weight and physical activity at least once a week. Daily may be best.
- Choose this if:
  - You are feeling confident and in control.

Copyright 2017 University of Pittsburgh

To Do:

Check the boxes when you complete each item:

- ☐ Keep track of your weight. Weigh yourself at home at least once a week. Record it.
- ☐ Record everything you eat and drink every day. Come as close as you can to your calorie and fat gram goals.
- ☐ Record your physical activity. Come as close as you can to the GLB goal of at least 150 minutes per week. Aim for at least 7,000 steps per day, or about 50,000 per week.

New things to practice:

- ☐ Try one way to stay motivated that will be helpful to you right now. Review the suggestions on pages 2-4.

  ____

- ☐ Take steps to connect with others who support your lifestyle goals (page 4).

  What is a good first step?

  ____

- ☐ Try to do three things you enjoy doing but rarely do. Copy below the list you made on page 8.

The three things I enjoy doing but rarely do are:

1. ____
2. ____
3. ____

Schedule these pleasures into your life.

- ☐ Remember your purpose. Commit to following your healthy lifestyle goals for weight, activity, and self-monitoring (pages 11 & 12).

Copyright 2017 University of Pittsburgh

Session 12: Resources	Page

Group Lifestyle Balance™ Monthly Calendar 16

Copyright 2017 University of Pittsburgh

Group Lifestyle Balance™ **Monthly** Calendar

Name: ___________________________

Goals: Activity ____________ minutes per week Steps ____________ per week
Weight Range ______ - ______ pounds.

Sunday	Monday	Tuesday	Wednesday	Thursday	Friday	Saturday	Totals
☐ Activity ____ Steps ____ Weight ____ ☐Recorded diet	☐ Activity ____ Steps ____ Weight ____ ☐Recorded diet	☐ Activity ____ Steps ____ Weight ____ ☐Recorded diet	☐ Activity ____ Steps ____ Weight ____ ☐Recorded diet	☐ Activity ____ Steps ____ Weight ____ ☐Recorded diet	☐ Activity ____ Steps ____ Weight ____ ☐Recorded diet	☐ Activity ____ Steps ____ Weight ____ ☐Recorded diet	Weekly Activity ______ minutes ______ steps
☐ Activity ____ Steps ____ Weight ____ ☐Recorded diet	☐ Activity ____ Steps ____ Weight ____ ☐Recorded diet	☐ Activity ____ Steps ____ Weight ____ ☐Recorded diet	☐ Activity ____ Steps ____ Weight ____ ☐Recorded diet	☐ Activity ____ Steps ____ Weight ____ ☐Recorded diet	☐ Activity ____ Steps ____ Weight ____ ☐Recorded diet	☐ Activity ____ Steps ____ Weight ____ ☐Recorded diet	Weekly Activity ______ minutes ______ steps
☐ Activity ____ Steps ____ Weight ____ ☐Recorded diet	☐ Activity ____ Steps ____ Weight ____ ☐Recorded diet	☐ Activity ____ Steps ____ Weight ____ ☐Recorded diet	☐ Activity ____ Steps ____ Weight ____ ☐Recorded diet	☐ Activity ____ Steps ____ Weight ____ ☐Recorded diet	☐ Activity ____ Steps ____ Weight ____ ☐Recorded diet	☐ Activity ____ Steps ____ Weight ____ ☐Recorded diet	Weekly Activity ______ minutes ______ steps
☐ Activity ____ Steps ____ Weight ____ ☐Recorded diet	☐ Activity ____ Steps ____ Weight ____ ☐Recorded diet	☐ Activity ____ Steps ____ Weight ____ ☐Recorded diet	☐ Activity ____ Steps ____ Weight ____ ☐Recorded diet	☐ Activity ____ Steps ____ Weight ____ ☐Recorded diet	☐ Activity ____ Steps ____ Weight ____ ☐Recorded diet	☐ Activity ____ Steps ____ Weight ____ ☐Recorded diet	Weekly Activity ______ minutes ______ steps
☐ Activity ____ Steps ____ Weight ____ ☐Recorded diet	☐ Activity ____ Steps ____ Weight ____ ☐Recorded diet	☐ Activity ____ Steps ____ Weight ____ ☐Recorded diet	☐ Activity ____ Steps ____ Weight ____ ☐Recorded diet	☐ Activity ____ Steps ____ Weight ____ ☐Recorded diet	☐ Activity ____ Steps ____ Weight ____ ☐Recorded diet	☐ Activity ____ Steps ____ Weight ____ ☐Recorded diet	Weekly Activity ______ minutes ______ steps

Copyright 2017 University of Pittsburgh

Session 12 Leader Guide:
Ways to Stay Motivated

Objectives
In this session, the participants will:

- Review their progress since Session 9, and if not at goal, develop a plan to improve.
- Discuss the importance of motivation and ways to stay motivated.
- Discuss how to prevent stress and cope with unavoidable stress.
- Learn how to set new goals and determine ways to reward themselves for reaching goals.
- Discuss how Group Lifestyle Balance can be a source of stress and how to manage that stress.
- Make a plan for continuing to reach and maintain lifestyle goals as they transition to the next phase of Group Lifestyle Balance.
- Understand that although the frequency of GLB meetings is reduced, they will have continued contact and support.

To Do Before the Session:

- If providing Food and Activity books; give two per participant.
- Review Food and Activity records from last session; add comments.
- Prepare Session 12 handout for participant notebook.

Available in the GLB Providers Portal:
https://www.diabetesprevention.pitt.edu/my/login.aspx

- **Guidelines for Reviewing Food and Activity Records**

Group Sharing Time (allow at least 5 minutes)

The last session was "Make Social Cues Work for You."

- Did you follow your actions plans for changing a problem social cue and adding a positive social cue? What went well? What could you do differently?
- Did you talk with anyone about how to give you the support you need? How did it go?

Let's take a few minutes to talk about your experiences this past week. *[Choose only a few of the following prompts to guide discussion.]*

- Overall, how did it go last week? What went well? What problems did you have? What could you do differently?

Copyright 2017 University of Pittsburgh

- Were you able to stay close to your calorie and fat gram goals? Follow your plan for physical activity, including lifestyle activity? *[Remind participants to graph their activity if they have not yet done so.]*
- Did you wear your pedometer? Did you add steps in your day? How?

[Problem solve with the participants to address any barriers.]

[Praise all efforts to self-monitor and to change eating and activity behaviors. Be positive and nonjudgmental. Encourage group discussion.]

Progress Review (page 1)

In GLB we will sometimes pause and give you a chance to think about the changes you have made so far in your eating and activity behaviors. This allows you to celebrate the positive lifestyle changes you have made. It also allows you to think about your focus for the upcoming month.

First, let's take some time to review your progress since Session 9, which is the last time we formally looked at how you were doing.

First, **what changes have you made to be more active**? We've talked about increasing both structured/planned activity, the kind you've been recording, and spontaneous/ unstructured activity, like taking the stairs instead of an elevator, that add more total movement to your day.

[Give the participants some time to briefly record on page 1 some of the changes they've made. Ask volunteers to share, if they feel comfortable doing so. Praise and encourage the maintenance of these changes.]

Now let's talk about the **changes you have made in your eating pattern.** Think about changes you have made to eat fewer calories and to make healthier food choices. *[Again, have participants briefly record some of the changes they've made. Ask volunteers to share, if they feel comfortable doing so. Praise and encourage the maintenance of these changes.]*

Now let's look at whether the scale is reflecting your healthy eating and activity behavior changes.

Look at your **Weekly Record**. Have you reached the GLB 7% weight loss goal? Check yes or no.

Are you on track with your personal weight loss goal? We talked about this in Session 9. Some people have the goal of losing 7% while others would like to continue losing until they reach a personal weight loss goal. We also talking about having a 5 pound range as your weight goal.

Copyright 2017 University of Pittsburgh

Now let's look at your physical activity. [*Participants should refer to the* ***How am I Doing?*** *graph.]*

- Have you reached the goal of 150 minutes of physical activity per week?
- Some people have the own personal activity goal. Are you on track with this goal? *[Ask for volunteers to share, if they feel comfortable doing so.]*

[Emphasize that it's important for the participants to focus on their accomplishments so far, no matter how small. Express your confidence in the participants' potential for future success.]

Lifestyle change is a process. Look at you progress toward reaching your goals. Do not expect perfection.

[Be encouraging. Praise all small steps the participants have made so far.]

Ways to Stay Motivated (pages 2-4)

Today we're going to talk about ways to stay motivated for the long term, to make healthy eating and being active last for a lifetime.

In programs like GLB, **motivation is crucial to maintaining healthy eating and physical activity for the long term.** But how to stay motivated is **one of the biggest problems people face. It is normal for your healthy lifestyle motivation to ebb and flow.** *[Note: Feel free to use your own analogies or metaphors for describing motivation. For example, "How you choose to divide your energy pie" or "Motivation is like a ball of energy that may get bigger or smaller depending on how well you are taking care of yourself."]*

One reason it's difficult to stay motivated is the fact that many people do well. This sounds ironic--your progress itself makes it hard to *maintain* that progress. But think back to when you first joined GLB. *[Tailor the following examples to the participants' experiences thus far in the program.]* You may have felt tired when you walked up stairs and that motivated you to become more active. Now that you're more active, you can climb stairs without difficulty. So that source of motivation (feeling tired when you climbed stairs) has decreased.

It's the same for weight. When you first came into GLB, your clothes may have been tight and that motivated you to lose weight. If your clothes are looser on you now, you may no longer have tight-fitting clothes as a source of motivation.

However, it *is* possible to stay motivated for the long term. There are several strategies that others have found helpful for staying positive, motivated, and willing to work on their healthy lifestyle behaviors. *[Review pages 2-4 and include the following:]*

1. Stay aware of the benefits you've achieved and hope to achieve.

Copyright 2017 University of Pittsburgh

Again, think back to when you first joined GLB. What did you hope to achieve?

[Ask participants to record their answers. Refer the participants back to Session 1, page 4, "Remember Your Purpose", and ask them to review their answers. Ask volunteers to share.]

Have you reached these goals?

Have you received any benefits that you didn't expect?

What would you like to achieve during the next few months? Let's make a list and then you can review these when you need motivation. *[Give participants time to record their answers. Ask volunteers to share.]*

2. Recognize your successes.

- What changes in your eating and activity habits do you feel proudest of?
- What has been easier than you thought it would be?
- What has been harder than you thought it would be?

When you are feeling low on motivation, think about all of these positive changes and give yourself credit for them. Try not to lose the momentum you have gained so far.

3. **Keep visible signs of your progress.** Here are some options to consider. Think about which ones might work best for you.

   - **Keep weight and physical activity graphs where you can see them.** Not only will it keep you aware of your progress, but others may take note and congratulate you for your movement in the right direction.
   - **Mark your activity milestones on a map toward a particular goal.** For example, create a simple map of the number of miles it would take to walk to a favorite vacation spot or a favorite city. Mark milestones along the way. For example, the halfway point, cities you pass, etc.
   - **Measure yourself at monthly intervals.** Keep track of your progress in terms of specific measurements (for example, waist circumference or the number of holes on your belt).
   - What are some other concrete ways that would help to keep you motivated?

4. Do you need to vary your routine?

   We've talked before about how to "jump start" your activity plan.
   - Have you added some variety to keep from being bored with staying active?
   - Have you noticed any difference in how you feel about being active?

The same thing may be true with eating. If you have grown tired of using the same low calorie salad dressing every night, experiment with some new products. Consider trying new recipes and restaurants. If you are the kind of person who has a desire to be more creative there are many magazines and websites that provide an outlet to explore new, healthy meals.

On the other hand, if you are the kind of person who is comfortable with "routine eating" and simply do not require that much variation in your breakfast, lunch and dinner, that is perfectly fine too. Some people report doing better, over time, with structured meals and minimal variety, others do not. What is important is to get to know who you are and plan accordingly.

Are there meals, snacks, or particular foods that you are feeling the need to add variety?

[Ask participants to record their ideas on page 3 and to share their responses. Choose a few examples from the box below to prompt discussion.]

- Make one night a week an "ethnic night," "soup night," or "vegetarian night."
- If you eat out often, plan more meals at home.
- If you eat at home often, plan more meals out. (Have you stopped eating out because you're trying to lose weight? Has this left you feeling restricted and deprived? Have you stopped inviting friends over to eat or accepting invitations to eat at their homes? Don't deny yourself the pleasure of social eating. Instead, make a plan for how to handle these times, then try your plan, and see how it works. You may make a few mistakes at first, but it's important to know that you **can** eat out and still eat healthy, as we discussed in Session 10.)
- Share food preparation and dining with others as a way to relax. Invite people over to prepare dinner together. Cook with your children and spouse.
- Plan potluck dinners around a certain theme and share the best recipes as a group.
- You may want to subscribe to a magazine that includes healthy recipes.
- Or take a class to learn how to cook, at least the basics.

[If participants express interest in learning more about a specific topic such as ethnic cooking or vegetarian eating, address it briefly here and if possible, direct them to where to find books and/or courses on these topics. They may also ask about healthy meal kit home delivery services. Encourage discussion.]

[Emphasize the "bottom line" message at the bottom of the page.]

5. Create some friendly competition (page 4)

Ask a friend or relative to enter into a friendly competition with you. This should be the **kind of competition in which you both win.** Example: If you and your friend both meet your activity goal every week for a month, at the end of the month how will you reward yourselves?

Copyright 2017 University of Pittsburgh

Or compete with yourself. See how many days in a row you can be active for at least 10-15 minutes. Try to beat yourself. For example, if last month you were active for seven days in a row, see if you can do more days this month.

6. **Use others to help you stay motivated.**

   If you notice that your motivation is dropping, talk with someone who is supportive. Has anyone done this? How did it go?

   Everyone has trouble staying motivated sometimes, and we can encourage each other through the tough times. How can you use the group to stay motivated?

We're going to talk in more detail about the last two ways to stay motivated: manage stress and set new goals.

Manage Stress (page 5)

[Review page 5 and include the following:]

Stress is a natural part of living. Most people can manage to live a healthy lifestyle even with some amount of stress.

Any change, good or bad, big or small, can cause stress. Big changes or events in our life can cause stress such as getting married, a serious illness, or changing jobs. Small events like losing your keys, having a birthday, having a flat tire, or needing to get your errands done before picking up your children can also cause stress.

Why are we talking about stress in GLB? Because **many people react to stress by making unhealthy changes in their eating and activity habits.** Some people eat and drink too much as a way to deal with stress. Others may stop eating. Some people become very inactive and withdrawn.

Please take a few minutes and jot down your answers to the three questions at the top of the page. *[Give participants time to record their answers. Ask volunteers to share.]*

Discuss one or two situations that are common to the group (such as being under a deadline at work, being faced with unexpected responsibilities such as a sick child or car repair).
Ask how the participants feel or react in such situations.
Based on the responses, point out any physical and emotional symptoms (headache or muscle tension) and behavioral changes that might affect eating and activity.

- Do you get physical symptoms like a headache, stomach ache, or muscle tension?
- Do you change your behaviors when you feel stressed?
- Do you eat more when you are stressed?
- Do you change the kinds of food you eat?
- Do you change how active you are or the kind of physical activities you do?

An ounce of prevention is worth a pound of cure, and this is certainly true when it comes

Copyright 2017 University of Pittsburgh

to stress. The best approach is to **prevent stressful situations whenever you can**. Here are some ideas to help prevent or manage stress:

- **Practice saying, "No."**
  Practice saying "No" when someone else asks you to do something you don't want to do. Say "Yes" only when it is important to you.

  Saying "No" can be hard. It causes some tension or stress. But that stress is usually short-lived. If you say "Yes," you may have hours, weeks, or months stress as you do whatever you agreed to do.

- **Share some of your work with others.**
  This is important to do both at home and at work. Delegate what you can to someone else. For example, your spouse and children might be able to help clean the house, cut the lawn, shop for food, prepare meals, and do laundry. A co-worker might be able to help you with an overwhelming project at work.

  Sharing work doesn't mean you're being irresponsible. Giving responsibility to others, even if they aren't as experienced as you, gives them a chance to learn, participate, and gain experience. One warning: Don't expect them to be perfect. Criticizing the efforts of others who are trying to help can be another source of stress. Instead, support them for their efforts and be patient as they gain skills.

- **Set goals you can reach.**
  Sometimes we create our own stress by trying to be perfect. If you set reasonable goals, you are more likely to succeed. When you succeed, you are less likely to feel stressed. Remember, we talked about this when we discussed negative thoughts--if you try to be perfect, you probably won't succeed.

  Periodically, take a good look at the demands you are placing on yourself. Ask yourself, "Am I expecting myself to do more than anyone could possibly do?"

- **Take charge of your time.**

  Make schedules with the real world in mind. Don't try to accomplish in 30 minutes what realistically will take an hour. Take a good look at your to-do list, eliminate what isn't essential, and give yourself a realistic amount of time to accomplish the rest.

  Get organized. Chaos is very stressful. It's also inefficient. Devote some time every day to getting organized, and you will save time and stress in the long run.

- **Use the steps for solving problems.**
  If changing your eating and activity habits is causing stress, take action. Use the steps to solving a problem that we discussed in an earlier session. Discuss it with your family or friends if they are involved.

  Continue the problem-solving process until you find a solution. Sitting on problems can cause even more stress. Solve them instead and move on.

Copyright 2017 University of Pittsburgh

- **Plan ahead.**
  Think about what kind of situations are stressful for you. These are times when you are at high risk, so plan ahead for how to handle them or work around them.

  For example, are holidays especially stressful for you? If so, plan some ways to make your life easier during the holidays. Examples: Buy frozen meals to have on hand for busy days. Decide what parts of decorating the house are not essential to you and spend that time relaxing instead.

- **Keep things in perspective. Remember your purpose.** Maintain a positive attitude.

- **Reach out to people.**
  At our last group meeting we talked about social support and that research shows that people are better able to reach their eating and activity goals if they have some type of social support in place.

  Think about who you can turn to for support. **Ask supportive people to help** when you are overwhelmed or need someone to encourage you.

- **Be physically active.**
  Many people find that being active helps them cope with stress and feel more relaxed and able to manage stressful situations more smoothly. Have you noticed that being physically active helps you manage stress? [*Ask participants to share their experiences. Encourage group discussion.]*

Do you see how stress management builds on all the behavior change ideas you have been learning so far?

When You Can't Avoid Stress (page 6)

What about the times when you can't avoid stress? *[Review page 6 and include the following:]*

First, catch yourself feeling stressed as early as you can.
We talked before about action or behavior chains and that it's important to try to break them as early as possible. The same is true of stress. If you learn to recognize the signs of stress and catch yourself early in the process, you may have a chance to avoid some of the harmful consequences such as overeating or being inactive.

Do you have any signs when you are getting stressed? When you first notice the signs you are getting stressed…

Take a 10-minute "time out."

Copyright 2017 University of Pittsburgh

Develop a new habit of responding to stress with a "time out"--stop what you are doing and take a few minutes for **yourself.** Do whatever you find helpful that doesn't involve food. Examples:

- **Move those muscles.** Research has shown that being active relieves tension, reduces anxiety, and counters depression. So, when you notice yourself feeling stressed, make yourself go out for a 10 or 15-minute brisk walk. Or get on your exercise bike and pump for 10 minutes. The distraction and breathing can do a lot to make you feel better.

- **Pamper yourself.** Take a bath. Manicure your nails. Massage your feet. Read a magazine or watch sports. **Just take out 10 minutes for YOURSELF.**

- **Breathe.** Our breathing may become when we are under stress, which creates more tension in the body and mind. So, when you catch yourself feeling stressed, try this:
  Take a full, deep breath. Count to five. Then let go of your breath slowly. Let the muscles in your face, arms, legs, and body go completely loose.

We understand that GLB **and the lifestyle changes we recommend may cause stress.** Changing your behaviors and helping your family to make related changes can create pressure and tension.

Here are some possible ways GLB may cause stress and some examples of how to manage that stress.

[Note that some of the possible sources of stress may not apply to every participant. For example, a participant's family may enjoy low-calorie foods. Do not allow the discussion to become negative. The goal is to help participants feel able to cope in the face of stress.]

Set New Goals for Yourself (page 7)

The final suggestion for ways to manage stress is to **Set new goals for yourself, and find ways to reward yourself when you meet each goal.**

The **goal** should be **specific and short-term** ("I will not eat any potato chips this week"). It should also be something that's not too easy or too hard (something that will present **"just enough" of a challenge** for you that you will be able to do it and will also feel that you've accomplished something).

The **reward** should be **something that you will do or buy if and only if you reach your goal**. The reward doesn't need to be fancy or cost a lot of money. It can be something that you normally enjoy doing (like reading the paper or taking a hot bath) with the difference being that you will do it *only if* you reach your goal. For example, "After I finish this walk, I'll call my friend and chat." Then, if you need a boost to keep you going during your walk, you can think about what you'll talk about on the phone with your friend.

Copyright 2017 University of Pittsburgh

What are some non-food ways you can reward yourself for reaching a goal?
[Ask participants to record their ideas, such as: Buy myself fresh flowers, treat myself to a manicure or massage, get tickets to a sporting event, set money aside for something special you want to buy or do. Ask volunteers to share.]

What's Your Pleasure? (pages 8-10)

When you feel discouraged… Remember, it takes time to change. You deserve to be good to yourself for all of the efforts you make.

What are three things you enjoy doing but rarely do? Choose things that aren't related to food and that are within the realm of possibility. See pages 9 and 10 for ideas.

Try and schedule the pleasures you've chosen into your life. Make appointments with yourself. Follow through with your plan...no matter how odd it feels.

Treat your appointments with yourself as seriously as you would those with someone else you care about.

What is Your Purpose Now? (pages 11-13)

You have been making changes in your eating and activity behaviors for about three months. You deserve credit for the changes you have made, no matter how small. It's a challenge to change eating and activity habits, and every step along the way is a reason to celebrate.

First, you will need to keep in mind **why reaching and staying at a healthy weight and being active are important to you.** *[Ask participants to write their answer and to share, if comfortable doing do.]*

Is your purpose the same as when you started GLB? Has it grown or changed in any way? *[Ask participants to write their answer and to share, if comfortable doing do.]*

Our sessions will be less frequent but ongoing. It is important to consider your longer term goals for activity, weight, and self-monitoring. This is a good time to revisit and recommit to your lifestyle goals.

[Review pages 11-12 and include the following:]

My Weight Goal
[Allow time for participants to select their weight goal and if their focus will be on weight loss or weight loss maintenance.]

[Encourage participants to continue to weigh themselves at least once a week.]

Copyright 2017 University of Pittsburgh

My Physical Activity Goals (page 12)

[Allow time for participants to select their physical activity goal and if their focus for the next month. Encourage participants to continue to strive to reach and maintain at least 150 minutes per week of moderate physical activity. Also encourage them to wear their pedometer daily and to find ways to add more steps.]

My Self-Monitoring Goals

Research demonstrates that it's important to **keep track of your weight, eating and activity.**

It's common to "drift" away from new habits. You may gradually make small changes in your eating and activity over a long period of time, and not even be aware that you are slowly going back to your old habits. The best way to prevent this and stay in control is to continue to self-monitor. Keeping track will help you catch changes before they sneak up on you.

[Review page 13, ***How I Will Keep Track****. Participants should choose and commit to the Gold, Silver, or Bronze level of self-monitoring.]*

Some of you may enjoy tracking your weight, activity minutes, and steps in a way that lets you see the month as a whole. The **Group Lifestyle Balance™ Monthly Calendar,** on page 16, is an option for self-monitoring. *[Announce that you will provide additional copies of this calendar at each session.]*

To Do (page 14)

Turn to page 14 and let's focus on what you can do between now and the next session.

Please continue to keep track of your weight, eating, activity minutes, and steps. Come as close as you can to your calorie, fat gram, activity, and step goals.

New things to practice:

- Try one way to stay motivated that you think would be most helpful to you right now (see pages 2-4). Choose something that is very likely to work and that you can do.
- Take steps to connect with others who support your lifestyle goals. *[Ask participants to share their "first step", if comfortable doing so.]*
  Try three things you enjoy but rarely do. Schedule these pleasures and do them. You can share your experience/feedback next week.
- Remember your purpose. Commit to following your lifestyle goals for weight, activity, and self-monitoring.

[Announce the day, time, and place for the next session. Ask participants to wear comfortable clothes because we will be doing some exercises.]

Copyright 2017 University of Pittsburgh

[Review the schedule of classes for the remainder of the year with the participants. Emphasize the importance of continuing to attend the GLB sessions.]

You have gained important self-management strategies that will serve you well. Your motivation may ebb and flow, but the learning tools will help you each time you pick them up. As we continue in GLB, we will be revisiting these basic strategies and learning new ones to help keep everyone on track over the long run. I look forward to seeing you soon!

After the session:

- Weigh participants who did not do so prior to the group meeting.
- Complete data forms and documentation required in your setting.
- Follow your program's protocol for managing absences.
- Review the self-monitoring records from the previous week. Write brief comments. Be positive and nonjudgmental.
    - Praise all efforts to self-monitor and to change eating and activity behaviors.
    - Highlight especially any positive changes made that relate to the session topic of the week before the records were collected: making social cues work for them, adding steps, coming close to their calorie, fat gram, activity, and step goals.
    - Refer to **Guidelines for Reviewing Food and Activity Records** available in the GLB Providers Portal.

Copyright 2017 University of Pittsburgh

Session 13: Strengthen Your Physical Activity Plan

Well-rounded physical fitness is made up of four parts: aerobic fitness (cardiovascular), flexibility, muscular strength, and muscular endurance. Each can be achieved by doing different types of activities. Each has its own benefits.

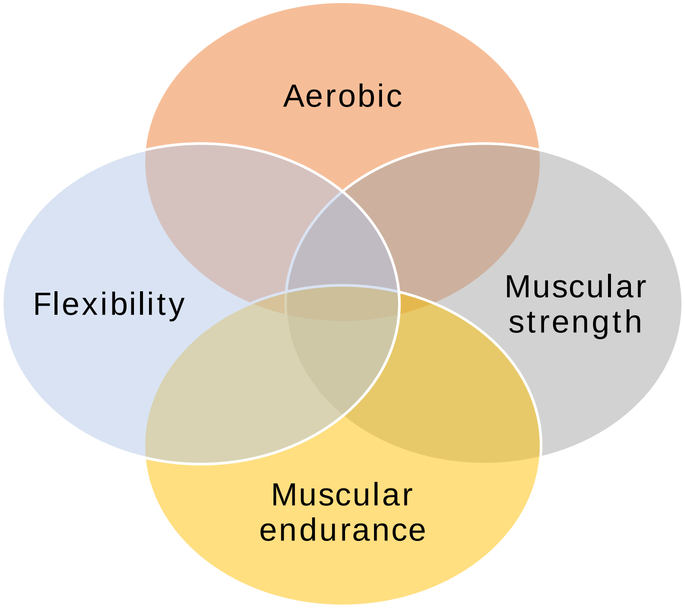

Up until now we have focused on moderate intensity physical activity (like brisk walking) and stretching exercises that improve aerobic fitness and flexibility. Improving muscular strength and endurance is also important, particularly as you get older.

Muscular strength is the ability of your muscles to exert force in a short amount of time. Examples are the strength you need to lift a heavy object (a bag of groceries) or to get up off the floor with ease.

Muscular endurance is the ability of your muscles to move repeatedly without getting tired. It is a measure of how much stamina you have to do things like climbing stairs, raking, and shoveling.

Resistance training (also known as strength training) will improve both muscular strength and endurance. It is any type of physical activity in which you move your muscles against resistance such as that provided by bands or weights.

Copyright 2017 University of Pittsburgh

Stay Stronger Longer:
The Benefits of Resistance Training

Your body is challenged every day to do many tasks that need a certain level of muscular strength and endurance. A safe resistance training program has many benefits and can help you maintain an independent lifestyle as you age.

Helps Prevent Diabetes

- Can improve the body's ability to use insulin and maintain healthy glucose levels.
- Doing both resistance training and aerobic activity has been shown to be the most helpful in preventing diabetes.

Prevents Muscle Loss with Aging

We naturally lose muscle mass with age. Resistance training:

- Can help preserve your muscle mass at any age.
- Firms and tones muscles. The GLB resistance training plan will not make muscles bulky or large or cause weight gain.
- Makes it easier to do daily chores such carrying groceries or doing yard work.
- Helps maintain physical independence.

Helps Maintain and Build Strong Bones

By using your muscles and applying a moderate amount of stress to your bones, resistance training can:

- Increase bone density.
- Reduces the risk of osteoporosis and bone fractures.

Reduces Your Risk of Injury

- Protects your joints from injury.
- Improves balance, coordination, and posture.
- Provides better mobility and balance, which leads to a lower risk of falling or being injured.

Copyright 2017 University of Pittsburgh

Prevents Loss of Muscle Mass with Weight Loss

When you lose weight, it is normal to also lose some muscle mass.

- Resistance training can help reduce the amount of muscle mass you lose, more than aerobic training alone.
- Muscle burns more calories at rest than fat tissue. Having more muscles may help you lose weight and keep it off.

Improves Your Sense of Well-Being

- Improves body image, self-confidence, and sleep.
- Reduces the risk of depression.

All these benefits confirm that maintaining muscular strength and endurance are very important. Strength is needed for activities of daily living such as:

- ✓ Getting in or out of a chair or car
- ✓ Carrying groceries
- ✓ Going up and down stairs
- ✓ Taking care of your personal needs
- ✓ Getting up off the floor

Can you think of any other activities of daily life that need strength and endurance?

____

____

The GLB goal is to do resistance training exercises 2 or more times per week.

Please note: This strength training goal is *in addition* to your goal of 150 minutes of moderate intensity aerobic activity per week. Aerobic activity improves your heart fitness more than strength training.

The 2008 Physical Activity Guidelines for Americans clearly state that, "*in addition to aerobic exercise, adults should perform muscle-strengthening activities that involve all major muscle groups on 2 or more days per week*."

Copyright 2017 University of Pittsburgh

The F.I.T.T. Principle
A Guide for Resistance Training

F.I.T.T. is an acronym used to describe the components of a solid aerobic activity program and was presented to you in session 8. It can also be modified for use with resistance training to guide you in how to improve muscular strength and endurance in a safe manner.

Here are some key definitions:

- Range of motion: the extent of movement around a specific joint
- **Repetition (or "rep"):** taking one exercise through its complete range of motion one time
- Set: a group of repetitions performed before resting. For example, Sadie does a wall push-up. She repeats it 10 times. This is one set.
- RPE (Rating of Perceived Exertion): Session 8 introduced this scale that allows you to rate from 1-10 how hard you feel you are working. For resistance training, aim to be working in the 5-6 range ("somewhat hard" to "hard").

F.I.T.T. for Resistance Training	
Frequency	• 2-3 days/week<br>• Rest at least one day in between
Intensity	• 8-10 exercises*<br>• 10-15 repetitions<br>• 1-3 sets<br>• RPE: "somewhat hard" to "hard"
Type	• Exercise bands, free weights, weight machines, body weight, milk jugs filled with water, etc.
Time	• 15-60 minutes

*At least one exercise should be performed on each major muscle group (e.g. chest, back, quadriceps (front of thigh), hamstrings (back of thigh), calves, shoulders, biceps, and triceps).

It is important that you don't exercise the same muscle group two days in a row. Your muscles need time to rest and recover.

Copyright 2017 University of Pittsburgh

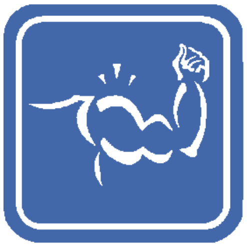

Keep it Safe:
Guidelines for Resistance Training

Check Your Equipment

- Check the resistance band or tube for nicks, worn spots, or cuts.
- Be secure:
    - Always get a good grip on your band or other equipment.
    - If using resistance bands or tubing in a door, it is important to secure the band with a proper anchor for certain exercises.

Warm Up

- Be sure to get your blood flowing by doing a warm-up for 5-10 minutes. This can be a low intensity activity like going for a short walk or walking in place.

Do Only the Exercises That You Are Able to Do Safely

- Stop any exercise that causes pain or makes existing pain worse.
- If an exercise causes pain, speak to your health care provider or a physical therapist.

Do Not Hold Your Breath

- Breathe throughout the exercise. Holding your breath can cause a dangerous rise in blood pressure.
- Exhale more often during the hardest part of the exercise, with each repetition.

Keep Each Movement Slow and Controlled

- Avoid doing any exercises with fast jerky movements.
- For example, when doing a bicep curl, raise the band for 3 seconds and lower the band for 3 seconds.

Maintain Stable and Proper Posture for Each Exercise

- Keep your back straight. Pull your belly button to your spine.
- Avoid rounding your shoulders. Keep shoulders relaxed.

Cool Down

- Do some gentle stretches. Use the stretches from Session 4 as a guide.
- Or, walk around for a few minutes.

Copyright 2017 University of Pittsburgh

Move it Forward:
Guidelines for Progression

Start Slow. Gradual progression is key with resistance training.

- Is this your first time doing resistance training? Or is this your first time back after taking a break?
- Begin with 1 set of 10 repetitions (reps). Gradually work up to doing 3 sets of 10-12 reps. Over time, you can start to increase the number of sets and reps.

Here is a suggested plan for progression:

Week Number	Number of Sets	Number of Reps	Intensity (Rating of Perceived Exertion)	Frequency (days/week)
1-2	1	10	moderate	2-3
3-4	2	8	moderate - somewhat hard	2-3
5-6	2	10	somewhat hard	2-3
7-8	2	12	somewhat hard	2-3
9-12	3	8	hard	2-3
13-24	3	10-12	hard	2-3

Rest

- Rest for 30-60 seconds after each set.
- Do not do strength training on the same muscles two days in a row.
- Do NOT skip the warm up and cool down.

Progression is key

Once your body gets stronger it adapts to the resistance level you have been using. This is the time to move to the next level.

- Increase the intensity of your work-out program. Either increase the number of reps or the amount of resistance (see table above).
- After you have been doing an exercise regularly for at least 2 weeks, check your RPE. On the last rep, you should feel that you are not able to do much more and that you are close to fatigue.

"NO PAIN, NO GAIN" is a MYTH, but slight SORENESS may occur.

Some soreness is normal especially when you are getting started. If you have pain during strength training, STOP. Speak to your health care provider if you have lasting pain.

Copyright 2017 University of Pittsburgh

Group Lifestyle Balance
Resistance Training Program

GLB has created a sample resistance training program for you to try.

Equipment needed:

- Resistance bands or resistance tubing
- A good sturdy chair, preferably without arms
- A wall

You will also need:

- A copy of the suggested exercises with instructions
- A method to record and track your progress

Keep in mind all the points discussed in this session and give it a try.

We will spend time reviewing the exercises together as a group and revisit this topic later on in GLB.

Copyright 2017 University of Pittsburgh

To Do:

The Basics:

- ☐ Keep track of your weight.
- ☐ Record what you eat and drink using your preferred method. Come as close as you can to your calorie and fat gram goals.
- ☐ Record your physical activity using your preferred method. Come as close as you can to your activity goals.
  - Be physically active for at least 150 minutes per week.
  - Aim for an average of at least 50,000 steps per week.
- ☐ Keep moving throughout the day.

What's New: Add Resistance Training

- ☐ Aim to complete the GLB Resistance Training program at least two times per week. Remember: Use resistance training exercises in addition to your aerobic (cardiovascular) program, not to replace it.
- ☐ Keep track of your resistance training program using the GLB Resistance Training Log or your preferred method.

Copyright 2017 University of Pittsburgh

Session 13 Leader Guide:
Strengthen Your Physical Activity Plan

Objectives
In this session, the participants will:

- Discuss the benefits of resistance training.
- Recognize safety issues related to resistance training before, during, and after a session and know when to stop exercising.
- Understand techniques for safe resistance training which emphasize warming up and stretching to prevent injury.
- Learn proper form and technique for each exercise and how to modify the exercises to meet an individual's skill or comfort level.
- Discuss when to increase repetitions or weight.
- Develop an activity plan to use between this and the next session.

To Do Before the Session:

- If providing Food and Activity books; have a supply available for participants.
- Review Food and Activity records from last session; add comments.
- Prepare Session 13 handout for participant notebook.
- Print copies of the **Group Lifestyle Balance Resistance Training Program Poster.** For readability, we suggest copying 8 ½ X 14 (legal size) paper or cardstock. Ideally, have it printed on 11 X 17 paper or cardstock at a print shop.
- Print copies of the **GLB Resistance Training Log**; one per participant.
- Print copies of the handout, **When You Reach Your Weight Goal** to review with participants, as they achieve their weight goals.
- Print copies of the **Group Lifestyle Balance Monthly Calendar**; have a supply available for participants.
- Optional: Visit www.parcph.org for additional information. Determine if you will print copies of the PARC-PH Resistance Training Handout for participants.
- Optional: To become familiar with how to perform resistance training exercises, view the Resistance Training DVD. Ordering information can be found in the GLB Providers Portal.
- Determine if you would like to provide your participants with the Resistance Training DVD to view outside of the group meeting.

Copyright 2017 University of Pittsburgh

Available in the GLB Providers Portal:
https://www.diabetesprevention.pitt.edu/my/login.aspx

- **GLB Resistance Training Log**
- **Group Lifestyle Balance Resistance Training Program Poster**
- **Optional: 2017 GLB Supplies Ordering Information** for how to order the Resistance Training DVD
- **When You Reach Your Weight Goal** handout
- **Group Lifestyle Balance Monthly Calendar**
- **Guidelines for Reviewing Food and Activity Records**

[illegible]

Note: The Leader Guide for this session is written as if the participants have no experience with resistance training before this time. Use your judgment to change your presentation of the session for those participants who are already significantly active.

Group Sharing Time (allow at least 5 minutes)

Let's take a few minutes to see how things went since our last meeting.

Overall, how did it go? What went well? What problems did you have? What could you do differently?

The last session was "Ways to Stay Motivated".
Were you able to apply what you learned at the last lesson to something in your life? If yes, how?

- Did you try one of the ways to stay motivated? Was it helpful?
- Did you take steps to connect with others who support your lifestyle goals? How did it go?
- Did you do things you enjoy but rarely do? How did it go?
  If you changed how you were recording, how did it go?

[Problem solve with the participants to address any barriers.]

[Praise all efforts to self-monitor and to change eating and activity behaviors. Be positive and nonjudgmental. Encourage group discussion.]

Strengthen Your Physical Activity Plan (page 1)

Introduce this session by reminding the participants that a well-rounded exercise program has four components: aerobic, strength, flexibility, and muscular endurance activities.

Each component benefits your body in a different way. Aerobic activity may have the greatest impact on weight control and cardiovascular disease risk, but resistance training

Copyright 2017 University of Pittsburgh

(also known as strength training) will provide additional benefits for your body and overall health. Flexibility is also an important component, especially for mobility. *[Refer participants to the stretching handout in Session 4 as a resource.]*

It is important to remember that your body is challenged everyday to perform many tasks that require a certain level of muscular strength and endurance. A safe resistance training program can help you maintain a lifetime of physical independence by providing many proven benefits.

Stay Stronger Longer: The Benefits of Resistance Training (pages 2 - 3)

There are many health benefits of resistance training. *[Review pages 2 and 3 and include the following:]*

Helps Prevent Diabetes

- The combination of resistance training **and** aerobic exercise will provide even greater benefits. This is why GLB strongly endorses you to do both.

Helps Maintain and Build Strong Bones

- If you already have osteoporosis or reduced bone density, resistance training can lessen its impact.

Reduces Your Risk of Injury

- Specific types of resistance training, such as free weights, resistance bands, and body weight exercises can enhance balance, coordination, and posture.

Prevents Loss of Muscle Mass with Weight Loss

- Your body burns calories while at rest. You use calories for just staying alive (for bodily functions like breathing).
- As you lose muscle, your body burns calories less efficiently. This can lead to increases in body fat and make it difficult to lose weight.

Improves Your Sense of Well-Being

- Regular resistance training can improve sleep patterns and aid in a better night's rest.
- A program of consistent resistance training will allow you to perform activities of daily living with greater ease.
- Enhanced skeletal muscle strength and endurance can help prevent exhaustion and soreness associated with strenuous activities of daily living.

[Review the list of activities of daily living listed. Ask participants to name some other activities that need strength and/or endurance. Encourage group discussion. Note that "taking care of your personal needs" includes any type of self-care such as bathing, getting dressed, cooking, etc.]

Copyright 2017 University of Pittsburgh

The latest recommendations from the American College of Sports Medicine and the American Heart Association states that resistance training exercise should be performed on all major muscle groups (8-10 exercises) 2 to 3 days per week on non-consecutive days. This means you should have at least one day of rest between days of resistance training.

These benefits of resistance training are in addition the benefits of aerobic exercise. Resistance training is only one of the four components of an exercise program along with aerobic, flexibility, and muscular endurance activities. The benefits that each provides are important, and resistance training is meant to complement **NOT** replace aerobic exercise or any of the other components of an exercise program.

The F.I.T.T. Principle: A Guide for Resistance Training (page 4)

We discussed the F.I.T.T. Principle and how it relates to your aerobic fitness program in Session 8. The F.I.T.T principle can also be used to with resistance training to increase muscular strength and endurance in a safe manner. *[Review page 4.]*

Keep it Safe: Guidelines for Resistance Training (page 5)

[Review page 5 and include the following:]

Before your workout:

Check your equipment

- Inspect machines, free weights, and bands/tubes to ensure they are in good condition

Warm up

- Perform low intensity exercises (e.g. walking in place) and gentle stretches as a good warm up.

Other things to consider:

- Get comfortable - Wear clothing that allows you to move freely through a complete range of motion

During your workout:

- Only Do the Exercises That You Are Able to Do Safely
  - Know what joints and muscles each exercise targets
  - Know how to modify or eliminate exercises that may be inappropriate for you

- Do Not Hold Your Breath
- Breathe throughout the exercise, especially when moving against any resistance

Copyright 2017 University of Pittsburgh

- Keep Each Movement Slow and Controlled
    - Always use proper form (do not use momentum)
    - Use slow and controlled movements (never fast or jerky motions)
    - Rest for 30-60 seconds in between sets

After your workout:

- Cool down
    - It is a good idea to do some gentle stretching after the resistance exercises.
    - Allow your body to ease back down to where you were before you began the workout

Move it Forward: Guidelines for Progression (page 6)

Some soreness after a workout is natural but there are steps you can take to minimize soreness. Making slow progressions, resting, and warming-up/stretching are important for helping to minimize soreness.

[Review page 6 and include the following:]

Progression is Key

You should start out performing only 1 set and gradually increase over time. *[Refer to the box that shows the suggested plan for progression.]*

It's important to know when to increase sets, repetitions, and the level of resistance. *[Emphasize the importance of gradually increasing their intensity and review some rules of thumb for knowing when to step it up.]*

Increasing Sets: Once you are able to perform 1 set of 12-15 repetitions with your choice of equipment without any problems, you should increase to 2 sets of 12-15 repetitions, and finally 3 sets of 12-15 repetitions. *[Refer to box.]*

Increasing Weight: A good indication that you are working at the proper weight or tension is that the last two or three repetitions of the set become somewhat challenging.

For example, if you are performing an exercise for 15 repetitions, then the exercise should become somewhat difficult and challenging around the 12th or 13th repetition. However, you should be able to work through the difficulty to complete the 15 repetitions.

- If you have difficulty before you get to the last 2-3 repetitions, then the weight or tension may be too advanced for your current level of training. You may need to work with less weight or tension.

- If you can perform the entire set with no difficulty at the last 2-3 repetitions, then you should increase to the next dumbbell weight or resistance band/tubing tension

Copyright 2017 University of Pittsburgh

increment or add weight to body weight exercises (i.e. holding dumbbells or soup cans in each hand during a Chair Sit-to-Stand).

[Note: While working with the proper dumbbell weight or resistance tubing tension for your workout program, the difficulty experienced in the last 2-3 repetitions of your set should not in any way affect the proper form of the exercise being performed or your breathing.]

Group Lifestyle Balance Resistance Training Program (page 7)

[Distribute the ***Group Lifestyle Balance Resistance Training Program poster****. Aim to allow time to review and try all of the exercises.]*

[Demonstrate proper form and technique for each exercise before the participants give them a try. If the exercise requires resistance band/tubing, make sure that you have the participants perform the exercise WITHOUT it first. Correct form is important and must be corrected to avoid injury. If you notice that the participants cannot perform the exercise without any equipment, then they will definitely be doing the exercise wrong once they have the equipment in hand.]

Although there are cues written on the poster for each exercise, here are a few tips on common mistakes to look for and correct:

1. Wall Pushups:
   - people commonly raise their shoulders up; keep them down and neutral
   - don't allow the back to sag; keep abs tight
2. Seated Row:
   - Again, people may raise their shoulders up; keep them down and neutral
   - Keep spine straight; do a posture check
3. Bicep Curl:
   - Keep elbows tucked into the sides of your body in order to isolate the bicep
4. Tricep Extension:
   - Keep elbow tucked into your side
   - It is not a very big movement
5. Lateral Raise:
   - Keep thumb pointing up
   - Don't lift arm higher than parallel with the floor; raising it higher (more than 90 degrees) can impinge the joint space
6. Chair Sit-to-stand:
   - Don't swing torso for momentum
   - Don't allow knees to come together; keep them in line you're your ankles
   - Don't let knees go over toes; no knock knees

Copyright 2017 University of Pittsburgh

7. Inner Thigh Squeeze:
    - Keep proper posture with spine straight.
    - Remember to breathe
8. Sitting Hip Exercise
    - Keep proper posture with spine straight and abs engaged.
9. Heel Raises:
    - Keep it safe for your balance
    - Use control in lowering heel; don't let them just "plunk" onto the floor

[Distribute the ***Resistance Training Log****. Explain how to record each of the 9 exercises in the Log. Point out that the suggested progression plan seen on page 6 of their handout is also printed on the Log. Tell participants that they will turn in the Log at the next session for the coach to review.]*

*******Optional********

The Resistance Training DVD is an available option for coaches to use. It provides instructions and tips on how to correctly perform some of the exercises. It is not a "follow along" exercise video. Information on ordering the Resistance Training DVD can be found in the GLB Providers Portal.

(Optional) Provide participants with the Resistance Training DVD and encourage them to watch it if they are interested in more information. It is recommended that the Coach watch the DVD so s/he is aware of the content.

Note: Although the GLB resistance training exercise session has been updated, many of the exercises remain the same so the video can be a useful tool.

To Do (page 8)

Turn to page 8 and let's focus on what you can do between now and the next session.

New Things to Practice

Pick a resistance training activity. Add this to your exercise program twice a week. Record it.

[Emphasize the importance of the GLB goal of at least 150 minutes of moderately intense physical activity per week.]

[Announce the day, time, place for the next session. Ask participants to dress in comfortable clothes because they will be reviewing the resistance exercises.]

Copyright 2017 University of Pittsburgh

After the session:

- Weigh participants who did not do so prior to the group meeting.
- Complete data forms and documentation required in your setting.
- Follow your program's protocol for managing absences.
- Review the self-monitoring records. Write brief comments. Be positive and nonjudgmental.
    - Praise all efforts to self-monitor and to change eating and activity behaviors.
    - Highlight any positive changes made that relate to the previous session topic: trying ways to stay motivated, doing something they enjoy but rarely do, connecting with others who support their lifestyle goals, coming close to their calorie, fat gram and activity goals.
    - Refer to **Guidelines for Reviewing Food and Activity Records** available in the GLB Providers Portal.

Copyright 2017 University of Pittsburgh

Session 14:
Take Charge of Your Lifestyle

In GLB, you have learned:

1. Many facts about healthy eating and being more physically active.
2. What makes it hard to change long-standing lifestyle behaviors.
3. Ways to take charge of what's around you (such as cues, thoughts, social situations and more).

We want to keep supporting you.

We want to make sure that your behavior changes work for you, not against you.

So, what lies ahead?

- Ongoing support.
- Learn and practice ways to reach (or maintain) your personal eating, activity, and weight goals, even when it is hard.
- Keep building healthy and positive lifestyle habits.
- Solve problems with fellow group members and other important people in your life.
- Become *your own* Lifestyle Coach.

Attendance matters.

- Many research studies, including the DPP, report that continued contact makes it more likely that you will succeed.

- Continued contact makes it more likely that you will keep your lifestyle behavior change goals on "the front burner".

Copyright 2017 University of Pittsburgh

Physical activity matters.

- Maintaining a physically active lifestyle is a key part of healthy living and aging. Those who commit to staying active typically have better health, physical function, greater independence, and improved quality of life over their lifespan.
- Activity helps with weight loss and weight maintenance. Research studies suggest that those who monitor and maintain regular physical activity tend to eat a healthier diet. Many studies have also shown that remaining physically active (or better yet, increasing the total amount of physical activity minutes performed per week) can help with long-term weight-management. Activity and long-term weight-management go hand in hand.

Group sessions matter.

- They are a great place to talk about challenges and slips, find better ways to tackle problems, and make new plans.
- The group can support you in achieving and maintaining your weight and physical activity goals.

Why do group sessions matter? They:

- Provide structure and a checkpoint ("be accountable") for your weight, healthy eating and physical activity progress.
- Strengthen the self-management skills you have already learned.
- Offer new strategies for long-term success.

Whether or not you have reached your weight and activity goals, we will talk about ways to "get there from here".

The ultimate goal of GLB is to help you build skills that last a lifetime.

Copyright 2017 University of Pittsburgh

Long Term Weight Loss Maintenance: Who succeeds? How do they do it?

You may have heard that no one is successful at keeping weight off.

Here are two examples showing that long-term weight loss is possible.

The National Weight Control Registry (NWCR) was started by obesity researchers Rena Wing, Ph.D. and James O. Hill, Ph.D. in 1994. The registry surveys people in the US who have succeeded at weight loss maintenance in an effort to learn more about their habits.

The NWCR has tracked over 10,000 adults who lost at least 30 pounds and kept it off for at least one year. Results show that the average participant:

- Maintained their weight loss for over five years
- Ate a diet that was low in calories and fat
- Ate the same way on weekdays and weekends
- Watched less than 10 hours of TV per week
- Exercised often, mostly walking

The NWCR also found that the majority of people surveyed weighed themselves at least once a week, and ate breakfast every day.

The U.S. National Health and Nutrition Examination Survey (NHANES) suggests more good news. This survey included over 14,000 adults, of all races and ethnicities.

NHANES reported that people who had ever been overweight or obese and had lost weight:

- Had an average weight loss maintenance of 5% from their highest lifetime weight
- One in three adults kept their weight off for at least one year

Copyright 2017 University of Pittsburgh

Weight Loss Plateaus

After a period of steady weight loss, there may be times when the scale will not budge.

- This can last a few days or a few weeks, despite your best efforts.
- This is common and frustrating, but there are ways to cope.

The biological and behavioral reasons for weight loss plateaus are complex.

Theory and research suggests:

- Biological factors that helped our ancestors survive in ancient times may have something to do with it.
- The less you weigh the fewer calories your body needs to maintain the new, lower weight. A smaller body generally burns fewer calories than a larger one, just as smaller cars burn less fuel than larger ones.
- Motivational plateaus are common after many months of keeping track of eating and activity carefully. You may be having more slips.

Do not give up (or beat yourself up). We will focus on the behaviors that you CAN modify. Try any of the following:

1. Work on "calorie creep". Be mindful of your calorie goal. You may want to go lower for a short period (but not less than 1200 per day).

- Research shows that people often <u>underestimate</u> how much they eat and drink by about 300 – 500 calories per day.
- Bring back the measuring cups and spoons for accuracy.
- Record everything, including "bites, licks, and tastes".
- Limit eating out for a while. Restaurant portions are large and it can be difficult to keep track of calories.

Copyright 2017 University of Pittsburgh

2. Focus on both structured and spontaneous physical activity. Add a few days of resistance training to your weekly routine.

- Research shows that people often <u>overestimate</u> the amount of structured exercise they do.
  - Record daily activity.
  - You may want to set your sights beyond 150 minutes of moderately intense aerobic activity (brisk walking) per week if you are able.
  - Exercise is one of the best ways to boost your long-term weight loss maintenance.

- Research has shown that there are many good reasons to do resistance ("strength") training. As discussed in Session 13:
  - It is good for overall health and can help you maintain and independent lifestyle as you age.
  - Strength training counters the muscle loss that can happen as you lose weight and help with weight management.

- Focus on sitting less at home, work, and play. If you move very little in your daily routine, your weight loss will slow down.

- To sum it up—keep moving in whatever way you can as often as you can.

3. Manage stress and get adequate sleep.

- Practice belly-breathing, meditation, prayer, yoga and other relaxation techniques to decrease stress and avoid eating or being inactive in response to stress.

- Research shows that good sleep can reduce stress and may help with weight control by regulating the hormones that affect metabolism and appetite.

Copyright 2017 University of Pittsburgh

4. Balance your thoughts. Think of signs of progress that don't involve the scale.

- Do your clothes fit better?
- Do you have more energy?
- Do you have better self-esteem or confidence?
- Has your glucose, blood pressure, and/or cholesterol improved?

5. Practice motivational strategies and positive imagery.

- Get a mental picture of yourself being more fit and weighing less.
- Visualize yourself preparing and eating healthy foods.
- Visualize yourself doing physical activities that you like to do.

Take charge of your long-term lifestyle self-management.

- Maintaining contact with GLB will improve your chances of meeting your weight loss and physical activity goals.
- The more often you reach these healthy lifestyle goals now, the more likely you are to continue meeting those goals when GLB ends.
- In other words, "Nothing breeds success like success."

What does "taking charge of your lifestyle" mean to you?

____

____

The bottom line:

If you have had success, you are still a success.

Remind yourself often of all the great things you have accomplished on your journey to better health.

Copyright 2017 University of Pittsburgh

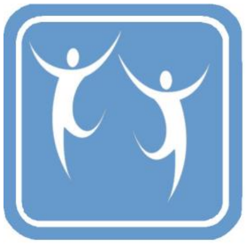

We Will Continue to Work Together as a TEAM

We will count on you to:

- Keep coming to sessions (and let us know when you can't so we can make a plan for you to get materials).
- Do your best to keep reaching your eating and physical activity goals. That includes practicing on your own what you have learned at group meetings.

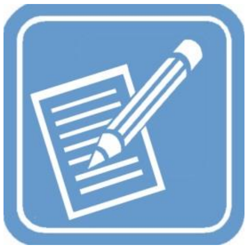

- Keep track of your eating, activity and weight according to the plan you made in Session 12.
- Pay close attention to the things in your life that help or hurt your ability to stick to healthy lifestyle behavior change.
- Share some of your challenges at the group sessions so that everyone can benefit and work on ways to be more successful.
- Keep at it (even when the slope gets slippery).

You can count on us to:

- Continue providing information, tools and coaching support to help you succeed over the long haul.
- Believe you can be independent and successful.
- Always "hang in there" for you.

Renew Your Commitment.

Based on what we have discussed today, and my desire for lifestyle self-management, I commit to attending ongoing GLB sessions. I will work with my lifestyle coach and fellow group members in the ways described above.

Signed: ___________________________________ Date: _________

Lifestyle Coach: ____

Copyright 2017 University of Pittsburgh

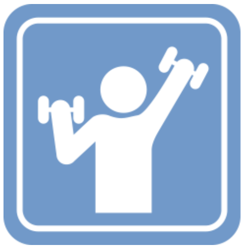

Group Lifestyle Balance: Resistance Training Review

You have been introduced to the importance of resistance training.

Here is a reminder of the many benefits of resistance training:

- Helps prevent diabetes
- Prevents muscle loss with aging
- Help maintains strong bones
- Reduces risk of injury
- Prevents loss of muscle mass during times of weight loss
- Helps to maintain well-being and physical independence

How has it been going with adding resistance training to your routine?

- Have you been able to try the exercises on your own?
- Are you recording it?
- Are you having trouble with any of the exercises?
- Do you have any questions or concerns?

As you move forward in GLB, we strongly encourage you to make resistance training a key part of your activity plan. Aim to:

- Complete the exercises on at least 2 non-consecutive days per week.
- Keep track of your progress in the GLB Resistance Training Log or other method.
- Turn your log in to your coach at the next session.
- <u>Always keep it safe</u>.

Copyright 2017 University of Pittsburgh

To Do:

Check the boxes when you complete each item:

The Basics:

☐ Keep track of your weight.

☐ Record what you eat and drink using your preferred method. Come as close as you can to your calorie and fat gram goals.

☐ Record your physical activity using your preferred method. Come as close as you can to your activity goals.

- Be physically active for at least 150 minutes per week.
- Aim for an average of at least 50,000 steps per week.
- Include resistance training at least 2 times per week.

☐ Keep moving throughout the day.

What's New:

☐ Why is it important for you to keep coming to the group meetings?

____

☐ Review "Weight Loss Plateaus" on pages 4-6. What are you willing to do?

____

Copyright 2017 University of Pittsburgh

Session 14: Resources	Page
When You Reach Your Goal Weight	11

Copyright 2017 University of Pittsburgh

When You Reach Your Goal Weight

Congratulations.

Plan for weight maintenance:

Goal Weight: Decide on a 5-pound weight range you will stay within.

Calories: Find the calories you need to maintain your weight.

It is important to keep track of your eating and activity during your calorie adjustment experiment.

Calories	Fat Grams
1200	33
1500	42
1800	50
2000	55
2200	61
2500	69

Be active: Continue to exercise at a moderate intensity for at least 150 minutes per week. This is a key factor in weight maintenance.

Weigh in: Weigh yourself at least once per week.

Plan to stop weight regain:

If you reach the upper limit of your 5-pound target weight range, take action right away.

My plan to stop weight regain:

- ☐ Record eating and activity every day.
- ☐ Measure foods and beverages.
- ☐ Follow a lower calorie and fat gram goal.
- ☐ Be active for at least 150 minutes per week.
- ☐ Other: ____

Copyright 2017 University of Pittsburgh

Session 14 Leader Guide:
Take Charge of Your Lifestyle

Objectives
In this session, the participants will:

- Understand that although the frequency of GLB meetings is reduced, they will have continued contact and support.
- Understand that some self-monitoring is better than no self-monitoring.
- Describe the benefits of continuing to attend GLB meetings.
- Review scientific evidence regarding why attendance matters.
- Learn the importance of maintaining regular physical activity for long-term success.
- Understand that long-term weight loss maintenance is possible.
- Define "weight loss plateau" and learn strategies to overcome it.
- Renew their commitment to GLB.
- Review resistance training information and practice the exercises.

To Do Before the Session:

- If providing Food and Activity books, have a supply available for participants.
- Review Food and Activity records; add comments.
- Prepare Session 14 handout for participant notebook.
- Print **GLB Resistance Training Log**; one per participant.
- Print copies of the handout, **When You Reach Your Weight Goal** to review with participants, as they achieve their weight goals.
- Print copies of the **Group Lifestyle Balance Monthly Calendar**; have a supply available for participants.
- Decide if your program will provide **Resistance Training Logs** at each session.
- Given the array of self-monitoring tools provided, determine your protocol for review and feedback to the participants. Note: As session frequency decreases, the number of records returned at each session may increase.

Available in the DPP-GLB Providers Portal:
https://www.diabetesprevention.pitt.edu/my/login.aspx

- **GLB Resistance Training Log**
- **Group Lifestyle Balance Resistance Training Program Poster**
- **When You Reach Your Weight Goal Handout**
- **Group Lifestyle Balance Monthly Calendar**
- **Guidelines for Reviewing Food and Activity Records**

*[**Note:** For those participants who have reached their goal weight, review with them the handout entitled **When You Reach Your Weight Goal**. This may be done before or after the group meeting.]*

Copyright 2017 University of Pittsburgh

Note: Allow at least 15 minutes at the end of the session for the resistance training exercises to be revisited.

Group Sharing Time (allow at least 5 minutes)

Let's take a few minutes to see how things went since our last meeting.

Overall, how did it go? What went well? What problems did you have? What could you do differently?
Where you able to take what you learned at the last session and apply it to your lifestyle routine? If yes, how?

Were you able to add resistance training to your physical activity routine? How did it go?

[Problem solve with the participants to address any barriers.]

[Praise all efforts to self-monitor and to change eating and activity behaviors. Be positive and nonjudgmental. Encourage group discussion.]

We will transition to meeting on a monthly basis for the rest of this year. For some people, meeting less frequently with the group may not be an issue of concern, but for others, this change may cause uncertainty and anxiety. The idea is that we will work with you to take charge of your healthy lifestyle behaviors as you move toward your increasing independence. *[Allow for brief group discussion about meeting less often.]*

Take Charge of Your Lifestyle (pages 1-2)

First, let's look at all you have learned in GLB. *[Review "So far in GLB, you have learned"]*

[Review "So, what lies ahead?"]

As you go forward in the program, attending the GLB sessions remains a key component for reaching and maintaining your healthy lifestyle goals. Taking part in the sessions will help you to become more skillful in making long-term healthy lifestyle changes by continuing to provide support, accountability and feedback. This is kind of like a new driver who has been in training, but is now ready to take the wheel, while the coach takes the back seat to continue providing encouragement and support.

[Ask participants to share their thoughts.]

The goal of GLB is to provide you with the information, skills and practice that will last not only well beyond the end of this program, but throughout your lifetime.

Copyright 2017 University of Pittsburgh

[Review "Attendance Matters" and include the following:]

Let's talk a little more about why attendance really does matter. Several research studies have looked at factors related to long-term success in making healthy lifestyle changes, and found regular attendance at group meetings over an extended period of time made a difference in personal success in reaching lifestyle goals.

As we have discussed previously, the participants in the DPP, on average, were successful in reaching their weight and activity goals. The long-term follow up study of the DPP participants, the DPP Outcomes Study, has provided an opportunity for researchers to look at predicting future healthy lifestyle success. Guess what they found?

After one year, those who attended more sessions had a higher percentage of weight loss than those who attended fewer or none.

The results were similar when looking at attendance and physical activity. Participants who attended the most DPPOS sessions were more likely to be at their physical activity goal at one year compared to those who attended fewer or none.

The bottom line is that research findings demonstrate the importance of regular attendance over time in meeting lifestyle goals.

[Review "Physical Activity Matters" (page 2) and include the following:]

In addition to attendance, another key component to long-term healthy lifestyle success is the maintenance of regular physical activity levels.

Research studies have shown that those who continued to maintain their physical activity levels tended to have a healthier diet and were more successful in reaching their weight loss goals than those who did not continue to be physically active.

[Review "Group sessions provide you with structure and support".]

As we move forward, we will continue to count on you to do your part in reaching your goals, and you can count on us to be there to support you.

[Review "When you come to group sessions, you will:" and include the following:]

The format of the monthly meetings will be the same as always. You will weigh-in and give feedback on how you've been doing since your last meeting. The topic of each session will continue to relate to healthy eating, physical activity and behavior change. We will continue to support your lifestyle change efforts.

Some of you have reached your weight loss and physical activity goals, and others have not. The bottom line is that continuing to attend GLB sessions will increase your chances

Copyright 2017 University of Pittsburgh

of meeting the GLB 7% weight loss goal (or your personal weight loss goal) and the 150 minute physical activity goal.

We know that the more often you consistently reach your healthy lifestyle goals, the more likely you are to continue to do so in the future.

Long Term Weight Loss Maintenance: Who succeeds? How do they do it? (page 3)

I'm sure we can all think of someone who has lost weight and then regained it. This lack of weight loss maintenance is likely due to several factors. Maybe the person lost weight by following a fad diet or one with many "forbidden foods" that would be difficult (or impossible) to stick with long-term. Maybe the person wasn't exercising or didn't have support for his/her efforts.

The bottom line is that long-term weight loss maintenance is possible. Do you know anyone who has done so?

[Review the information on page 3. If participants are interested in additional information about the NWCR, the website is on page 3.]

The good news is that GLB is designed to promote long-term weight maintenance:

- We promote gradual weight loss of about 1-2 pounds per week by using a moderate decrease in calories and fat (no fad diets).
- We encourage self-monitoring as a way of being aware of and changing eating and activity behaviors.
- You design your own physical activity plan and you do activities of your choice at the place and time that works for you. You are also moving more in your everyday life. This is something you can do for a lifetime.
- You design a healthy eating pattern that works for you….without any "forbidden foods". This is something you can do for a lifetime.
- GLB is a 12 month program. This allows for ongoing support of your behavior change efforts.

Research indicates the key to keeping weight off is maintaining the behaviors that allowed weight loss to begin with: social support, self-monitoring, low-calorie eating, and regular physical activity.

Weight Loss Plateaus (pages 4-6)

[Review information on pages 4-6. Encourage group discussion.]

[Ask participants to answer the question on the bottom of page 6. Ask participants to share their answers, if comfortable doing so.]

Copyright 2017 University of Pittsburgh

We Will Continue to Work Together as a Team (page 7)

In Session 1 you signed a lifestyle contract.

Now it is time to renew your commitment to your efforts to reach and maintain your healthy lifestyle behaviors.

[Review page 7. Ask participants to renew their commitment to GLB by agreeing to continue to attend the sessions and to work with their coach in the ways described in the agreement.]

If you are ready to renew your commitment to the program, please sign the agreement on page 7. I will sign also.

We are very excited about the next phase of GLB and remain committed to continuing to work with you to reach your healthy lifestyle goals.

Group Lifestyle Balance: Resistance Training Review (page 8) Allow at least 15 minutes.

[Review the information on page 8.]

Practice each exercise.

[Optional: Collect completed Resistance Training Logs and distribute blank ones. Explain that they will turn in the Log at the next session for the coach to review.]

To Do (page 9)

Turn to page 9 and let's focus on what you can do between now and the next session.

[Ask participants to look at page 11, ***When you Reach Your Goal Weight****. Explain that when they want to stop losing weight, whether at the GLB goal of 7% or their own personal goal, you will review this page with them. Make sure participants understand that they do not have to stop losing at 7%; rather, they may continue to work toward their personal weight goal as discussed in Session 9. Emphasize the importance of having a plan to stop weight regain.]*

*[**Note:** At each session from now on, review this handout with those participants who have reached their goal weight. This may be done before or after the group meeting.]*

Copyright 2017 University of Pittsburgh

[Announce the day, time, and place for the next session.]

After the session:

- Weigh participants who did not do so prior to the group meeting.
- Complete data forms and documentation required in your setting.
- Follow your program's protocol for managing absences.
- Follow your program's protocol for reviewing and commenting on the various self-monitoring records.
- Review the self-monitoring records. Write brief comments. Be positive and nonjudgmental.
    - Praise all efforts to self-monitor and to change eating and activity behaviors.
    - Highlight any positive changes made that relate to the topic of the last session before the records were collected.
    - Refer to **Guidelines for Reviewing Food and Activity Records** available in the GLB Providers Portal.
- If collecting **Resistance Training Logs,** review them**.** Write brief comments. Be positive and encouraging.

Copyright 2017 University of Pittsburgh

Session 15:
Mindful Eating, Mindful Movement

Are there times when you realize that you are still eating and simply haven't noticed that you are not even hungry anymore?

Do you ever get to the end of a long day and realize how little you have moved and how much time you spent sitting?

These patterns are common.

All of us can benefit from practicing being more "mindful" of our eating and movement habits throughout the day.

Being more mindful can help us to reach or maintain our healthy lifestyle goals. It may also help our physical and mental well-being.

Let's start with mindful eating.

1. Mindful eating means to eat slowly and with awareness.
   - Tune into all your senses while you eat.
   - Be aware of your level of hunger and fullness.

2. Mindful eating helps with weight management.
   - Your brain has time to register that you are full. This takes about 20 minutes. If you eat too quickly, you are more likely to overeat.
   - When you notice and enjoy every mouthful, you will feel more satisfied at the end of the meal.

Copyright 2017 University of Pittsburgh

3. Prepare to eat slowly and mindfully.

- Create a calm eating environment. This lets you pay attention to what you are eating. Put flowers, plants, or a candle on the table. Turn on calming music.
- Decrease distractions. Avoid (or limit) eating while doing other things at the same time. Turn off the TV, electronics, phones, and put away reading material.
- Sit down and relax. Avoid (or limit) eating while standing in the kitchen or when on the run.
- Appreciate the moment. When you first sit down to eat, pause for a minute. Take a few deep breaths. Say grace, if that is your tradition. Or think about all of the hands that brought the food to your table.

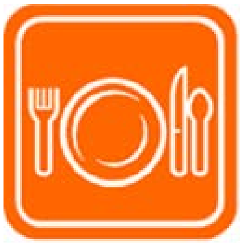

4. Focus on your food. Eat slowly, with awareness. Try to make each meal last at least 20 minutes.

- Look at the food on your plate. Notice the colors, shapes, and aroma of the food.
- Take small bites. Be aware of how the food feels in your mouth, the texture. Take time to savor the taste of the food.
- Chew slowly and be aware of how much you are chewing. Try to chew at least 5 times more per mouthful than you usually do.
- Finish chewing and swallowing each bite before you put more food on your fork.
- Put your fork down for 10-15 seconds after a few bites. Pause to take a sip of water or take a deep breath. These small changes will really help you extend your mealtime.
- You may be distracted by others or your own thoughts. Pause, and return your focus to the food in front of you.

5. Stay tuned to your level of hunger and fullness.

- Several times during the meal, check your level of hunger and fullness. Do you need more food? Do you want more food?
- Are you still eating just because there is food left on your plate?
- Are you ready to stop eating? If so, push away your plate and pay attention to any physical sensations.

Copyright 2017 University of Pittsburgh

6. Practice eating mindfully with others and alone.

- Take time to enjoy one another's company.
- You may want to try eating a meal in silence. Even short periods of silence can be very calming.

> Enjoy each and every bite of your food.
>
> Practice eating slowly and mindfully.
>
> This may help you feel physically and mentally satisfied with a smaller amount of food.

What can you see yourself doing to improve your mindful eating experience?

____

____

Copyright 2017 University of Pittsburgh

Mindful Movement

Life is busy. Many people tell us that they feel stressed and overwhelmed by all they need to accomplish at home, at work, for their family and community.

For some of you, this means long periods of sitting with little time spent stretching, moving, or doing much planned physical activity.

Practicing mindful movement can help you break this pattern.

1. Mindful movement means to be aware of your total movement throughout the day.
   - Tune in to how much sitting you do.
   - Add movement and activity when and where you can.

2. Mindful movement also means doing physical activity (such as a simple 15-20 minute walk) with purpose and increased awareness of the full experience.
   - Tune into your posture and breathing as you prepare for walking (or any other exercise). Notice how your body feels.
   - Pay attention to the rhythm of your breathing and how it changes as you move.
   - Notice the movement of your feet. Feel how your feet make contact with the ground and then move through the air. Also notice the contact between your feet and your socks or shoes as you move.
   - Do you notice any other physical sensations? Some people get distracted by changes in body heat or other minor discomforts. Try to counter these thoughts by reflecting on the opportunities that movement allows. Gently bring your attention back to the present moment.
   - Appreciate the outdoors. Tune into the sights and sounds of nature.

Copyright 2017 University of Pittsburgh

3. Pay attention to how your body feels when moving after long periods of sitting.

   - Be aware of how you feel when you make a shift from long periods of sitting to stretching, moving, and being active.

4. Stop and consider all the amazing work your body does day in and day out. Think about adding movement to your routine that increases awareness of and appreciation for your physical well-being (such as simple stretches or gentle yoga).

   - Use mindful movement to relax, release tension, and reduce stress.
   - Use mindful awareness to notice how often you give yourself permission to be physically active or to take movement breaks during long bouts of sitting.

> Use mindfulness to identify <u>any</u> positive feelings you get from movement.

Moving mindfully means being alert to any and all opportunities for movement. This may include sitting less or making time for planned physical activity you enjoy.

- Use your pedometer to stay aware of how much you move during the day. What happens to your pedometer steps as you become more mindful about movement?
- Use the Active Head Challenge (Session 8) to help you find ways to add as many short bursts of "spontaneous activity" as you can. Do you feel better at the end of an Active Head day?
- How can you turn a spare minute into an "active minute"?
- List ways you could put mindful movement into a typical day.

  ____

  ____

Copyright 2017 University of Pittsburgh

To Do:

Check the boxes when you complete each item:

The Basics:

☐ Keep track of your weight.

☐ Record what you eat and drink using your preferred method. Come as close as you can to your calorie and fat gram goals.

☐ Record your physical activity using your preferred method. Come as close as you can to your activity goals.

- Be physically active for at least 150 minutes per week.
- Aim for an average of at least 50,000 steps per week.
- Include resistance training at least 2 times per week.

☐ Keep moving throughout the day.

What's New:

Practice Mindful Eating

☐ Create a calm eating environment. See suggestions on page 1.

☐ Practice eating slowly and mindfully. Try to make each meal last about 20 minutes. See suggestions on page 2.

☐ When practicing mindful eating were you aware of any new thoughts or feelings? ______________________________________

☐ On a scale of 1-5 (1=none and 5=a lot) rate your experience of:

Physical fullness	1	2	3	4	5
Enjoyment of the food	1	2	3	4	5
Overall satisfaction	1	2	3	4	5

Practice Mindful Movement

☐ Be aware of how much time you spend sitting.

☐ Add movement and activity when and where you can.

☐ When practicing mindful movement were you aware of any new thoughts or feelings? ______________________________________

☐ Try some of the strategies listed on page 5. How did it go?

____

Copyright 2017 University of Pittsburgh

Session 15 Leader Guide:
Mindful Eating, Mindful Movement

Objectives
In this session, the participants will:
- Analyze and describe current eating and activity behaviors.
- Define "mindful eating" and "mindful movement".
- Describe the negative effects of eating mindlessly.
- Discuss the benefits of eating slowly and mindfully.
- Review the techniques for eating mindfully.
- Practice eating slowly and mindfully.
- Discuss the benefits of mindful movement.
- Review the techniques for moving mindfully.
- Make a plan for applying mindful eating and mindful movement behaviors.

To Do Before the Session:
- If providing Food and Activity books, have supply available for participants.
- Prepare Session 15 handout for participant notebook.
- Review Food and Activity records; add comments.
- If collecting **Resistance Training Logs**; add comments.
- Print copies of the handout, **When You Reach Your Weight Goal** to review with participants, as they achieve their weight goals.
- Print copies of the **Group Lifestyle Balance Monthly Calendar**; have a supply available for participants.
- If providing **Resistance Training Logs;** have a supply available for participants.
- Given the array of self-monitoring tools provided, determine your protocol for review and feedback to the participants. Note: As session frequency decreases, the number of records returned at each session may increase.
- Purchase napkins and food items. Suggest mini crackers, low-fat cheese cubes, jumbo raisins, or a small piece of chocolate.
- Optional: For additional information on mindful eating, visit http://thecenterformindfuleating.org/
- Optional: For additional information on mindful movement, visit https://www.mindful.org/

Available in the DPP-GLB Providers Portal:
https://www.diabetesprevention.pitt.edu/my/login.aspx
- **GLB Resistance Training Log**
- **When You Reach Your Weight Goal Handout**
- **Group Lifestyle Balance Monthly Calendar**
- **Guidelines for Reviewing Food and Activity Records**

Copyright 2017 University of Pittsburgh

*[**Note:** For those participants who have reached their goal weight, review with them the handout entitled **When You Reach Your Weight Goal**. This may be done before or after the group meeting.]*

Group Sharing Time (allow at least 5 minutes)

Let's take a few minutes to see how things went since our last meeting.

Were you able to apply what you learned at the last lesson to something in your life? If yes, how? How did it go?

How does it feel to be working on your healthy lifestyle behaviors on your own for longer periods of time between group meetings?

[Problem solve with the participants to address any barriers.]

[Praise all efforts to self-monitor and to change eating and activity behaviors. Be positive and nonjudgmental. Encourage group discussion

Mindful Eating (pages 1-3)

Throughout GLB we have talked about healthy eating. But it is important to not only consider ***what*** we eat, but ***how*** we eat.

Today's lesson will first focus on the concept of what is called "mindful eating". Then we will talk about "mindful movement".

[Review page 1 and include the following:]

1. **Mindful eating means to eat slowly and with awareness**. It means you are aware of not only what food is on the plate, but aware of the entire experience of eating as well. It tends to enhance the whole experience of eating.

   Mindful eating is being totally focused on each sensation that happens while eating; chewing, tasting, swallowing, and savoring each bite.

   Most people don't think about, or even enjoy, the taste of what they are eating; they are just focused on the next bite and/or what is happening in the environment (TV, computer, driving, reading, etc). This is called "mindless eating". Some call this "distracted eating".

   What are the effects of mindless eating? *[Answers: Decreased awareness of the amount of food eaten, less enjoyment of food, decreased ability to assess level of hunger or fullness, disruption of the mind-body connection so signals that regulate food intake may not be sensed, leading to overeating.]*

Copyright 2017 University of Pittsburgh

2. **Mindful eating helps with weight management**.

   How long does it usually take you to eat your meals and snacks? You may want to record how long it takes you to eat your meals this coming week. You can give feedback about this at our next meeting.

   Research shows that when people eat slowly and mindfully they tend to eat less food. Enjoying each bite leads to being more satisfied at the end of the meal, often with less food and fewer calories.

   Other benefits of mindful eating:
   - increases awareness of the amount of food being eaten
   - helps with learning how to ignore the urges to snack that aren't associated to hunger
   - can reduce binge eating
   - enhances the whole experience of eating

3. **Prepare to eat slowly and mindfully**.

   Where do you typically eat your breakfast? Lunch? Dinner? Snacks?

   We talked in Session 6, Take Charge of What's Around You, that one way to help manage cues that make you want to eat is to decrease the number of places you eat. Have you done this? How did it work for you? If you haven't, try it.

   Can you describe times when you have eaten while distracted? *[Examples include watching TV, working on the computer, driving, reading, and cooking]*
   - What usually happens to the amount of food being eaten while distracted? *[usually more is eaten]*
   - Do you tend to get more or less enjoyment from food? *[usually less is eaten]*
   - Do you feel you tend to be more or less aware of what and how you're eating? *[usually less aware of what and how much is eaten]*

4. **Focus on your food.**
   Here are some ways to make your meals last least 20 minutes. *[Review this section. Encourage group discussion.]*

5. **Stay tuned to your level of hunger and fullness**.
   Check your level of hunger before you start eating and several times during the meal. You want to rate your hunger or fullness on a scale from 1-5. What can you see yourself doing to eat slowly and mindfully?

Copyright 2017 University of Pittsburgh

6. **Practice eating mindfully with others and alone.** *[Review and encourage discussion. Ask participants to answer the question on page 3 and to share their responses, if comfortable doing so.]*

Now, we are going to practice eating slowly and mindfully.

The purpose of this activity is to exaggerate the act of tasting and eating by slowing down and focusing moment by moment on all of the sensory qualities associated with the food.

The goal is to take 60-90 seconds to eat each food item.

[Distribute napkins with food item(s). Options include 1 jumbo raisin, 1 cracker, and/or 1 cheese cube, or one small piece of chocolate. Use the following script to guide participants through this experience.]

First, look at what you are about to eat. What is it? How does it look? Where does it come from? How do you feel about putting this food into your body? How does your body feel anticipating eating at this moment?

Tune into your breathing as you look at the food, knowing you are about to take it into your mouth and body. Focus only on this food.

Put the item into your mouth. Do not begin chewing yet. Feel the food in your mouth. Be aware of the sensations in your mouth; taste, texture, temperature, etc.

Now chew slowly and focus your energy on the food's taste and texture. You might try chewing longer than you normally do to fully experience the process of chewing and tasting. Focus only on the food. Notice each movement of your jaw and tongue.

Note any impulse you have to rush through this mouthful so that you can go on to the next. Let such impulses remind you that you already have food in your mouth, so you needn't go on to the next bite to have a complete experience of eating. Stay in the present moment with this mouthful, rather than rushing on to the next.

Before swallowing, be aware of the intention to swallow. Then feel the actual process of swallowing so that you become more conscious of this action. Now be aware of no longer feeling any sensation of food remaining in the mouth.

Take a deep breath and exhale.

[Ask participants to comment on the experience. Repeat this exercise if using additional food items.]

This was an exercise in exaggerated eating and we don't anticipate that every meal will be eaten in this fashion.

We should find pleasure in food, not guilt. Eating slowly and mindfully will help identify what is enough to nourish and sustain your body.

Copyright 2017 University of Pittsburgh

Mindful eating takes discipline and practice. Try to eat one meal or snack mindfully every day. Even eating the first few bites mindfully can help break the habit of wolfing it down without paying attention. Even very healthy eaters must remind themselves from, time to time, to eat slowly and mindfully.

The objective is to help to bring awareness to how much you are eating, how fast, and how your body feels during and after the meal. Everyone's minds wander easily. If this happens, just return to the awareness of that taste, chew, or swallow.

In previous sessions, we have suggested that you try to make your meal last 20 minutes. This is will also help you with your mindful practice. The goal is for you to feel both physically and mentally satisfied at the end of the meal.

During which meal or snack will you begin to practice eating mindfully?

I look forward to hearing about your experiences with eating slowly and mindfully when we meet next month.

Mindful Movement (pages 4-5)

Now let's talk about how mindfulness can apply to movement as well. It can be a way to enhance your physical activity experience.

[Review information on pages 4-5 and encourage group discussion. Include the following:]

1. **Mindful movement means to be aware of your total movement throughout the day.**

   We have talked about this since early in GLB. Do you feel that you are more aware? How are you doing with adding movement and activity to your life?

2. **Mindful movement also means doing physical activity with purpose and increased awareness.**

   Mindful movement also involves an increased awareness of how physical activity affects your body. Try to experience and enjoy movement with every step. Focus on the process, not just reaching the goal of getting a certain number of minutes of activity.

   While you are active, tune into all your senses. What do you see around you? What do you hear? Smell? Feel?

   [Review and discuss each bullet point.] Has anyone ever done this?

   What are some minor discomforts or annoyances you have (or might) experience while being active? What was your response? Were you able to overcome this?

Copyright 2017 University of Pittsburgh

Mindful movement helps you stay fully engaged in what you're doing; experiencing and enjoying each step.

3. **Pay attention to how your body feels when moving after long periods of sitting.**

   What sensations do you experience?

   Before you exercise, think about how you are feeling. Choose an exercise that will best satisfy your present needs. Example: Would you prefer to walk outside and enjoy being alone in nature or would you rather go to an aerobic dance class and enjoy the music and being with others?

4. **Stop and consider all the amazing work your body does. Increase awareness and appreciation for your physical well-being.**

   People often are critical of their bodies. Being mindful while moving is an opportunity to appreciate your body and all it does for you.

   Think about all your body does just to keep you balanced and upright as you move.

   Mindful movement also involves increased awareness of how activity affects your mood, thinking, energy level, and level of stress.
   - Have you noticed any positive thoughts and feelings with movement?
   - Do you notice that you feel energized or less stressed while being active?
   - Notice as you are exercising, is your mind clear or dull; busy or calm?

   During your time in GLB, have you experienced a shift in your attitude about being active? In what way?

   Some people exercise because they feel they have to, it's required by GLB, or they feel guilty if they don't. Mindful movement can help people learn to enjoy being active for its own sake. It can help you to become more aware of the feelings of health and well-being that come from an active lifestyle.

   Mindful movement is not about becoming a perfect exerciser. You still will have days that you struggle with motivation to get up off the couch and take a walk. But practicing mindful movement will help you enjoy all the positive feelings and sensations you get from being active.

   [Review and discuss the questions at the bottom of the page. Encourage discussion.]

I look forward to hearing about your experiences with mindful movement at our next meeting.

To Do (page 6)

Copyright 2017 University of Pittsburgh

Turn to page 6 and let's focus on what you can do between now and the next session.

[Review and encourage participants to try at home what was discussed in class. Ask them to stay open-minded and try it.]

[Announce the day, time, and place for the next session.]

After the session:

- Weigh participants who did not do so prior to the group meeting.
- Complete data forms and documentation required in your setting.
- Follow your program's protocol for managing absences.
- Follow your program's protocol for reviewing and commenting on the various self-monitoring records.
- Review the self-monitoring records. Write brief comments. Be positive and nonjudgmental.
    - Praise all efforts to self-monitor and to change eating and activity behaviors.
    - Highlight any positive changes made that relate to the topic of the last session before the records were collected.
    - Refer to **Guidelines for Reviewing Food and Activity Records** available in the GLB Providers Portal.
- If collecting **Resistance Training Logs,** review them**.** Write brief comments. Be positive and encouraging.

Copyright 2017 University of Pittsburgh

Session 16:
Manage Your Stress

Stress is part of life. However, you can learn better ways to take care of yourself when faced with stress.

Stress is not always bad. Some stress can make life interesting and exciting (for example births, weddings, a new job).

Too much stress can have a negative effect on your body and mind. It may lead to physical symptoms, high blood glucose levels, or feeling moody (for example, anxious, angry, depressed).

Stress comes in many forms and may be related to:

- Health (pain, chronic medical conditions)
- Basic needs (work, housing, transportation, finances, even the weather, traffic, noise, or crime in your community)
- Social conflicts (fighting with a spouse or partner, children, friends, coworkers, neighbors)
- Emotional troubles (worry, sadness, grief, anger, guilt)

Take a minute to answer these questions:

1. How often do you feel stressed? ☐ Often ☐ Sometimes ☐ Seldom

2. How do you know you are stressed? What is it like for you?

____

3. Name some things that are making you feel stressed right now.

____

4. What are some *unhealthy* ways you deal with stress?

____

5. What are some *healthy* ways you deal with stress?

____

Copyright 2017 University of Pittsburgh

Too much stress can make it harder for you to reach and keep a healthy weight or stay physically active. Under stress you may withdraw from healthy activities and go back to old habits.
For example, you may:

- Move less (watch too much TV, play on the computer, sleep more)
- Eat or drink too much (often foods with a lot of fat and sugar or more alcohol)

Instead, use healthy ways to cope with stress. Check the ideas below that you would like to try.

1. Prevent or reduce stress when you can. Are there one two areas you are willing to work on?

   ☐ Practice saying "No." Say "Yes" only when it is important to *you*.

   ☐ Share some of your work or duties with others.

   ☐ Set goals you can reach.

   ☐ Take charge of your time. Be realistic. Get organized. Make schedules that are likely to work.

   ☐ Use problem solving:
   - Describe the problem in detail.
   - Brainstorm your options.
   - Pick one option to try.
   - Make an action plan.
   - Try it. See how it goes.

   ☐ Plan ahead. Think about the kind of situations that cause stress for you. Plan ways to get little breaks from chronic stress. This may help you cope better over the long run.

   ☐ Keep things in perspective. Think of all the good things in your life. Maintain a positive attitude.

   ☐ Reach out to people.

   ☐ Focus on wellness.
   - Find healthy foods that also comfort you (nutritious snacks, special teas).
   - Get enough sleep.

Copyright 2017 University of Pittsburgh

- Practice ways to relax. Do yoga. Listen to music.
- Nurture your spiritual health. Many people do so with religious activities, or through art, music or being in nature.
- Laugh often. Enjoy humor.

☐ Re-commit to your physical activity routine. Find something you are willing and able to do. Get started.

2. **When you can't prevent stress**, try to manage it better. Catch yourself feeling stressed as early as you can. Take a 10-minute "time out."

   ☐ Stand. Stretch. Move those muscles anyway you can.

   ☐ Take care of yourself. Take 10 minutes just for YOURSELF. Water the garden, take a bath or shower, talk to a friend, organize your tools or pictures, read the funnies, walk the dog.

   ☐ Do something that quiets your mind <u>and</u> relaxes your body. Meditate, pray, do yoga, listen to soothing music, practice "belly breathing."

   ☐ Other ideas. ____

Belly breathing is how newborn babies and sleeping adults breathe. The breaths are slow and deep and fill the belly not the chest.

<u>To learn belly breathing:</u>
1. Sit comfortably. Keep your spine straight. Bend your knees, with your feet on the floor about eight inches apart.
2. Put one hand on your belly. Put the other hand on your chest.
   - Breathe in slowly and deeply through your nose. Let your belly push your hand up and out.
   - Gently press down on your belly as you breathe out through your nose.
   - Let your chest move just a little. It should follow the movement of your belly.

Practice belly breathing when you notice yourself getting tense. You can do it sitting down or standing still. Try it in rush hour traffic or while waiting in line.

Good breathing is "belly breathing." The breaths are slow and deep and fill the abdomen, not the chest. This is how newborn babies and sleeping adults breathe.

Copyright 2017 University of Pittsburgh

Manage Sleep to Manage Stress

"Sleep is the best meditation**."** ~ Dalai Lama

A good night's sleep is as important for your body and mind as eating, drinking, and breathing.

How do you feel when you get *enough* sleep? ____________________

How do you feel when you get *too little* sleep? ___________________

How do you feel when you get *too much* sleep? __________________

One in three Americans doesn't get enough sleep.

- Many adults say they get less than the recommended 7-9 hours.
- Only one in five adults say their sleep is good or excellent.
- Poor sleep makes it harder to cope with stress, which further disturbs sleep, leading to a bad cycle.
- Poor sleep increases risk for (and makes it harder to manage):
    - Obesity (sleep helps balance hormones that affect metabolism, appetite, and weight). This may lead to sleep apnea and a slippery slope of health decline.
    - Pre-diabetes
    - Heart disease
    - Depression

Sleep problems steal your energy and make it harder to work on your eating and physical activity habits during the day.

Start by taking a close look at your sleep habits and patterns. Is there room for improvement?

Not everyone needs the same number of hours. What is your ideal "sleep number"? _________.

Track your sleep this week and see what amount helps you feel your best.

Copyright 2017 University of Pittsburgh

Tips for Getting Better Sleep

Think about behaviors related to sleep and bedtime that you might be able to change. Check one or two that you can try.

- ☐ Be active. Regular physical activity helps many people fall asleep faster, sleep longer, and spend less time awake during the night.
- ☐ Use routines that help you relax each night before bed.
- ☐ Don't go to bed unless you are sleepy.
- ☐ Turn off all electronics before getting in bed. Even better, keep them all out of the bedroom.
- ☐ If you are not asleep after 20 minutes, then get out of bed. Do something relaxing in another room if possible.
- ☐ Go to bed and get up at roughly the same time each day.
- ☐ Avoid taking naps. Or keep them short (less than one hour and before 3:00 pm).
- ☐ Avoid large amounts of caffeine.
- ☐ Avoid large amounts of alcohol or nicotine.
- ☐ Do not go to bed hungry or overly full.
- ☐ Avoid sleeping pills, or use them with caution.
- ☐ Make your bedroom quiet, dark, and a little bit cool.

Psychiatric conditions such as major depression, anxiety disorders, or post-traumatic stress disorder (PTSD) affect sleep in many different ways.

Talk to your health care provider if you want more help. In some cases, a sleep evaluation, behavioral sleep intervention, or medications may be needed.

Copyright 2017 University of Pittsburgh

Laughter is Good Medicine

"With mirth and laughter let old wrinkles come."~William Shakespeare

"Laughter is internal jogging." ~Norman Cousins

Does a laugh a day keep the doctor away? Much more research needs to be done, but more and more experts believe that strong laughter (about 10-15 minutes or more each day) can help:

- ☐ Ease stress
- ☐ Boost your immune system
- ☐ Lower your blood pressure
- ☐ Lift your mood
- ☐ Burn a few calories

Laughter may also help your brain and hormones handle blood sugar. Here is what one Japanese study found:

- People with type 2 diabetes were given a meal to eat. Then they watched a live comedy show. Their blood sugar was measured two hours later.
- At another time, the same people ate the same meal. But afterwards, they listened to a boring lecture. Again, their blood sugar was measured.
- Can you guess what happened? Their blood sugar went up less after watching the comedy show.

Try your own laughter experiments. Look up "Laughter Yoga" on You Tube, watch funny movies, listen to funny radio shows, get together with friends who enjoy laughing and see what happens.

Copyright 2017 University of Pittsburgh

To Do:

Check the boxes when you complete each item:

The Basics:

☐ Keep track of your weight.

☐ Record what you eat and drink using your preferred method. Come as close as you can to your calorie and fat gram goals.

☐ Record your physical activity using your preferred method. Come as close as you can to your activity goals.

- Be physically active for at least 150 minutes per week.
- Aim for an average of at least 50,000 steps per week.
- Include resistance training at least 2 times per week.

☐ Keep moving throughout the day.

What's New:

☐ Practice ways to prevent or reduce stress (pages 2 and 3). Did it work? ____

☐ Practice relaxation techniques at home and/or at your worksite. Find what works best for you. ____________________________

☐ Find your best sleep number. Monitor and track your sleep hours. What did you learn? ______________________________________

☐ Try adding more laughter to your life. Did you notice a difference in how you feel? _______________________________________

☐ To become more aware of how much time you spend sitting, fill in the 7-Day Sitting Diary for one week (see next page). Have it ready for your next session.

Copyright 2017 University of Pittsburgh

7-Day Sitting Diary

What's in Your Week?

Do you really know how much time you spend sitting? There is no better way to find out than for you to keep track of it.

For one week, please keep track of your sitting time by filling in this 7-Day Sitting Diary (see next page).

Write the abbreviation for the day of the week below each numbered day.

Shade in each 30-minute block in which more than 15 minutes was spent sitting (exclude sleep time).

You may want to use different colors to track job sitting (black) versus non-job sitting (red).

Start date ______________________________________

End date _______________________________________

Copyright 2017 University of Pittsburgh

Time	Day 1	Day 2	Day 3	Day 4	Day 5	Day 6	Day 7
12:00AM							
12:30AM							
1:00AM							
1:30AM							
2:00AM							
2:30AM							
3:00AM							
3:30AM							
4:00AM							
4:30AM							
5:00AM							
5:30AM							
6:00AM							
6:30AM							
7:00AM							
7:30AM							
8:00AM							
8:30AM							
9:00AM							
9:30AM							
10:00AM							
10:30AM							
11:00AM							
11:30AM							
12:00PM							
12:30PM							
1:00PM							
1:30PM							
2:00PM							
2:30PM							
3:00PM							
3:30PM							
4:00PM							
4:30PM							
5:00PM							
5:30PM							
6:00PM							
6:30PM							
7:00PM							
7:30PM							
8:00PM							
8:30PM							
9:00PM							
9:30PM							
10:00PM							
10:30PM							
11:00PM							
11:30PM							

Copyright 2017 University of Pittsburgh

Session 16 Leader Guide: Manage Your Stress

Objectives
In this session, the participants will:

- Discuss stress and how it affects their lifestyle habits.
- Describe different aspects of their stress experience.
- Describe how they cope with stress currently and determine whether these strategies are healthy or unhealthy.
- Consider the ways in which stress is a barrier to making healthy changes in eating and exercise behaviors.
- Review and discuss ways to "take charge" of their personal response to stress. Group members will identify ways to reduce or prevent stress. They will also discuss ways of managing stress in the moment.
- Practice relaxation techniques, specifically belly breathing.
- Consider the importance of good sleep habits and learn tips for improving sleep.
- Consider the healthy effects of humor and laughter, and "making time" to laugh.

To Do Before the Session:

- If providing Food and Activity records, have a supply available for participants.
- Prepare Session 16 handout for participant notebooks.
- Review Food and Activity Records; add comments.
- If collecting **Resistance Training Logs**; add comments.
- Print copies of the handout, **When You Reach Your Weight Goal** to review with participants, as they achieve their weight goals.
- Print copies of the **Group Lifestyle Balance Monthly Calendar**; have a supply available for participants.
- Given the array of self-monitoring tools provided, determine your protocol for review and feedback to the participants. Note: As session frequency decreases, the number of records returned at each session may increase.
- If providing **Resistance Training Logs;** have a supply available for participants.

Available in the DPP-GLB Providers Portal:
https://www.diabetesprevention.pitt.edu/my/login.aspx

- **GLB Resistance Training Log**
- **When You Reach Your Weight Goal Handout**
- **Group Lifestyle Balance Monthly Calendar**
- **Guidelines for Reviewing Food and Activity Records**

Copyright 2017 University of Pittsburgh

*[**Note:** For those participants who have reached their goal weight, review with them the handout entitled **When You Reach Your Weight Goal**. This may be done before or after the group meeting.]*

Group Sharing Time (allow at least 5 minutes)

Let's take a few minutes to see how things went since our last meeting.

Were you able to apply what you learned at the last lesson to something in your life? If yes, how?

[Problem solve with the participants to address any barriers.]

[Praise all efforts to self-monitor and to change eating and activity behaviors. Be positive and nonjudgmental. Encourage group discussion

Manage Your Stress (pages 1-3)

[Review page 1 and include the following:]

- Stress is part of everyone's life but the personal experience of stress varies from person to person.
- Positive events (births, weddings etc.) may cause stress.
- The "inverted U-shaped curve" (also referred to as the Yerkes-Dodson law) is sometimes used as a visual for showing that there is an "optimal" range for stress, arousal, and behavior for each individual. Some amount of stress and arousal is good and provides excitement in life, but too much stress and arousal can be uncomfortable and harmful to your health and well-being.

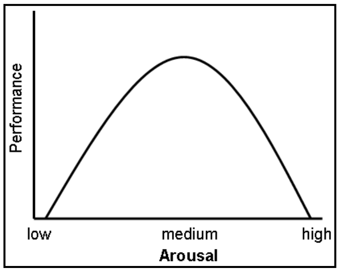

Stress comes in many forms and may be related to:
[Ask the group members to think about different categories of stress (physical, environmental, social and emotional) and their personal experiences with some of these.]

Think about your stress experiences, particularly as it relates to your efforts at healthy eating and physical activity. **Take a minute and answer** the questions on the bottom of the page. *[Ask participants to share their answers, if comfortable doing so.]*

[Review and discuss why too much stress can make it harder to reach and keep a healthy body weight or stay physically active (page 2).]

[Review and discuss the multiple ways to prevent or reduce stress listed on pages 2 and 3.]

Copyright 2017 University of Pittsburgh

When stress cannot be avoided, the task becomes one of problem solving and planning for the future so that stress can be circumvented and/or better managed. "Taking charge of stress" is a learned behavior similar to the other "taking charge" behavior change lessons you have been working on. *[Review* ***When you can't prevent stress, try to manage it better*** *(page 3). Ask the group to share their 10-minute time out strategies. Encourage discussion of other ideas for healthy ways to respond to stress.]*

Let's practice a specific relaxation technique called "belly breathing". *[This practice may be done while sitting in chairs. You can set the stage by dimming lights and guiding the group through the exercise for approximately five minutes.]*

Manage Sleep to Manage Stress (page 4)

[Ask group members to share how they feel when they have just the right amount of sleep compared to too little, or too much sleep.]

[Review basic sleep research facts (www.cdc.gov/features/sleep) and the basic premise that sufficient sleep is increasingly being recognized as an essential aspect of chronic disease prevention and health promotion. Encourage participants to track their sleep for a week.]

Tips for Getting Better Sleep (page 5)

[Review and discuss the factors that can have a positive or negative impact on personal sleep hygiene.]

Laughter is Good Medicine (page 6)

[Review and discuss the benefits of humor and laughter for a healthy lifestyle and for blood glucose control.]

To Do (page 7)

Turn to page 7 and let's focus on what you can do between now and the next session.

[Review and discuss. Encourage participants to take what they learned in today's group meeting and apply it. See if it helps them in their lifestyle change efforts.]

[Explain the 7-Day Sitting Diary on pages 8-9. Ask participants to bring the competed form to the next group meeting.]

Copyright 2017 University of Pittsburgh

[Announce the day, time, and place for the next session.]

After the session:

- Weigh participants who did not do so prior to the group meeting.
- Complete data forms and documentation required in your setting.
- Follow your program's protocol for managing absences.
- Follow your program's protocol for reviewing and commenting on the various self-monitoring records.
- Review the self-monitoring records. Write brief comments. Be positive and nonjudgmental.
    - Praise all efforts to self-monitor and to change eating and activity behaviors.
    - Highlight any positive changes made that relate to the topic of the last session before the records were collected.
    - Refer to **Guidelines for Reviewing Food and Activity Records** available in the GLB Providers Portal.
- If collecting **Resistance Training Logs,** review them**.** Write brief comments. Be positive and encouraging.

Copyright 2017 University of Pittsburgh

Session 17: Sit Less for Your Health

You know how important it is to reach and maintain your healthy lifestyle goal of at least 150 minutes of physical activity per week. However, there is something else that you can do to increase your movement and improve your health.

> The amount of physical activity you do in a day is important.
> How much <u>time you spend sitting</u> also matters.

To live a healthy lifestyle, public health research suggests that people need to both:

- maintain adequate levels of physical activity and
- limit the amount of time spent sitting.

It is possible to reach your 150 minutes of moderate intensity activity goal each week, yet still sit too much. This is often referred to as being an "active couch potato".

Two people could have the same amount of moderate intensity activity (such as brisk walking) but differ on the amount of sitting and light movement they do. Let's look at an example about Sadie and Sam.

- Sadie is a computer analyst.
    - o At work: She spends most of her day sitting.
    - o At home: She enjoys spending time on the computer playing games, connecting with friends, and searching the internet.
- Sam is an elementary school teacher.
    - o At work: He spends most of his day on his feet in front of his class and rarely sits.
    - o At home: He enjoys spending time cooking, gardening, and doing little projects around the house.

Copyright 2017 University of Pittsburgh

The graphs below show how much time Sam and Sadie spend in an average day doing moderate activity, light movement, and sitting.

- Moderate activity (like brisk walking): Sam and Sadie spend about the same amount of time doing their moderate intensity activities.
- Light movement: Sam spends much more time doing light movement during the day.
- Sitting: Sadie spends much more time sitting during the day.

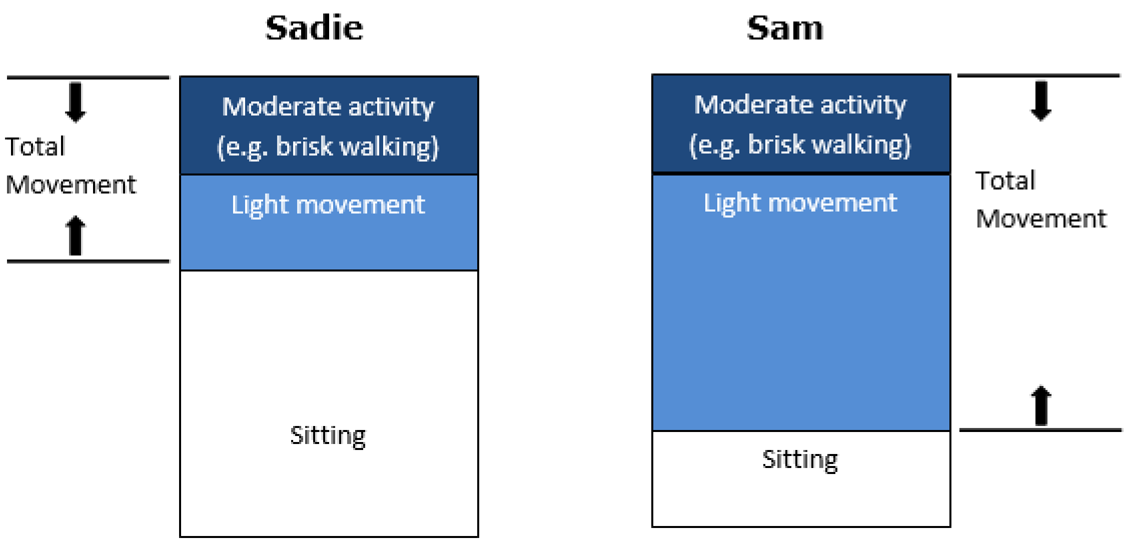

- Total Movement: Although Sam and Sadie have the same amount of moderate activity, there is a big difference in their amount of daily total movement. Sam is getting much more per day than Sadie.

Copyright 2017 University of Pittsburgh

Sitting is Becoming the New Normal

Progress over time has led to less and less movement in any given day. In general, people now perform most of their day-to-day tasks with little physical effort.

- The good news is that you tend to get more done in a day.
- The bad news is that the need to move is being removed, little by little, from your daily routine.

On average, adults spend more than half of their walking hours sitting. It is amazing how easily sitting time can add up:

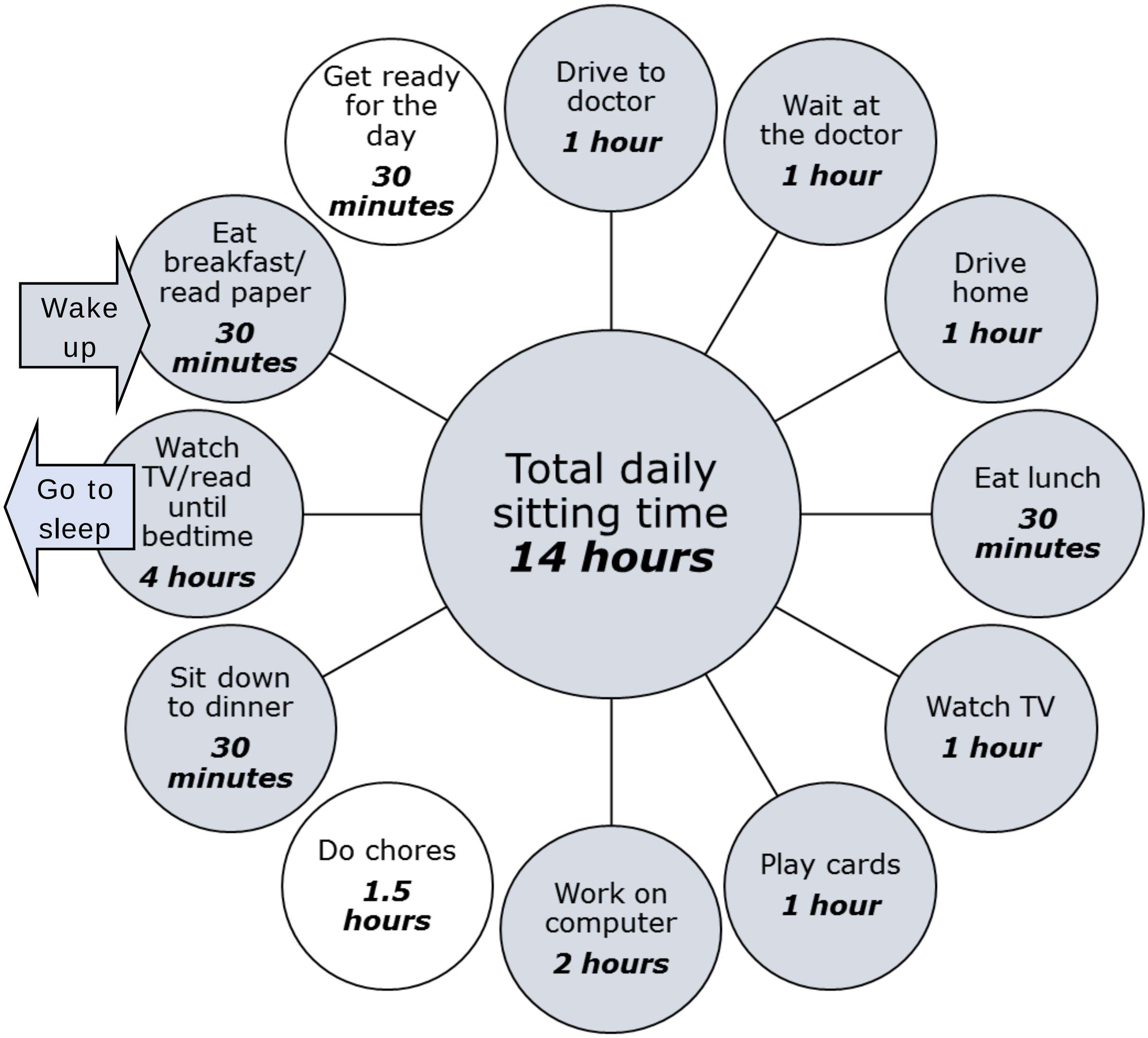

Copyright 2017 University of Pittsburgh

The Problem with Too Much Sitting

Research is finding that long bouts of time spent sitting can be harmful to your health. Let's look at a few studies:

- In the DPP, the risk of developing diabetes in overweight adults with pre-diabetes increased an average of 3.4% for each hour spent watching TV per day.
- Pima Indian adults who watched an average of 3 or more hours per day of TV weighed more than those who watched less.
- An Australian study showed that more than 2 hours of TV viewing per day was associated with an increased risk of death from heart disease and all other causes.
- Another Australian study found adults who broke up long bouts of sitting time by getting up and moving around had healthier blood cholesterol and glucose levels than those who do not.
- A review of a large number of research studies found that higher amounts of sitting were related to a higher risk of:
    - diabetes
    - heart disease
    - some types of cancers
    - early death

How much sitting is too much?

So far, not enough is known to set a healthy limit on the total number of hours in a day you spend sitting. In general, it is safe to say that most people would benefit from sitting less.

Here are some tips from experts:

- Limit screen-based sitting time (e.g. TV or computer) outside of work to less than 2-3 hours per day.
- Break up long continuous bouts of sitting with periods of movement (standing up, stretching, walking around).

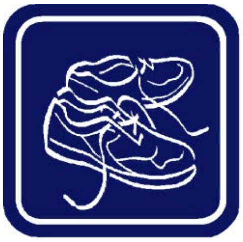

Sitting Less

Look at your 7-day Sitting Diary from the last session.

- What are your thoughts about the amount of time you sit?

  ____

- When did you tend to sit for a long period?

  ____

To **get started "sitting less" think about:**

- Where can you break up long periods of sitting with a brief movement break (standing up, stretching, or walking around)?
- Where can you replace a bout of sitting with a physical activity that lasts about 10 minutes or more?

You can find ways to "**sit less"** in many areas of your daily life. Think about a typical day. How much time do you spend sitting in each of these four major areas?

- Home
- Work
- Transportation
- Out and about (social or on your own)

Target blocks of time when you can sit less. What can you see yourself doing to make these changes?

	Reduce total sitting during the day	Break up long periods of sitting
Home		
Work		
Transportation		
Out and About		

Copyright 2017 University of Pittsburgh

Here are some more ideas. Check the ones you would like to try.

At Home
☐ Get up and move around the house during TV commercials
☐ Read the mail/newspaper while standing at the kitchen counter
☐ Do household chores or exercise while watching television
☐ Fold laundry while standing
☐ Stand or pace while on the phone
**At Work**
☐ Get up and take a break from your computer every 30 minutes
☐ Take breaks from sitting in long meetings
☐ Stand to meet with a visitor at your work space
☐ Stand during phone calls
☐ Drink more water – going to the water cooler and bathroom will break up sitting time
**Transportation**
☐ Park your car further away from stores, the shopping mall, or work
☐ Stand up and move around during long airplane flights
☐ Offer your seat to a person who really needs it when riding the bus
☐ Stop and take regular breaks from sitting during long car trips
☐ Avoid using the car for short trips – walk or bike instead
**Out and About**
☐ Stand instead of sitting to have coffee
☐ Suggest an active option when meeting a friend for a visit
☐ Take breaks from sitting when playing a game
☐ Get up after dinner instead of sitting at the table

Copyright 2017 University of Pittsburgh

Ways to Achieve the "Sit Less" Goal

In addition to doing your planned physical activity, you are now encouraged to "sit less". The first step is to be aware of your sitting habits throughout the day. Then find ways to reduce your sitting time.

To help you "sit less", remember these key ideas:

> Replace sitting activities with non-sitting activities
>
> Decrease daily screen time (TV, computer, tablets, smart phones)
>
> Avoid long periods of sitting. Break up your sitting time as often as possible.

There are <u>two</u> ways to help you **"**sit less**"**:

1. TABS = Take A Break from Sitting

- TABS is when you break up your sitting time by standing up. In other words, a "sit-to-stand" moment. This can be done in many ways.
- Each time you break up sitting by standing up counts as a TABS.

Once you have been successful at adding TABS into your day, you can build on this by adding Super TABS.

2. Super TABS

- This is a TABS that lasts 10 minutes or more. It is a longer block of time in which you replace sitting with standing or moving.
- Can be anything from standing still to taking a brisk walk.
- Helps you achieve even more health benefits.

Double Bonus: If your Super TABS involve physical activity at least as intense as a brisk walk, it can be added toward your daily activity goal.

Copyright 2017 University of Pittsburgh

The **GLB "Sit Less" Goal**

The GLB daily **"sit less" goal** is to do at least:

- 5 TABS (quick break) and
- 1 Super TABS (longer break of 10 minutes or more)

Get started:

- Take at least 3 short breaks from sitting (TABS) per day.
- Think of your typical day. List 3 times when you could break up your sitting time and do a TABS.

  1. ____
  2. ____
  3. ____

- Record using the "Sit Less" Tracker. Research suggests that self-monitoring a behavior will help you be more successful.

Keep going:

- Add more TABS to reach the goal of 5 TABS per day.
- Add 1 Super TABS per day.
- Work slowly and steadily toward this goal over the next month.

Week 1	Complete 3 TABS each day
Week 2	Complete 5 TABS each day
Week 3	Complete 5 TABS and 1 Super TABS each day

Once you reach this goal, you may choose to add additional TABS and Super TABS to your day.

A final thought…**"Failing to plan is planning to fail."**
- *Chinese Proverb*

Copyright 2017 University of Pittsburgh

To Do:

Check the boxes when you complete each item:

The Basics:

☐ Keep track of your weight.

☐ Record what you eat and drink using your preferred method. Come as close as you can to your calorie and fat gram goals.

☐ Record your physical activity using your preferred method. Come as close as you can to your activity goals.

- Be physically active for at least 150 minutes per week.
- Aim for an average of at least 50,000 steps per week.
- Include resistance training at least 2 times per week.

☐ Keep moving throughout the day.

What's New:

☐ Complete 3 TABS each day next week.

☐ Keep going by following the chart on page 8. Complete your TABS and Super TABS each week over the next month.

☐ Keep track of TABS and Super TABS by using the "**Sit Less**" Tracker (pages 10-11).

References and further reading for those who are interested:

Fitzgerald, S. J., Kriska, A. M., Pereira, M. A., & De Courten, M. P. (1997). Associations among physical activity, television watching, and obesity in adult Pima Indians. *Medicine and science in sports and exercise*, *29*(7), 910-915.

Rockette-Wagner, B., Edelstein, S., Venditti, E.M., Reddy, D., Bray, G.A., Carrion-Petersen, M.L., Dabelea, D., Delahanty, L.M., Florez, H., Franks, P.W., Montez, M.G., Rubin, R., & Kriska, A.M. (2015). The impact of lifestyle intervention on sedentary time in individuals at high risk of diabetes. *Diabetologia*, *58*(6), pp.1198-1202.

Healy, G. N., Dunstan, D. W., Salmon, J., Cerin, E., Shaw, J. E., Zimmet, P. Z., & Owen, N. (2008). Breaks in sedentary time beneficial associations with metabolic risk. *Diabetes care*, *31*(4), 661-666.

Dunstan, D. W., Barr, E. L. M., Healy, G. N., Salmon, J., Shaw, J. E., Balkau, B., & Owen, N. (2010). Television viewing time and mortality the Australian diabetes, obesity and lifestyle study (AusDiab). *Circulation*,*121*(3), 384-391.

Wilmot, E.G., Edwardson, C.L., Achana, F.A., Davies, M.J., Gorely, T., Gray, L.J., Khunti, K., Yates, T. and Biddle, S.J. (2012). Sedentary time in adults and the association with diabetes, cardiovascular disease and death: systematic review and meta-analysis. *Diabetologia*, *55*, pp.2895-2905.

Copyright 2017 University of Pittsburgh

Group Lifestyle Balance™ – "Sit Less" Tracker

Name: ___________________________

Day	TABS – short breaks from sitting. Goal = 5 or more per day.	Super TABS – Replace 10 minutes or more of sitting. Goal = 1 per day
Sun	☐☐☐☐☐☐☐☐☐☐	10 10 10
Mon	☐☐☐☐☐☐☐☐☐☐	10 10 10
Tue	☐☐☐☐☐☐☐☐☐☐	10 10 10
Wed	☐☐☐☐☐☐☐☐☐☐	10 10 10
Thu	☐☐☐☐☐☐☐☐☐☐	10 10 10
Fri	☐☐☐☐☐☐☐☐☐☐	10 10 10
Sat	☐☐☐☐☐☐☐☐☐☐	10 10 10
Sun	☐☐☐☐☐☐☐☐☐☐	10 10 10
Mon	☐☐☐☐☐☐☐☐☐☐	10 10 10
Tue	☐☐☐☐☐☐☐☐☐☐	10 10 10
Wed	☐☐☐☐☐☐☐☐☐☐	10 10 10
Thu	☐☐☐☐☐☐☐☐☐☐	10 10 10
Fri	☐☐☐☐☐☐☐☐☐☐	10 10 10
Sat	☐☐☐☐☐☐☐☐☐☐	10 10 10

Copyright 2017 University of Pittsburgh

Group Lifestyle Balance™ – "Sit Less" Tracker

Name: ____________________________

Day	TABS – short breaks from sitting. Goal = 5 or more per day.	Super TABS – Replace 10 minutes or more of sitting. Goal = 1 per day
Sun	☐☐☐☐☐☐☐☐☐☐	10 10 10
Mon	☐☐☐☐☐☐☐☐☐☐	10 10 10
Tue	☐☐☐☐☐☐☐☐☐☐	10 10 10
Wed	☐☐☐☐☐☐☐☐☐☐	10 10 10
Thu	☐☐☐☐☐☐☐☐☐☐	10 10 10
Fri	☐☐☐☐☐☐☐☐☐☐	10 10 10
Sat	☐☐☐☐☐☐☐☐☐☐	10 10 10
Sun	☐☐☐☐☐☐☐☐☐☐	10 10 10
Mon	☐☐☐☐☐☐☐☐☐☐	10 10 10
Tue	☐☐☐☐☐☐☐☐☐☐	10 10 10
Wed	☐☐☐☐☐☐☐☐☐☐	10 10 10
Thu	☐☐☐☐☐☐☐☐☐☐	10 10 10
Fri	☐☐☐☐☐☐☐☐☐☐	10 10 10
Sat	☐☐☐☐☐☐☐☐☐☐	10 10 10

Copyright 2017 University of Pittsburgh

Session 17 Leader Guide:
Sit Less for Your Health

Objectives
In this session, the participants will:
- Learn why it is important to be aware of their sitting time.
- Discuss why it is important to limit the amount of time spent sitting.
- Discuss suggestions for reducing the amount of time spend sitting.
- Describe how much time they currently spend sitting.
- Determine ways they will decrease and/or interrupt sitting in a typical day.
- Review and discuss ways to achieve the "sit less" goal by using TABS and Super TABS.
- Learn how to slowly and safely add TABS and Super TABS to their day.
- Make a plan to break up sitting time and complete TABS and Super TABS.

To Do Before the Session:
- If providing Food and Activity records, have a supply available for participants.
- Prepare Session 17 handout for participant notebooks.
- Review Food and Activity records; add comments.
- If collecting Resistance Training Logs; add comments.
- Print copies of the handout, **When You Reach Your Weight Goal** to review with participants, if needed.
- If providing **Resistance Training Logs**; have a supply available for participants.

Available in the GLB Providers Portal:
https://www.diabetesprevention.pitt.edu/my/login.aspx
- **Guidelines for Reviewing Food and Activity Records**
- **GLB Resistance Training Log**
- **When You Reach Your Weight Goal Handout**

*[**Note:** For those participants who have reached their goal weight, review with them the handout entitled **When You Reach Your Weight Goal**. This may be done before or after the group meeting.]*

Group Sharing Time (allow at least 5 minutes)

Let's take a few minutes to see how things went since our last meeting.

Were you able to apply what you learned at the last lesson to something in your life? If yes, how?

[Problem solve with the participants to address any barriers.]

[Praise all efforts to self-monitor and to change eating and activity behaviors. Be positive and nonjudgmental. Encourage group discussion.]

Sit Less for Your Health (pages 1-2)

So far in GLB you have learned about

- planned activity and the importance of reaching and maintaining at least 150 minutes of moderately intense activity per week
- spontaneous activity and ways to add more movement to your day
- how to use a pedometer to be aware of your total movement during the day
- the importance of resistance training and the health benefits of adding 2-3 days per week of resistance exercises to your physical activity plan

Today we're going to talk about another way to increase your overall movement. This topic has more recently received a lot of attention from the world of public health, specifically, the amount of time we spend sitting.
[Review pages 1 and 2 include the following:]

Read the examples of Sam and Sadie. Notice that they have the same amount of moderate intensity activity but differ in the amount of light movement and sitting that they each do. Sam has more light movement and Sadie spends more time sitting.

Would you say that your typical day is more like Sam or Sadie's day?

Sitting is Becoming the New Normal (page 3)

Do you agree with the comment that the amount of time each day that most people spend sitting has increased over time?

[Review page 3 and include the following:]

Review the graphic. Does it seem to you to be a pretty typical day?
Notice how easily you can end up spending most of your day sitting.

Look at the graph again. When and how could this person add some movement so there is less sitting time in a day?

Copyright 2017 University of Pittsburgh

The Problem with Too Much Sitting (page 4)

Current research evidence suggests that it is important to both reduce the amount of time you spend sitting as well as to break up longer periods of sitting.
[Review page 4 and include the following:]

The Problems with Too Much Sitting: Are you surprised by the relationship between TV watching and health? Total daily sitting and health?

How Much Sitting is Too Much?
Although there is not currently a current solid public health recommendation for daily time spent sitting, it is very well known that you would benefit from sitting less. It is suggested that you limit your screen time to 2-3 hours per day and break up long bouts of sitting.

Can you see yourself limiting your screen time to less than 2-3 hours per day? How could you make this happen?

Let's talk a little more about breaking up long bouts of sitting. What are you typically doing when you have long bouts of sitting? What could you do to break it up?

Sitting Less (pages 5-6)

Please take out your 7 Day Sitting Diary from the last session. Let's talk about the two questions listed at the top of page 5.

[Review each question. Encourage group discussion.]

Let's talk about the different areas of your life and which ones you tend to sit more in.

[Review the grid in page 5. Encourage group discussion about ways to reduce total sitting during the day and ways to break up long periods of sitting within each of the four major areas. Discuss additional ideas on page 6.]

Ways to Achieve the "Sit Less" Goal (pages 7)

GLB has emphasized the importance of first being aware of your behavior, then making a plan for change, and then applying what you learned in the group meeting and following your plan. You have done this with your eating and activity behaviors. Now it's time to follow this same pattern with your sitting behaviors.

[Review page 7.]

Now let's talk about how you can "Sit Less" by doing TABS and Super TABS.

Copyright 2017 University of Pittsburgh

[Discuss TABS and Super TABS clearly describing the difference between the two. Make sure that particiants understand that these TABS and Super TABS are changes that would now be made to their normal day. Already existing breaks from sitting should not be counted as a TABS or Super TABS. Discuss how you can fit a double bonus into your day.]

The GLB "Sit Less" Goal (page 8)

[Review page 8.]

GLB has set the sit less goal to 5 TABS and 1 Super TABS each day.

[Discuss the initial goal for getting started and the weekly goals increases.]

[Explain the ***Sit Less Tracker*** *located on pages 10-11.]*

To Do (page 9)

[Review and discuss. Encourage participants to take what they learned in today's group meeting and apply it. See if it helps them in their lifestyle change efforts.]

[Encourage participants to record their TABS and Super TABS in their ***Sit Less Tracker****. They will turn it in at the next session for the coach to review.]*

[Announce the day, time, and place for the next session.]

After the session:

- Weigh participants who did not do so prior to the group meeting.
- Complete data forms and documentation required in your setting.
- Follow your program's protocol for managing absences.
- Follow your program's protocol for reviewing and commenting on the various self-monitoring records.
- Review the self-monitoring records. Write brief comments. Be positive and nonjudgmental.
    - Praise all efforts to self-monitor and to change eating and activity behaviors.
    - Highlight any positive changes made that relate to the topic of the last session.
    - Refer to **Guidelines for Reviewing Food and Activity Records** available in the GLB Providers Portal.
- If collecting **Resistance Training Logs,** review them**.** Write brief comments. Be positive and encouraging.

Copyright 2017 University of Pittsburgh

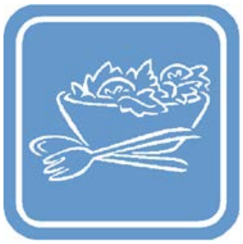

Session 18: More Volume, Fewer Calories

We have discussed many important nutrition topics to help you build new skills and follow a healthy eating pattern.

You learned from using Nutrition Facts labels, calorie and fat counters, menus, menu boards, and displays that all foods and drinks provide a certain number of calories per serving. Now you can learn some new ways to think about where your calories are coming from.

When you make food choices, think about:

The difference in calories for the same amount (volume) of food.

For example:

- o 1 cup of raw spinach = 7 calories
- o 1 cup of premium ice cream = 500 calories

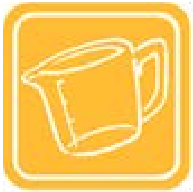

It's the same amount of food, but a big difference in calories.

The amount (volume) of food you get for the calories you spend.

For example:

- o 1 slice of apple pie = 360 calories

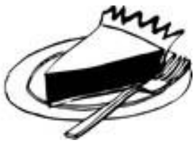

- o 6 apples = 360 calories

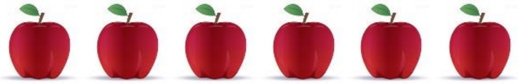

The same calories, but a big difference in the amount (volume) of food.

Copyright 2017 University of Pittsburgh

Adding volume to your meals and snacks

You can learn ways to feel fuller on fewer calories by adding more volume to your meals.

This approach is called "Volumetrics" (Rolls, B.J. (2012). The Ultimate Volumetrics Diet. William Morrow, New York, New York).

The main idea in **"Volumetrics" is to add volume to your meals**. You do this by eating foods that are:

- Lower in fat
- Higher in fiber (such as beans, split peas, lentils, fruits, vegetables)
- Higher in water content (such as broth-based soups, fruits, vegetables)
  Note: Just drinking more water has not been shown to have the same effect.

Eating lower fat foods that are higher in fiber and water content will:

- Encourage a plant-based diet
- Provide many healthful nutrients
- Follow the recommendations of MyPlate and the 2015 Dietary Guidelines

This "Volumetrics" way of eating is helpful in weight management because it allows you to:

- Eat a greater amount or volume of food
- Consume fewer calories overall
- Enjoy a satisfying portion of food
- Feel fuller on fewer calories

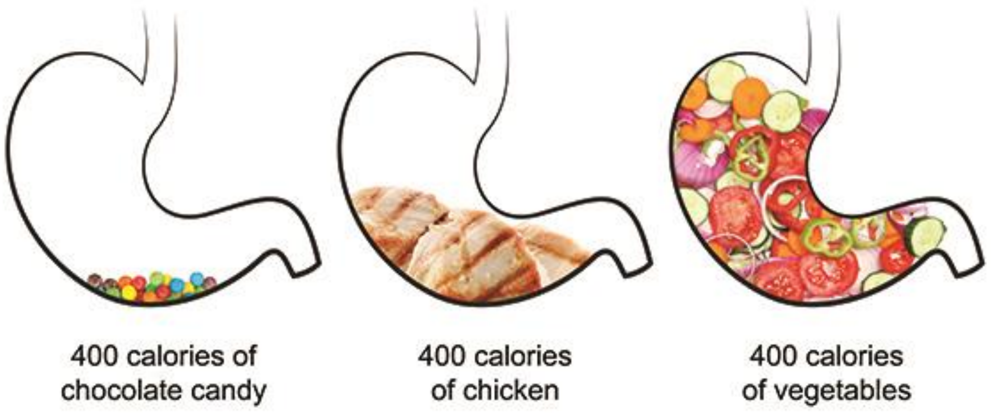

Copyright 2017 University of Pittsburgh

How to Increase Volume in Your Meals

1. Eat vegetables

- Start your meals with a salad, raw vegetables, vegetable juice, or a clear broth-based vegetable soup.
- Experiment with new vegetables. Try some "new" kinds of greens in your salad. Try grilled or oven roasted vegetables.
- For a snack, choose raw vegetables with hummus or a low-fat dressing.
- Have fresh, frozen or canned vegetables on hand to add to soups, casseroles, chili, and pasta or rice dishes. (Add spinach, diced carrots, and extra onions to chili. Add broccoli to baked ziti. Add plenty of vegetables to jars of tomato sauce).
- To reduce sodium, use fresh, frozen, or canned vegetables without added salt.

2. Eat fruit

- Start your meal with fruit.
- Add fruit to your favorite yogurt, cereal, cooked rice, and tossed salad.
- Keep frozen fruit in your freezer; blend with yogurt for a great smoothie.
- Choose whole fruits, fruits canned in juice, and frozen fruits without added sugar.
- Replace part of the oil in baked goods with unsweetened applesauce.
- Fruit is a sweet way to end your meal.

Copyright 2017 University of Pittsburgh

3. Eat broth-based soups (about 100 calories per cup)

- Make your own soup. Add plenty of vegetables.
- Remove fat from the top of chilled leftover soup before reheating and eating.
- To reduce sodium, use reduced sodium broth. Flavor with herbs and spices rather than salt.
- Add extra vegetables or beans to prepared soups.

Note: "Volumetrics" research studies report that starting a meal with a serving of fruit, vegetable, or 1 cup of broth-based soup helps people eat less at the meal.

4. Eat beans, split peas, and lentils

- Add beans (black, kidney, chickpea, soybean) or lentils to soups, stews, chili, salads, pasta, rice, casseroles, and pasta sauces.
- Enjoy split pea soup, lentil soup, and chili with kidney beans.
- Use hummus as a dip or sandwich spread.
- To reduce sodium, use dried beans that have been soaked and cooked or canned beans that have been rinsed and drained.

5. Eat whole grains rather than ones that are highly refined

MyPlate recommends that at least half of the grains eaten each day should be whole grains, such as:

- High fiber ready-to-eat breakfast cereal.
- Cooked oatmeal or whole grain cereal.
- 100% whole wheat bread, buns, bagels, pita, English muffins, tortillas, and crackers.
- Brown rice, barley, bulgur, whole wheat couscous, farro, quinoa.
- Popcorn that is low-fat or air popped.

Note: It is important to *substitute* whole grain products for refined grain products, rather than *adding* whole grain products, in order to stay within your calorie goals.

Copyright 2017 University of Pittsburgh

6. Eat small amounts of nuts

Nuts are a good source of fiber but also high in calories and fat.
Use a small amount:

- Added to cooked cereal.
- On tossed salads in place of cheese or croutons.
- Added to yogurt.
- Sprinkled on cooked vegetables.

Research studies have shown that people tend to eat about the same amount or volume of food each day. It is the amount of food in your stomach that determines how full you feel.

If you "volumize" your meals, you can eat more food. This will leave you feeling fuller and more satisfied.

If you try to rely on just eating less, you may be left feeling hungry and deprived.

By increasing the volume of food, you will consume fewer calories, enjoy a satisfying portion of food, and keep hunger in check.

Copyright 2017 University of Pittsburgh

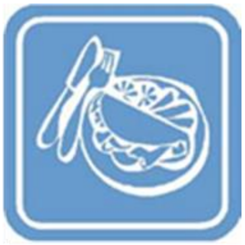

Satiety

Satiety means how full you are feeling. The more satisfied you feel, the easier it will be to stay close to your calorie goals and manage your weight.

Adding volume is just one way to feel more satisfied after a meal. Here are some other ways:

Eat foods high in protein at each meal.
- Protein may help you feel fuller longer.
- Choose lean protein foods from MyPlate's Protein and Dairy Groups. Examples include lean meats, chicken, seafood, eggs, low-fat dairy products, and beans, or small servings of nuts or peanut butter.

Eat high fiber foods instead of simple carbs.
- Simple carbs (sugar, sweets, highly refined grains) are digested quickly so you may feel hungry again very soon after eating.
- High fiber foods digest more slowly so you feel fuller longer.
- Foods with 3 grams of fiber/serving are a good source of fiber; those with 5 grams or more are an excellent source.

Stay within your fat gram goal **(but don't go too low)**.
- Fats delay stomach emptying so you feel fuller longer.
- Eating too little fat during the day decreases satiety so increases feelings of hunger.

Divide calories throughout the day.
- Eating at regular intervals throughout the day increases feelings of satiety. It is usually best to eat every 3-4 waking hours.
- Experiment with timing of your meals and snacks to learn what helps you stay the most energized and satisfied.

Drink water.
- Stay well-hydrated. Dehydration increases hunger.
- Drink water and other calorie-free beverages.
- Limit sugar-sweetened beverages that contain few nutrients. Liquid calories are less satisfying than those from food.

Eat slowly and mindfully. Make meals last about 20 minutes.
- Enjoy every bite so you feel both physically and mentally satisfied.

Copyright 2017 University of Pittsburgh

Practice:
Help Sam Feel Less Hungry

Sam reports feeling hungry during the day.

What changes could he make to his meal and snacks to help him feel fuller longer?

**Sam's Menu**	Ways to feel less hungry
Breakfast	
Plain bagel with jelly	
Orange Juice	
Black Coffee	
Lunch	
Tossed Salad with	
Fat-free dressing	
2 sugar cookies	
Sweetened Iced Tea	
Snack	
Candy bar	
Dinner	
Spaghetti with tomato sauce	
Garlic bread	
Wine	
Snack	
Pretzels	

Copyright 2017 University of Pittsburgh

To Do:

Check the boxes when you complete each item:

The Basics:

☐ Keep track of your weight.

☐ Record what you eat and drink using your preferred method. Come as close as you can to your calorie and fat gram goals.

☐ Record your physical activity using your preferred method. Come as close as you can to your activity goals.

- Be physically active for at least 150 minutes per week.
- Aim for an average of at least 50,000 steps per week.
- Include resistance training at least 2 times per week.

☐ Sit less. Be aware of long periods of sitting and break them up. Keep moving throughout the day.

What's New:

☐ Practice adding volume to your meals (see pages 3-5).

☐ Experiment with other ways to feel more satisfied after a meal (see page 6).

Did you feel fuller and more satisfied at the end of the meal?

____

Did you notice any difference in your calorie intake?

____

Copyright 2017 University of Pittsburgh

Session 18: Resources — Page

Roasted Vegetables 10

Light and Fresh Vegetable Soup 11

Copyright 2017 University of Pittsburgh

Roasted Vegetables

Classic Roasted Vegetables

(adapted from Weight Watchers® New Complete Cookbook)

2 medium red bell peppers, seeded and cut into 8 wedges
2 medium zucchini, trimmed and cut into ¼ inch slices
1 medium Vidalia or other sweet onion, cut into 8 wedges
1 pound asparagus, trimmed
3 cloves garlic, minced
4 teaspoons olive oil
Salt and pepper to taste
(optional: ½ teaspoon dried basil)

Directions:

- Preheat oven to 450° F.
- Spray 2 baking sheets or oblong pans with cooking spray.
- In a large bowl, place all vegetables, garlic, oil, salt and pepper (and basil, optional). Toss well to evenly coat with seasonings.
- Divide the vegetables between the 2 baking sheets or oblong pans.
- Bake, tossing occasionally, until tender and lightly browned, 25-30 minutes.
- Serve immediately.

Note: Leftover vegetables plus hummus make a great sandwich!

Note: This recipe works well with just broccoli and/or cauliflower and red onion.

Servings = 4 servings of 1 cup each
One serving (1 cup) = 107 calories; 5 fat grams
Source: Weight Watchers® New Complete Cookbook (Wiley Publishing, Inc., 2008)

Copyright 2017 University of Pittsburgh

Vegetable Soup

Light and Fresh Vegetable Soup

2 teaspoons olive oil
4 cloves garlic, minced
5 cups low-fat or fat-free chicken broth
2 sprigs thyme
½ cup frozen green peas, thawed
1 cup thinly sliced red bell pepper
1 cup cut fresh asparagus--(1 ½ -inch long pieces)
1¾ cups sliced Swiss chard or spinach
1½ cups cooked penne pasta

Directions:

- Heat olive oil in large saucepan over medium heat. Add garlic and sauté 1 minute.
- Add the broth and thyme, and bring to a boil. Cover, reduce heat, and simmer 10 minutes.
- Add Swiss chard, bell pepper, and peas; cover and simmer 5 minutes.
- Add the asparagus, cover and simmer 2-4 minutes or until the asparagus is just tender.
- Stir in the pasta, and cook 1 minute.

Servings = 4 servings of 2 cups each
One serving (2 cups) = 150 calories, 3 grams fat
Source: 'Volumetrics: Feel Full on Fewer Calories' by Barbara Rolls, PhD. (HarperCollins Publishers, 2000)

Copyright 2017 University of Pittsburgh

Session 18 Leader Guide:
More Volume, Fewer Calories

Objectives
In this session, the participants will:
- Learn the four principle ways of adding volume to meals.
- Learn how adding volume to meals is helpful in weight management.
- Discuss ways for adding volume to meals.
- Review recipes that add volume to meals.
- Define satiety and discuss how it impacts weight management.
- Discuss ways to increase satiety.
- Complete an exercise where they brainstorm ways to increase satiety.

To Do Before the Session:
- If providing Food and Activity books, have a supply available for participants.
- Prepare Session 18 handout for participant notebooks.
- Review Food and Activity records; add comments.
- If collecting **Resistance Training Logs**; add comments.
- Print copies of the handout, **When You Reach Your Weight Goal** to review with participants, as they achieve their weight goals.
- Print copies of the **Group Lifestyle Balance Monthly Calendar**; have a supply available for participants.
- Given the array of self-monitoring tools provided, determine your protocol for review and feedback to the participants. Note: As session frequency decreases, the number of records returned at each session may increase.
- If providing **Resistance Training Logs;** have a supply available for participants.
- If providing **Sit Less Trackers**; have a supply available for participants.
- Optional: Prepare one of the recipes from the handout for participants to taste-test during class.

<u>Available in the DPP-GLB Providers Portal:</u>
https://www.diabetesprevention.pitt.edu/my/login.aspx
- **GLB Resistance Training Log**
- **When You Reach Your Weight Goal Handout**
- **Group Lifestyle Balance Monthly Calendar**
- **Sit Less Tracker**
- **Guidelines for Reviewing Food and Activity Records**

*[**Note:** For those participants who have reached their goal weight, review with them the handout entitled **When You Reach Your Weight Goal**. This may be done before or after the group meeting.]*

Copyright 2017 University of Pittsburgh

Group Sharing Time (allow at least 5 minutes)

Let's take a few minutes to see how things went since our last meeting.

Were you able to apply what you learned at the last lesson to something in your life? If yes, how?

[Problem solve with the participants to address any barriers.]

[Praise all efforts to self-monitor and to change eating and activity behaviors. Be positive and nonjudgmental. Encourage group discussion.]

More Volume, Fewer Calories (pages 1-2)

[Review information on page 1.]

[Review information on page 2 and include the following:]

Have any of you heard of a book entitled Volumetrics ("Volumetrics" (Rolls, B.J. (2012). The Ultimate Volumetrics Diet. William Morrow, New York, New York) or read about it in a magazine?

The basic idea is that by increasing the volume of food, you will consume fewer calories, enjoy a satisfying portion of food, and keep hunger in check.

[Note that just drinking more water has not been shown to have the same effect on fullness as eating foods high in water content. This is because water is emptied rapidly from your stomach/GI tract. But drinking water is still a healthy addition to your day.]

The picture at the bottom of page 2 clearly shows how what you eat can have an impact on how full you feel after eating about 400 calories.

- You could eat less than ½ cup of chocolate candy, about 1 ¾ cups of roasted chicken breast without the skin, or a little over 18 cups of mixed raw salad vegetables.
- Can you see how adding volume to your meals can help you feel fuller?

Let's look at some suggestions for how to add volume to your meals.

How to Increase Volume in Your Meals (pages 3-5)

Copyright 2017 University of Pittsburgh

[Review each category. Ask participants to share additional suggestions or ideas. Include the following information:]

1. **Eat vegetables.** Enjoy raw or cooked vegetables. Has anyone tried roasting vegetables? What did you think? Page 10 of your handout has a recipe for roasted vegetables. If you try this recipe, you can give your feedback at our next group meeting.

2. **Eat fruit** – Not only do fruits and vegetables provide many healthful nutrients, they add fiber and water to help you feel more satisfied after a meal.

3. **Eat broth-based soups (about 100 calories per cup) -** Soup takes a long time to eat, fills your stomach, and takes time to empty from your stomach, so you feel fuller longer. It is also a good way to add water, fiber, and vegetables to your healthy eating pattern.

   Read the Nutrition Facts label for the sodium content in prepared soups. There are many reduced sodium soups available. One advantage of making homemade soups is being able to control the amount of salt.

   Prepared soups can be enhanced by adding extra fresh, frozen, canned or leftover vegetables or legumes. Any suggestions? (Spinach to chicken rice soup, legumes to vegetable soup, etc.)

   Soups could be enjoyed as an appetizer or as a main dish. Page 11 has a soup recipe from one of the Volumetrics books. I encourage you to try it (or any broth-based soup), and to be aware of your level of fullness after the meal. If you try this recipe, you can give your feedback at the next meeting.

   Notice that all of the suggestions for ways to add volume to your meals are in line with the recommendations of MyPlate and the Dietary Guidelines.

[Optional: Taste test the food you prepared using one of the recipes in the session handout.]

Satiety (page 6)

As we discussed, adding volume to your meals can help you feel fuller. Another word for feeling full is “satiety”. Satiety is not necessarily dependent on the number of calories eaten in a meal. In addition to adding volume to your meals, here are some other ways to help you feel satisfied with fewer calories.

[Review page 6 and include the following:]

Eat high fiber foods instead of simple carbohydrates

Copyright 2017 University of Pittsburgh

- Adding fiber to your meals will add volume to your meals and help you feel fuller.
- There are also many health benefits to eating a high fiber diet. Can you name some of the health benefits?
    - Reduced risk of heart disease and some types of cancer
    - Can help lower blood cholesterol levels and control blood glucose levels
    - Keeps your intestinal tract healthy. Reduces the risk of constipation, hemorrhoids, and diverticular disease
    - Helps with weight management. Fiber absorbs water in your intestinal tract so you feel full more quickly and for a longer period of time.
- One of the easiest ways to increase the fiber in your diet is by eating a high fiber breakfast cereal. Read the Nutrition Facts label. The main reason for eating cereal is for the fiber.
- How much fiber should you eat? There are different recommendations:
    - The Dietary Guidelines tell us to choose foods rich in dietary fiber.
    - The Daily value on the Nutrition Facts label is 25 grams per day, based on a 2000 calorie/day diet.
    - The FDA recommends about 14 grams of fiber per 1000 calories eaten. So for each of the GLB calorie goals, the daily fiber goal would be:
        - 1200 calories = 17 grams of fiber
        - 1500 calories = 21 grams of fiber
        - 1800 calories = 25 grams of fiber
        - 2000 calories = 28 grams of fiber
- Remember to drink plenty of fluid as you increase the fiber in your diet.

Drink water. Remember that the goal is to have light yellow urine. If urine is very dark in color, this can be a sign of dehydration.

Practice: Help Sam Feel Less Hungry (page 7)

Let's practice applying what you learned about adding volume and other ways to increase satiety.

[Work as a group to brainstorm suggestions for Sam. Emphasize that there are many suggestions for each meal and snack. Examples:]
- *Breakfast – whole grain bagel, add protein, fruit instead of juice*
- *Lunch – add protein to salad, can use low-fat dressing, whole grain roll or crackers, calorie-free beverage*
- *Snack – replace with yogurt and fruit or apple with peanut butter*
- *Dinner – add protein to sauce, add salad and fruit*
- *Snack – substitute low-fat popcorn (this is a whole grain) or carrots with hummus*

Copyright 2017 University of Pittsburgh

To Do (page 8)

How can you add volume to your meals and apply other ways to increase satiety?

Turn to page 8 and let's focus on what you can do between now and the next session.

[Review this page. Encourage participants to practice adding volume to their meals and to experiment with other ways to increase satiety.]

[Announce the day, time, and place for the next session.]

After the session:

- Weigh participants who did not do so prior to the group meeting.
- Complete data forms and documentation required in your setting.
- Follow your program's protocol for managing absences.
- Follow your program's protocol for reviewing and commenting on the various self-monitoring records.
- Review the self-monitoring records. Write brief comments. Be positive and nonjudgmental.
    - Praise all efforts to self-monitor and to change eating and activity behaviors.
    - Highlight any positive changes made that relate to the topic of the last session before the records were collected.
    - Refer to **Guidelines for Reviewing Food and Activity Records** available in the GLB Providers Portal.
- If collecting **Resistance Training Logs** and/or **Sit Less Trackers,** review them**.** Write brief comments. Be positive and encouraging.

Copyright 2017 University of Pittsburgh

Session 19: Stay Active

To prevent or delay diabetes and improve health, keep an eye on your physical activity goals. Aim for a well-rounded plan that blends moderate intensity activities (like brisk walking) with strength training, sitting less and moving more.

Today, you will:

- revisit the "sit-less" activity goal and plan for a "sit-less" week
- give yourself credit for your efforts to stay active
- learn how good posture supports your physically active lifestyle
- remind yourself <u>why</u> you want to stay active
- discuss your biggest barriers and ways to cope with them

Sit less review

Let's look back at the past week. Refer to your Sit Less Tracker if you have one.

- Please mark the times each day with a "T" when you were able to add at least one TABS (you took a short break from sitting).
- Next, mark the times each day with an "ST" when you added at least one Super TABS (you replaced your sitting with standing or moving for 10 minutes or more).

	Mon	Tue	Wed	Thu	Fri	Sat	Sun
Morning							
Afternoon							
Evening							

Copyright 2017 University of Pittsburgh

Look at the grid above. Is there a specific day or time of day when you are getting more of your TABS or Super TABS?

____

____

When and where could you add more TABS or Super TABS? Be specific.

____

____

GLB has encouraged you to try to sit less in several different areas of your life. Are there specific changes you have made in any of these areas?

- ☐ Home ____
- ☐ Work ____
- ☐ Transportation_______________________________________
- ☐ Out and about (socially, on your own) _____________________

____

When you reflect on <u>all</u> your efforts to sit less, what is working best for you? What are some of the challenges?

____

____

Give yourself credit for any "double bonuses" (swapping sitting activities with moving more). What sit less/move more changes are you most proud of?

____

____

Good work. Keep going. Keep moving.

Copyright 2017 University of Pittsburgh

Plan for a "Sit Less" Week

"A goal without a plan is just a wish"
-Antoine de Saint-Exupery, French writer

You can find the time to sit less.

- Find blocks of time where you can add more TABS (brief breaks from sitting) or Super TABS (replacing sitting for at least 10-minutes at a time)
- Make your plan in the table below. Be specific.

	What I Will Do	When	Where
Mon			
Tues			
Wed			
Thurs			
Fri			
Sat			
Sun			

Don't just wait for the sitting breaks to happen.

You need to make them happen. Have a clear plan for when and where you will add TABS and Super TABS in your week.

What are some things that could make it hard to carry out this plan?

____

To overcome these challenges, I will:

____

Remember to give yourself credit. Record your TABS and Super TABS so you can see your progress.

Copyright 2017 University of Pittsburgh

Good Posture Supports Your Physically Active Lifestyle

The term *posture* describes how your body is positioned when you sit, stand, or move. Let's look at why proper posture is so important as you move forward with your physically active lifestyle.

Good Posture

- Supports healthy physical movement and may prevent injury.
- Helps your body function more efficiently.
- Projects poise, confidence, and dignity.
- Improves appearance.

Bad Posture

- Can cause some muscles to work harder than others. This creates a muscle imbalance.
- Because some muscles are working harder than they should, you may feel fatigued.
- Slouching bends the back, can limit lung movement, and hinder breathing.
- May cause muscles or joints to become stiff and sore.

Your goal is to stay active over the long term. Healthy posture will help you stay more active.

Strong, flexible muscles are the key to good posture.

- Strong abdominal (core/belly) muscles support your back.
- Weak or tight hip or leg muscles can pull on your back. Example: "tight hamstrings" (the muscles on the back of your thigh) can lead to poor posture and back pain.

Healthy, functioning joints are the key to fluid movement.

- Hip, knee, and ankle joints support your back's natural curves when you are moving.
- Allow you to have good posture for standing and moving.

Copyright 2017 University of Pittsburgh

Good Posture, Good Movement

When you set out to do any physical activity (spontaneous or planned), take a moment to check your standing posture.

Keep these images in mind:

Front View

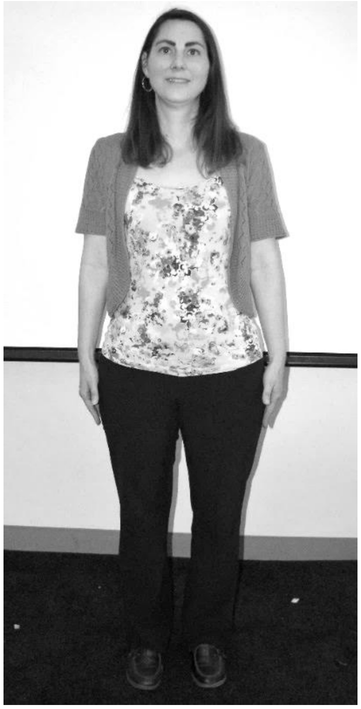

Side View

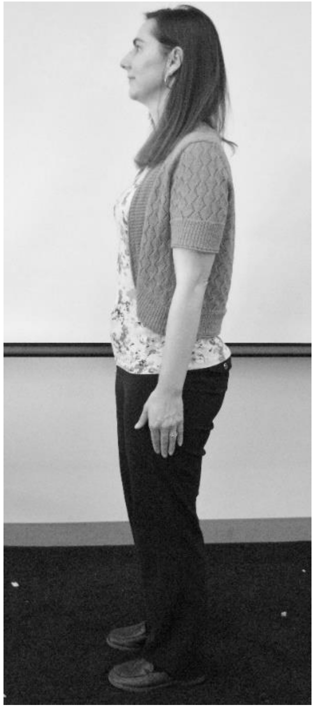

Front View

- Imagine a straight line from the top of your head, through the center of your body, to your feet. This is good alignment.
- Head should be held straight with chin level. Do not tilt your head to one side.
- Both shoulders, hips, and knees should be of equal height, relative to each other.

Side View

- Imagine a straight line through your ear, shoulder, hip, knee and ankle.
- Maintain the three natural curves of your back (neck, upper back, and lower back).

Copyright 2017 University of Pittsburgh

Good Sitting Posture

Good sitting posture may reduce strain on your muscles, tendons, and bones.

- Head is held erect (no slouching or leaning forward).
- Maintain the three natural curves of the back (neck, upper back, and lower back).

Guidelines for Proper Sitting Position at Your Desk

- Use a chair with good back support and position yourself close to your desk/keyboard.
- Keep your feet flat on the floor. If they do not reach the floor, use a footrest.
- Knees and hips should be bent at a 90-degree angle.
- Keep your wrists in line with your arms – not angled up or down.
- Keep your head and neck straight and facing forward. Computer monitor should be at eye level.
- Keep your elbows at your side with your forearms parallel to the floor.

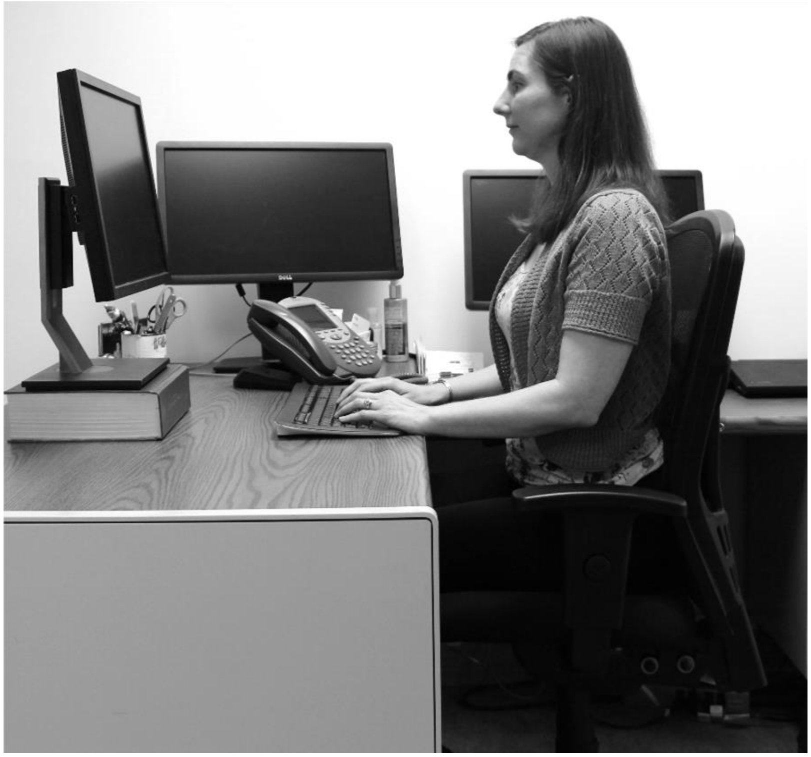

Copyright 2017 University of Pittsburgh

Take a Break from Sitting

Despite your best efforts, some days you have to sit at a desk for a long time. This prolonged sitting can cause stiffness and pain, and be hazardous to your muscles and joints.

Here are some quick and easy tips to help ease the strain on your muscles and joints.

- Set a timer that cues you to stand up or move each 30 minutes.
- Shift positions in your chair often.
- Make small adjustments to your chair.
- Stand up, march in place, stretch out your arms.
- Walk up and down your hallway for 100 steps.
- Try a standing desk or a keyboard that moves up and down.
- Try some of the desk stretches below.

Desk Stretches

1. Hand – Open and Fist

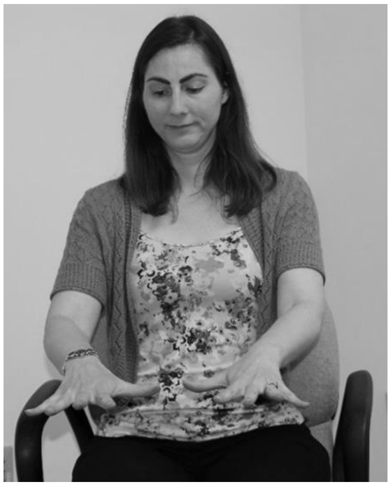

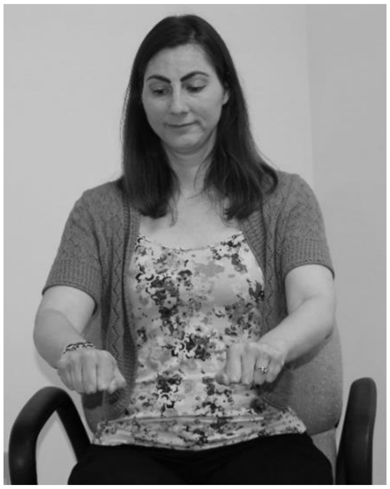

- Separate and straighten your fingers until you feel a stretch.
- Hold 10 seconds.
- Make a fist – squeeze tight.
- Hold 10 seconds.

Copyright 2017 University of Pittsburgh

2. Neck – Head Tilt

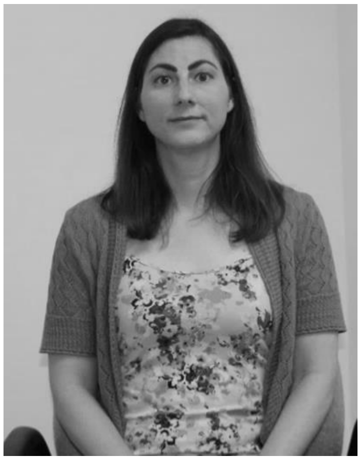

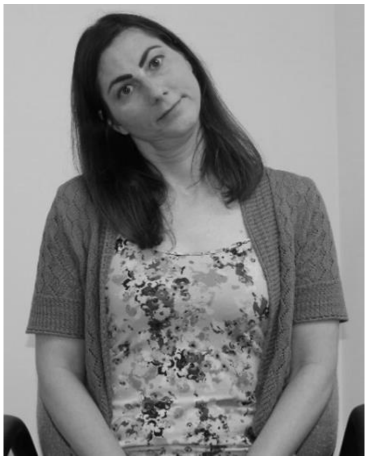

- Keep shoulders relaxed with arms hanging loosely at your sides.
- Look straight ahead, tilt your head to one side (ear towards your shoulder).
- Keep shoulders relaxed and do not raise your shoulders.
- Hold for 5-10 seconds.
- Relax – bring head to starting position.
- Repeat on the other side.

3. Shoulder Shrug

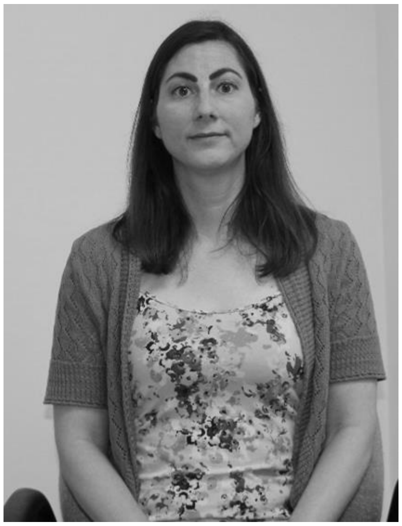

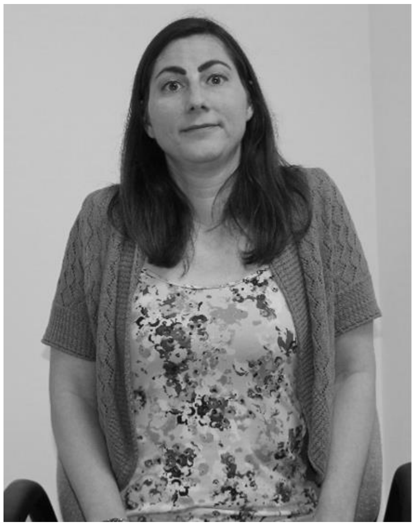

- Raise your shoulders up to your ears until you feel slight tension in your neck and shoulders.
- Hold for 5-10 seconds.
- Relax and let your shoulders drop into their resting position.
- Repeat 3-5 times.

Copyright 2017 University of Pittsburgh

4. Arms, Shoulders and Upper Back

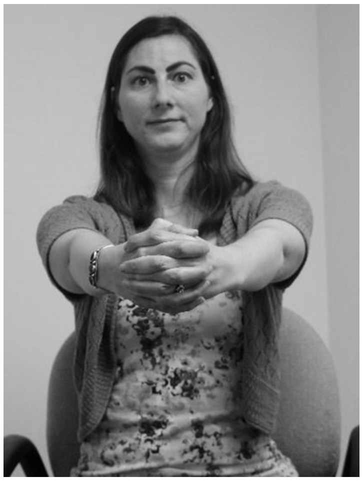

- Interlace your fingers.
- Straighten your arms out in front of you. (You can also turn palms outward).
- Feel the stretch in your arms and the upper part of your back.
- Hold for 10-20 seconds, repeat 2-3 times.

5. Arms, Shoulders, and Sides of Trunk

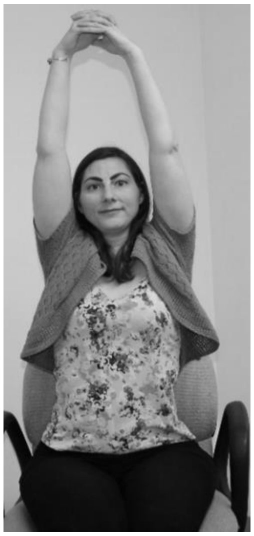

- Interlace your fingers. Straighten your arms overhead. (You can also turn palms outward).
- Think of elongating your arms.
- Feel the stretch in your arms and upper sides of your rib cage.
- Hold for 10-20 seconds, repeat 2-3 times.

6. Upper back

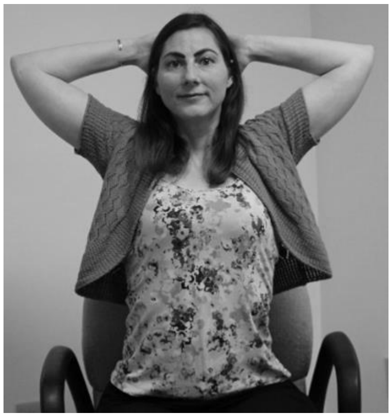

- Interlace your fingers behind your head with elbows out to the side.
- Pull shoulder blades back toward each other.
- Hold for 5-10 seconds.
- Repeat 2-3 times.

Copyright 2017 University of Pittsburgh

Why Do I Want to Stay Active?

Reasons to stay active

Let's pause for a moment to focus on why you choose to stay active.

The top three reasons I stay active (even when it feels hard):

1. ____
2. ____
3. ____

Barriers and ways to cope with them

Most people face daily challenges that move their activity plans to the back burner. Things that get in the way of your plans to exercise are called exercise barriers.

People who stay active find ways to overcome these barriers.

What keeps you from exercising?

My biggest activity barriers/challenges	Not-so-healthy responses	Healthier responses

Social support

Having social support has been shown to help people overcome barriers to physical activity.

Who supports your efforts to stay active? _______________________

What do they say or do to support you? _______________________

How can you get more of the support you need? _________________

Copyright 2017 University of Pittsburgh

To Do:

Check the boxes when you complete each item:

The Basics:

- ☐ Keep track of your weight.
- ☐ Record what you eat and drink using your preferred method. Come as close as you can to your calorie and fat gram goals.
- ☐ Record your physical activity using your preferred method. Come as close as you can to your activity goals.
  - Be physically active for at least 150 minutes per week.
  - Aim for an average of at least 50,000 steps per week.
  - Include resistance training at least 2 times per week.
- ☐ Sit less. Be aware of long periods of sitting and break them up. Keep moving throughout the day.

What's New:

- ☐ Break up your sitting with TABS and Super TABS.
- ☐ Use the "Sit Less" Tracker (pages 14-15) and give yourself credit for TABS and Super TABS.
- ☐ Maintain good posture as part of your physically active lifestyle.
- ☐ Try quick tips to ease your muscles and joints when you get stuck sitting for long periods. Practice desk stretches (pages 7-9).
- ☐ Remind yourself often of the top three reasons you choose to stay active.
- ☐ Be aware of your barriers to physical activity and ways to cope with them.
- ☐ Find the support you need to stay active.

Copyright 2017 University of Pittsburgh

Session 19: Resources

Session 19: Resources	Page
Healthy Back Posture Helps You Stay Active	13
Group Lifestyle Balance™ - "Sit Less" Tracker	14-15

Copyright 2017 University of Pittsburgh

Healthy Back Posture Helps You Stay Active

A healthy back has three natural curves in balanced alignment.

- Cervical curve – slight forward curve of the neck
- Thoracic curve – a slight backward curve of the upper back
- Lumbar curve – a slight forward curve of the low back

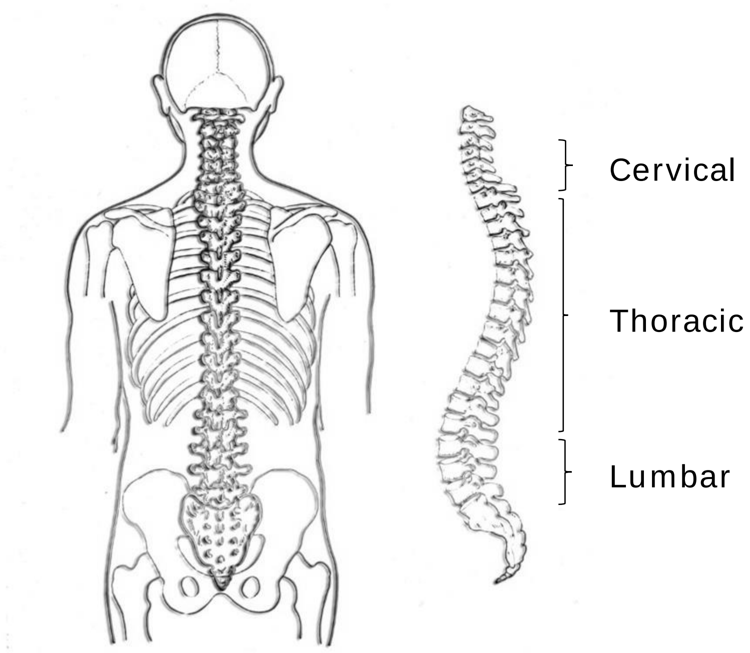

Good posture supports healthy physical movement and may prevent injury.

Copyright 2017 University of Pittsburgh

Group Lifestyle Balance™ – "Sit Less" Tracker

Name: ___________________________

Day	TABS – short breaks from sitting. Goal = 5 or more per day.	Super TABS – Replace 10 minutes or more of sitting. Goal = 1 per day
Sun	□□□□□□□□□□	10 10 10
Mon	□□□□□□□□□□	10 10 10
Tue	□□□□□□□□□□	10 10 10
Wed	□□□□□□□□□□	10 10 10
Thu	□□□□□□□□□□	10 10 10
Fri	□□□□□□□□□□	10 10 10
Sat	□□□□□□□□□□	10 10 10
Sun	□□□□□□□□□□	10 10 10
Mon	□□□□□□□□□□	10 10 10
Tue	□□□□□□□□□□	10 10 10
Wed	□□□□□□□□□□	10 10 10
Thu	□□□□□□□□□□	10 10 10
Fri	□□□□□□□□□□	10 10 10
Sat	□□□□□□□□□□	10 10 10

Copyright 2017 University of Pittsburgh

Group Lifestyle Balance™ – “Sit Less” Tracker

Name: ___________________________

Day	TABS – short breaks from sitting. Goal = 5 or more per day.	Super TABS – Replace 10 minutes or more of sitting. Goal = 1 per day
Sun	☐☐☐☐☐☐☐☐☐☐	10 10 10
Mon	☐☐☐☐☐☐☐☐☐☐	10 10 10
Tue	☐☐☐☐☐☐☐☐☐☐	10 10 10
Wed	☐☐☐☐☐☐☐☐☐☐	10 10 10
Thu	☐☐☐☐☐☐☐☐☐☐	10 10 10
Fri	☐☐☐☐☐☐☐☐☐☐	10 10 10
Sat	☐☐☐☐☐☐☐☐☐☐	10 10 10
Sun	☐☐☐☐☐☐☐☐☐☐	10 10 10
Mon	☐☐☐☐☐☐☐☐☐☐	10 10 10
Tue	☐☐☐☐☐☐☐☐☐☐	10 10 10
Wed	☐☐☐☐☐☐☐☐☐☐	10 10 10
Thu	☐☐☐☐☐☐☐☐☐☐	10 10 10
Fri	☐☐☐☐☐☐☐☐☐☐	10 10 10
Sat	☐☐☐☐☐☐☐☐☐☐	10 10 10

Copyright 2017 University of Pittsburgh

Session 19 Leader Guide:
Stay Active

Objectives

In this session, the participants will:

- Review ways to achieve the "sit less" goal by doing TABS and Super TABS.
- Be aware of their efforts to safely add TABS and Super TABS to their day.
- Make a plan to break up sitting time and complete TABS and Super TABS.
- Learn the benefits of good posture and how it supports a physically active lifestyle.
- Learn the risks of bad posture and how it could impact on their ability to achieve and maintain a physically active lifestyle.
- Describe and practice good standing posture.
- Describe and practice good sitting posture.
- Discuss ways to take a break from sitting that will ease the strain on muscles and joints.
- Learn and practice six desk stretches.
- Identify personal reasons for wanting to stay active.
- Identify barriers to physical activity and ways to overcome them.
- Discuss the importance of having social support for physical activity.
- Identify those who support their efforts to stay active and discuss how to get more of the support they need.

To Do Before the Session:

- If providing Food and Activity records, have a supply available for participants.
- Review Food and Activity records; add comments.
- Prepare Session 19 handout for participant notebooks.
- If collecting **Resistance Training Logs** and/or **Sit Less Trackers**; add comments.
- Print copies of the handout, **When You Reach Your Weight Goal** to review with participants, as they achieve their weight goals.
- Print copies of the **Group Lifestyle Balance Monthly Calendar**; have a supply available for participants.
- If providing **Resistance Training Logs;** have a supply available for participants.
- Given the array of self-monitoring tools provided, determine your protocol for review and feedback to the participants. Note: As session frequency decreases, the number of records returned at each session may increase.

Available in the DPP-GLB Providers Portal:

https://www.diabetesprevention.pitt.edu/my/login.aspx

- **GLB Resistance Training Log**
- **When You Reach Your Weight Goal** handout
- **Group Lifestyle Balance Monthly Calendar**
- **Guidelines for Reviewing Food and Activity Records**

Copyright 2017 University of Pittsburgh

*[**Note:** For those participants who have reached their goal weight, review with them the handout entitled **When You Reach Your Weight Goal**. This may be done before or after the group meeting.]*

Group Sharing Time (allow at least 5 minutes)

Let's take a few minutes to see how things went since our last meeting.

Overall, how did it go? What went well? What problems did you have? What could you do differently?

Were you able to apply what you learned at the last lesson to something in your life? If yes, how?

[Problem solve with the participants to address any barriers.]

[Praise all efforts to self-monitor and to change eating and activity behaviors. Be positive and nonjudgmental. Encourage group discussion.]

Sit Less Review (pages 1-2)

[Note: This is the final session with an activity/movement focus.]

Throughout GLB you have been asked to be aware of your total movement. You were gradually introduced to the components of a well-rounded activity plan that includes moderate intensity physical activity, strength training, sitting less and moving more. You have been working on achieving both your moderate activity and your sit less goals as important parts of your physically active lifestyle.

First, let's revisit the "sit less" activity goal.

[Review "Today, you will:"]
Please take out your **Sit Less Tracker** handout from last week or from Session 17. If you don't have it with you, please think about this past week.

[Review the directions for completing the chart on page 1. Encourage group discussion about what times of the day/week they are getting most of their TABS and Super TABS.]

[Discuss strategies participants have been using to add TABS and Super TABS to their day. Be sure to explore specific changes they have made in each of the areas listed.]

[Be sure to devote time to discussing the challenges participants are having with trying to sit less.]

[Review the definition of a "double-bonus". Emphasize that while reducing sitting alone can be good for your health, swapping sitting with moderate (or greater) intensity physical activity has been shown to be even more beneficial.]

Copyright 2017 University of Pittsburgh

[Give an example of a double bonus: If you replace sitting time with a moderate intensity physical activity (like brisk walking) for at least 10 minutes, you would get credit for sitting less (a Super TAB) and it would also count toward your weekly physical activity goal.]

Plan for a "Sit Less" Week (page 3)

[Review page 3. Emphasize the importance of having a plan.]

[If time permits, ask participants to complete their plan for the upcoming week. If you do not have time in class, encourage them to complete the chart at home.]

Encourage participants to record TABS and Super TABS. One option is to use the ***Sit Less Tracker*** *found on pages 14-15.]*

Good Posture Supports Your Physically Active Lifestyle (pages 4-5)

Now let's talk about how good posture supports your physically active lifestyle and may prevent injury.

[Review the information on page 4 about good/bad posture and the role of healthy muscle and joints in maintaining good posture and fluid movement.]

How would you rate your posture: Consistently good or needs some work?

Good Posture, Good Movement (page 5)

A healthy back has three natural curves in balanced alignment. Please look at the pictures on page 5.

Let's practice good standing posture.

[Ask participants to stand. Review the information on page 5 and ask the participants to correct their standing posture accordingly.]

Do you feel differently when your posture is correct? Do you think you look better? More confident? Do you notice that it is easier to breathe?

How much more of an effort was it for you to stand with good posture?

Good posture is important to help you stay active.

- On one hand, good posture supports your efforts to have a physically active lifestyle. It encourages healthy movement and may prevent injury. This can lead to a positive cycle of moving more.

Copyright 2017 University of Pittsburgh

- On the other hand, bad posture may discourage your efforts to move forward with a physically active lifestyle. This is because bad posture makes movement more difficult, less safe, and can cause muscles or joints to become stiff or sore. This can lead to a negative cycle of sitting more and moving less.

Good Sitting Posture (page 6)

[Ask participants to sit. Review the information on page 6.]

Let's practice good sitting posture. *[Ask participants to correct their sitting posture based on the information and the picture on page 6.]*

Do you notice a difference in how you feel when your sitting posture is correct?

How much more of an effort was it for you to sit with good posture?

Take a Break from Sitting/Desk Stretches (pages 7-9)

[Review and discuss how to ***Take a Break from Sitting****. Encourage discussion.]*

[Review the six different stretches. As you explain and demonstrate each stretch, ask participants to try it.]

Can you see yourself doing these desk stretches at home and/or at work? When are other times that you could do these stretches? [*Examples include on a plane, in your car when stuck in traffic, while watching a movie at home.]*

Why Do I Want to Stay Active? (page 10)

We have discussed all the different components of a well-rounded activity plan: moderately intense activity of at least 150 minutes per week, strength training 2-3 times per week, sitting less and moving more.

Reasons to stay active
Why do you stay active? *[Ask participants to jot down their answers on page 10. Ask participants to share their responses, if comfortable doing do.]*

Barriers and ways to cope with them
[Review and discuss the information in this section. You may want to give some examples of very busy people who still find time to be active.]
[Ask participants to complete the chart. Allow a few minutes for them to jot down their answers and then discuss.]

What are your biggest barriers/challenges to being active?

Copyright 2017 University of Pittsburgh

[After the group discussion, point out that the most common barriers to exercise that were reported by the group. Note: Typical barriers are lack of time, weather, feeling tired, and lack of motivation.]

What are some not-so-healthy responses? *[Examples: not doing any activity, spending more time sitting, eating more or making unhealthy food and beverage choices]*

What are some healthier responses; ways you have found to overcome these barriers? *[Examples:*

- *Lack of time: walk in short bouts of 10-15 minutes, multi-task by walking in the treadmill while watching the evening news*
- *Weather: walk at the mall; use an exercise program on DVD, TV or computer; walk early in the day during the summer*
- *Feeling tired: be active earlier in the day; walk in short bouts*
- *Lack of motivation: find an exercise buddy; join an exercise class you enjoy; say to yourself that you can walk for 10-15 minutes and stop if you want]*

Social Support
[Review the information and encourage group discussion.]

To Do (page 11)

Turn to page 11 and let's focus on what you can do between now and the next session.

[Review and discuss. Encourage participants to take what they learned in today's group meeting and apply it. See if it helps them in their lifestyle change efforts.]

[Announce the day, time, and place for the next session.]

After the session:

- Weigh participants who did not do so prior to the group meeting.
- Complete data forms and documentation required in your setting.
- Follow your program's protocol for managing absences.
- Follow your program's protocol for reviewing and commenting on the various self-monitoring records.
- Review the self-monitoring records. Write brief comments. Be positive and nonjudgmental.
    - Praise all efforts to self-monitor and to change eating and activity behaviors.
    - Highlight any positive changes made that relate to the topic of the last session before the records were collected.
    - Refer to **Guidelines for Reviewing Food and Activity Records** available in the GLB Providers Portal.
- If collecting **Resistance Training Logs** and/or **Sit Less Trackers,** review them**.** Write brief comments. Be positive and encouraging.

Copyright 2017 University of Pittsburgh

Session 20:
Balance Your Thoughts

Changing your old lifestyle habits is hard. However, you have already learned that it is possible. In addition, many of you comment on all the positive things that have come from simply learning about yourself as you change eating and activity behaviors.

Let's take some time to reflect on what you have learned about yourself so far in GLB.

Eating behaviors

- I am most proud of these changes in my eating behavior:

  ____

- Here are the positive things I have noticed:

  ____

Physical activity behaviors

- I am most proud of these changes in my activity behavior:

  ____

- Here are the positive things I have noticed:

  ____

What impact has making these healthy lifestyle changes had on your life, family, friends or community?

____

____

Copyright 2017 University of Pittsburgh

My Reasons for Behavior Change

What are the top 5 reasons for making healthy changes that are most important to you right now.

Why I want to keep working on my healthy lifestyle behavior changes	
I want to look in the mirror and feel OK.	
I want to look better to others.	
I want to be able to wear a smaller size.	
I want to be able to shop for clothes with ease.	
I want to stop dwelling on how others view me.	
I want to hear compliments on my appearance.	
I want to prevent physical illness and disease.	
I want to be comfortably active.	
I want to live longer.	
I want to have more energy.	
I want to be fit.	
I want to wake up in the morning and feel healthier.	
I want to like myself more.	
I want to feel more in charge of my life.	
I want to feel as if I've accomplished something important.	
I want to feel self-confident.	
I want to stop saying negative things to myself.	
I want to feel happier in social situations.	
I want to do more and different kinds of activities.	
I want my family to be proud of me.	
I want to be able to be more assertive.	
I want to eat with others and feel comfortable.	
I want to stop being nagged about my weight.	
Other reasons:	

Copyright 2017 University of Pittsburgh

Do Your Thinking Patterns Help or Hurt Your Lifestyle Progress?

Earlier in GLB we asked you to "listen" to the kinds of things you say to yourself as you work on building healthier habits.

We hope some of these self-statements are positive. We want you to give yourself credit for small changes in the right direction.

What are some positive self-statements you make about your:

Eating behaviors

____

Activity behaviors

____

Weight

____

Overall, have you noticed any changes in your thinking about healthy eating, physical activity, and weight management?

____

____

Self-defeating thoughts are also a normal part of lifestyle change, but they get in the way of your lifestyle progress. Do you struggle with self-defeating thoughts? What are they?

____

____

Copyright 2017 University of Pittsburgh

Here are some common self-defeating thoughts with helpful ways you can respond to them and move toward lifestyle balance:

Self-Defeating Thoughts	Example	Helpful Response
All-or-nothing thinking	*Either I'm perfect on my eating and activity plan or I've completely failed.*	Making a mistake is definitely not the same as failure.
Jumping to conclusions	*Since I ate chocolate today, reaching my healthy eating goal is impossible.*	I don't have to be perfect. I make healthy food choices most of the time.
Negative fortune-telling	*I won't be able to resist the desserts at the party.*	No one will force me to eat. I need to prepare myself in advance.
Discounting the positive	*I only walked for 100 minutes this week. I deserve credit only if I reach my activity goal.*	I deserve credit for every positive action that I take.
Emotional reasoning	*Since I feel so hopeless about reaching my goals, it really must be hopeless.*	Everyone gets discouraged from time to time. My goals are unreachable <u>only</u> if I decide to give up.
Labeling	*I'm a failure for overeating or skipping my walk today.*	Of course I'm not a failure. I reach my goals most weeks.
Mythologizing	*If I eat standing up, it doesn't count.*	A calorie is a calorie, no matter how I consume it.
Excuses & Rationalizations	*It's okay to sit and stare at the TV all night because I'm so stressed out.*	I need to find healthier ways to cope with my problems.
Exaggeration	*I'm totally out of control with my eating and lack of activity.*	I may have strayed from my plan, but I can start following my program again right away.

Copyright 2017 University of Pittsburgh

Excuses and Rationalizations

Some thoughts give you permission to steer away from your healthy lifestyle goals and plans. These thoughts often have a kernel of truth to them at the time. They also give you permission to stray.

Have you heard yourself saying any of these things?

It's okay to stray because…

___ It's just one little piece.
___ It's not *that* fattening.
___ I'll just eat less later today.
___ It won't matter in the long run.
___ It'll go to waste if I don't eat it.
___ I'll disappoint someone if I don't eat it.
___ Everyone else is eating it too.
___ I worked out today.
___ I'm tired and deserve to rest.
___ No one will see me eating it.
___ It's free.
___ It's a special occasion.
___ I'm upset, and I just don't care.
___ I'm craving it.
___ I'll probably just eat it eventually.
___ I want to get my money's worth.
___ I'll exercise tomorrow.
___ People are depending on me right now.

Your own excuses:

____

____

A healthier response:

____

____

Copyright 2017 University of Pittsburgh

What are the specific situations in which you are most likely to give yourself permission to stray from your healthy eating or physical activity plans?

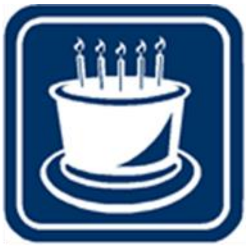

____

____

What is going through your mind?

____

____

Your goal is to keep working towards balance with a new, healthier self-statement. Examples:

"Even though I had a bad day and crave a sweet, I will eat an apple instead of this candy bar because I want to stay on track to reach my goals".

"Yes, I am tired but I will feel so much better after I take a walk".

When you give yourself permission to stray
you strengthen your old habits.

This makes its more likely that you will give in again in the future.

However....

When you respond in a healthier way,
you break your old negative cycle.

You also strengthen your new habit of self-control.

Do You Give Yourself Credit?

Do you tend to think mostly about the things you have done wrong with your lifestyle program rather than everything you did well?

Do you ever pat yourself on the back for the healthy lifestyle behaviors you did well? Focusing only on your mistakes, without paying attention to your successes, may cause you to feel weak or hopeless when you "slip".

To counter feeling discouraged or defeated, it is important to give yourself credit for everything you do right.

Give Yourself Credit:

Starting today, record 1 or 2 healthy behaviors you do *right* each day.

List your credit-worthy behaviors and write down positive words or phrases to give yourself a pat on the back.

For example:

I didn't take a second helping at the party. I'm in control.
I walked for 30 minutes today. I feel terrific.
I weighed myself today.

When you begin to give yourself credit every time you do something right, you get better at seeing that slips are just today's mistakes. They are not the end of your healthy lifestyle goals and plans.

By giving yourself credit, you will reinforce your self-confidence and begin to realize that you are strong and in control.

The more you practice healthy restraint, the easier it becomes.

To Do:

Check the boxes when you complete each item:

The Basics:

☐ Keep track of your weight.

☐ Record what you eat and drink using your preferred method. Come as close as you can to your calorie and fat gram goals.

☐ Record your physical activity using your preferred method. Come as close as you can to your activity goals.

- Be physically active for at least 150 minutes per week.
- Aim for an average of at least 50,000 steps per week.
- Include resistance training at least 2 times per week.

☐ Sit less. Be aware of long periods of sitting and break them up. Keep moving throughout the day Keep moving throughout the day.

What's New:

☐ Find the situations where you are most likely to give yourself permission to stray.

☐ Practice replacing self-defeating thoughts with positive ones.

☐ Give yourself credit. Write down 1 or 2 healthy lifestyle behaviors you do right each day.

Copyright 2017 University of Pittsburgh

Session 20 Leader Guide:
Balance Your Thoughts

Objectives
In this session, the participants will:

- Reflect on the long-term process of engaging in weight management behaviors and the impact that losing weight has on their life.
- Rank the importance of personal reasons for persisting at weight management efforts.
- Identify common types of self-defeating thoughts that interfere with lifestyle goal achievement.
- Practice countering characteristic negative thoughts with more helpful and effective responses.
- Practice countering excuses and rationalizations in order to strengthen a new habit of healthy restraint and self-control.
- Consider how to respond to "slips" as a normal, ongoing part of the weight management experience.
- Practice giving themselves credit for the positive behaviors they have been able to maintain.

To Do Before the Session:

- If providing Food and Activity books, have a supply available for participants.
- Prepare Session 20 handout for participant notebooks.
- Review Food and Activity records; add comments.
- If collecting **Resistance Training Logs** and/or **Sit Less Trackers**; add comments.
- Print copies of the handout, **When You Reach Your Weight Goal** to review with participants, as they achieve their weight goals.
- Print copies of the **Group Lifestyle Balance Monthly Calendar**; have a supply available for participants.
- If providing **Resistance Training Logs;** have a supply available for participants.
- If providing **Sit Less Trackers**; have a supply available for participants.
- Given the array of self-monitoring tools provided, determine your protocol for review and feedback to the participants. Note: As session frequency decreases, the number of records returned at each session may increase.
- Optional: Print and cut the **Thoughts** cards for an interactive group activity.

Copyright 2017 University of Pittsburgh

<u>Available in the DPP-GLB Providers Portal:</u>
https://www.diabetesprevention.pitt.edu/my/login.aspx

- **GLB Resistance Training Log**
- **When You Reach Your Weight Goal Handout**
- **Group Lifestyle Balance Monthly Calendar**
- **Sit Less Tracker**
- **Thoughts cards**
- **Guidelines for Reviewing Food and Activity Records**

*[**Note:** For those participants who have reached their goal weight, review with them the handout entitled **When You Reach Your Weight Goal**. This may be done before or after the group meeting.]*

Group Sharing Time (allow at least 5 minutes)

Let's take a few minutes to see how things went since our last meeting.

Were you able to apply what you learned at the last lesson to something in your life? If yes, how?

[Problem solve with the participants to address any barriers.]

[Praise all efforts to self-monitor and to change eating and activity behaviors. Be positive and nonjudgmental. Encourage group discussion.]

Balance Your Thoughts (page 1)

Let's start our meeting today by taking a few minutes to reflect on what you have learned about yourself so far in GLB.

Please take a few minutes and answer the questions on page 1. *[Ask participants to share their responses, if comfortable doing so. Encourage group discussion.]*

My Reasons for Behavior Change (page 2)

Think about the process of being involved in a weight management experience and/or the impact weight loss has had on your life.

Copyright 2017 University of Pittsburgh

Rank the top five personal reasons you want to continue to focus on losing weight (or maintain their weight loss) and improving health.

[Discuss (while recording group members comments on the board or a flip chart) the different reasons that can be identified for persisting at lifestyle weight management efforts over the long haul, even when it is hard.]

Notice that the reasons listed can be divided into four main categories: the first 6 relate to appearance; the next 6 to health; the next 5 to self-confidence; and the last 6 relate to social reasons. You may have other reasons. Please add them to the list.

Do Your Thinking Patterns Help or Hurt Your Lifestyle Progress? (pages 3-4)

In Session 9 we talked about how thoughts can help or hurt the lifestyle change process. What did you learn to do about self-defeating thoughts? *[Answer - Be aware of the negative thought, STOP, and change it to a more positive thought.]*

Effectively managing the thinking patterns that are part and parcel of one's lifestyle weight management experience is critical to long term success. There are several common categories of self-defeating thoughts that characterize the "mental game" of weight management and it is helpful for you to identify and label such thoughts. The goal is to become more skillful at countering negative self-defeating thoughts with positive self-statements.

[Review page 3. Encourage group discussion as participants answer the questions.]

Please turn to page 4 and let's talk about some of the common self-defeating thoughts.

[Review with the group each of the common categories of negative, self-defeating thoughts listed in their participant handout. Provide examples, and ask the group members to consider which of these types of "discouraging thought traps" they have found themselves falling into in the past. Encourage the group to help one another by coming up with alternative responses or counter-challenges that would be more helpful for long term weight loss efforts in the future.]

[Optional activity: Distribute one ***Thoughts*** *card to each participant. You may choose to just use the most meaningful cards for the group. Read each question below and encourage group discussion. If the group is responsive to this activity, distribute additional cards and keep going.]*

- This is an example of which of the nine categories of self-defeating thoughts listed on page 4?
- Has anyone every had this thought? Is this a common thought for you or rare? In what situation(s) does it occur?
- How did it impact your behavior?
- Did it keep you from reaching your goals?
- How would you change this to a more positive thought?

Copyright 2017 University of Pittsburgh

Excuses and Rationalizations (pages 5-6)

One type of self-defeating thinking pattern deserves further attention and discussion.

Making excuses and rationalizations for a slip from healthy eating or activity is a common type of thought pattern in which weight losers engage, particularly when they are experiencing weight regain. Because changing old habits can be difficult, there is a "kernel of truth" (or more) to such thoughts (e.g., it is hard to say no to a tempting treat when others around you are indulging, or you are saying to yourself that you will get back on track "tomorrow"). Nonetheless, the bottom line is that these types of self-defeating thoughts give the individual permission to stray from their healthy lifestyle behaviors and long-term goals.

Please look at the list of typical statements of why "It's okay to stray". *[Review and discuss. Ask participants to highlight their own excuses and identify healthier responses.]*

It is important that you be vigilant for these types of thoughts as you continue your efforts at weight management.

The more often an individual "gives in" to such thoughts the more he or she strengthens the habit of "giving in or giving up". The more often an individual practices "not giving in", the more likely it is he or she will strengthen the behavior of self-restraint and healthy self-control. Countering excuses and rationalizations requires repeated practice just like other elements of behavioral self-management during the weight loss process.

[Review and discuss the information on page 6. Encourage group discussion. Emphasize the "bottom line" messages in the two text boxes at the bottom on the page.]

Do You Give Yourself Credit? (page 7)

Ultimately, the most effective weight loser/maintainers are those who can maintain a positive long-term outlook regarding their behavior change efforts and build on "small wins".

It may be difficult for you to focus on what you are doing well, day after day, particularly in the face of slips. To prevent discouragement practice giving yourselves credit for everything you are managing to do well.

You talked about the many eating and activity behaviors that are going well and the positive things you have noticed.

Copyright 2017 University of Pittsburgh

Starting today, please record 1-2 healthy behaviors you do right each day. Feel free to jot them down in your Food and Activity record. The goal is for you to reinforce your self-confidence by building on each instance of personal success.

In GLB, we focus on progress; not perfection.

To Do (page 8)

Turn to page 8 and let's focus on what you can do between now and the next session.

[Review and discuss. Encourage participants to take what they learned in today's group meeting and apply it. See if it helps them in their lifestyle change efforts.]

[Announce the day, time, and place for the next session.]

After the session:

- Weigh participants who did not do so prior to the group meeting.
- Complete data forms and documentation required in your setting.
- Follow your program's protocol for managing absences.
- Follow your program's protocol for reviewing and commenting on the various self-monitoring records.
- Review the self-monitoring records. Write brief comments. Be positive and nonjudgmental.
    - Praise all efforts to self-monitor and to change eating and activity behaviors.
    - Highlight any positive changes made that relate to the topic of the last session before the records were collected.
    - Refer to **Guidelines for Reviewing Food and Activity Records** available in the GLB Providers Portal.
- If collecting **Resistance Training Logs** and/or **Sit Less Trackers,** review them**.** Write brief comments. Be positive and encouraging.

Copyright 2017 University of Pittsburgh

Session 21: Heart Health

Heart disease and stroke are the leading causes of death in the world for *both* men and women. People with pre-diabetes, diabetes, and/or the metabolic syndrome are at higher risk for developing heart disease.

What is heart disease?

- Heart disease, also called cardiovascular disease, is a group of diseases and conditions that affect the heart (cardio) and blood vessels (vascular).
- Examples of heart disease include heart attack and stroke.

What are the risk factors for heart disease? The American Heart Association has identified the following risk factors:

**Risk factors that can't be changed**	Risk factors that can be changed
Increasing age	High blood cholesterol
Gender (male)	High blood pressure
Family history	Physical inactivity
	Obesity and Overweight
	Smoking
	Diabetes
	Poor diet

Also linked with an increased risk of heart disease: stress, depression, poor sleep quality or sleep apnea, drinking more alcohol than recommended.

The more risk factors you have, the greater your risk of heart disease.

What are your risk factors? ________________________________

The good news is that being physically active, eating a healthy diet, and managing your weight can help reduce your risk.

A heart-healthy lifestyle is your best defense against heart disease.

Copyright 2017 University of Pittsburgh

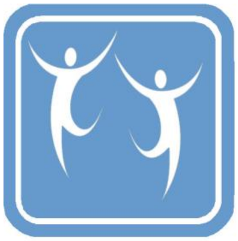

Preventing or Managing Heart Disease with **"Life's Simple 7™"**

The American Heart Association lists seven diet and lifestyle changes to improve heart health and enhance quality of life.

1. Get active

- The American Heart Association recommends
  - at least 150 minutes of moderate intensity activity each week AND
  - strength training on at least 2 days.
- Regular physical activity
  - improves risk factors such as blood pressure, cholesterol, and blood glucose levels.
  - strengthens your heart muscle, improves blood circulation, reduces feelings of stress, and helps control body weight.

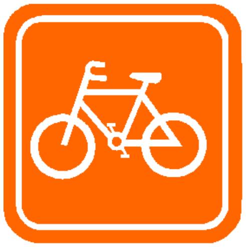

2. Control cholesterol (see pages 5-8 for more information)

- Adults ages 20 and older should have their cholesterol tested every 5 years.
- Healthy cholesterol levels help your arteries remain free of plaque which can clog arteries and lead to heart disease.

3. Eat better

- Eat a variety of fruits, vegetables, whole grains, fat-free or low-fat dairy products, skinless poultry, lean meat, fish, nuts, and legumes.
- Eat fish at least twice a week.
- Limit saturated fat, trans fat, sodium, added sugars, and alcohol.

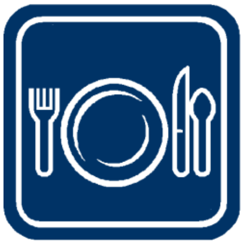

Copyright 2017 University of Pittsburgh

4. Manage blood pressure (see pages 6-7 for more information)

- High blood pressure is a major risk factor for heart disease. The higher the blood pressure; the greater the risk.
- Healthy blood pressure reduces strain on your heart, arteries, and kidneys.
- Aim for blood pressure that is less than 120/80 mm/Hg.
  - Pre-hypertension - 120-139/80-89 mm/Hg
  - High blood pressure/hypertension - 140/90 mm/Hg or higher

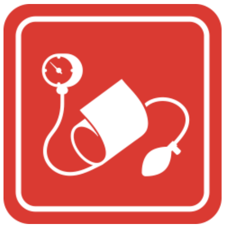

5. Lose weight and keep it off

- A healthy weight reduces the burden on your heart, blood vessels, lungs, and muscles, bones, and joints.
- When your weight is in a healthy range you are less likely to develop heart disease, high blood pressure, high cholesterol, and diabetes.

6. Reduce blood sugar (glucose)

- Healthy blood sugar (glucose) levels help prevent damage to your heart, kidneys, eyes, and nerves that can occur when levels are too high.
- Aim for a fasting blood glucose of under 100 mg/dl.
  - Pre-diabetes – 100-125 mg/dl
  - Diabetes – 126 mg/dl or higher

7. **Don't smoke** and avoid secondhand smoke

Even one lifestyle change can make a difference.

A combination of two or more lifestyle changes may achieve even better results.

Copyright 2017 University of Pittsburgh

Heart disease can happen at any age. So, do what you can to stay heart-healthy and delay or prevent heart disease.

Did you notice that "Life's Simple 7™" guidelines reflect what you have already learned in GLB?

Which recommendations do you meet?

____

____

Which recommendations need your attention?

____

____

GLB not only can lower your risk of developing diabetes, it is also heart-healthy!

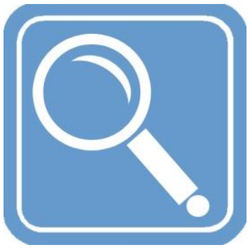

Web sites for heart health information:

- American Heart Association: www.heart.org
- National Heart, Lung and Blood Institute: www.nhlbi.nih.gov
- DASH diet http://www.nhlbi.nih.gov/files/docs/public/heart/dash_brief.pdf

Copyright 2017 University of Pittsburgh

Facts about Cholesterol

Cholesterol is a soft, waxy substance needed by your body to stay healthy. It helps make hormones, Vitamin D, and bile that is used to digest fat. It is part of your cell membranes, nerves, and brain.

Your body makes all the cholesterol it needs. You also get it from the animal foods you eat such as meat, fish, poultry, eggs, and dairy products. There is no cholesterol in the plant foods you eat.

If blood cholesterol is too high, it puts you at high risk for heart disease. It is important to know your numbers. Your health care provider may order a "blood lipid panel test" that measures:

- Total cholesterol (the sum of LDL, HDL and triglycerides in your blood)

- LDL (low-density lipoprotein) cholesterol
    - It is called the "bad cholesterol" because it carries cholesterol to the arteries. Think "L" for "lousy".
    - High levels can lead to blocked arteries.

- HDL (high-density lipoprotein) cholesterol
    - It is called the "good cholesterol" because it removes cholesterol from arteries and takes it to the liver for removal. Think "H" for "healthy".
    - It helps prevent blocked arteries.

- Triglycerides
    - It is the most common type of fat found in your blood.
    - High levels may increase the risk of heart disease.
    - High levels are often found in people who have high cholesterol, heart disease, or diabetes. It is also found in those who are inactive, overweight, smoke, or have a high intake of alcohol and/or sugary foods and drinks.

Your health care provider will assess your risk and overall health and the best way to manage your cholesterol.

Copyright 2017 University of Pittsburgh

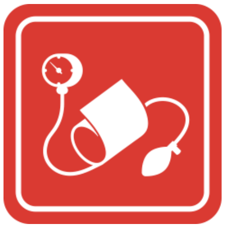

Facts about Blood Pressure

Blood pressure is the force exerted against the walls of the blood vessels as blood flows through.

Blood pressure is measured with two readings:

- Systolic is the upper number. It is the pressure against the walls of the arteries when the heart contracts (beats).
- Diastolic is the lower number. It is the pressure against the walls of the arteries when the heart is at rest between beats.

High blood pressure is called the "silent killer". There are usually no symptoms to warn you something is wrong.

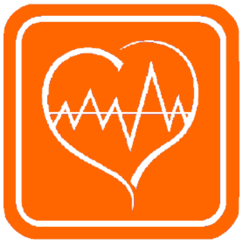

Tips to Control Cholesterol and Blood Pressure

Heath care providers often encourage their patients to first try to lower cholesterol and blood pressure with healthy lifestyle changes. Talk to your health care provider about the following:

- Maintain a healthy weight.
    - This improves LDL and HDL cholesterol, triglycerides, and blood pressure.
- Get active.
    - Move more in your day. Keep up with your physical activity program and limit the time you spend sitting.
    - This improves cholesterol, triglycerides, and blood pressure.
    - Being active enhances your immune system and decreases the risk of heart disease.
- Get enough sleep.
    - Too much or too little sleep can increase the risk of cardiovascular problems.
    - Poor sleep quality is linked to an increased risk of high blood pressure.
    - Aim for about 6-8 hours of sleep per night.

Copyright 2017 University of Pittsburgh

- If you use tobacco, quit. Avoid tobacco smoke.
    - This may improve HDL cholesterol and blood pressure.

- Eat a healthy diet.
    - This may improve triglyceride, HDL and LDL cholesterol levels, and blood pressure.

- Know your fats (see page 8).
    - Limit saturated and trans fats.
    - Replace saturated and trans fats with healthier unsaturated fats. This may improve blood cholesterol and triglycerides.

- Limit added sugars.
    - Too much sugar in your diet increases your risk of obesity, high blood pressure and high cholesterol
    - Reducing the intake of alcohol and sugary foods and drinks may help lower triglycerides.

- Reduce salt intake.
    - This may be recommended to help manage blood pressure.
    - Consider the DASH diet.

DASH diet (Dietary Approaches to Stop Hypertension)

- The DASH diet increases the intake of the minerals calcium, potassium, and magnesium by including more fruit, vegetables, whole grains, legumes, nuts, and low-fat dairy products.
- It recommends a moderate fat intake with unsaturated fats used in place of saturated fats.
- The diet limits saturated fat and trans fats, sodium, added sugar, sugar-sweetened beverages, and alcohol.
  www.nhlbi.nih.gov/health/public/heart/hbp/dash/new_dash.pdf

If your lifestyle changes don't reduce your risk enough, your health care provider will likely prescribe medications. It is important to take them as prescribed.

However, lifestyle changes are still important even when medications are prescribed. Do all that you can to stay heart-healthy.

Copyright 2017 University of Pittsburgh

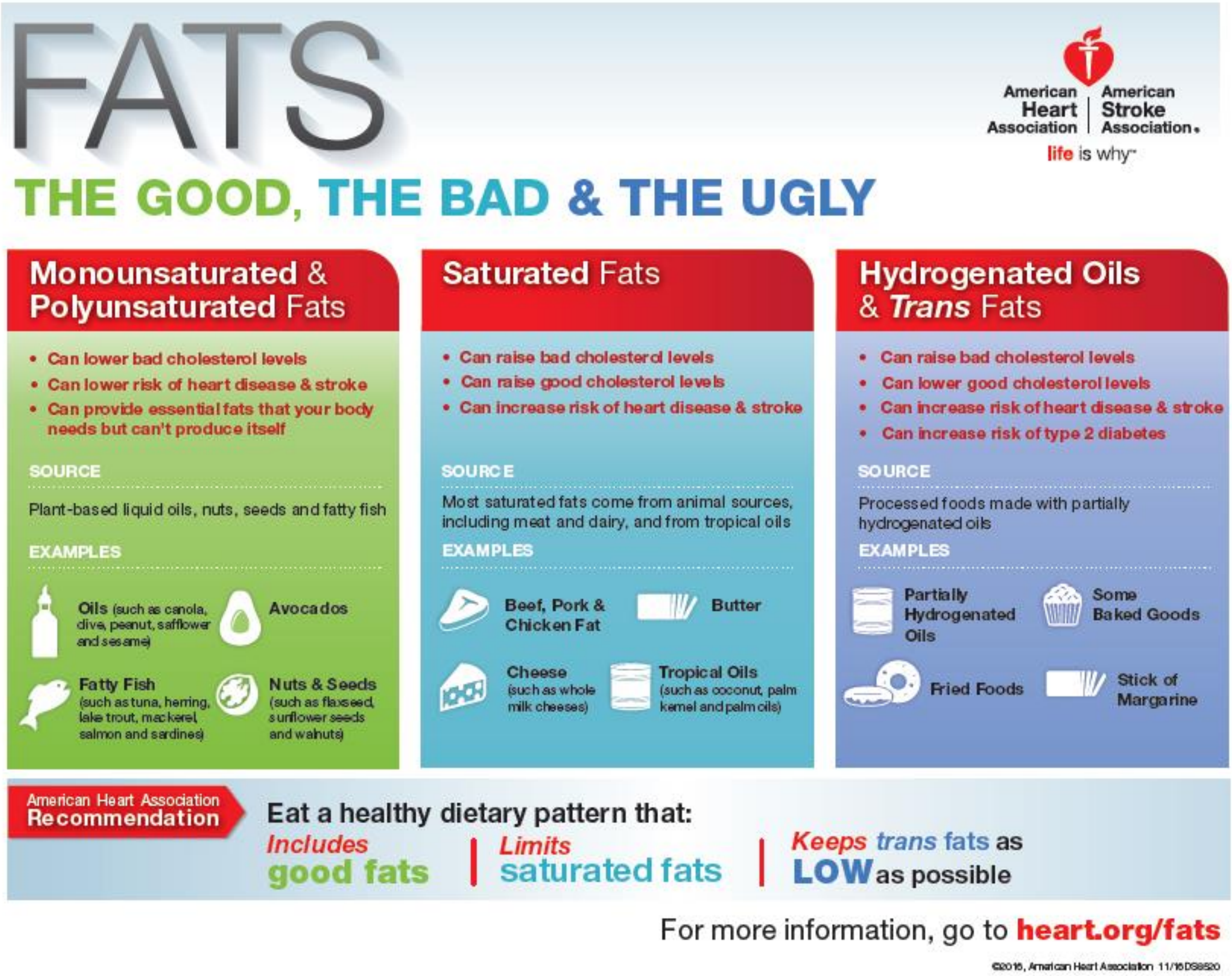
FATS
THE GOOD, THE BAD & THE UGLY
American Heart Association | American Stroke Association.
life is why
Monounsaturated & Polyunsaturated Fats
• Can lower bad cholesterol levels
• Can lower risk of heart disease & stroke
• Can provide essential fats that your body needs but can't produce itself
SOURCE
Plant-based liquid oils, nuts, seeds and fatty fish
EXAMPLES
Oils (such as canola, olive, peanut, safflower and sesame)
Avocados
Fatty Fish (such as tuna, herring, lake trout, mackerel, salmon and sardines)
Nuts & Seeds (such as flaxseed, sunflower seeds and walnuts)
Saturated Fats
• Can raise bad cholesterol levels
• Can raise good cholesterol levels
• Can increase risk of heart disease & stroke
SOURCE
Most saturated fats come from animal sources, including meat and dairy, and from tropical oils
EXAMPLES
Beef, Pork & Chicken Fat
Butter
Cheese (such as whole milk cheeses)
Tropical Oils (such as coconut, palm kernel and palm oils)
Hydrogenated Oils & Trans Fats
• Can raise bad cholesterol levels
• Can lower good cholesterol levels
• Can increase risk of heart disease & stroke
• Can increase risk of type 2 diabetes
SOURCE
Processed foods made with partially hydrogenated oils
EXAMPLES
Partially Hydrogenated Oils
Some Baked Goods
Fried Foods
Stick of Margarine
American Heart Association Recommendation
Eat a healthy dietary pattern that:
Includes good fats
Limits saturated fats
Keeps trans fats as LOW as possible
For more information, go to heart.org/fats
©2016, American Heart Association 11/16DS9520

Make heart-healthy choices:

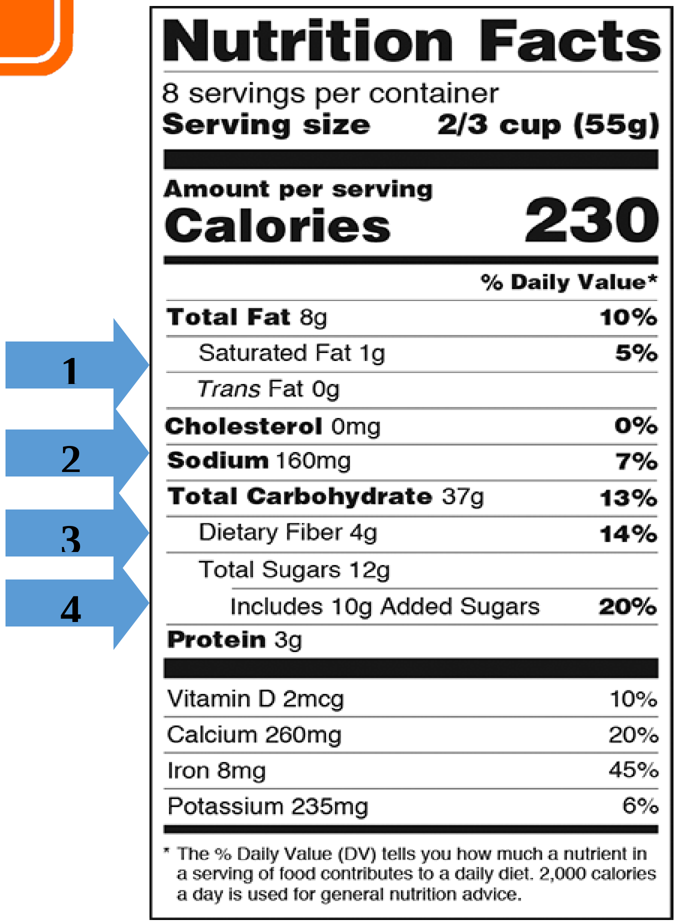

Use the label to help you make heart healthy choices*.

Recommended choices are those that:	Changes I can make:
1. Have the least amount of unhealthy saturated and trans fat.	
2. Are lower in sodium. (Aim for less than 2300 mg per day.)	
3. Are higher in fiber. (Foods with 3 grams of fiber/serving are a good source of fiber; those with 5 grams or more are an excellent source.)	
4. Have less added sugar.	

*These reflect the 2015 Dietary Guidelines

Copyright 2017 University of Pittsburgh

To Do:

The Basics:

- ☐ Keep track of your weight.
- ☐ Record what you eat and drink using your preferred method. Come as close as you can to your calorie and fat gram goals.
- ☐ Record your physical activity using your preferred method. Come as close as you can to your activity goals.
  - Be physically active for at least 150 minutes per week.
  - Aim for an average of at least 50,000 steps per week.
  - Include resistance training at least 2 times per week.
- ☐ Sit less. Be aware of long periods of sitting and break them up. Keep moving throughout the day.

What's New:

- ☐ Know your numbers. Do your blood pressure, blood glucose, blood cholesterol and triglyceride levels fall within the recommended guidelines?
- ☐ What is one heart-healthy change you are willing to make?

  ____

- ☐ Check the Nutrition Facts labels for the amount of saturated fat, trans fat, sodium, fiber, and added sugars. Use this information to help you make heart-healthy food choices.

Copyright 2017 University of Pittsburgh

Session 21 Leader Guide:
Heart Health

Objectives
In this session, the participants will:

- Identify the leading cause of death in American adults.
- Define "heart disease".
- Learn the risk factors for heart disease.
- Discuss how eating a healthy diet and being physically active affect the risk factors for heart disease.
- Define terms related to blood cholesterol.
- Review the healthier fats to include and the unhealthier fat to limit as part of a healthy eating pattern.
- Define terms related to blood pressure.
- Discuss the American Heart Association's "Life's Simple 7® lifestyle changes to improve heart health.
- Discuss ways to use the Nutrition Facts label to make heart-healthy food choices.
- Identify changes they can make to have a heart healthy eating pattern.

To Do Before the Session:

- If providing Food and Activity books, have a supply available for participants.
- Prepare Session 21 handout for participant notebooks.
- Review Food and Activity records; add comments.
- If collecting **Resistance Training Logs** and/or **Sit Less Trackers**; add comments.
- Print copies of the handout, **When You Reach Your Weight Goal** to review with participants, as they achieve their weight goals.
- Print copies of the **Group Lifestyle Balance Monthly Calendar**; have a supply available for participants.
- If providing **Resistance Training Logs;** have a supply available for participants.
- If providing **Sit Less Trackers**; have a supply available for participants.
- Given the array of self-monitoring tools provided, determine your protocol for review and feedback to the participants. Note: As session frequency decreases, the number of records returned at each session may increase.
- Prepare information about local smoking cessation programs to distribute to interested participants.
- Optional: Purchase food and small snack baggies for participants to make healthy trail mix in class. Another option is for you to make and give each participant a snack bag of trail mix that you prepared. Make a label that includes ingredients and nutrition information. See page 7 for a suggested trail mix recipe.
- Optional: Review information from the American Heart Association website. http://www.heart.org/HEARTORG/

Copyright 2017 University of Pittsburgh

Available in the DPP-GLB Providers Portal:
https://www.diabetesprevention.pitt.edu/my/login.aspx

- **GLB Resistance Training Log**
- **When You Reach Your Weight Goal Handout**
- **Group Lifestyle Balance Monthly Calendar**
- **Sit Less Tracker**
- **Guidelines for Reviewing Food and Activity Records**

*[**Note:** For those participants who have reached their goal weight, review with them the handout entitled **When You Reach Your Weight Goal**. This may be done before or after the group meeting.]*

[Do NOT distribute Session 21 handout before class starts.]

Group Sharing Time (allow at least 5 minutes)

Let's take a few minutes to see how things went since our last meeting.

Were you able to apply what you learned at the last lesson to something in your life? If yes, how?

[Problem solve with the participants to address any barriers.]

[Praise all efforts to self-monitor and to change eating and activity behaviors. Be positive and nonjudgmental. Encourage group discussion.]

Heart Health

What is the number one killer of men in the United States? ***[Answer = heart disease]***

What is the number one killer of women in the United States? ***[Answer = heart disease]***

What is the leading cause of death in the world? ***[Answer = heart disease]***

This is an important topic. Today we will be talking today about the ways you can prevent or manage heart disease.

Let's start with a short quiz to see how "heart smart" you are:

1. How big is your heart? Is it the size of your eye, fist, head, or foot? ***[Answer = Fist]***

2. How many times does the average heart beat per minute? 20, 50, 70, or 120 beats per minute? ***[Answer = 70 beats per minute]***

3. If a person doesn't exercise and is out of shape, would his/her heart beat faster or slower than 70 beats per minute? *[**Answer – faster than 70 beats per minute.** The heart is a muscle, in fact, it is the most important muscle in the body. If someone doesn't do regular aerobic exercise such as brisk walking, the heart isn't as strong and has to work harder to pump blood through the body. So it has to beat more than the average 70 beats per minute.]*

4. As a person exercises and becomes more fit, does the heart beat faster or slower? *[**Answer = Slower.** When someone does aerobic exercise such as brisk walking, the heart muscle becomes stronger and can pump more blood with each heartbeat. Athletes often have resting heart in the 40s because their heart muscles are so strong.]*

5. When does the heart rest? When you are sleeping, when you are sedentary like while watching TV, between beats, or it never rests and is always working? *[**Answer = between beats.** This adds up to about 5 hours of resting time per day for the average person. Someone who doesn't exercise and is overweight and out of shape would have less rest time because their heart has to beat more times per minute than average. Doing regular aerobic exercise (similar to brisk walking) makes the heart muscle stronger and more efficient. It beats fewer times per minute which allows for more rest time between beats.]*

People with pre-diabetes and/or metabolic syndrome are at a higher risk for developing heart disease. What are some other risk factors?

Today's lesson will focus on heart disease and how living a healthy lifestyle can reduce your risk. According to the American Heart Association, a heart healthy lifestyle can reduce the risk of heart disease by as much as 80%.

[Distribute handout and review page 1. Include the following:]

The chart on page 1 lists the risk factors for heart disease. High cholesterol, high blood pressure, diabetes, overweight/obesity are all part of metabolic syndrome.

Notice that there are more risk factors that can be changed than those that can't be changed. Research suggests that about 70% of all heart disease could be prevented if people addressed their risk factors.

It is important to know your family history. Ask family members if you don't know. Tell your children and grandchildren if heart disease runs in your family. Even if heart disease runs in the family, a heart-healthy lifestyle can reduce the risk. Don't wait to get heart disease and treat it; do all you can to prevent it.

Copyright 2017 University of Pittsburgh

[Ask participants to list their risk factors.]

Preventing or Managing Heart Disease with "Life's Simple 7™" (pages 2-4)

[Review pages 2-3 and include the following:]

1. **Get Active** – Notice that the physical activity goals you are asked to achieve in GLB are the same as the American Heart Association (AHA) recommendations. Research demonstrates that a low fitness level increases the risk of heart disease independent of body weight.

2. **Control Cholesterol** - How many of you have had your cholesterol tested? Do you know the results? This is an important conversation to have with your health care provider. We will talk more about cholesterol in a few minutes.

3. **Eat better** – Notice that the healthy eating pattern recommended by GLB and the AHA are the same.

4. **Manage blood pressure** – High blood pressure is also known as hypertension. We will talk more about this in a few minutes. How many of you know your most recent blood pressure? Every time someone takes your blood pressure, ask for the results and write it down. This is an important conversation to have with your health care provider.

5. **Lose weight and keep it off**
   - Reaching and maintaining your GLB weight loss goal will help reduce the risk of heart disease and other health issues.
   - Research demonstrates that as weight and physical activity improve, so do the risk factors for heart disease. But the risk factors for heart disease increase if there is a negative trend in weight and activity.

6. **Reduce blood sugar (glucose)** - The goal is to achieve and maintain blood glucose levels that are normal or as close to normal as possible. Have a conversation with your health care provider about your risk for diabetes. If you have pre-diabetes or diabetes, work closely with him/her to manage your blood glucose.

7. **Don't smoke and avoid secondhand smoke** - Smoking causes plaque to build up in the arteries more quickly. The good news is that the risks start to decrease as soon as someone stops smoking. *[Explain that you have information about local smoking cessation programs available for interested participants.]*

[Review and discuss the information on page 4. Ask participants to answer the questions and share their responses. Encourage discussion.]

[Encourage interested participants to visit the websites for heart health information.]

Copyright 2017 University of Pittsburgh

Facts about Cholesterol (page 5)

People are often surprised to learn that the body actually needs cholesterol.

[Review the information on page 5 and include the following:]

It is important to know your total blood cholesterol level, as well as your blood levels for LDL, HDL, and triglycerides. Speak with your health care provider about having a blood lipid panel test, or ask for the results if you have already had it tested.

Facts about Blood Pressure/Tips to Control Cholesterol and Blood Pressure

[Review Facts about Blood Pressure and include the following:]

It is important to have your blood pressure checked on a regular basis.

When blood pressure is high, it creates too much force against the artery walls. This can cause damage to the arteries.

When blood pressure stays within healthy ranges, it reduces the strain on your heart, arteries, and kidneys which is important for health.

The good news is that there are lifestyle changes you can make to help control your cholesterol and blood pressure.

[Review Tips to Control Cholesterol and Blood Pressure on pages 6 and 7.]

Get enough sleep. Adequate sleep not only helps your heart, it also has a positive effect on your stress hormones, immune system, metabolism, and mental status.

If you use tobacco, quit. Smoking damages your entire circulatory system and increases the risk of heart disease.

Eat a healthy diet. Following the recommendations of the Dietary Guidelines and MyPlate will help you have a heart healthy eating pattern.

- One of the Dietary Guidelines is to eat less added sugar and the American Heart Association agrees because studies suggest that getting too much added sugar in the diet may contribute to high blood pressure and high cholesterol.
- Eating a diet high in fiber helps reduce the risk of heart disease.

Know your fats: In Session 3 we discussed the healthy unsaturated fats and the unhealthy saturated and trans fats.

Page 8, from the AHA website, has a nice summary of the different fats.

Copyright 2017 University of Pittsburgh

- Replace unhealthy saturated and trans fat with healthier unsaturated fats because, according to a June 2017 advisory from the AHA, it helps reduce the incidence of heart disease. The advisory reports that this lowers heart disease risk as much as cholesterol-lowering statin drugs (about 30% reduction).
  - The healthy fats do not raise “lousy” LDL cholesterol and may help raise the “healthy” HDL cholesterol.
  - Unsaturated fats, including Omega-3 fatty acids, appear to have an anti-inflammatory affect. This may lower the risk of heart disease.

  [Note: As of June 2017, the AHA continues to recommend against eating coconut oil because of its high saturated fat content that raises LDL cholesterol.]

- Be careful not to get too much of a good thing. The AHA recommends that you don’t exceed 35% of your total daily calories from healthy types of fat. The GLB fat gram goal is about 25% of daily calories. Be aware of calories.

If interested, visit the AHA website for more information and updates.

[Note: Based on the latest scientific evidence, the 2015 Dietary Guidelines do not include the previous recommendation to limit cholesterol intake to less than 300mg/day. Many foods that contain cholesterol are also high in saturated fat, so limiting saturated fat may also limit cholesterol intake.]

Reduce salt intake. Higher salt intake puts you at risk for high blood pressure. About 75% of the salt in the average American diet comes from salt added to processed food and restaurant food. Control and cut out salt where you can.

DASH diet

- The DASH diet lowered blood pressure as quickly and as much as medication. It is important to follow all the DASH diet recommendations. Just taking a supplement of calcium, magnesium, and potassium did not lower blood pressure.
- The website has a lot of information, including meal plans.

When people are diagnosed with high blood cholesterol levels or high blood pressure, their health care provider will assess their risk and overall health and the best way to manage it. Health care providers often suggest they first try to lower their cholesterol or blood pressure with heart-healthy lifestyle changes. For some people, this will reduce their cholesterol or blood pressure. For others, they may also need to take prescription medication. The heart-healthy lifestyle changes are important while taking medications.

Make heart-healthy choices (page 9)

The Nutrition Facts labels can be used to help you follow the recommendations of the 2015 Dietary Guidelines and to make heart healthy food choices.

Copyright 2017 University of Pittsburgh

[Ask the participants to locate on the sample label the 4 facts: grams of saturated and trans fat, milligrams of sodium, grams of dietary fiber, and grams of added sugar (4 grams equal about 1 teaspoon). Make sure they are able to locate this information on the sample label.]

In the box, jot down a few changes you think you can make to help you shift to more heart healthy food choices.

Optional: Give participants a bag of trail mix you prepared ahead of time. Or…
Optional: Have participants make a heart-healthy trail mix. Set out on a table:

- Disposable plastic gloves; one pair for each participant
- Snack size baggies; one per participant
- Bowl of whole grain cereal. Either use
    - Cheerios™ with ½ cup measuring cup (1/2 cup per bag) OR
    - Kashi® Heart to Heart® cereal (1/4 cup per bag)
- Bowl of almonds (9 almonds per bag).
- Bowl of dried blueberries with 1 Tablespoon measuring spoon (1 Tablespoon per bag).
- Bowl of dried cherries with1 Tablespoon measuring spoon (1 Tablespoon per bag).
- Have printed labels with the recipe and nutrition information

	**Calories**	**Fat (grams)**	**Fiber (grams)**
¼ cup Kashi® Heart to Heart® cereal	40	.5	1.6
1 Tablespoons each of dried blueberries and cherries	70	0	2
9 almonds	69	6	1.4
**Totals**	**179**	**6.5**	**5**

To substitute Cheerios™ in place of Kashi®, use ½ cup which provides 50 calories, 1 gram of fat, and 1.5 grams of fiber. Adjust "Totals" accordingly.

This trail mix contains:

- Whole grain for healthy carbohydrates and fiber.
- Nuts for protein, healthy unsaturated fat, and fiber.
- Dried fruit for healthy carbohydrate, fiber, and natural sweetness. Most people have eaten raisins and craisins, so this is an opportunity to try different dried fruits.

Plant foods naturally provide many healthy antioxidants and phytochemicals.

To Do (page 10)

Turn to page 10 and let's focus on what you can do between now and the next session.

Copyright 2017 University of Pittsburgh

[Review and discuss. Encourage participants to take what they learned in today's group meeting and apply it. See if it helps them in their lifestyle change efforts.]

[Review this page. Encourage participants to experiment with ways to make shifts to more heart healthy food choices.]

[Encourage interested participants to take a copy of the information about smoking cessation programs available in your area.]

[Announce the day, time, and place for the next session.]

After the session:

- Weigh participants who did not do so prior to the group meeting.
- Complete data forms and documentation required in your setting.
- Follow your program's protocol for managing absences.
- Follow your program's protocol for reviewing and commenting on the various self-monitoring records.
- Review the self-monitoring records. Write brief comments. Be positive and nonjudgmental.
    - Praise all efforts to self-monitor and to change eating and activity behaviors.
    - Highlight any positive changes made that relate to the topic of the last session before the records were collected.
    - Refer to **Guidelines for Reviewing Food and Activity Records** available in the GLB Providers Portal.
- If collecting **Resistance Training Logs** and/or **Sit Less Trackers,** review them. Write brief comments. Be positive and encouraging.

Copyright 2017 University of Pittsburgh

Session 22: Look Back and Look Forward

There's an old saying, "A path is made by walking on it." Research suggests that people who succeed in changing their lifestyle, shift their thinking and behavior along the way. What path did they take?

Move From:	Toward:
Trying to fool themselves	Being honest with themselves<br>*(I consume a lot of calories in my night-time snacks)*
Looking for a "magic" cure	Working at it, tolerating some discomfort<br>*(I take the stairs instead of the elevator even* ***when I don't*** *feel like it)*
Looking for a "cookbook" approach that applies to everyone	Fitting tried-and-true ways of behavior change into their own lifestyle and culture<br>*(I ask my aunt to give me just a small piece of her special cake)*
Wishing someone else could fix or take the blame for lifestyle barriers	Taking "sole responsibility" for doing or not doing what needs to be done<br>*(I had to* ***stay with dad at the doctor's*** *office all day but I took breaks from sitting and walked the hallways)*
Thinking of weight loss and activity totals (numbers on the scale and activity record) as your only goals	Focusing on the pleasure that comes with every small change in eating better and moving more<br>*(It feels great not to be winded after climbing one flight of stairs)*
Being afraid of slips and lapses or punishing themselves when they happen	Believing they can make mistakes and learn from them. Being willing to try, try, and try again<br>*(That buffet was hard. I will make a better plan for next time)*

Copyright 2017 University of Pittsburgh

What path have you been on now? Think about some of the "shifts" you have made in your lifestyle and write them down.

I have moved from:	Toward:
Wanting to do it perfectly right away	Being willing to take *small steps* toward my most valued goals
My changes:	
Wishing for "willpower" made of steel	Being flexible, mindful, and trusting myself to *take charge* more often
My changes:	
Putting others first most of the time	Working on *healthy self-interest* even though it sometimes conflicts with needs and demands of others
My changes:	

Congratulations. You have reached an important one-year milestone in Group Lifestyle Balance™. You have worked hard to improve your eating and physical activity habits.

You know that healthy lifestyle change is possible.

Take a moment and give yourself credit for all your efforts.

Copyright 2017 University of Pittsburgh

Look Back:
Write Your Story

Take a minute to reflect on your own healthy lifestyle story. What change strategies worked best for you? What changes are you most proud of?

____

____

____

Imagine you are speaking with others at risk for type 2 diabetes and heart disease or those who simply want to take better care of themselves by eating healthier and moving more. What words of wisdom could you share?

____

____

____

What helped you the most when you faced challenges, or felt down about your progress? Were there specific people (including family and friends) who helped you move forward?

____

____

____

Write down a message to yourself to help motivate and guide you in the next six months.

____

____

____

Now that you have reflected on your personal lifestyle change journey, complete the checklist on the next page.

Copyright 2017 University of Pittsburgh

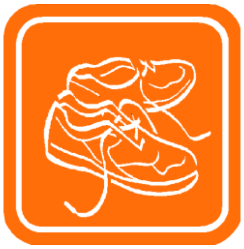

Look Forward: Write Your Next Chapter

Given where you have been, and where you are right now, what are your plans looking forward?

My Weight Goal

In the next 6 months I will focus on:

- ☐ Reaching the 7% weight loss goal.
- ☐ Staying at the 7% weight loss goal.
- ☐ Working on a personal weight loss goal.

Refer to **"When You Reach Your Goal Weight"** in Session 14, page 11 for more information.

My Physical Activity Goals

In the next 6 months I will focus on:

- ☐ Reaching the 150 minutes per week goal.
- ☐ Staying at 150 minutes per week goal.
- ☐ Working on a personal physical activity goal of _____ minutes per week.
- ☐ Adding spontaneous physical activity.
- ☐ Working on a step goal of _______________
- ☐ Doing resistance training activities at least 2 times per week.
- ☐ Reducing and breaking up time spent sitting.

Refer to Session 4, 8, 13, 17, and 19 for activity tips.

Copyright 2017 University of Pittsburgh

To Do:

These behaviors are your foundation for healthy lifestyle success.
Let’s review them one more time.

The Basics:

- Keep track of your weight. Make peace with the scale.
  - Weigh yourself daily (or at least once/week, same time of day)
  - Keep a weight record. This will allow you to monitor and respond to weight gain sooner rather than later.

- Record what you eat and drink using your preferred method. If you notice your weight creeping up, keep track more often.

- Record your physical activity using your preferred method. Consider tracking minutes, steps and resistance training. Aim to maintain the minimum goal of 150 minutes per week of moderate physical activity.

- Sit less. Be aware of long periods of sitting and break them up. Keep moving throughout the day.

- Get the support you need. List 1-3 people who will support your healthy lifestyle efforts on the road ahead.

You have the tools that you need for healthy lifestyle success.
You have learned through trial and error what works best for you on the slippery slope of lifestyle change. You know how to get back on track after a slip.

Now your challenge is to keep using those skills. Don’t let them get rusty. Stay aware. Solve problems. Practice.

It has been a pleasure to support you.

> “Success is the sum of small efforts, repeated day in and day out”
>
> ~Robert Collier

Copyright 2017 University of Pittsburgh

Session 22 Leader Guide:
Look Back and Look Forward

Objectives
In this session, the participants will:
- Discuss the shift in thinking patterns that may be integral to a successful weight loss experience.
- Describe the behaviors that have been demonstrated to characterize individuals who are able to maintain weight loss over the long term.
- Look back on their own "personal healthy lifestyle story". Identify positive changes and ongoing challenges.
- Identify key lessons that they believe to be most important to share with others who learn they are at risk for diabetes and/or cardiovascular disease.
- Discuss the "foundation behaviors" that are most critical to ongoing lifestyle weight management success.
- Acknowledge that this is the final session of their group work together and discuss thoughts and feelings related to looking ahead at their ongoing lifestyle weight management efforts.
- Look ahead to the next 3-6 month period. Make a plan for eating and physical activity.

To Do Before the Session:
- If providing Food and Activity books, have a supply available for participants.
- Prepare Session 21 handout for participant notebooks.
- Review Food and Activity records; add comments.
- If collecting **Resistance Training Logs** and/or **Sit Less Trackers**; add comments.
- Print copies of the handout, **When You Reach Your Weight Goal** to review with participants, as they achieve their weight goals.
- Print copies of the **Group Lifestyle Balance Monthly Calendar**; have a supply available for participants.
- If providing **Resistance Training Logs;** have a supply available for participants.
- If providing **Sit Less Trackers**; have a supply available for participants.
- Determine if you will provide extra copies of self-monitoring materials for participants to take with them to use after GLB ends.

Available in the DPP-GLB Providers Portal:
https://www.diabetesprevention.pitt.edu/my/login.aspx
- **GLB Resistance Training Log**
- **When You Reach Your Weight Goal Handout**
- **Group Lifestyle Balance Monthly Calendar**
- **Sit Less Tracker**
- **Guidelines for Reviewing Food and Activity Records**

Copyright 2017 University of Pittsburgh

*[**Note:** For those participants who have reached their goal weight, review with them the handout entitled **When You Reach Your Weight Goal**. This may be done before or after the group meeting.]*

Group Sharing Time (allow at least 5 minutes)

Let's take a few minutes to see how things went since our last meeting.

Were you able to apply what you learned at the last lesson to something in your life? If yes, how?

[Problem solve with the participants to address any barriers.]

[Praise all efforts to self-monitor and to change eating and activity behaviors. Be positive and nonjudgmental. Encourage group discussion.]

Look Back and Look Forward (pages 1-2)

[Review and discuss information on page 1.]

As we have discussed, making lifestyle changes for weight management involves an ongoing self-review process. This means you need to "look back" (on old ways, or habits, of thinking and doing) and to "look forward" (on new ways of approaching pro-active lifestyle behavior change).

For many people, the "new" thinking is characterized by honest self awareness, personal responsibility for behavior change, a willingness and openness to figuring out what works, and persistence in the face of lapses.

Look back. What are some of your old "thinking traps" that did not result in effective self-management of a healthy lifestyle?

[Review and discuss the different categories of ineffective thinking and ask the group members to identify (and share examples) of the ways in which they have been able to move away from these perspectives and work towards newer, more useful approaches.]

Now let's look at where you are now. What are some "shifts" you have made? *[Review the chart on page 2. Ask participants to jot down their answers and encourage group discussion.]*

Which behaviors so you believe are most critical to your own success (or lack of success)?

Copyright 2017 University of Pittsburgh

Look Back: Write Your Story (page 3)

As we come to the close of our last session together, let's take some time to write down your own "lifestyle story". Think about the past year, and the changes that you have made. What are some thoughts and strategies that have been most important and helpful when you have felt discouraged about your lifestyle progress?

Send a "message to yourself for the future". Try to make this a personally powerful statement that will help sustain you during rough times.

[*Ask the group to share their thoughts with the others if they are willing to do so.]*

Look Forward: Write Your Next Chapter (page 4)

Ask group members to consider other types of tracking tools they could utilize as they continue their healthy lifestyle. Encourage the group members to consider continuing these basic practices as needed in the future. In addition, ask group members to think about what kinds of formal (e.g. other community based lifestyle management programs, web-based resources or physical activity options) or informal networks (e.g. natural social supports such as friends, family and coworkers) they can access if and when they need it.

As you go forward, I encourage you to continue using all of the tools, skills and strategies that you have learned and practiced over the past year. Remember the importance of weighing yourself on a regular basis and monitoring your food intake and activity.

How do you plan to self-monitor now that GLB is over. You could use a notebook or journal, or one of the many digital tracking tools that are available online. Ask your insurance providers if they offer paper or online self-monitoring tools. While you may not need to monitor your food intake and activity on a daily basis, it may be helpful to monitor during difficult times or if you feel you are slipping. You may want to refer back to the Gold, Silver, and Bronze tracking options we discussed in Session 12.

Why do you want to stay active, eat a healthy diet, and manage your weight? Is your answer now the same or different than when you started GLB? In what ways?

At this point, some of you would like to focus on continued weight loss. Others have reached their goal weight and would like to focus on weight loss maintenance. *[Ask participants who have been maintaining their weight to share their plan and to offer suggestions and advice.]*

Copyright 2017 University of Pittsburgh

Former participants have reported that they found it helpful to review session handouts. Some found it helpful to review their old Food and Activity records.

To Do (page 5)

Please turn to page 5 and let's review the behaviors that are the foundation for healthy lifestyle success. *[Review page 5.]*

You have the tools you need for success in maintaining your healthy eating and activity behaviors. You know what you must do to succeed, and your lifelong learning challenge will be keeping these skills ready and available even when you may find yourself on the slippery slope.

It has been a pleasure working with you this year and I wish you the best of health as you go forward. Remember that you have the tools that you need to be successful in staying healthy, and that you can do it!

After the session:

- Weigh participants who did not do so prior to the group meeting.
- Complete data forms and documentation required in your setting.
- Follow your program's protocol for managing absences.
- Follow your program's protocol for reviewing and commenting on the various self-monitoring records.
- Review the self-monitoring records. Write brief comments. Be positive and nonjudgmental.
    - Praise all efforts to self-monitor and to change eating and activity behaviors.
    - Highlight any positive changes made that relate to the topic of the last session before the records were collected.
    - Refer to **Guidelines for Reviewing Food and Activity Records** available in the GLB Providers Portal.
- If collecting **Resistance Training Logs** and/or **Sit Less Trackers,** review them**.** Write brief comments. Be positive and encouraging.
- Return all completed Food and Activity records, Resistance Training Logs, and Sit Less Trackers to the participants.

Copyright 2017 University of Pittsburgh
